# Metal ligand cooperativity in the direct carboxylation and esterification of terminal alkynes by Cu-CNC complexes bearing 2,6-lutidine linkers

**DOI:** 10.1039/d5sc08379f

**Published:** 2026-01-08

**Authors:** Nick Back, Emylie Guthrie, Chengxu Zhu, Sam P. de Visser, Laleh Tahsini

**Affiliations:** a Department of Chemistry, Oklahoma State University Stillwater Oklahoma 74078 USA tahsini@okstate.edu; b Manchester Institute of Biotechnology, The University of Manchester 131 Princess Street Manchester M1 7DN UK; c Department of Chemical Engineering, The University of Manchester Oxford Road Manchester M13 9PL UK

## Abstract

CO_2_ utilization is a significant and emerging field in catalysis, playing a crucial role in reducing atmospheric CO_2_ and mitigating climate change. In this work, we report on Cu(i) complexes that utilize atmospheric CO_2_ for the direct carboxylation and esterification of terminal alkynes. The Cu(i) complexes bear ligands of the type 2,6-bis(3-alkyl/arylimidazol-2-ylidene) methylpyridine I(R)^C^N^C^, where R = ^i^Pr, Me, 2,6-^i^Pr_2_Ph (Dipp), 2,4,6-Me_3_Ph (Mes), and 4-CF_3_Ph. While copper-catalyzed carboxylation reactions are not unprecedented, this work presents the first example of metal ligand cooperativity (MLC) through a dearomatization-aromatization process used in the direct carboxylation of terminal alkynes. It also presents the first dearomatized Cu-CNC complexes that have been crystallographically and spectroscopically characterized. Further investigation using UV-vis spectroscopy revealed the enthalpy and entropy of formation, as well as the activation parameters for the dearomatized [Cu^I^(I(^i^Pr)^C^N^C^)*] complex. This marks the first time such data have been reported for dearomatized-metal-CNC systems. To establish mechanistic details of the reaction, we performed stoichiometric reactions and characterized products with a variety of NMR methods. Combined with supporting computational studies, the work yields several new CNC-supported copper intermediates, including copper-styrenyl, copper-acetylide, and copper-propiolate. While the reactive and labile nature of some of these intermediates precludes their solid-state characterization, DFT-computed structures are consistent with spectroscopic characterization.

## Abstract 2

This work highlights the first structurally characterized dearomatized Cu-CNC complexes and their application as catalysts for the direct carboxylation of terminal alkynes *via* metal–ligand cooperativity (MLC).

## Graphical abstract

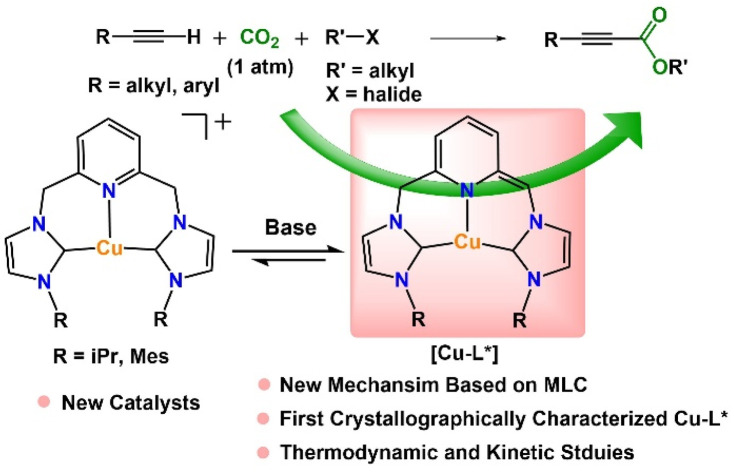

## Introduction

Catalytic carbon dioxide activation and functionalization has emerged as a critical research area. It provides a pathway for carbon recycling and contributes to the synthesis of valuable chemicals and fuels, thereby addressing the dual challenges of energy sustainability and reducing greenhouse gas emissions. The activation of CO_2_ is a critical step in the conversion process due to its thermodynamic stability and kinetic inertness, requiring significant energy input. Over the last two decades, various catalysts, including transition metals,^1–4^ metal–organic frameworks (MOFs),^5,6^ covalent organic frameworks (COFs),^7^ and hybrid systems combining plasma and catalysts,^8^ have been reported for CO_2_ reduction into methanol and hydrocarbons. However, the scope of technologies for converting CO_2_ into fine chemicals *via* direct insertion into C–H bonds has been somewhat limited.

**Scheme 1:**
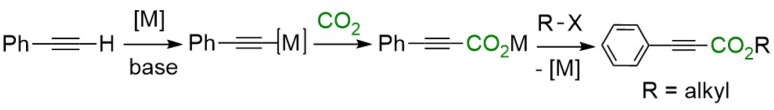
Metal-catalyzed C–H carboxylation and esterification with CO_2_.

In this context, coinage metal-based catalysts have primarily been reported to convert arenes, heteroarenes, and terminal alkynes directly to their corresponding carboxylic acids.^9–16^ The C–H carboxylation of terminal alkynes with CO_2_ yields propiolic acids, valuable intermediates for the chemical and pharmaceutical industries.^17–19^ Although alkyne carboxylation reactions can occur without metals at high temperatures or under ambient conditions,^20–22^ metal catalysts such as copper or silver still dominate this field of research.^23^ The reaction mechanism generally involves coordination of the alkyne to the metal center after initial deprotonation by a base, yielding a metal acetylide that serves as a nucleophile toward CO_2_ (Scheme 1).^24^ A subsequent reaction with an alkyl halide is often performed *in situ* to convert the metal propiolates into the corresponding esters to (i) avoid the dissociation of the carboxylic acids at high temperatures and (ii) render the carboxylation irreversible against the competitive decarboxylation reaction.

In the 2010s, several groups reported the application of molecular copper catalysts in the direct carboxylation of terminal alkynes with CO_2_.^13,25–28^ The copper(i) centers in these complexes were supported by 1,10-phenanthroline, phosphine, and N-heterocyclic carbene (NHC) ligands, with the NHC present in three out of five reported examples. Recently, copper complexes supported by azothioformamide (ATF) ligands were reported as catalysts for the carboxylation of terminal alkynes.^29^ Despite the use of different ligand platforms, the existing catalysts perform through similar CO_2_ and alkyne activation steps.

Previous studies have shown that the [Cu(NHC)(Base)] (Base = OH, O^*t*^Bu, *etc*) is the active form of all complexes, wherein copper(i) is bound to a monodentate NHC ligand.^10,11^ The species is readily formed, *in situ* or isolated, through the displacement of the halide anion trans to NHC in the structure of [Cu(NHC)(X)] complexes. Following the Brønsted–Lowry framework, the [Cu(NHC)(Base)] species can only activate and carboxylate the C–H bonds of the substrates that are more acidic than the catalyst center. This explains the low reactivity of [Cu(IPr)(OH)] (IPr = 1,3-bis(diisopropyl)phenylimidazol-2-ylidene) complex (p*K*_aDMSO_ = 27.7)^11^ in the direct carboxylation of terminal alkynes (p*K*_a_ ∼ 28.8).^30^ Previously, it was shown that the reaction can be performed effectively only under high CO_2_ pressure (1.5 MPa).^27^

Interestingly, switching the coordination environment of copper from monodentate NHC to polydentate NHC ligands improved the catalyst reactivity, such that the P(NHC)_0.5_(NHC-Cu)_0.5_ complex could carboxylate terminal alkynes under atmospheric pressure of CO_2_. The proposed reaction mechanism involved internal activation of CO_2_ by the free NHC arm of the ligand.^13^ Later, the carboxylation mechanism was investigated by theoretical studies of a copper complex containing a pincer bis-NHC ligand as a simpler model. The computational data showed that the most favorable pathway for carboxylation involves activation of CO_2_ by a free NHC arm, followed by chelation of the metal by the carboxylated bis-NHC ligand.^31^ Despite using a reasonably similar ligand, the calculated structure of the Cu-CNC complex reported in the work is quite distinct from those in our study, suggesting a potentially different reaction mechanism (*vide infra*).

**Scheme 2:**
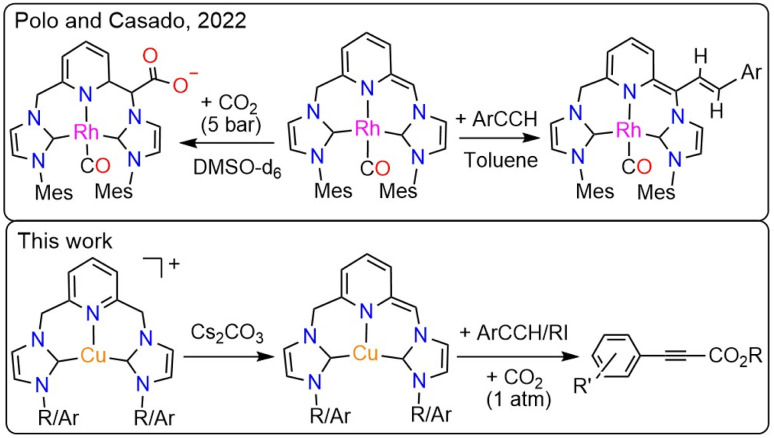
The reported CO_2_ and alkyne activation by a dearomatized Rh-CNC complex and the present work, describing the direct carboxylation of alkynes using Cu-CNC complexes.

The 2,6-dimethylpyridyl-linked bis-NHC ligands resemble the lutidine-derived PNP platforms that have been intensely investigated in catalysis. An essential feature of the PNP-containing catalysts is the “non-innocent” behavior of the ligands that can assist the transition metal in activating the substrates. This mechanism, discovered by Milstein,^32^ is referred to as metal–ligand cooperation (MLC) and operates through the ligand's dearomatization/aromatization process. The MLC was demonstrated for numerous TM-PNP complexes (TM = Ru, Fe, Co, Rh, Ir, Ni, Pd, Pt, and Re), with Ru, Fe, and Re systems serving as versatile catalysts for hydrogenation, dehydrogenation, and, more recently, CO_2_-derived carbonylation.^33–35^ Additionally, several Cu-PNP systems were reported to exhibit electrophilic addition reactivity toward thiols and methyl triflate *via* a dearomatization/aromatization mechanism on a stoichiometric scale. However, the high solubility of the dearomatized species precluded crystallographic characterization.^36,37^ The use of MLC in activating substrates, particularly dihydrogen, was recently extended to lutidine-derived bis-NHC systems, as phosphine rivals, and several Ru-CNC complexes with pronounced reactivity were reported.^38,39^ Interestingly, the catalytic activity of the Ru-CNC complexes in CO_2_ hydrogenation was strikingly lower than that of their Ru-PNP counterpart. Recently, a dearomatized Rh-CNC complex bearing mesityl wingtips was synthesized and characterized crystallographically,^40^ and its reaction with CO_2_ and aryl alkynes was examined individually under stoichiometric conditions (Scheme 2).^41^ To date, no reports have been published on the carboxylation activity of this or any other TM-CNC complex, which requires simultaneous exposure to both terminal alkynes and CO_2_ either stoichiometrically or catalytically. Understanding the fundamental differences between PNP- and CNC-based systems is essential for developing more effective catalysts.

Our group has developed several copper complexes containing 2,6-dimethylpyridyl-linked bis-NHC (CNC) ligands,^42,43^ Herein, we report the use of some of these complexes and new Cu-CNC^Ar^ (Ar = Mes, Dipp) systems as catalysts for the direct carboxylation of terminal alkynes with CO_2_ under atmospheric and sub-atmospheric pressures. We also report detailed mechanistic studies of the carboxylation reaction using multiple spectroscopic methods and DFT. This study presents the first spectroscopic and crystallographic characterization of a dearomatized Cu-CNC complex, as well as the role of MLC in the direct carboxylation of terminal alkynes, distinguishing it from previously reported Rh- and Ru-CNC analogs.^40,44,45^

## Results and discussion

### Synthesis of the Cu-CNC complexes

**Fig. 1:**
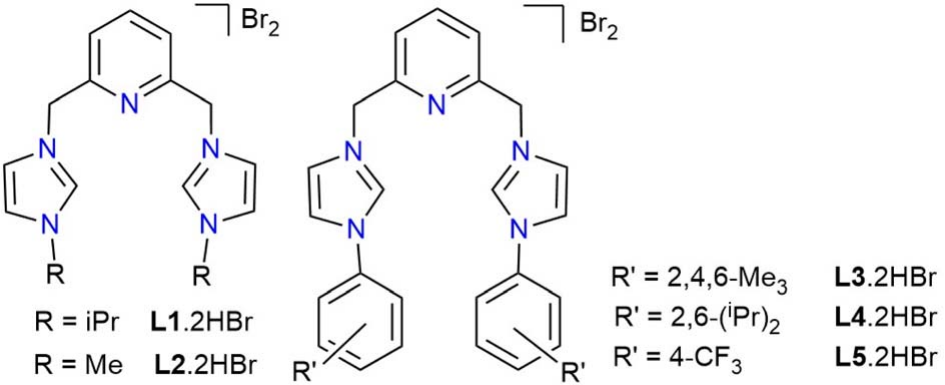
The pincer CNC^R^ and CNC^Ar^ ligand precursors, R = ^i^Pr (L1.2HBr), Me (L2.2HBr), Ar = Mes (L3.2HBr), Dipp (L4.2HBr), and p-CF_3_Ar (L5.2HBr).

2,6-Dimethylpyridyl-linked bis-imidazolium salts of ligands L1–L5 were prepared according to published procedures (Fig. 1).^42,43^ The 2-(4-trifluoromethylaryl)-1-*H* imidazole precursor was synthesized through a Ullman-type C–N coupling reaction catalyzed by the [Cu(I^i^Pr^C^N^C^)]PF_6_ complex.^46^ The new imidazolium salts, L3.2HBr and L4.2HBr, were characterized by ^1^H and ^13^C NMR spectroscopy in DMSO-*d*_6_ and elemental analysis. The corresponding Cu-CNC complexes were prepared at room temperature by reacting [Cu(CH_3_CN)_4_PF_6_] with the *in situ* generated CNC ligand through either the method of subsequent addition or combining all solid reactants.^43^ The new copper complexes, [Cu(IMes^C^N^C^)]PF_6_ ([Cu-L3]PF_6_) and [Cu(IDipp^C^N^C^)]PF_6_ ([Cu-L4]PF_6_), were characterized by NMR spectroscopy, elemental analysis, and X-ray crystallography. In addition, the [Cu-L1]BArF and [Cu-L3]BArF complexes were synthesized as more soluble derivatives for mechanism studies and were characterized by NMR spectroscopy and elemental analysis. The pattern of the ^1^H and ^13^C{^1^H} NMR spectra of these complexes in CD_3_CN resembles that of their analogs with alkyl and para-substituted aryl wingtips.^42,43^ The fluxional processes relating both arms of the pincer ligand under ambient conditions lead to sharp singlets for the methylene linkers in the ^1^H NMR spectra of these complexes. It was previously shown that the conformational exchanges at the pyridinic hydrogens of the Cu-CNC complexes occur too rapidly to be detected by variable-temperature (VT) NMR spectroscopy, even at 193 K.^43^

### X-ray crystallography of Cu-CNC complexes

**Fig. 2:**
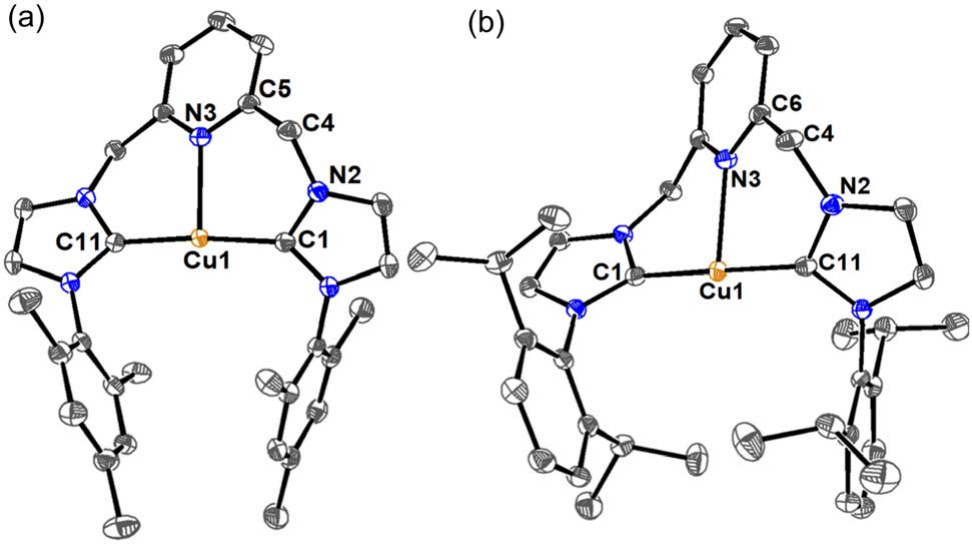
ORTEP diagrams of (a) [Cu-L3]PF_6_ and (b) [Cu-L4]PF_6_. Anions, hydrogen atoms, and solvent molecules have been omitted for clarity. Ellipsoids are shown at the 60% probability level.

Single crystals of complexes [Cu-L3]PF_6_ and [Cu-L4]PF_6_ were obtained at room temperature through the slow diffusion of diethyl ether into a solution of these complexes in acetonitrile. Crystallographic data collection parameters and selected bond distances and angles are shown in Tables S1 and S2, respectively. In the crystal structure of both complexes, each copper is bound to two NHC rings from the same CNC unit, supporting the chelating coordination mode of the ligand (Fig. 2). The Cu⋯N_py_ distances of 2.221(1) Å in [Cu-L3]^+^ and 2.269(1) Å in [Cu-L4]^+^ resemble those of their analog with bulky *tert*-butyl wingtips,^47^ and are among the shortest distances found for the Cu-CNC complexes with electron-donating alkyl and aryl wingtips.^43^ The Cu–C_NHC_ bond distances in these complexes are consistent with those reported for other Cu(i)-NHC and Cu(i)-CNC complexes. The C–Cu–C bond angles of 173.97(6)° in [Cu-L3]^+^ and 178.04(6)° in [Cu-L4]^+^ are among the largest values determined for the Cu-CNC complexes of this type. Another notable feature of the Cu-CNC systems is the dihedral angle between the chelate and pyridine rings, which was determined as 162.2(1)° in [Cu-L3]^+^ and 178.8(1)° in [Cu-L4]^+^. These values support a nearly coplanar arrangement of the pyridine and NHC rings in the solid-state structure of the complexes. This arrangement is consistent with other Cu-CNC complexes bearing aryl wingtips and is distinct from their alkyl analogs except [Cu(I^*t*^Bu^C^N^C^)]PF_6_ whose average dihedral angle was 171.5(3)°.

### Direct carboxylation of terminal alkynes

#### Reaction condition screening

Initial transformations of phenylacetylene to propiolic ester were performed using [Cu-L1]PF_6_. All reactions were conducted in a sealed Schlenk tube connected to a Schlenk line to maintain 1 atm of CO_2_ pressure, as measured by a pressure gauge. The model reaction was initially performed at 80 °C in different solvents, including those that have been optimal solvents with other copper complexes, such as dimethylformamide (DMF),^13,25^ dimethyl sulfoxide (DMSO),^29^ and dimethylacetamide (DMA).^28^ Initial screenings were conducted using a 10 mol% loading of [Cu-L1]^+^, with Cs_2_CO_3_ as the base and iodoethane as the coupling partner.

As shown in Table 1, the highest yield of ethyl 3-phenylpropiolate was obtained in nitrile solvents, including acetonitrile and propionitrile (entries 4 and 5). Reducing the temperature to 60 °C resulted in a significantly lower product yield (entry 6). For the optimized base, two equivalents of Cs_2_CO_3_ resulted in significantly higher yield (entry 5) than the other inorganic bases tested (entries 7–10). The reaction did not proceed in the presence of KOH (entry 10) due to the base's insolubility.^48^ The reaction time also had a notable effect on the catalyst's performance. The highest product yield was achieved when the reaction was stirred for 12 hours at room temperature, followed by an additional 12 hours at 80 °C (entry 11). Reducing the reaction times at either temperature resulted in a lower yield if the total reaction time was less than 24 hours (entries 13–15). This observation was further supported by the nearly identical yield obtained to that in entry 11 when the reaction mixture was stirred for 1 hour at room temperature and 23 hours at 80 °C (entry 16). While a mixed-temperature state appears essential for optimal catalytic performance, conducting the reaction at room temperature with an extended stirring time of 48 hours also yields the ester product in high yield (entry 17). Furthermore, performing the reaction without iodoethane afforded the carboxylic acid at 48% yield (entry 11). The reduced yield of acid compared to the corresponding ester is linked to the instability of propiolic acid derivatives at elevated temperatures. To further improve catalyst performance in the model carboxylation reaction, the concentrations of all reactants were doubled, resulting in a notable increase in the ester product yield at catalyst loadings as low as 2.5 mol% (Fig. S1, SI). In addition, a control experiment was conducted under argon to verify that the carboxylate group originates from CO_2_ rather than Cs_2_CO_3_, yielding no detectable product (entry 18).

**Table 1:** Screening of reaction conditions with [Cu-L1]PF_6_^a^

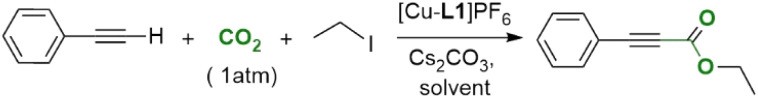				
No.	Solvent	Base (eq.)	Time & temp. (°C)	Yield^b^ (%)
1	DMSO	Cs_2_CO_3_ (2)	24 h (RT), 24 h (80)	56
2	DMF	Cs_2_CO_3_ (2)	24 h (RT), 24 h (80)	55
3	DMA	Cs_2_CO_3_ (2)	24 h (RT), 24 h (80)	Trace
4	PrCN	Cs_2_CO_3_ (2)	24 h (RT), 24 h (80)	75
5	CH_3_CN	Cs_2_CO_3_ (2)	24 h (RT), 24 h (80)	77
6	CH_3_CN	Cs_2_CO_3_ (2)	24 h (RT), 24 h (60)	54
7	CH_3_CN	Cs_2_CO_3_ (1.1)	24 h (RT), 24 (80)	30
8	CH_3_CN	K_2_CO_3_ (2)	24 h (RT), 24 h (80)	11
9	CH_3_CN	K_3_PO_4_ (2)	24 h (RT), 24 h (80)	63
10	CH_3_CN	KOH (2)	24 h (RT), 24 h (80)	0
11	CH_3_CN	Cs_2_CO_3_ (2)	12 h (RT), 12 h (80)	88 (48)^c^
12	CH_3_CN	Cs_2_CO_3_ (2)	12 h (RT), 12 h (80)	82^d^
13	CH_3_CN	Cs_2_CO_3_ (2)	9 h (RT), 9 (80)	80
14	CH_3_CN	Cs_2_CO_3_ (2)	6 h (RT), 6 h (80)	75
15	CH_3_CN	Cs_2_CO_3_ (2)	3 h (RT), 3 h (80)	69
16	CH_3_CN	Cs_2_CO_3_ (2)	1 h (RT), 23 h (80)	86
17	CH_3_CN	Cs_2_CO_3_ (2)	48 h (RT)	67
18	CH_3_CN	Cs_2_CO_3_ (2)	12 h (RT), 12 h (80)	0^e^

a Reaction conditions: phenylacetylene (0.68 mmol), [Cu-L1]PF_6_ (10 mol%), iodoethane (2.0 mmol), base, solvent (5 mL), CO_2_ (1 atm).

b Isolated yields.

c The yield in the absence of iodoethane.

d The isolated yield obtained using CO_2_ (0.5 atm).

e The reaction was performed under an Ar atmosphere.

#### Copper catalyst screening

The performance of several Cu-CNC complexes as catalysts was examined in the direct carboxylation of phenylacetylene under the established optimal conditions (Table 2). For comparison, the reaction was also conducted without any copper catalyst (entry 1) and with I^i^Pr^C^N^C^.2HBr salt as the catalyst and source of free NHC (entry 2). While there have been reports suggesting that commercial Cs_2_CO_3_ can carboxylate terminal alkynes under high CO_2_ pressure and elevated temperatures,^15,20^ our experiments using optimized conditions without a copper source yielded no detectable products (entry 1). According to the data in Table 2, the highest yields of propiolate were obtained with [Cu-L3]^+^ and [Cu-L5]^+^ complexes containing the mesityl (Mes) and para-CF_3_ aryl wingtips, respectively (entries 6 and 8). In contrast, [Cu-L4]^+^, containing the bulky diisopropylphenyl (Dipp) wingtips, provided the lowest carboxylation yield among the Cu-CNC complexes (entry 7). A practical aspect of the current system is that the Cu-CNC catalysts can be generated *in situ* and still afford a comparable yield (entry 4) to that obtained using an isolated complex (entry 3). The control experiments also indicated that the [Cu(CH_3_CN)_4_]PF_6_ salt could provide the propiolate ester at a reasonable yield under optimal conditions (entry 9).

**Table 2:** Screening of Cu(i) catalysts for direct carboxylation of phenylacetylene^a^

Entry	Catalyst	Yield^b^ (%)
1	No copper	0^c^
2	I(^i^Pr)^C^N^C^.2HBr	11
3	[Cu(I^i^Pr^C^N^C^)]PF_6_, [Cu-L1]PF_6_	88
4	[Cu(I^i^Pr^C^N^C^)]PF_6_ (*in situ*)^d^	83
5	[Cu(IMe^C^N^C^)]PF_6_, [Cu-L2]PF_6_	85
6	[Cu(IMes^C^N^C^)]PF_6_, [Cu-L3]PF_6_	95
7	[Cu(IDipp^C^N^C^)]PF_6_, [Cu-L4]PF_6_	64
8	[Cu(^4-CF3^Ar^C^N^C^)]PF_6_, [Cu-L5]PF_6_	99
9	[Cu(CH_3_CN)_4_]PF_6_	64

a Reaction conditions: phenylacetylene (0.68 mmol), cat. (10 mol%), iodoethane (2.00 mmol), Cs_2_CO_3_ (1.37 mmol), CH_3_CN (5 mL), CO_2_ (1 atm).

b Isolated yields.

c No copper catalyst.

d Conditions: Cu(CH_3_CN)_4_PF_6_ (0.068 mmol), I(^i^Pr)^C^N^C^.2HBr (0.082 mmol), Cs_2_CO_3_ (1.37 mmol), phenylacetylene (0.68 mmol), iodoethane (2.00 mmol), CH_3_CN (5 mL), CO_2_ (1 atm).

#### Substrate scope screening

**Fig. 3:**
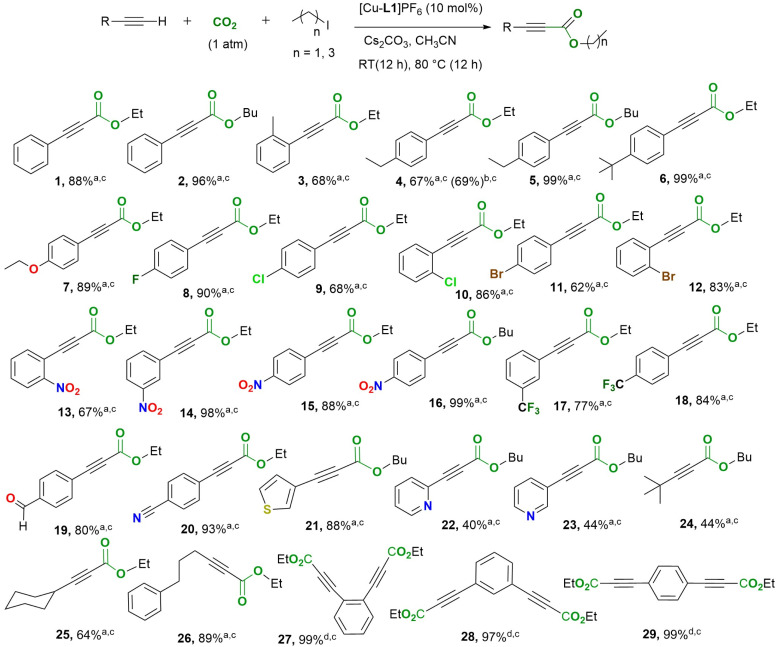
Direct carboxylation-esterification of terminal alkynes with CO_2_ catalyzed by [Cu-L1]PF_6_. Reaction conditions include (a) [Cu] (10 mol%), terminal alkyne (1.0 equiv.), Cs_2_CO_3_ (2.0 equiv.), EtI or ^*n*^BuI (2.9 equiv.), CO_2_ (1 atm), CH_3_CN (5 mL), 12 h at RT, 12 h at 80 °C. (b) 6 h at RT, 6 h at 80 °C. (c) Isolated yield. (d) [Cu] (10 mol%), terminal alkyne (1.0 equiv.), Cs_2_CO_3_ (4.0 equiv.), EtI (6.0 equiv.), CO_2_ (1 atm), CH_3_CN (5 mL), 12 h at RT, 12 h at 80 °C.

To investigate the scope of application of the Cu-CNC catalysts, the direct carboxylation of a diverse range of terminal alkynes was examined under optimized conditions using [Cu-L1]^+^. This choice of complex over [Cu-L5]^+^, which provided the highest yield, was made to keep consistency between catalytic reactions and mechanistic studies. As shown in Fig. 3, the ethyl or butyl 2-alkynoates of various alkyl- and aryl-substituted terminal alkynes were obtained in good to excellent yields. Phenylacetylenes containing electron-donating groups, *e.g.* Me, Et, ^*t*^Bu, and OEt, were converted to the corresponding alkynoates in good (67%) to excellent (99%) yields. The relatively low yield of ethyl 3-(4-ethylphenyl)propiolate (4) could be related to the instability of this product at the reaction temperature since a significant improvement was observed with ^*n*^BuI as the esterification reagent (5). In addition to activated alkynes, electron-withdrawing groups on the *ortho*-, *meta*- or *para*-positions of phenylacetylene were well tolerated and provided good (62%) to excellent (98%) yields. This is important because terminal aromatic alkynes with strong electron-withdrawing substituents, such as 4-nitro or 4-cyano, are the least reactive substrates in carboxylation due to their reduced nucleophilicity.^49^ This feature has led to their absence from the carboxylation studies performed on terminal alkynes in the presence of several copper complexes.^25,27,50^ In some cases, zero product yields have been reported for these derivatives.^28^ For the 4-halophenylacetylenes, the substrate activity is related to the electronegativity of halogen in the order F >> Cl > Br. Notably, 3-(4-fluorophenyl)propiolate (8) presented the highest yield among the para-substituted derivatives. Moreover, the 2-halophenyl acetylenes (10 and 12) demonstrated higher reactivity than their para-substituted analogs (9 and 11). Interestingly, these trends have not been observed in other copper systems supported by poly-NHC^13^ or non-NHC ligands,^29,50^ suggesting a different reaction mechanism. The position of the electron-withdrawing group also influences the reactivity of the nitrophenylacetylenes (13–15), with the *meta*-nitro derivative presenting the highest yield of the propiolate product (14). In addition to the aryl terminal alkynes, aliphatic alkynes were also converted efficiently to their corresponding esters at yields ranging from 44% to 89%. The low and moderate yields of 3,3-dimethyl-1-butyne and 1-cyclohexyne, respectively, are attributed to their volatility and/or instability at the reaction temperature (24, 25). In contrast, the less volatile substrates, such as 5-phenyl-1-pentyne, provided decent yields of the corresponding propiolate product (26). Furthermore, substrates bearing a heteroaromatic ring system were also employed, including 3-ethynyl thiophene, 2-ethynyl pyridine, and 3-ethynyl pyridine. Both ethynyl pyridine derivatives provided relatively low yields (22 and 23), whereas 3-ethynyl thiophene gave a decent yield (21). The poor performance of copper complexes toward basic nitrogen heterocycles was previously observed in CuI/PEt_3_,^28^ Cu(IPr)Cl,^27^ and Cu-ATF systems.^29^ This could be attributed to the coordinating ability of nitrogen heterocycles, which slows down catalysis. We also examined the direct carboxylation of double-terminal alkynes under optimized conditions. This assessment revealed the complete conversion of all three diethynyl benzene derivatives to their corresponding carboxylate forms (27–29). Attempts to obtain the mono-carboxylate derivative using reduced base concentrations resulted in a mixture of products.

#### Catalyst performance metrics

To investigate the stability of the copper catalyst and potential deactivation during the reaction cycle, a catalyst recovery method was developed. In this process, the crude mixture from the catalytic reaction underwent multiple hexane triturations to extract the propiolate ester product (83%). The remaining solid was purified through a series of trituration and recrystallization to yield the copper complex in its entirety. The ^1^H NMR spectrum of the recovered complex revealed two sets of resonance signals corresponding to [Cu-L1]PF_6_ (85%) and another Cu-CNC species (15%) with the same pattern but slightly different chemical shifts (Fig. S2, SI). Despite the difference, the minor copper derivative also contains methylene linkers, which can generate a dearomatized species upon deprotonation. Reusing the recovered catalyst in its entirety yielded 83% of the corresponding propiolate ester for at least five cycles, confirming the stability of the Cu-CNC complexes under the reaction conditions. Furthermore, tracking the reaction's progress over time indicates a consistent increase in yield, supporting the catalyst's stability in this process (Fig. S3, SI). It is important to note that the catalyst selectively promotes carboxylation without producing any other side products. Furthermore, when evaluating the catalyst's performance under scaled-up conditions (see Fig. S1, SI), a turnover number (TON) of 30.0 and a turnover frequency (TOF) of 1.25 were observed for the [Cu-L1]PF_6_ catalyst.

### Experimental mechanistic study

Despite the promising application of a handful of Cu-NHC complexes as catalysts in the direct carboxylation of terminal alkynes with CO_2_, the reaction mechanisms are still unclear.^13,27,51^ This is partly due to NHC's ambiguous role in these reactions and the limited scope of the available molecular copper catalysts. In the [Cu(NHC)Cl] complexes, wherein NHC is a monodentate ligand, the strong σ-donation of NHC and the lability of the halide trigger the formation of [Cu(NHC)(OH)] as a versatile synthon capable of activating various X–H bonds (X = C, N, O).^52^ A subsequent step involving CO_2_ insertion into the Cu-acetylide bond results in the propiolate anion, which can undergo an S_N_2 reaction with an alkyl/allyl halide, producing a propiolate ester.^53^ On the other hand, the carboxylation mechanism with the Cu-poly(NHC) complex is notably different. While the alkynyl C–H bond activation in this complex is similar to that of neutral [Cu(NHC)Cl], CO_2_ activation occurs at the nucleophilic site of the free NHC in the structure.^31^ A unique feature of the Cu-CNC complexes in this study is the chelation of copper by the pincer ligand and the *trans*-orientation of the two NHC units. This arrangement disfavors the displacement of a trans NHC ligand, avoiding the formation of reactive [Cu(NHC)(X)] (X = Brønsted base) species. In contrast, it enables the formation of other reactive species, which are examined in the following stoichiometric and single-step reactions.

#### Deprotonation of [CuL1]BArF and [CuL3]BArF complexes with a base

**Fig. 4:**
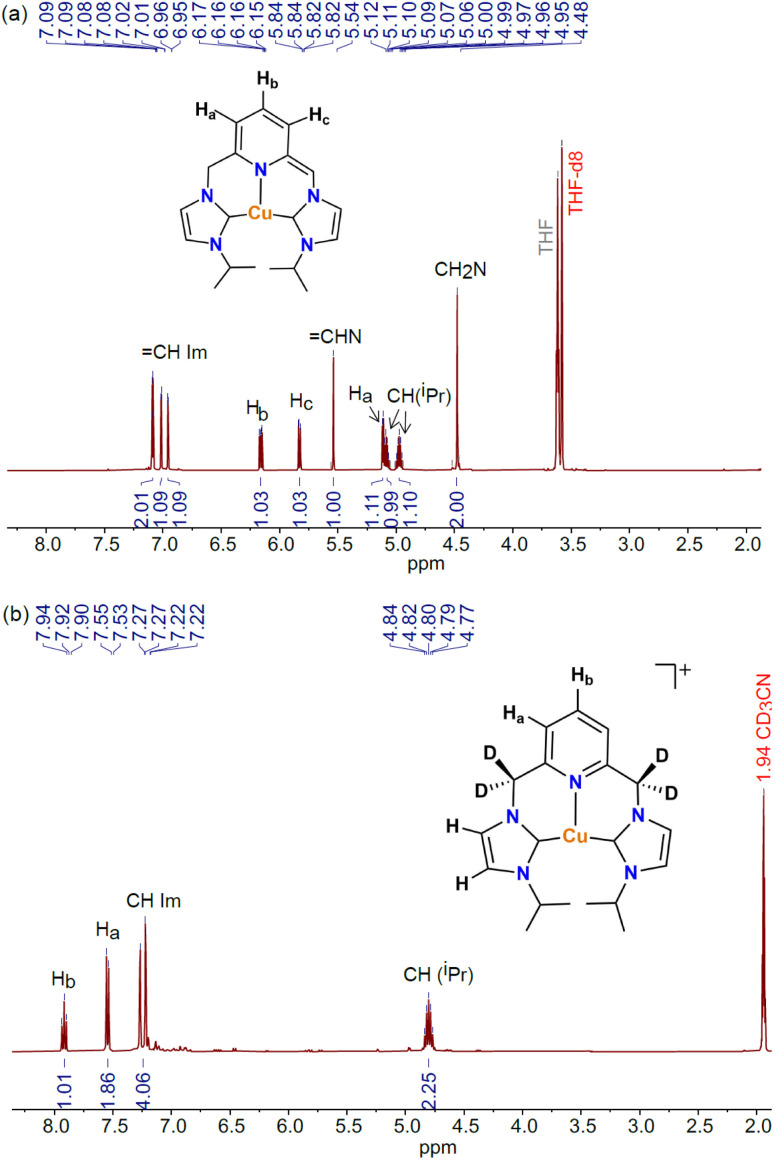
(a) ^1^H NMR spectrum (8.0–2.0 ppm) for [Cu(I^i^Pr^C^N^C^)*], [Cu-L1*], in THF-*d*_8_ at 298 K. (b) ^1^H NMR spectrum (8.0–2.0 ppm) of the reaction of [Cu-L1]PF_6_ (1 equiv.) and Cs_2_CO_3_ (5 equiv.) in CD_3_CN after 24 h stirring at room temperature.

The coordination of lutidine-based pincer ligands to late-transition metals results in acidic methylene linkers that are susceptible to deprotonation by a base, as reported by Milstein.^33^ The resulting dearomatized species are highly reactive and are often characterized by spectroscopic methods in solution. The reaction of [Cu-L1]BArF with KO^*t*^Bu in a 1 : 20 molar ratio was performed in THF-*d*_8_ at room temperature and was monitored by ^1^H NMR spectroscopy. The UV-vis studies of the reaction revealed the need for an excess of base to allow the complete formation of the dearomatized complex (*vide infra*). The ^1^H NMR spectrum of the dearomatized complex [Cu-L1*], obtained upon mixing the reagents (Fig. 4a) or after 24 h of stirring at room temperature, displayed a drastic change in the position and number of signals compared to [Cu-L1]BArF (Fig. S4, SI). The dearomatization of the pyridine ring is reflected in three distinct aromatic signals corresponding to the para and meta hydrogen atoms that experience a significant upfield shift to 6.16, 5.83, and 5.11 ppm. The methylene group resonances appear at 4.48 ppm as a singlet, a pattern known for copper complexes containing lutidine-based CNC ligands.^42,43^ Furthermore, the =CHN signal of the deprotonated arm was observed as a singlet at 5.54 ppm. Similar resonance signals were also detected for [Cu-L3*] prepared *in situ* through the reaction of [Cu-L3]BArF and KO^*t*^Bu (5 equiv.) in THF-*d*_8_ (Fig. S5, SI). These key resonances of [Cu-L1*] and [CuL3*] resemble a similar pattern to that reported earlier for the Rh-CNC analog in THF-*d*_8_.^40^

While generating the dearomatized species in THF-*d*_8_ using excess KO^*t*^Bu is imperative for its characterization, understanding its stability and reactivity in acetonitrile is crucial for catalysis. To explore this concept, the [Cu-L1]PF_6_ complex was treated with stoichiometric and excess amounts of Cs_2_CO_3_ in CD_3_CN to enable the formation of [Cu-L1*]. The ^1^H NMR spectra of reaction mixtures were obtained after 24 hours of stirring at room temperature. As shown in Fig. 4b, the spectrum closely resembles the resonances observed in the initial complex (Fig. S6, SI), but the methylene linkers have entirely vanished. This result is due to the full deuteration of the CH_2_ groups in [Cu-L1]PF_6_, as confirmed by a broad peak in the ^2^H NMR spectrum of the reaction in CD_3_CN (Fig. S7, SI). The same phenomenon occurred with a stoichiometric amount of Cs_2_CO_3_, resulting in the starting complex with fully deuterated methylene linkers ([Cu-L1^D^]^+^, Fig. S8, SI). These data highlight the reactive nature of the dearomatized species in acetonitrile.

**Fig. 5:**
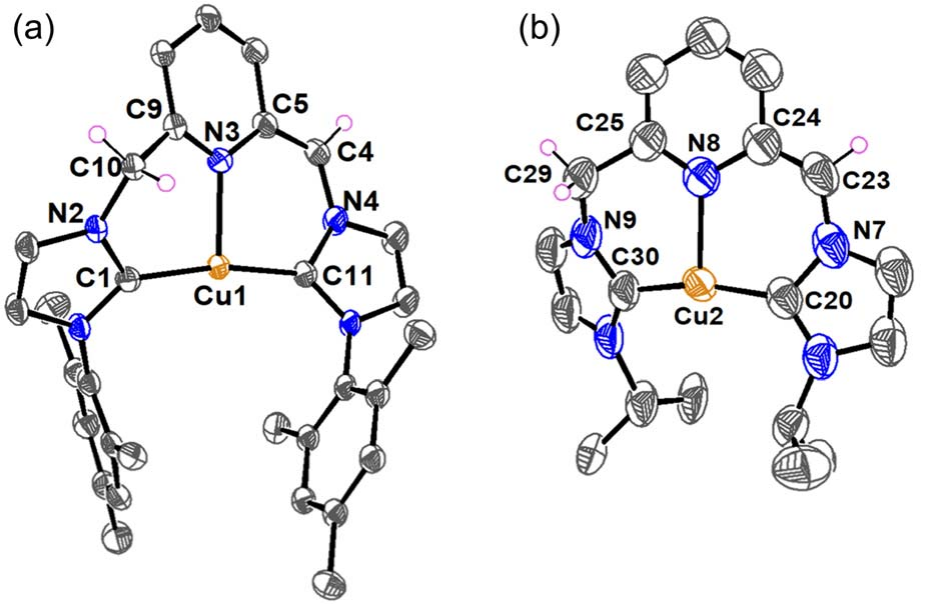
ORTEP diagrams of (a) [Cu-L3*] and (b) [Cu-L1*]. Hydrogen atoms have been omitted for clarity, except those of the methine and methylene arms. Only one of the crystallographically unique molecules is shown for [Cu-L1*]. The disorder sites for this molecule were omitted for clarity. Ellipsoids are shown at the 60% probability level.

In addition to spectroscopic characterization, we successfully grew single crystals of [Cu-L3*] and [CuL1*] in a THF solution at −30 °C for X-ray crystallography studies. The molecular structures of the complexes are shown in Fig. 5, along with the main bond angles and bond distances in Table S2. A set of relevant bond distances and angles in the aromatized and dearomatized complexes is also given in Table 3 for comparison. While the coordination environment and the Cu–C_NHC_ band distances of [Cu-L3*] and [Cu-L1*] resemble those of [Cu-L3]^+^ and [Cu-L1]^+^, respectively, the Cu–N_py_ distances are significantly shorter, due to an increase in the donor character of the pyridinic nitrogen. Furthermore, the increase in the C(5)–C(4)–N(4) bond angle from 115.0(1)° in [Cu-L3]^+^ to 128(1)° in [Cu-L3*] and the decrease of the C(5)–C(4) bond distance from 1.512(2) Å in [Cu-L3]^+^ to 1.36(1) Å in [Cu-L3*] are noticeable. The same trend was observed for the C(24)–C(23)–N(7) bond angle and the C(23)–C(24) bond length in [Cu-L1*] compared to their counterparts in [Cu-L1]^+^. Additionally, the alternated bond lengths of the pyridyl groups in [Cu-L3*] and [Cu-L1*] indicate the disruption of the aromaticity within the ring (Table S2, SI). Due to the limited number of crystallographically characterized dearomatized TM-CNC, the structural data for the current [Cu-L*] derivatives can be compared with the Rh-CNC counterpart, the only previously reported example before our work.^40^ Although the coordination geometry differs and a π-acceptor CO is present on Rh, the essential structural characteristics of the dearomatized Cu-CNC complexes—such as the shorter metal-N_py_ distance, the shorter C–C bond, and the larger C–C–N bond angle of the methine linker—are consistent with those observed in the rhodium counterpart.

**Table 3:** Significant bond distances and angles in the [Cu-L]PF_6_ and [Cu-L*]

Complex	Bonds	Distances (Å)/angles (°)	Ref.
[Cu-L1]PF_6_	Cu(1)–N(1)	2.294(1)	42
	C(5)–C(4)	1.510(2)	
	C(9)–C(8)–N(3)	114.5(1)	
[Cu-L3]PF_6_	Cu(1)–N(3)	2.221(1)	This work
	C(5)–C(4)	1.510(2)	
	C(5)–C(4)–N(2)	115.0(1)	
[Cu-L1*]	Cu(2)–N(8)	2.10(1)	This work
	C(23)–C(24)	1.35(3)	
	C(25)–C(29)	1.55(2)	
	C(24)–C(23)–N(7)	129(2)	
	C(25)–C(29)–N(9)	114(1)	
[Cu-L3*]	Cu(1)–N(3)	2.095(4)	This work
	C(5)–C(4)	1.36(1)	
	C(9)–C(10)	1.52(2)	
	C(5)–C(4)–N(4)	128(1)	
	C(9)–C(10)–N(2)	113(1)	

Next, the formation and stability profile of the dearomatized species was further investigated using UV-vis spectroscopy at low temperatures. The reaction of [Cu-L1]BArF with excess KO^*t*^Bu in THF produces a dark red chromophore with ligand-centered absorption bands around 280–350 nm and four absorption maximum wavelengths (*λ*_max_) at 423 nm (*ε* = 7.2 × 10^3^ M^−1^ cm^−1^), 475 nm (*ε* = 2.7 × 10^3^ M^−1^ cm^−1^), 505 nm (*ε* = 2.0 × 10^3^ M^−1^ cm^−1^), and 539 nm (*ε* = 9.0 × 10^2^ M^−1^ cm^−1^). The *ε* values of [Cu-L1*] were determined by spectral titration of [Cu-L1]BArF with base in THF at 223 K (Fig. S9, SI). We also attempted to clarify the nature of electronic transitions in the UV-vis spectrum using time-dependent density functional theory (TD-DFT). The calculated spectrum resembled prominent features similar to those found experimentally; however, the positions and numbers of absorption bands differed slightly (Fig. S10, SI). This difference is attributed to the solvent effects and interactions with other ions present in solution, including excess base. According to the calculations, the absorbance at 340 nm is primarily ligand-to-ligand charge-transfer (LLCT), while the peak at 423 nm is a hybrid of metal-to-ligand charge-transfer (MLCT) and LLCT transitions. Furthermore, the absorption band at 505 nm originates primarily from ligand-to-metal charge-transfer (LMCT) transitions.

**Fig. 6:**
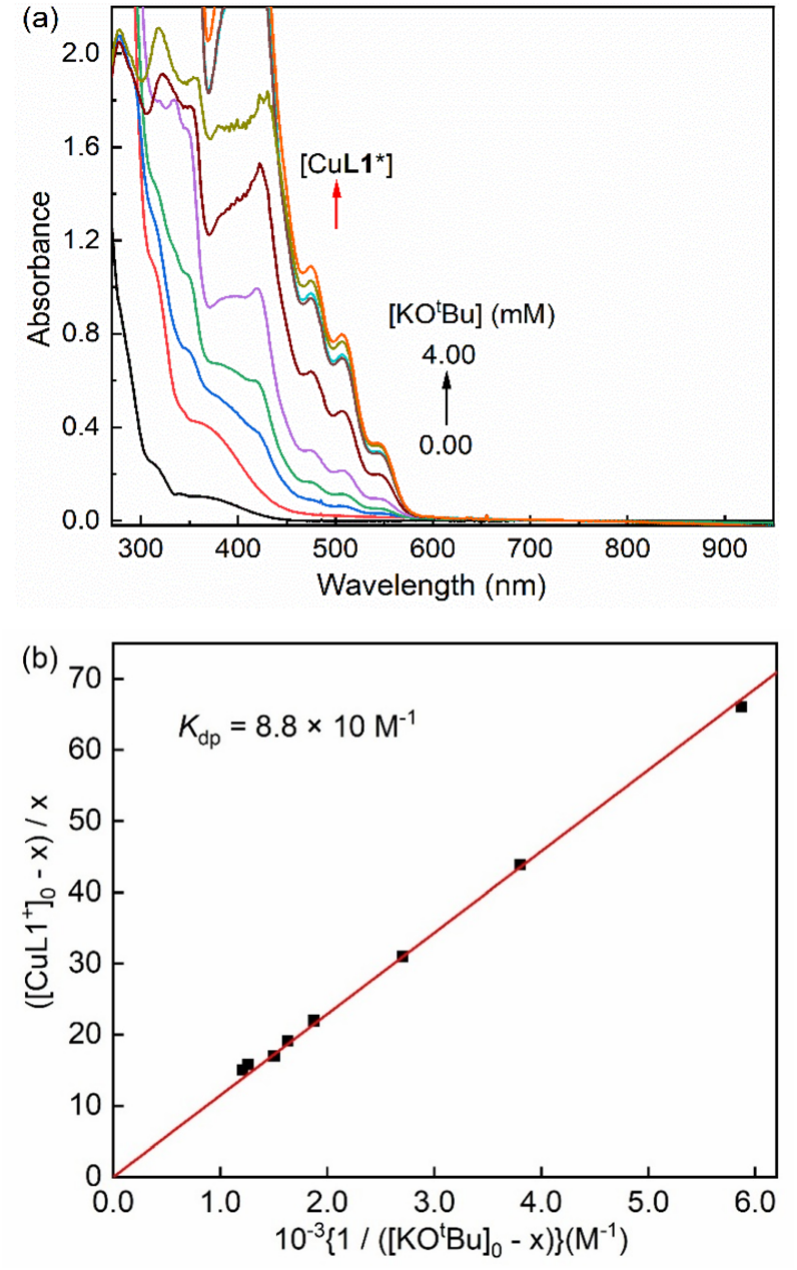
(a) UV-vis spectral changes of [Cu-L1]BArF (0.50 mM) in the presence of KO^*t*^Bu (0.0–4.0 mM) in THF at 193 K. (b) Plot of ([CuL1^+^]_0_ – x)/x *versus* 1/([KO^*t*^Bu]_0_ – x) to determine the deprotonation constant of [CuL1]^+^ upon addition of KO^*t*^Bu (0.0–4.0 mM) into the solution of [CuL1]^+^ (0.40 mM) in THF at 193 K.

Since the formation of the dearomatized complex requires excess base amounts, an equilibrium between the [Cu-L1]^+^ and [Cu-L1*] species is postulated in solution (eqn (1)). A spectral titration of [Cu-L1]^+^ with KO^*t*^Bu was performed in THF at 193 K to determine the deprotonation constant (*K*_dp_) using eqn (2). This equation is easily converted to eqn (3), where [CuL1^+^]_0_ and [KO^*t*^Bu]_0_ are initial concentrations of [Cu-L1]^+^ and base, respectively, and x is the equilibrium concentration of [Cu-L1*] calculated using Beer's law and the absorbance change at 505 nm due to [Cu-L1*] (Fig. 6). The *K*_dp_ value is then determined from the inverse of the slope of a linear plot of ([CuL1^+^]_0_ – x)/x *versus* 1/([KO^*t*^Bu]_0_ – x) to be 8.8 × 10 M^−1^.

1 [Cu**L1**^+^]X + KO^*t*^Bu ⇌ [Cu**L**1∗] + KX(s) + HO^*t*^Bu(l)

2 *K*_dp_ = [Cu**L**1^∗^]/[Cu**L**1^+^][KO^*t*^Bu]

3 ([Cu**L**1^+^]_0_ − x)/x = *K*_dp_^^−1^^(1/([KO^*t*^Bu]_0_ − x))

In the next step, the temperature dependence of *K*_dp_ was examined (Fig. S11, SI), and the van't Hoff plot afforded the enthalpy of formation (Δ*H* = −2.3 ± 0.2 kcal mol^−1^) and the entropy of formation (Δ*S =* −3.2 ± 0.7 cal K^−1^ mol^−1^) for the dearomatized complex [CuL1*] in THF (Fig. S12, SI).

**Fig. 7:**
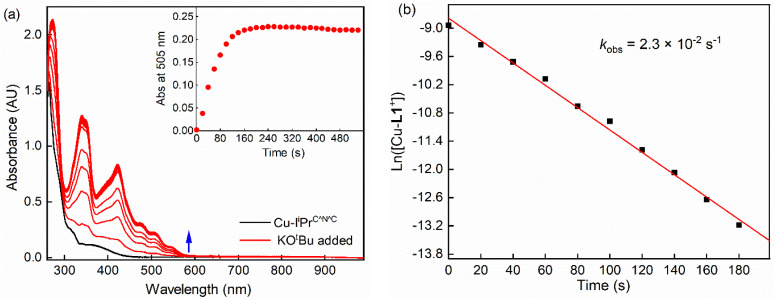
(a) UV-vis spectral changes observed in the deprotonation of [Cu-L1]BArF (0.10 mM) with KO^*t*^Bu (0.50 mM) in THF at 223 K. The inset shows the time profile of the absorbance at 505 nm due to [Cu-L1*]. (b) The first-order plot of [Cu-L1*] formation under these conditions.

In addition to the formation constant of [Cu-L1*], the dynamics of its reaction with the base were examined by UV-vis spectroscopy at 223 K. Fig. 7 shows the spectral changes corresponding to the formation of [Cu-L1*] (0.10 mM) in the presence of 0.5 mM KO^*t*^Bu. At this temperature, [CuL1]^+^ is fully converted to the dearomatized complex, as shown by the plateaued time profile within 160 seconds. The species remains reasonably stable at this temperature in the absence of air and moisture. The formation rate of [Cu-L1*] follows pseudo-first-order kinetics in THF at 223 K with a pseudo-first-order rate constant (*k*_obs_) of 2.3 × 10^−2^ s^−1^ and a second-order rate constant (*k*_2_) of 4.6 × 10 M^−1^ s^−1^, considering the concentration of the base. The formation of this species was also performed under a pseudo-first-order condition at 193 K, where a pseudo-first-order rate constant of 8.4 × 10^−3^ s^−1^ and a second-order rate constant of 8.4 M^−1^ s^−1^ were determined (Fig. S13, SI). The Eyring analysis of the rate constants at 223 K and 193 K (eqn (4)) provided an estimate of the activation enthalpy (Δ*H*^‡^ = 4.43 ± 0.1 kcal mol^−1^) and activation entropy (Δ*S*^‡^ = −30.5 ± 2 cal K^−1^ mol^−1^). The relatively small value of Δ*H*^‡^, and the negative value of Δ*S*^‡^ is usually consistent with an associative mechanism or an entropy-governed process.^54,55^ These values are comparable to those obtained for the photochemical carbonylation of benzene by a dearomatized Rh-PNP species, which follows an associative mechanism.^35^ Currently, no documented activation parameters have been reported for similar CNC-based dearomatized species in the literature.4
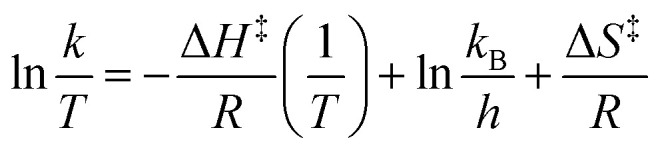


#### Reactivity of [Cu-L1]PF_6_ with phenylacetylene in the presence of the base

**Fig. 8:**
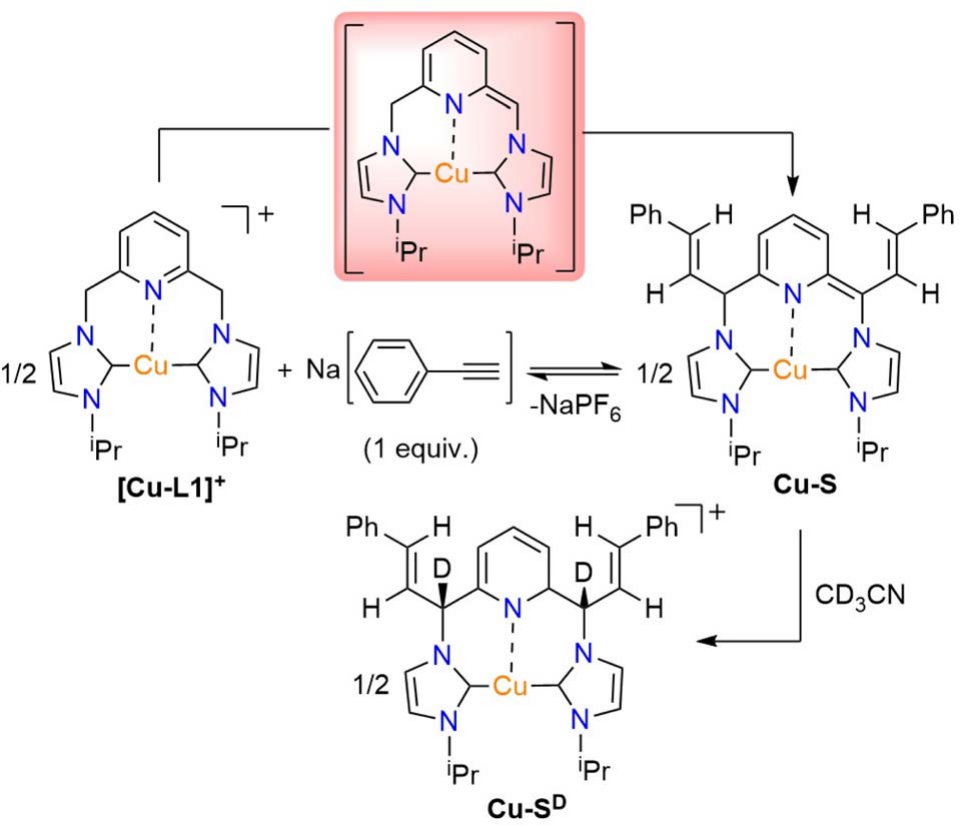
The Cu-styrenyl complex (Cu-S) formed from the reaction of [Cu-L1]PF_6_ and one equivalent of NaPA.

To develop an understanding of the species involved in the carboxylation mechanism, the reaction of [Cu-L1]PF_6_ and phenylacetylene was examined in the presence of a base. Initially, it is assumed that the reaction between phenylacetylene and the base takes precedence over the formation of the dearomatized species. To investigate this idea, the copper complex was treated with one equivalent of sodium phenylacetylide (NaPA) in CD_3_CN. The reaction was monitored by ^1^H NMR spectroscopy both at the mixing time and after 24 hours of stirring at room temperature, revealing the formation of a new species (Fig. 8). This species is characterized by two olefinic hydrogen signals at 6.93 and 6.47 ppm, which correlate with signals at 121.46 and 120.24 ppm in the ^13^C{^1^H} NMR spectrum, respectively (Fig. S14 and S16, SI). Additionally, a set of aromatic signals ranging from 7.07 to 7.28 ppm is observed, which corresponds approximately to a hydrogen ratio of 4 : 1. This pattern somewhat resembles that observed for a Rh-styrenyl complex, which is formed as an addition product of phenylacetylene on the dearomatized Rh-CNC complex.^41^ In contrast to the rhodium analog, the Cu-styrenyl species (Cu-S) is highly symmetrical, as evident from a single set of methine hydrogens (CH_iPr_) and the CHN linkers, as well as an integrated ratio of 1 : 1 : 1 for the CH(^i^Pr) : CHN(linker) : olefinic hydrogens. Moreover, the presence of a singlet at *δ*(^13^C) 187.67 ppm for the two carbenic NCN atoms is consistent with the symmetrical substitution of styrene units in this complex (Fig. S16, SI). While the initial Cu-S species is expected to be dearomatized, it readily undergoes saturation in acetonitrile, as indicated by the presence of CHN linkers at 5.02 ppm (Fig. S14, SI). In addition to signals from the Cu-S species, a set of low-intensity resonances was observed in the ^1^H NMR spectrum at 6.27, 6.09, 5.95, and 5.57 ppm. These features, which are only visible in the spectrum within the mixing time of the reaction, resemble those in the spectrum of [Cu-L1*] in THF-*d*_8_ (Fig. 4a). This is significant as it demonstrates the deprotonation of the methylene linkers of [Cu-L1]^+^ by the acetylide anion. Moreover, the low signal intensity indicates the involvement of the dearomatized species in the formation of the Cu-S complex, as previously shown for the Rh-CNC systems. Extending the reaction time to 24 hours afforded the Cu-S^D^ complex with fully deuterated methine linkers as the primary product, as shown by the complete absence of these signals in the ^1^H NMR spectrum (Fig. S15a, SI). The results were further confirmed by the ^2^H NMR analysis of the reaction mixture in CD_3_CN (Fig. S15b, SI). The lack of detectable pyridine and NHC backbone signals in the ^1^H NMR spectrum is attributed to rapid hydrogen–deuterium exchange, which was not detectable by ^2^H NMR at room temperature. Remarkably, the formation of Cu-S species appears to be reversible owing to the presence of NaPA resonances in the ^1^H NMR spectrum either upon mixing or after 24 h of stirring. This was further supported in the CO_2_ addition process, *vide infra*.

**Fig. 9:**
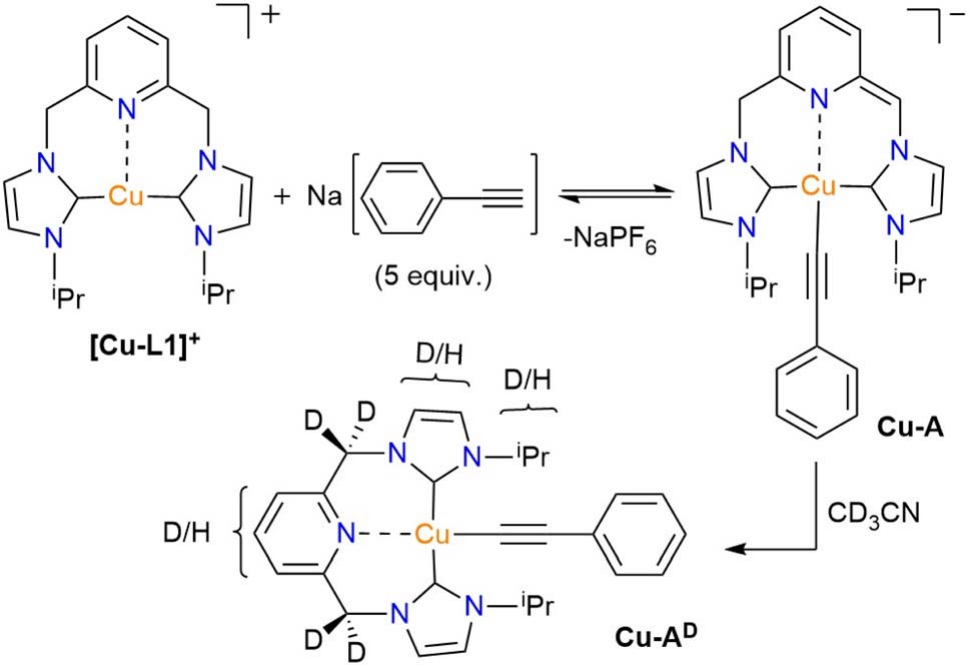
The Cu-acetylide complex (Cu-A) from reacting [Cu-L1]PF_6_ with excess NaPA.

Increasing the amount of NaPA to five equivalents in the reaction with [Cu-L1]^+^ resulted in a different product, as evident from the absence of two olefinic hydrogens in the ^1^H NMR spectrum after 24 hours of stirring (Fig. S17a, SI). A set of aromatic resonances (7.08–7.25 ppm) at a hydrogen ratio of 2 : 2 : 1 is also distinct from those of the styrenyl groups of the Cu-S species. The signals' pattern and relative integration resemble those of copper-acetylide complexes bearing NHC ligands prepared by others and by us (Fig. S18a, SI).^56,57^ The presence of acetylenic carbon signals at *δ* 94.19 and 83.68 ppm in the ^13^C{^1^H} NMR spectrum of Cu-A further supports this assignment (Fig. S18b, SI). Furthermore, the extensive deuterium exchange of the methylene linkers, the CH group of the isopropyl wingtips, and the pyridyl hydrogens support an initial formation of a dearomatized Cu-A species (Fig. 9). This theory was further supported by the appearance of resonance signals at 4.82, 5.31, and 7.59 ppm in the ^2^H NMR spectrum of the reaction, which correspond to the absent hydrogen groups in the ^1^H NMR spectrum (Fig. S17b, SI).

To evaluate the formation speed of Cu-A, the reaction between [Cu-L1]^+^ and NaPA (5 equiv.) was examined during the mixing time. The ^1^H NMR spectrum of the reaction confirmed the complete formation of the species and less deuteration. Additionally, the Cu-S peaks, including the methine linkers, isopropyl CH, and olefinic hydrogens, were observed (Fig. S19, SI). While these signals are much weaker than those of the Cu-A species, their presence suggests a possible transformation of the Cu-S to Cu-A in the presence of excess NaPA. Although the mechanism of this transformation remains to be clarified, it provides a valuable approach for utilizing the nucleophilic character of the dearomatized species beyond stoichiometric conditions. The Cu-acetylide formation is significant for catalysis, given its role as an active intermediate in the copper-catalyzed carboxylation of sp C–H bonds.^58^

To shed light on the Cu-S to Cu-A conversion, the reaction of [Cu-L1]^+^ and NaPA (1 equiv.) was examined at both room and high temperatures. The ^1^H NMR spectrum of the reaction within one hour of mixing at room temperature revealed the resonance signals of Cu-S (Fig. S20a, SI). Stirring this mixture at 80 °C for 12 h yielded the Cu-A species (Fig. S20b, SI), as indicated by NMR signals similar to those observed in the presence of excess NaPA. Slight broadening of the peaks is related to the dissociation of the acetylide anion from copper at high temperatures and to the reduced solubility of the acetylide salt. The experiment was repeated with [Cu-L3]PF_6_ complex and NaPA (1 equiv.), resulting in the formation of Cu-S species initially (Fig. S21, SI). Storing the solution under an inert atmosphere overnight resulted in yellow/orange crystals, which were collected and characterized by X-ray crystallography. As shown in the ORTEP diagram (Fig. S22, SI), the acetylide anion is directly bound to copper along with two NHC units from each of the two L3 ligands in the asymmetric unit. While the coordination mode of L3 is bridging for the Cu-A in the solid state, this could change in solution to the chelating mode, as was shown previously for the pincer CNC ligands bearing aryl wingtips.^43^

**Fig. 10:**
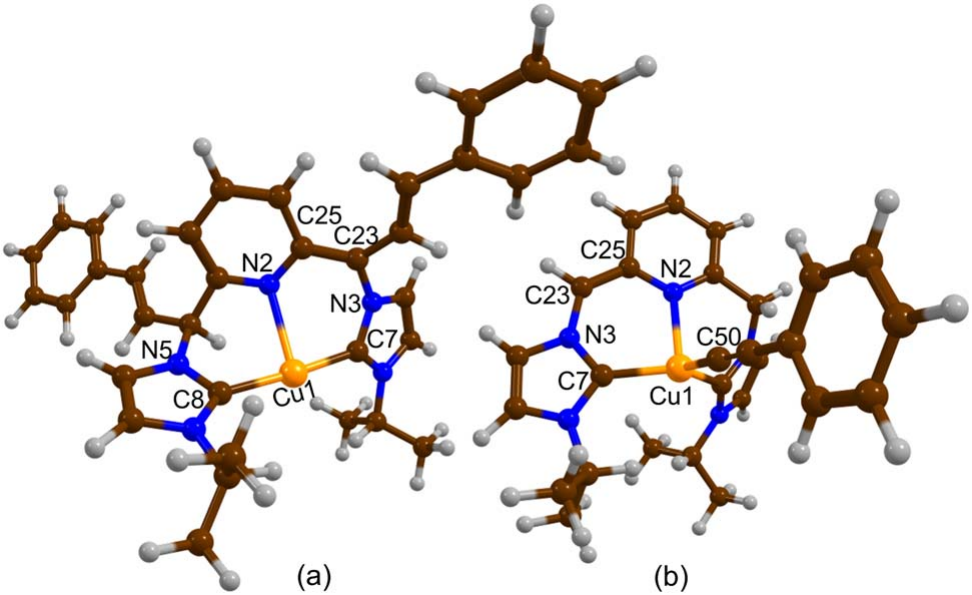
Optimized structure of (a) Cu-S and (b) Cu-A calculated by DFT UB3LYP-GD3BJ containing the basis set containing LANL2TZ^+^ (+ECP) on Cu and 6-311+G* on the rest of the atoms. Selected distances (Å) and angles (°): Cu-S; Cu(1)–N(2) = 2.415, Cu(1)–C(7) = 1.925, Cu(1)–C(8) = 1.931, C(23)–C(24) = 1.427, C(31)–C(32) = 1.527, C(24)–C(23)–N(3) = 118, C(31)–C(33)–N(5) = 108. Cu-A; Cu(1)–N(2) = 2.341, Cu(1)–C(7) = 2.005, Cu(1)–C(8) = 2.043, Cu(1)–C(50) = 1.965, C(23)–C(25) = 1.382, C(32)–C(33) = 1.518, C(25)–C(23)–N(3) = 128, C(32)–C(33)–N(5) = 112.

Despite the feasibility of forming the L1-supported Cu-S and Cu-A species in solution, isolating these species for further characterization by X-ray crystallography and elemental analysis has proven somewhat challenging. The solid collected after removing solvent from the Cu-S^D^ solution contains both Cu-A^D^ and Cu-S^D^ species at a mole ratio of approximately 35% to 65%, as indicated by the ^1^H NMR spectrum in CD_3_CN (Fig. S23, SI). Further work-up of the crude solid through THF trituration resulted in 82% of the [Cu-L1]^+^ along with 18% of Cu-S^H^ species (Fig. S23, SI). In the absence of X-ray data, the DFT-optimized structures of Cu-S and Cu-A were obtained, thereby supporting the proposed intermediates from NMR spectroscopy (Fig. 10).

In the next step, [Cu-L1]PF_6_, base, and alkyne were combined and reacted in a single step. This setup more closely resembles that of the catalytic reaction. The initial study involved a stoichiometric 1 : 1 : 1 ratio of the reagents, mixed as a solid, and stirred for 24 hours at room temperature in CD_3_CN. The ^1^H NMR spectrum of the reaction mixture revealed two sets of methine and methylene linker resonances corresponding to the Cu-A^H^ (27%) and Cu-S^H^ (73%) species, respectively (Fig. S24a, SI). The resonance signals of the starting complex in the spectrum indicate the existence of an equilibrium between [Cu-L1]^+^ and the products (eq (5)). Furthermore, the NMR spectrum showed an acidic signal for phenylacetylene at 3.39 ppm, with a 70 : 30 ratio of phenylacetylene to the acetylide salt, supporting the priority of deprotonating the methylene linkers over deprotonating the alkyne.

5 [Cu-**L1**]^+^ + H-PA + Base ⇌ [**Cu-S**]^+^ + [**Cu-A**]

**Fig. 11:**
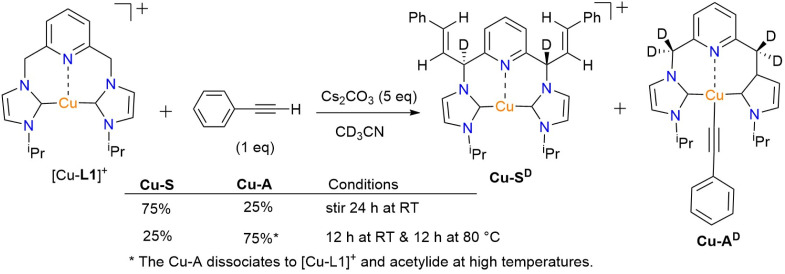
Cu-acetylide (Cu-A^D^) and Cu-styrene (Cu-S^D^) complexes formed by reacting [Cu-L1]PF_6_, phenylacetylene, and Cs_2_O_3_ in CD_3_CN.

Employing an excess amount of the base (5 equiv.) in the reaction of [Cu-L1]PF_6_ and phenylacetylene did not affect the mole ratio of the Cu-A and Cu-S drastically. However, it shifted the equilibrium toward the complete formation of the Cu-S and Cu-A species (Fig. 11). In addition, the deuteration of the methylene linkers in Cu-A and the methine linkers in Cu-S was accelerated. This was demonstrated by the significantly reduced intensity of these signals in the ^1^H NMR spectrum of the reaction (Fig. S24b, SI), as well as the appearance of the methine linkers of Cu-S in the ^2^H NMR spectrum (Fig. S25, SI). The absence of the methylene linkers of Cu-A from the ^2^H NMR spectrum is attributed to the low concentration of this species in solution. These findings support the preference of Cu-S formation over Cu-A under both stoichiometric and non-stoichiometric conditions at room temperature. Interestingly, this pattern changed when a similar time-temperature profile was used to that in the catalytic carboxylation. Stirring a reaction mixture of copper, base, and alkyne for 12 hours at room temperature and 12 hours at 80 °C resulted in a significant drop in the amount of Cu-S. This was demonstrated by the 75 : 25 ratio of the CH(^i^Pr) hydrogens for the [Cu-L1]^+^/Cu-S in the ^1^H NMR spectrum (Fig. S26, SI). The absence of Cu-A signals, which are readily distinguished from those of [Cu-L1]^+^ by the presence of the phenyl group in the aromatic region, is attributed to the species' instability at high temperatures, resulting in the formation of the starting complex and the acetylide anion (Fig. 9).

#### Reactivity of the Cu-CNC complexes with CO_2_ in the presence of the base and phenylacetylene

**Fig. 12:**
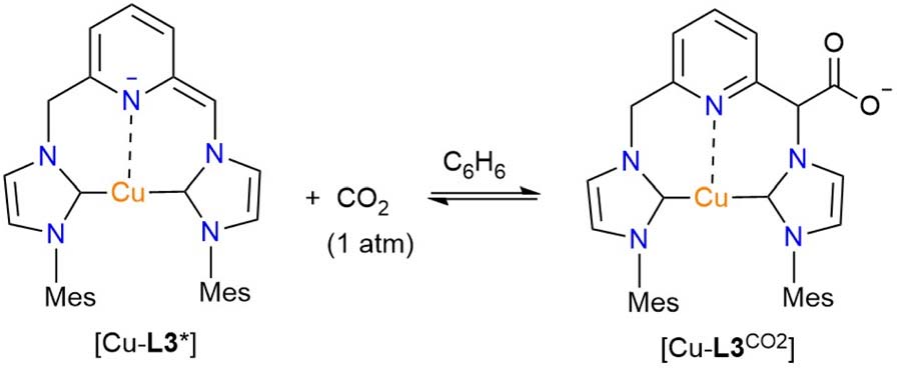
The Cu-carboxylate complex formed in the reaction of [Cu-L3*] and CO_2_ in benzene.

To examine any potential reaction with CO_2_, the in situ-formed [Cu-L3*] complex was treated with CO_2_ at atmospheric pressure in benzene. The rapid color change from dark red to orange, accompanied by precipitation upon CO_2_ exposure, indicates the formation of a new species. The ^1^H NMR spectrum of the orange solid in CD_3_CN after filtration and solvent removal under vacuum displays the characteristic peaks of the starting complex and a second set of resonance signals of much lower intensity (Fig. S27, SI). In the aromatic region of the spectrum, after masking the dominant peaks of the initial complex, seven notable resonance signals are observed. These signals are attributed to a complex featuring a carboxylate methylene (CHCO_2_) linker. The complex's unsymmetrical nature is evident from the presence of seven aromatic peaks, rather than the five signals expected for a symmetrical dicarboxylate derivative. Additionally, the low-field singlet at 5.79 ppm is associated with the CHCO_2_ linker and resembles a feature observed in the ^1^H NMR spectrum of the rhodium analog.^41^ The lack of methylene signals for the other half of the [Cu-L3^CO_2_^] complex and the diminished intensity of the CHCO_2_ linker are likely due to deuteration (Fig. 4b). Furthermore, the significantly reduced intensities of the carboxylate complex resonances are attributed to the equilibrium nature of the reaction and its reversal upon solvent removal under vacuum (Fig. 12).

**Fig. 13:**
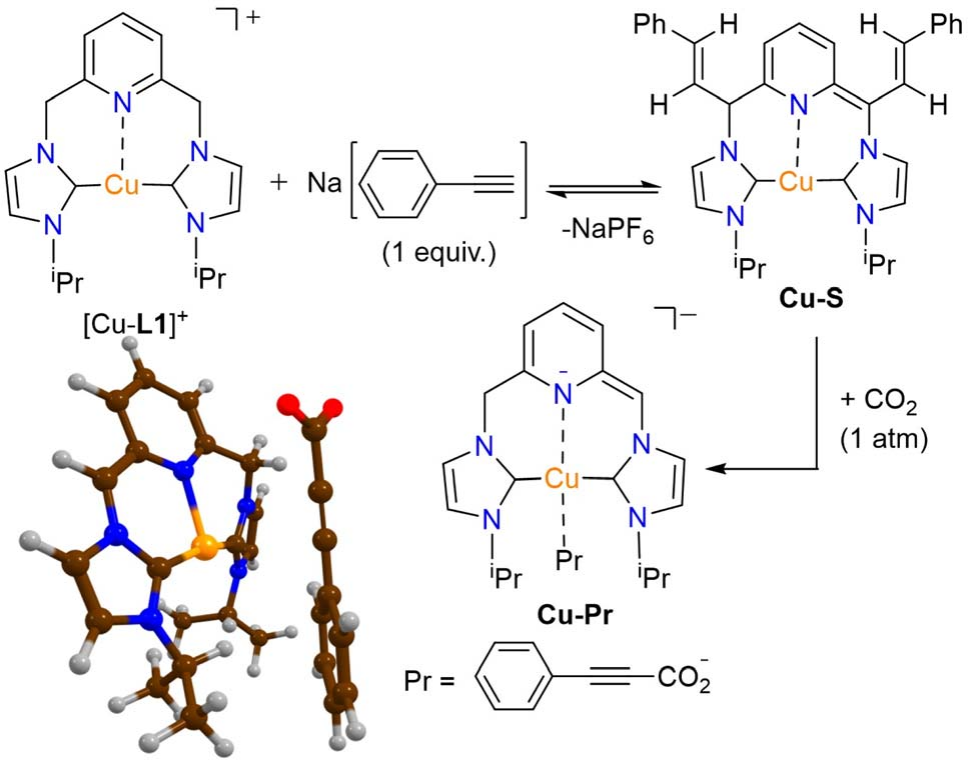
The Cu-propiolate complex formed from the reaction of [Cu-L1]PF_6_, NaPA, and CO_2_ in CD_3_CN, along with its DFT-optimized structure.

After examining the nucleophilic reactivity of the dearomatized Cu-CNC complex toward CO_2_ and phenylacetylene individually, it would be insightful to investigate the reaction under simultaneous exposure to both reagents. To explore this concept, the in situ-formed Cu-S complex, obtained from the reaction of [Cu-L1]PF_6_ with sodium acetylide (1 equiv.), was reacted with CO_2_ (1 atm). The ^1^H NMR spectrum of the reaction mixture revealed significantly different signals from those of the Cu-S^D^ and [Cu-L1]^+^ complexes (Fig. S28 and S29a, SI). The pyridinic signals (*p*-Py *δ*(^1^H) 7.75 ppm; *m*-Py *δ*(^1^H) 7.37 ppm) have experienced an upfield shift compared to these signals of [Cu-L1]PF_6_. Additionally, the aromatic resonances of the phenyl group have shifted upfield relative to the corresponding signals of the Cu-S^D^ and appear at a 2 : 2 : 1 integrated ratio of hydrogen. These signals also appear at a higher field (lower ppm) than those of the Cu-A^D^ complex (Fig. S29b, SI). The aromatic signals and the multiplet of the ^i^Pr methine hydrogens at 4.83 ppm suggest the formation of a new species, likely a copper propiolate complex (Cu-Pr), in solution (Fig. 13). This assignment was further supported by the IR spectrum of the reaction in CD_3_CN, which shows distinct absorption bands of C≡C stretching at *ν̄* = 2662 cm^−1^, asymmetric stretch of CO at *ν̄* = 1602 cm^−1^, and symmetric stretching of CO at *ν̄* = 1331 cm^−1^ (Fig. S30, SI). The more intense band of asymmetric stretching, compared to that of symmetric stretching, along with the significant frequency splitting of 271 cm^−1^, suggests that the propiolate anion is likely coordinated to the metal instead of existing as a free propiolate ion.^59^

It should be noted that the initially formed Cu-Pr complex is dearomatized, as is the case with Cu-S and Cu-A. This complex then undergoes deuterium exchange in CD_3_CN, as indicated by the absence of its methylene hydrogens in the ^1^H NMR spectrum (Fig. S28a, SI). Moreover, its formation is an equilibrium process that can be disrupted during a simple work-up to isolate the product. The ^1^H NMR spectrum of the solid collected after removing acetonitrile under vacuum displays the dominant signals of the starting [Cu-L1]PF_6_ and small amounts of protonated ligand (Fig. S31, SI). The same results were obtained when attempting to precipitate the Cu-Pr complex by adding a co-solvent to the reaction medium. The DFT-optimized structure of Cu-Pr is shown in Fig. 13 and is consistent with NMR spectroscopy findings in supporting the formation of the propiolate anion. However, the calculations showed the dissociated anion from copper in the gas phase as a lower-energy minimum in the optimization graph.

The formation of the Cu-Pr species from the reaction of Cu-S and CO_2_ suggests the internal conversion of Cu-S to Cu-A in the presence of CO_2_ (Fig. 13). While the mechanism of this transformation is yet to be understood, Cu-A complex is considered an essential intermediate in the carboxylation process due to its nucleophilic reactivity toward CO_2_. This was further verified by the reaction of the Cu-A complex with CO_2_. When the *in situ* formed Cu-A, produced by treating [Cu-L1]PF_6_ with excess phenylacetylide, reacted with CO_2_, the NMR spectrum of the reaction (Fig. S32, SI) was nearly identical to that observed in the Cu-S reaction with CO_2_ (Fig. S28a, SI). Furthermore, replacing phenylacetylene and Cs_2_CO_3_ with sodium phenylacetylide in the catalytic reaction yielded the propiolate ester product in 80% yield, thereby confirming the significance of the proposed intermediates in the carboxylation mechanism.

### Proposed mechanism of direct carboxylation catalyzed by Cu-CNC complexes

**Scheme 3:**
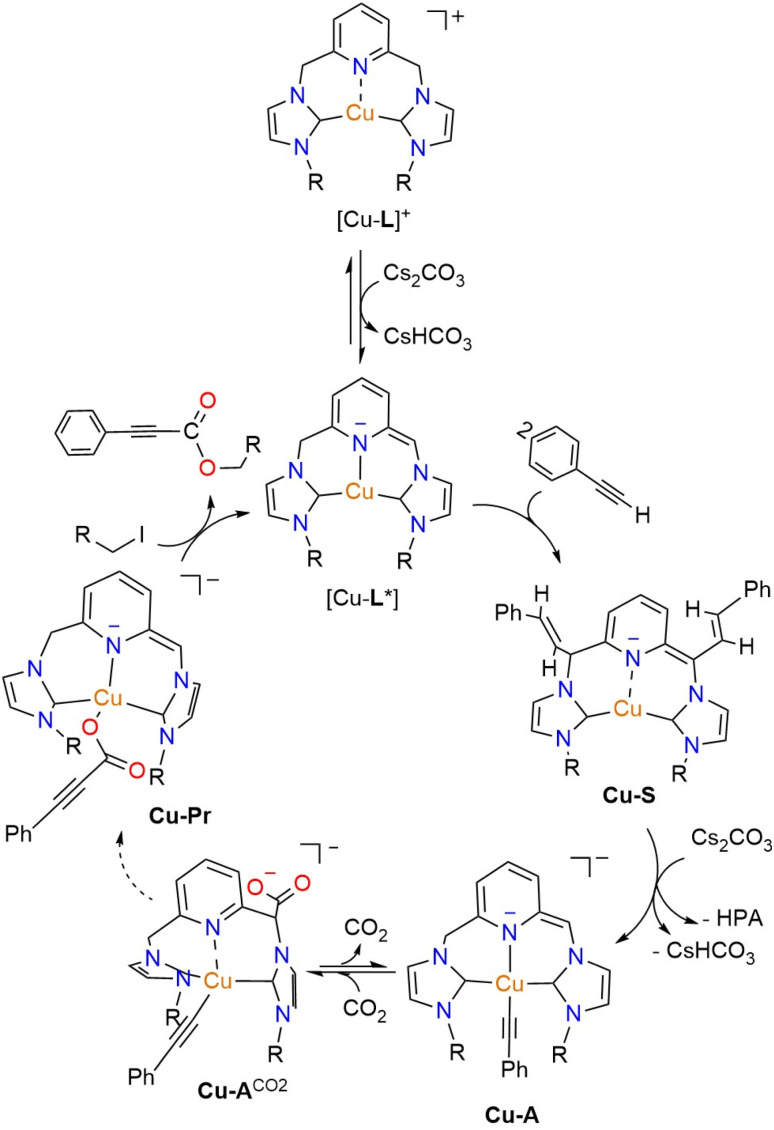
A possible mechanism for the direct carboxylation of terminal alkynes by Cu-CNC through MLC. The specific states involved in the transition from Cu-A to Cu-Pr are unclear.

Based on the experimental data described above, a possible mechanism for the Cu-CNC-catalyzed carboxylation of terminal alkynes with CO_2_ is proposed (Scheme 3). The initial deprotonation of the methylene linker produces a highly reactive, dearomatized intermediate (Cu-L*), which subsequently attacks phenylacetylene to form the styrene-functionalized Cu-S species. In the presence of CO_2_ and at high temperatures, this species undergoes an internal conversion to the CNC-supported copper-acetylide (Cu-A) intermediate. Insertion of CO_2_ into the Cu–C bond of Cu-A furnishes the copper-propiolate (Cu-Pr) in a reversible reaction. Further reaction of this species with alkyl halide regenerates the dearomatized complex and releases the propiolate ester product. It is worth noting that a subsequent reaction with solvent is a viable step for all the intermediates in the reaction cycle, including Cu-S, Cu-A, and Cu-Pr, as demonstrated by the ^1^H NMR experiments in CD_3_CN (*vide supra*). While this reaction results in the formation of saturated intermediates, the presence of excess Cs_2_CO_3_ ensures the steady regeneration of the dearomatized complexes and maintains their active form during the reaction.

After identifying the key intermediates in the mechanism, the mixed temperature profile of the carboxylation/esterification can be rationalized. While the formation of the dearomatized complex, as the active form of the catalyst, is exothermic, the conversion of Cu-S to Cu-A and the esterification reaction occur more efficiently at higher temperatures. When the coupling of phenylpropiolate salt and iodoethane was attempted in the absence of copper at room temperature, a minimal amount of ester (3%) was produced. The DFT calculations also confirmed the uphill nature of the Cu-S to Cu-A conversion and emphasized the necessity of elevated temperatures to facilitate the reaction toward Cu-A formation (Fig. S111, SI). Furthermore, the calculations supported a facile conversion of Cu-A to [Cu-L1]^+^ and acetylide ion in the absence of CO_2_ (Fig. S112, SI).

## Conclusions

We have discovered a new set of Cu-CNC complexes for the tandem carboxylation and esterification of terminal alkynes using CO_2_, and have experimentally established its catalytic reaction mechanism. The catalyst's structure and reaction mechanism are notably distinct from those of previously reported copper complexes. In contrast to other Cu-NHC complexes, wherein the [Cu(NHC)(OR)] (R = H, alkyl) is an active form of the catalyst, the Cu-CNC complexes perform through an aromatization-dearomatization mechanism. We have also obtained the enthalpy and entropy of formation, as well as the activation parameters, of a dearomatized Cu-CNC complex. These findings represent the first report of thermodynamic and kinetic data for a dearomatized complex supported by lutidine-linked pincer ligands. Analyzing the individual steps of the mechanism by NMR spectroscopy revealed the *in situ* formation of three reactive intermediates in the carboxylation process: Cu-S, Cu-A, and Cu-Pr. The Cu-S species was formed *via* nucleophilic attack by the dearomatized complex on phenylacetylene, in a reaction similar to that observed for a Rh-CNC analog. The symmetrically substituted Cu-S species, with two styrene linkers, is distinct from its unsymmetrical Rh-CNC counterpart because it can internally convert to a copper-acetylide intermediate. The Cu-A species is a vital intermediate that can react with CO_2_ and facilitates a subsequent carboxylation path. A similar reactivity and mechanism have not yet been documented with any other TM-CNC or carboxylation catalyst.

## Author contributions

N. B. conducted most of the experiments, including synthesis, characterization, catalytic reactions, and mechanistic investigations, in collaboration with E. G. He also contributed to the manuscript's writing under the supervision of L. T., alongside written contributions and edits from the other authors. C. Z. performed all DFT calculations under the guidance of S. P. de V.

## Conflicts of interest

The authors have no conflict of interest to declare.

## Supplementary Material

SC-OLF-D5SC08379F-s001

SC-OLF-D5SC08379F-s002

## Data Availability

CCDC 2415955, 2415975, 2488390, 2494324 and 2514575 contain the supplementary crystallographic data for this paper.^60*a*–*e*^ The data supporting this article are included in the supplementary information (SI). Supplementary information: synthetic procedures, structural characterizations (NMR spectra, mass data, X-ray data for [L3-Cu(PA)]_2_ and elemental analysis), UV-Vis studies of rate and equilibrium constants at different temperatures, NMR studies of the reaction mechanism, DFT procedures and data, crystallographic information files (CIF) for [Cu-L3]PF_6_, [Cu-L4]PF_6_, [Cu-L3*], and [Cu-L1*], [L3-Cu(PA)]_2_, and crystallographic data collection tables. See DOI: https://doi.org/10.1039/d5sc08379f.
