# Supplementary material for: Metal ligand cooperativity in the direct carboxylation and esterification of terminal alkynes by Cu-CNC complexes bearing 2,6-lutidine linkers
Source: Chem Sci. 2026 Jan 8;17(4):2087–101. doi: 10.1039/d5sc08379f (PMC12809709; doi:10.1039/d5sc08379f)
Supplement: SC-017-D5SC08379F-s001 [file SC-017-D5SC08379F-s001.pdf]

Supplementary Information (SI) for Chemical Science.
This journal is © The Royal Society of Chemistry 2026

Metal Ligand Cooperativity in the Direct Carboxylation and Esterification of Terminal Alkynes by Cu-CNC Complexes Bearing 2,6-Lutidine Linkers

*Nick Back[a], Emylie Guthrie[a], Chengxu Zhu[b,c], Sam P. de Visser[b,c], Laleh Tahsini[a]**

[a] Department of Chemistry, Oklahoma State University, Stillwater, Oklahoma, 74078, United States.

[b] Manchester Institute of Biotechnology, The University of Manchester, Manchester M1 7DN, United Kingdom. [c] Department of Chemical Engineering, The University of Manchester, Manchester M13 9PL, United Kingdom.

Electronic Supplementary Information

Table of Contents

Methods ... 3

Synthesis of ligand precursors, L3.2HBr and L4.2HBr ... 6

Synthesis of Cu-CNC complexes ... 6

Propiolate ester products ... 10

Single-crystal X-ray crystallography ... 20

- **Table S1.** Summary of X-ray crystallographic data collection parameters ... 23
- **Table S2.** Selected bond lengths and angles for the Cu-CNC complexes ... 24

NMR spectra ... 27

UV-Vis data ... 32

TD-DFT data ... 33

Kinetic Studies ... 34

NMR studies (mechanism) ... 37

NMR spectra of bis-imidazolium salts and Cu-CNC complexes ... 54

NMR spectra of propiolate ester products ... 74

DFT Calculations ... 103

METHODS

General procedure. The bis-imidazolium salts and copper complexes were synthesized under an argon atmosphere using standard air-sensitive techniques, including a Schlenk line and an MBraun glovebox. ***Caution!*** Extreme care should be taken when handling liquid nitrogen and using it in the Schlenk line trap to avoid condensation of oxygen from air. Solvents used in the syntheses and spectroscopic studies were distilled under N_2 and stored over molecular sieves in the glovebox. Potassium *tert*-butoxide and $[Cu(CH_3CN)_4]PF_6$ were obtained from Sigma-Aldrich and crystallized from tetrahydrofuran and acetonitrile solutions, respectively. 1-(2,6-diisopropylphenyl)imidazole and 1-(2,4,6-trimethylphenyl)imidazole were obtained from TCI America and used without further purification. The imidazolium salts **L1.**2HBr, **L2.**2HBr, and **L5.**2HBr and their corresponding Cu-CNC complexes were prepared using previously published procedures. [1, 2] The syntheses of **L3** and **L4** ligand precursors and their copper complexes have been described in the supporting information.

Instrumentation. NMR spectra were measured on a 400 MHz Bruker Avance III spectrometer and a 600 MHz Bruker Neo spectrometer. Chemical shifts (δ) for ^{1}H and ^{13}C NMR spectra were referenced to the resonance of TMS as an internal reference or the residual protio solvent. Elemental analyses were performed by Atlantic Microlabs, Inc. (Norcross, Georgia). UV−vis spectra were recorded on an Agilent Carry 8454 diode array spectrophotometer equipped with a UNISOKU Scientific Instruments Cryostat USP-203-B for low-temperature experiments.

Kinetic Measurements. The spectral change in the UV−visible was recorded on an Agilent Carry 8454 diode array spectrophotometer equipped with an Unisoku thermostatic cell holder for low-temperature experiments. In a typical experiment, the quartz cuvette is loaded with 3 mL of [Cu-**L1**]BArF in a degassed tetrahydrofuran solution. Aliquots of a pre-chilled KO^tBu stock solution in THF were then introduced into the copper solution via a needle. The reaction was monitored by the increase in absorbance at 505 nm ($\varepsilon = 2.0 \times 10^3\ M^{-1}\ cm^{-1}$), corresponding to the formation of the dearomatized species.

Room-Temperature Experiments Concerning the Formation of Cu-S and Cu-A in Solution. Under an argon atmosphere within a glovebox, [Cu-**L1**]PF_6 (0.027g, 0.05 mmol) was combined

with NaPA (**Cu-S**; 0.006g, 0.05 mmol, **Cu-A**; 0.031 g, 0.25 mmol) in 1 mL of CD_3CN, giving a yellow (**Cu-S**) and red (**Cu-A**) suspension. The reaction mixture was stirred for 24 hours in a sealed vial, then filtered into an NMR cell for analysis.

Room-Temperature Experiments Concerning the Conversion of Cu-S to Cu-A. Under an argon atmosphere within a glovebox, [Cu-**L3**]PF_6 (0.034g, 0.05 mmol) was combined with NaPA (0.012g, 0.05 mmol) in 1 mL of CD_3CN, giving an orange suspension. The reaction mixture was filtered through a filter pipette to remove the insoluble NaPA. The orange solution was left in a sealed vial overnight, after which it provided orange/red crystals that were collected and analyzed by X-ray crystallography.

Room-Temperature Experiments Concerning the Formation of [Cu-L3^{CO2}] in Solution. Under an argon atmosphere in a glovebox, [Cu-L3]PF6 (0.075g, 0.110 mmol) was combined with KOtBu (0.0615g, 0.548 mmol) in 1 mL of benzene, yielding a red suspension, which was filtered into a Schlenk flask. The flask was fully sealed with a septum, quickly removed from the glovebox, and exposed to CO_2 (1 atm) after multiple freeze-pump-thaw cycles. The resulting yellow/orange suspension was kept stirring under CO_2 for 30 min. After that time, the supernatant was removed using a cannula, and the orange solid was kept under vacuum until complete dryness. The resulting orange solid was transferred to the glovebox for NMR spectroscopy in CD_3CN.

Room-Temperature Experiments Concerning the Formation of Cu-Pr in Solution. Under an argon atmosphere within a glovebox, [Cu-**L1**]PF_6 (0.027g, 0.05 mmol) was combined with NaPA (0.006g, 0.05 mmol) in 1mL CD_3CN. The reaction mixture was stirred for 1 hour at room temperature, resulting in a dark yellow suspension, which was filtered into a J Young NMR cell and removed from the glovebox. The solution was exposed to CO_2 after multiple freeze-pump-thaw cycles and kept under this atmosphere for 30 minutes, after which the NMR and IR spectra were collected. This setup enables the observation of reaction dynamics and the identification of species present after carboxylation under controlled conditions.

Copper-Catalyzed Direct Carboxylation and Esterification of Terminal Alkynes. A Schlenk tube was charged with a Teflon stir bar, copper complex (0.068 mmol), and cesium carbonate (1.39 mmol) in 2 mL CH_3CN. To this suspension, phenylacetylene (0.684 mmol) and iodoethane (2.00 mmol) in 2mL CH_3CN were added. The tube was connected to a Schlenk line and degassed

through multiple freeze-pump-thaw cycles. It was then briefly placed under CO_2 (1 atm) while frozen and allowed to thaw to room temperature. The reaction mixture was stirred at room temperature for 12 hours, then heated at 80 °C for 12 hours. The crude mixture was then suspended in water, and the organic components were extracted using ethyl acetate. The solvent was then removed under vacuum, and the residue was purified by column chromatography to obtain an analytically pure product.

Synthesis of ligand precursors, L3.2HBr and L4.2HBr

*2,6-bis[(3-mesityl-2H-imidazol-1-yl)methyl]pyridine, I(Mes)$^{C^\wedge N^\wedge C}$·2HBr, **L3**·2HBr.* 2,6-dibromomethyl-pyridine (0.416 g, 1.57 mmol) was dissolved in 6 mL of 1,4-dioxane and added to 1-mesityl-1*H*-imidazole (0.731 g, 3.92 mmol) in 3 mL of 1,4-dioxane at room temperature. The reaction mixture was refluxed at 90 °C for 24 h, after which time it was filtered, and the resultant solid was rinsed with 1,4-dioxane and diethyl ether. The white solid was then collected with a yield of 85% (0.850 g). ^{1}H NMR (DMSO-d_6, 400 MHz): δ 9.57 (t, 0.5H; HCN^{Im}, *J = 1.6 Hz*), 8.09-7.93 (m, 3H; *p*-C_5H_3N, *m*-C_5H_3N, HCCH^{Im}), 7.52 (d, 1H; HCCH^{Im}, *J = 7.7 Hz*), 7.16 (s, 2H; CH^{Mes}), 5.65 (s, 2H; CH_2), 2.34 (s, 3H; Me^{Mes}), 2.03 (s, 6H; Me^{Mes}). ^{13}C NMR (DMSO-d_6, 101 MHz): δ 153.66, 140.28, 139.20, 138.30, 134.27, 131.10, 129.24, 123.94, 123.73, 122.30, 53.19, 20.58, 17.02. Elemental analysis (%) Calcd for $C_{31}H_{35}Br_2N_5$: C, 58.41; H, 5.53; N, 10.99. Found: C, 58.28; H, 5.55; N, 10.91.

*2,6-bis[(3-diisopropylphenyl-2H-imidazol-1-yl)methyl]pyridine, I(Dipp)$^{C^\wedge N^\wedge C}$·2HBr, **L4**·2HBr.* 2,6-dibromomethyl-pyridine (0.360 g, 1.36 mmol) was dissolved in 6 mL of 1,4-dioxane and added to a solution of 1-(2,6-diisopropylphenyl)-1*H*-imidazole (0.791 g, 3.47 mmol) in 2 mL of 1,4-dioxane at room temperature. The reaction mixture was refluxed at 90 °C for 24 h, after which time it was filtered, and the resultant solid was rinsed with 1,4-dioxane and diethyl ether. The fluffy white solid was collected at a yield of 80% (0.806 g). ^{1}H NMR (DMSO, 400 MHz): δ 9.71 (t, 0.5H; HCN^{Im}, *J = 1.6 Hz*), 8.21-8.05 (m, 3H; *p*-C_5H_3N, *m*-C_5H_3N, HCCH^{Im}), 7.64 (t, 1H; p-Dipp, *J = 7.8 Hz*), 7.51 (d, 1H; HCCH^{Im}, *J = 7.7 Hz*), 7.47 (d, 2H; m-Dipp, *J = 7.8 Hz*), 5.67 (s, 2H; CH_2), 2.30 (sept, 2H; CH^{Dipp}, *J = 6.9 Hz*), 1.15 (d, 6H; Me^{Dipp}, *J = 6.8*), 1.14 (d, 6H; Me^{Dipp}, *J = 6.8*). ^{13}C NMR (DMSO, 101 MHz): δ 153.70, 145.04, 139.39, 138.57, 131.53, 130.56, 125.23, 124.45, 123.85, 122.32, 53.47, 28.10, 23.74 (d, *J* = 5.8 Hz). Elemental analysis (%) Calcd for $C_{37}H_{47}Br_2N_5$: C, 61.58; H, 6.57; N, 9.71. Found: C, 61.42; H, 6.56; N, 9.57.

Synthesis of Cu-CNC complexes*.*

*[Cu(IMes$^{C^\wedge N^\wedge C}$)]PF$_6$, [Cu-**L3**]PF$_6$.* A portion of I(Mes)$^{C^\wedge N^\wedge C}$·2HBr (0.280 g, 0.439 mmol) was suspended in THF and combined with KO^{t}Bu (0.103 g, 0.921 mmol) in THF. A solution of $[Cu(CH_3CN)_4]PF_6$ (0.163 g, 0.439 mmol) in CH_3CN was added to this suspension, and the reaction mixture was allowed to stir for 24 h. The crude product was then filtered through a glass frit, rinsed with THF and CH_3CN, and kept under vacuum to complete dryness. The resulting yellow

solid from the filtrate was triturated with THF and hexane, filtered through the frit funnel, washed with THF, and dried under vacuum. The product was collected as yellow solid in a 92% yield (0.275 g). ^{1} H NMR (CD_3CN, 400 MHz): δ 7.96 (t, 0.5H; *p*-C_5H_3N, *J = 7.8 Hz*), 7.58 (d, 1H; *m*-C_5H_3N, *J = 7.8 Hz*), 7.39 (d, 1H; $HCCH^{Im}$, *J = 1.8 Hz*), 6.99 (d, 1H; $HCCH^{Im}$, *J = 1.8 Hz*), 6.84 (s, 2H; CH^{Mes}), 5.36 (s, 2H; CH_2N), 2.38 (s, 3H, Me^{Mes}), 1.67 (s, 6H, Me^{Mes}). ^{13}C NMR (CD_3CN, 101 MHz): δ 154.91 (C_1^{Mes}), 140.63 (*p*-C_5H_3N), 139.28 (*o*-C_5H_3N), 137.01 (*o*-Mes), 135.20 (*p*-Mes), 129.51 (CH^{Mes}), 124.84 (*m*-C_5H_3N), 123.55 ($HCCH^{Im}$), 122.10 ($HCCH^{Im}$), 55.06 (CH_2N), 21.17 (Me^{Mes}), 17.51 (Me^{Mes}). Elemental analysis (%) Calcd for $C_{31}H_{33}CuF_6N_5P$: C, 54.42; H, 4.86; N, 10.24. Found: C, 54.18; H, 4.84; N, 10.17.

*[Cu(IDipp$^{C\wedge N\wedge C}$)]PF$_6$·H$_2$O, [Cu-**L4**]PF$_6$.* A portion of I(Dipp)$^{C\wedge N\wedge C}$·2HBr (0.640 g, 1.32 mmol) was suspended in THF and combined with KO^{t}Bu (0.311 g, 2.77 mmol) in THF. A solution of [Cu(CH_3CN)$_4$]PF_6 (0.492 g, 1.32 mmol) in CH_3CN was added to this suspension, and the reaction mixture was allowed to stir for 24 h. The dark-brown mixture was then filtered through a glass frit, rinsed with CH_3CN, and kept under vacuum to complete dryness. The resulting solid was recrystallized from a mixture of CH_3CN and Et_2O affording the product as yellow solid in 86% yield (0.605 g). ^{1} H NMR (CD_3CN, 400 MHz): δ 7.98 (t, 1H; *p*-C_5H_3N, *J = 7.8 Hz*), 7.60 (d, 2H; *m*-C_5H_3N, *J = 7.8 Hz*), 7.42 (d, 2H; $HCCH^{Im}$, *J = 1.8 Hz*), 7.34 (t, 2H; *p*-Dipp, *J = 7.8 Hz*), 7.14 (d, 4H, *m*-Dipp, *J = 7.8 Hz*), 7.06 (d, 2H; $HCCH^{Im}$, *J = 1.7 Hz*), 5.28 (s, 4H; CH_2N), 2.12 (sept, 4H, CH^{Dipp}, *J = 7.0 Hz*), 1.02 (d, 12H, Me^{Dipp}, *J = 6.9 Hz*), 0.76 (d, 12H, Me^{Dipp}, *J = 6.8 Hz*). ^{13}C NMR (CD_3CN, 101 MHz): δ 183.28 (C_2^{NHC}), 153.76 ($C1^{Dipp}$), 146.50 (o-Dipp), 140.70 (*p*-C_5H_3N), 136.36 (*o*-C_5H_3N), 130.71 (*p*-Dipp), 124.43 (*m*-Dipp), 124.27 (*m*-C_5H_3N), 123.78 ($HCCH^{Im}$), 122.65 ($HCCH^{Im}$), 54.49 (CH_2N), 28.95 (CH^{Dipp}), 24.84 (Me^{Dipp}), 23.55 (Me^{Dipp}). Elemental analysis (%) Calcd for $C_{37}H_{47}CuF_6N_5OP$: C, 56.52; H, 6.02; N, 8.91. Found: C, 56.45; H, 5.93; N, 9.07.

Synthesis of [Cu(I^{i}Pr$^{C\wedge N\wedge C}$)]·0.05THF·1.05H$_2$O, [Cu-**L1***].* A portion of [Cu(I^{i}Pr$^{C\wedge N\wedge C}$)]PF_6 (0.0750 g, 0.141 mmol) was combined with a solution of KO^{t}Bu (0.316, 2.82 mmol) in 1mL THF, affording a dark red suspension. The reaction mixture was filtered through a filter pipette, and hexanes was added to the filtrate. The solution was left in the freezer at −30 °C overnight, after which the red supernatant was decanted off and the solid was dried under vacuum. The crude solid was then recrystallized from a mixture of THF and hexanes at −30 °C affording deep red

crystals at a 55% yield (0.0330 g). ^{1}H NMR (THF-d_8, 600 MHz): δ 7.10–7.07 (m, 2H, HCCH^{Im}), 7.01 (d, 1H, HCCH^{Im}, J = 1.7 Hz), 6.96 (d, 1H, HCCH^{Im}, J = 1.8 Hz), 6.16 (dd, 1H, p-C_5H_3N, J = 9.0, 6.1 Hz), 5.83 (d, 1H, m-C_5H_3N, J = 9.1), 5.54 (s, 1H), 5.11 (d, 1H, m-C_5H_3N, J = 6.1 Hz), 5.07 (sept, 1H, CH^{iPr}, J = 6.9 Hz), 4.97 (sept, 1H, CH^{iPr}, J = 6.9 Hz), 4.48 (s, 2H), 1.48 (d, 6H, Me^{iPr},6.8 Hz), 1.46 (d, 6H, Me^{iPr},6.9 Hz). ^{13}C NMR (THF-d_8, 150 MHz): δ 184.49 (C_2^{NHC}), 177.64 (C_2^{NHC}), 150.82 (o-C_5H_3N), 146.69 (o-C_5H_3N), 128.96 (p-C_5H_3N), 122.19 (HCCH^{Im}), 119.29 (HCCH^{Im}), 118.32 (m-C_5H_3N), 115.58 (HCCH^{Im}), 113.65 (HCCH^{Im}), 97.70 (m-C_5H_3N), 89.64 (=CHN), 68.53 (THF), 56.93 (CH_2N), 54.03 (CH^{iPr}), 53.22 (CH^{iPr}), 37.11 (Me^{iPr}), 36.62 (KO^{t}Bu), 25.83 (THF), 24.05 (Me^{iPr}). Elemental analysis (%) Calcd for $C_{19.2}H_{26.5}CuN_5O_{1.1}$: C, 56.45; H, 6.54; N, 17.14. Found: C, 56.30; H, 6.13; N, 16.87.

Synthesis of [Cu(IMes$^{C\wedge N\wedge C}$)]·1.0H$_2$O, [Cu-**L3***].* A portion of [Cu(IMes$^{C\wedge N\wedge C}$)]PF_6 (0.075 g, 0.110 mmol) was suspended in THF and combined with a solution of KO^{t}Bu (0.019 g, 0.164 mmol) in THF, affording a red suspension. The reaction mixture was filtered using a filter pipette, and hexanes were added to the filtrate. The crashed solid was removed, nd the supernatant was left in the freezer at −30 °C overnight affording bright red crystals at a 58% yield (0.034 g). ^{1}H NMR (THF-d_8, 400 MHz): δ 7.23 (d, 1H, HCCH^{Im}, J = 1.7 Hz,), 7.07 (d, 1H, HCCH^{Im}, J = 1.7 Hz), 6.84 (d, 1H, HCCH^{Im}, J = 1.8 Hz,), 6.78 (s, 2H, CH^{Mes}), 6.76 (d, 1H, HCCH^{Im}, J = 1.7 Hz), 6.72 (s, 2H, CH^{Mes}), 6.24 (dd, 1H, p-C_5H_3N, J = 9.0, 6.2 Hz), 5.90 (dd, 1H, m-C_5H_3N, J = 9.0, 1.0 Hz), 5.61 (s, 1H, =CHN), 5.19 (d, 1H, m-C_5H_3N, J = 6.1 Hz), 4.57 (s, 2H, CH_2N), 2.42 (s, 3H, Me^{Mes}), 2.39 (s, 3H, Me^{Mes}), 1.69 (s, 6H, Me^{Mes}), 1.67 (s, 6H, Me^{Mes}). ^{13}C NMR (THF-d_8, 150 MHz): δ 185.28 (C_2^{NHC}), 177.34 (C_2^{NHC}), 149.46 (o-C_5H_3N), 145.80 (o-C_5H_3N), 138.02 (o-Mes), 137.21 (C_1^{Mes}), 136.68 (p-Mes), 136.03 (p-Mes), 135.19 (o-Mes), 134.53 (C_1^{Mes}), 128.48 (CH^{Mes}), 128.09 (p-C_5H_3N), 128.07 (CH^{Mes}), 121.16 (HCCH^{Im}), 119.51 (HCCH^{Im}), 117.87 (HCCH^{Im}), 117.38 (HCCH^{Im}), 117.02 (m-C_5H_3N), 96.83 (m-C_5H_3N), 88.62 (=CHN), 55.96 (CH_2N), 20.34 (Me^{Mes}), 20.32 (Me^{Mes}), 17.06 (Me^{Mes}), 16.83 (Me^{Mes}). Elemental analysis (%) Calcd for $C_{31}H_{34}CuN_5O$: C, 66.94; H, 6.16; N, 12.59. Found: C, 66.91; H, 5.89; N, 12.39.

*[Cu(I^{i}Pr$^{C\wedge N\wedge C}$)]BArF, [Cu-**L1**]BArF.* A portion of I(^{i}Pr)$^{C\wedge N\wedge C}$•2HBr (0.100 g, 0.206 mmol) was suspended in THF and combined with a portion of KO^{t}Bu (0.049 g, 0.433 mmol) dissolved in THF. A solution of [Cu(CH_3CN)$_4$]BArF (0.187 g, 0.206 mmol) in THF was added to this suspension, and the reaction mixture was allowed to stir for 24 h. The crude product was then filtered through

Celite, rinsed with THF, and kept under vacuum to complete dryness. The collected solid from the filtrate was crystallized from THF and hexane to afford the product in a 96% yield (0.212 g). ^{1}H NMR (THF-d_8, 600 MHz): 7.94 (t, 1H, *p*-C_5H_3N, *J* = 7.7 Hz), 7.57 (d, 2H, *m*-C_5H_3N, *J* = 7.7 Hz), 7.36 (s, 4H, $HCCH^{Im}$), 5.33 (s, 4H, CH_2N), 4.84 (sept, 2H, $CH^{iPr,}$ *J* = 6.8 Hz), 1.52 (d, 12H, Me^{iPr}, *J* = 6.8 Hz). ^{13}C NMR (THF-d_8, 150 MHz): δ 179.07 (C_2^{NHC}), 155.52 (*o*-C_5H_3N), 140.82 (*p*-C_5H_3N), 124.79 (*m*-C_5H_3N), 123.68 ($HCCH^{Im}$), 117.57 ($HCCH^{Im}$), 55.36 (CH^{iPr}), 54.90 (CH_2N), 23.95 (Me^{iPr}). Elemental analysis (%) Calcd for $C_{43}H_{25}BCuF_{20}N_5$: C, 48.45; H, 2.36; N, 6.57. Found: C, 48.42; H, 2.31; N, 6.39.

*[Cu(IMes$^{C\wedge N\wedge C}$)]BArF, [Cu-**L3**]BArF*. A portion of IMes$^{C\wedge N\wedge C}$•2HBr (0.122 g, 0.192 mmol) was suspended in THF and combined with a portion of KO^{t}Bu (0.047 g, 0.422 mmol) dissolved in THF. A solution of $[Cu(CH_3CN)_4]$BArF (0.174 g, 0.192 mmol) in THF was added to this suspension, and the reaction mixture was allowed to stir for 24 h. The crude product was then filtered through Celite, rinsed with THF, and kept under vacuum to complete dryness. The yellow solid collected from the filtrate was crystallized from THF and hexane to afford the product in an 82% yield (0.191 g). ^{1}H NMR (THF-d_8, 600 MHz): δ 7.97 (t, 1H, *p*-C_5H_3N, *J* = 7.8 Hz), 7.60 (d, 2H, *m*-C_5H_3N, *J* = 7.8 Hz), 7.51 (d, 2H, $HCCH^{Im}$, *J* = 1.8 Hz), 7.15 (d, 2H, $HCCH^{Im}$, *J* = 1.8 Hz), 6.84 (s, 4H, CH^{Mes}), 5.46 (s, 4H, CH_2N), 2.40 (s, 6H, Me^{Mes}), 1.68 (s, 12H, Me^{Mes}). ^{13}C NMR (THF-d_8, 150 MHz): δ 181.30 (C_2^{NHC}), 155.36 (C_1^{Mes}), 140.73 (*p*-C_5H_3N), 139.35 (*o*-Mes), 137.39 (*o*-C_5H_3N), 135.27 (*p*-Mes), 129.83 (CH^{Mes}), 124.85 (*m*-C_5H_3N), 123.73 ($HCCH^{Im}$), 122.57 ($HCCH^{Im}$), 55.20 (CH_2N), 21.33 (Me^{Mes}), 17.72 (Me^{Mes}). Elemental analysis (%) Calcd for $C_{55}H_{33}BCuF_{20}N_5$: C, 54.23; H, 2.73; N, 5.75. Found: C, 54.15; H, 2.78; N, 5.76.

Propiolate ester products.

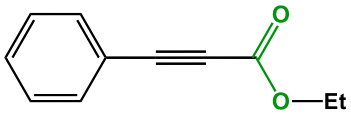

Ethyl 3-phenylpropiolate

Following the general procedure, the reaction of phenylacetylene, CO_2, and iodoethane provided the desired product with an average yield of 88%. ^{1}H NMR (400 MHz, $CDCl_3$): δ 7.61–7.54 (m, 2H), 7.48–7.40 (m, 1H), 7.40–7.31 (m, 2H), 4.29 (q, 2H, *J* = 7.1 Hz), 1.34 (t, 3H, *J* = 7.1 Hz). ^{13}C NMR (101 MHz, $CDCl_3$): δ 154.20, 133.10, 130.72, 128.68, 119.76, 86.17, 80.83, 62.21, 14.21. GC/MS: retention time = 16.1 min, M/Z = 174.

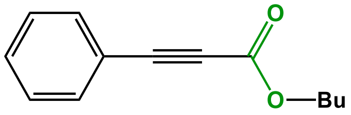

Butyl 3-phenylpropiolate

Following the general procedure, the reaction of phenylacetylene, CO_2, and iodobutane provided the desired product with an average yield of 96%. ^{1}H NMR (400 MHz, $CDCl_3$): 7.62–7.55 (m, 2H), 7.48–7.40 (m, 1H), 7.40–7.32 (m, 2H), 4.24 (t, 2H, *J* = 6.7 Hz), 1.70 (ddt, 2H, *J* = 8.9, 7.8, 6.5 Hz), 1.50–1.35 (m, 2H), 0.96 (t, 3H, *J* = 7.4 Hz). ^{13}C NMR (400 MHz, $CDCl_3$): ^{13}C NMR (101 MHz, $CDCl_3$) δ 154.36, 133.10, 130.71, 128.68, 119.80, 86.18, 80.84, 66.09, 30.60, 19.18, 13.79. GC/MS: retention time = 18.6 min, M/Z = 201.

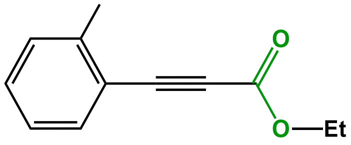

Ethyl 3-(o-tolyl)propiolate

Following the general procedure, the reaction of 2-ethynyltoluene, CO_2, and iodoethane provided the desired product with an average yield of 68%. ^{1}H NMR (400 MHz, $CDCl_3$): δ 7.54 (dd, 1H, *J* = 7.7, 1.4 Hz), 7.33 (td, 1H, *J* = 7.6, 1.5 Hz), 7.24 (d, 1H, *J* = 7.6 Hz), 7.21–7.14 (m, 1H), 4.30 (q, 2H, *J* = 7.2 Hz), 2.49 (s, 3H), 1.36 (t, 3H, *J* = 7.1 Hz). ^{13}C NMR (101 MHz, $CDCl_3$): δ 154.35, 142.34, 133.51, 130.69, 129.87, 125.90, 119.62, 85.23, 84.55, 62.15, 20.69, 14.25. GC/MS: retention time = 16.9 min, M/Z = 188.

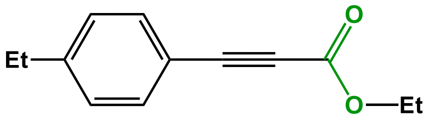

Ethyl 3-(4-ethylphenyl)propiolate

Following the general procedure, the reaction of 4-ethynyltoluene, CO_2, and iodoethane provided the desired product with an average yield of 67%. ^{1}H NMR (400 MHz, $CDCl_3$): δ 7.54–7.47 (m, 1H), 7.23–7.17 (m, 1H), 4.29 (q, 1H, *J* = 7.1 Hz), 2.67 (q, 1H, *J* = 7.6 Hz), 1.35 (t, 2H, *J* = 7.1 Hz), 1.23 (t, 2H, *J* = 7.6 Hz). ^{13}C NMR (101 MHz, $CDCl_3$): δ 154.36, 147.58, 133.23, 128.30, 116.86, 86.77, 80.48, 62.13, 29.11, 15.26, 14.24. GC/MS: retention time = 18.9 min, M/Z = 202.

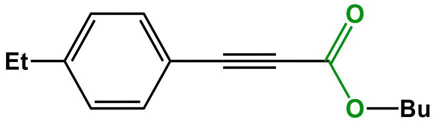

Butyl 3-(4-ethylphenyl)propiolate

Following the general procedure, the reaction of 4-ethynyltoluene, CO_2, and iodobutane provided the desired product with an average yield of 99%. ^{1}H NMR (400 MHz, $CDCl_3$): δ 7.54–7.47 (m, 2H), 7.23–7.17 (m, 2H), 4.23 (t, 2H, *J* = 6.7 Hz), 2.67 (q, 2H, *J* = 7.6 Hz), 1.70 (dq, 2H, *J* = 8.7, 6.8 Hz), 1.51–1.37 (m, 2H), 1.23 (t, 3H, *J* = 7.6 Hz), 0.96 (t, 3H, *J* = 7.4 Hz). ^{13}C NMR (101 MHz, $CDCl_3$): δ 154.52, 147.56, 133.23, 128.29, 116.89, 86.78, 80.49, 66.00, 30.63, 29.11, 19.20, 15.26, 13.80. GC/MS: retention time = 21.2 min, M/Z = 230.

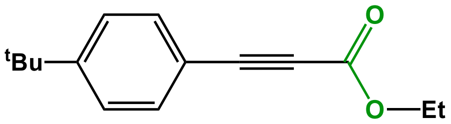

Ethyl 3-(4-(tert-butyl)phenyl)propiolate

Following the general procedure, the reaction of 4-tert-butylphenylacetylene, CO_2, and iodoethane provided the desired product with an average yield of 99%. ^{1}H NMR (400 MHz, $CDCl_3$): δ 7.56–7.46 (m, 2H), 7.42–7.36 (m, 2H), 4.29 (q, 2H, *J* = 7.1 Hz), 1.35 (t, 3H, *J* = 7.1 Hz), 1.31 (s, 9H). ^{13}C NMR (101 MHz, $CDCl_3$): δ 154.42, 154.38, 133.02, 125.76, 116.66, 86.73, 80.47, 62.13, 35.18, 31.18, 14.25. GC/MS: retention time = 20.5 min, M/Z = 230.

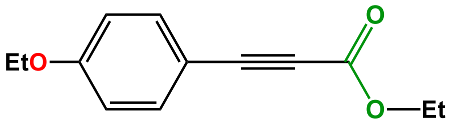

Ethyl 3-(4-ethoxyphenyl)propiolate

Following the general procedure, the reaction of 1-ethoxy-4-ethynylbenzene, CO_2, and iodoethane provided the desired product with an average yield of 89%. ^1H NMR (400 MHz, $CDCl_3$): δ 7.56–7.48 (m, 2H), 6.90–6.82 (m, 2H), 4.28 (q, 2H, *J* = 7.1 Hz), 4.05 (q, 2H, *J* = 7.0 Hz), 1.42 (t, 3H, *J* = 7.0 Hz), 1.35 (t, 3H, *J* = 7.1 Hz). ^{13}C NMR (101 MHz, $CDCl_3$): δ 161.04, 154.50, 135.05, 114.84, 111.29, 87.17, 80.23, 63.80, 62.03, 14.78, 14.25. GC/MS: retention time = 20.6 min, M/Z = 218.

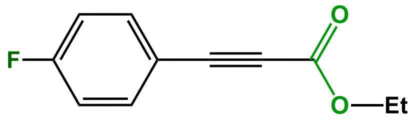

Ethyl 3-(4-fluorophenyl)propiolate

Following the general procedure, the reaction of 1-ethynyl-4-fluorobenzene, CO_2, and iodoethane provided the desired product with an average yield of 90%. ^1H NMR (400 MHz, $CDCl_3$): δ 7.63–7.53 (m, 2H), 7.12–7.02 (m, 2H), 4.29 (q, 2H, *J* = 7.1 Hz), 1.35 (t, 3H, *J* = 7.1 Hz). ^{13}C NMR (101 MHz, $CDCl_3$): δ 165.29, 162.77, 154.10, 135.38, 116.25, 85.13, 80.75, 62.28, 14.22. GC/MS: retention time = 15.8 min, M/Z = 192.

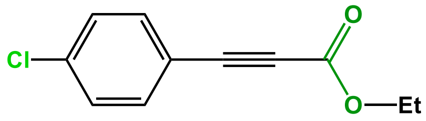

Ethyl 3-(4-chlorophenyl)propiolate

Following the general procedure, the reaction of 1-chloro-4-ethynylbenzene, CO_2, and iodoethane provided the desired product with an average yield of 68%. ^1H NMR (400 MHz, $CDCl_3$): δ 7.55–7.47 (m, 2H), 7.39–7.32 (m, 2H), 4.30 (q, 2H, *J* = 7.1 Hz), 1.35 (t, 3H, *J* = 7.1 Hz). ^{13}C NMR (101 MHz, $CDCl_3$): δ 153.99, 137.16, 134.30, 129.19, 118.25, 84.85, 81.63, 62.36, 14.21. GC/MS: retention time = 18.3 min, M/Z = 208.

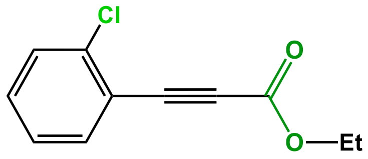

Ethyl 3-(2-chlorophenyl)propiolate

Following the general procedure, the reaction of 1-chloro-2-ethynylbenzene, CO_2, and iodoethane provided the desired product with an average yield of 89%. ^1H NMR (400 MHz, $CDCl_3$): δ 7.60 (dd, 1H, *J* = 7.7, 1.7 Hz), 7.44 (dd, 1H, *J* = 8.1, 1.3 Hz), 7.37 (td, 1H, *J* = 7.8, 1.7 Hz), 7.31–

7.22 (m, 1H), 4.31 (q, 2H, *J* = 7.1 Hz), 1.36 (t, 3H, *J* = 7.1 Hz). ^{13}C NMR (101 MHz, $CDCl_3$): δ 153.91, 137.43, 134.79, 131.68, 129.73, 126.78, 120.12, 85.09, 82.36, 62.42, 14.22. GC/MS: retention time = 18.3 min, M/Z = 208.

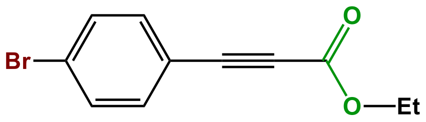

Ethyl 3-(4-bromophenyl)propiolate

Following the general procedure, the reaction of 1-bromo-4-ethynylbenzene, CO_2, and iodoethane provided the desired product with an average yield of 62%. ^{1}H NMR (400 MHz, $CDCl_3$): δ 7.55–7.48 (m, 2H), 7.47–7.39 (m, 2H), 4.29 (q, 2H, *J* = 7.1 Hz), 1.35 (t, 3H, *J* = 7.1 Hz). ^{13}C NMR (101 MHz, $CDCl_3$): δ 153.97, 134.39, 132.11, 125.53, 118.70, 84.87, 81.74, 62.35, 14.20. GC/MS: retention time = 19.5 min, M/Z = 254.

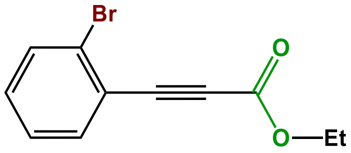

Ethyl 3-(2-bromophenyl)propiolate

Following the general procedure, the reaction of 1-bromo-2-ethynylbenzene, CO_2, and iodoethane provided the desired product with an average yield of 83%. ^{1}H NMR (400 MHz, $CDCl_3$): δ 7.60 (dd, 1H, *J* = 7.7, 1.7 Hz), 7.44 (dd, 1H, *J* = 8.1, 1.3 Hz), 7.37 (td, 1H, *J* = 7.8, 1.7 Hz), 7.31–7.22 (m, 1H), 4.31 (q, 2H, *J* = 7.1 Hz), 1.36 (t, 3H, *J* = 7.1 Hz). ^{13}C NMR (101 MHz, $CDCl_3$): δ 153.91, 137.43, 134.79, 131.67, 129.73, 126.79, 120.12, 85.10, 82.36, 62.42, 14.22. GC/MS: retention time = 19.6 min, M/Z = 254.

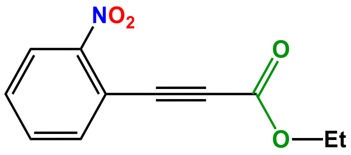

Ethyl 3-(2-nitrophenyl)propiolate

Following the general procedure, the reaction of 1-ethynyl-2-nitrobenzene, CO_2, and iodoethane provided the desired product with an average yield of 67%. ^{1}H NMR (400 MHz, $CDCl_3$): δ 8.16 (d, 1H, *J* = 8.1 Hz), 7.78 (dd, 1H, *J* = 7.6, 1.7 Hz), 7.64 (dtd, 2H, *J* = 23.1, 7.6, 1.5 Hz), 4.33 (q, 2H, *J* =

7.1 Hz), 1.37 (t, 3H, J = 7.1 Hz). ^{13}C NMR (101 MHz, $CDCl_3$): δ 153.55, 135.89, 133.39, 131.02, 125.18, 115.71, 86.95, 80.51, 62.67, 29.84, 14.19. GC/MS: retention time = 21.2 min, M/Z = 219.

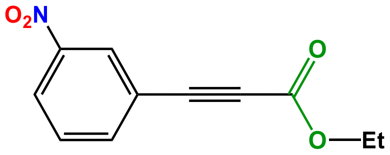

Ethyl 3-(3-nitrophenyl)propiolate

Following the general procedure, the reaction of 1-ethynyl-3-nitrobenzene, CO_2, and iodoethane provided the desired product with an average yield of 98%. ^{1}H NMR (400 MHz, $CDCl_3$): δ 8.43 (t, 1H, J = 1.9 Hz), 8.30 (ddd, 1H, J = 8.3, 2.3, 1.1 Hz), 7.92–7.85 (m, 1H), 7.59 (t, 1H, J = 8.0 Hz), 4.32 (q, 2H, J = 7.1 Hz), 1.37 (t, 3H, J = 7.1 Hz). ^{13}C NMR (101 MHz, $CDCl_3$): δ 153.47, 148.26, 138.43, 129.95, 127.79, 125.31, 121.72, 82.62, 82.50, 62.66, 14.17. GC/MS: retention time = 21.0 min, M/Z = 219.

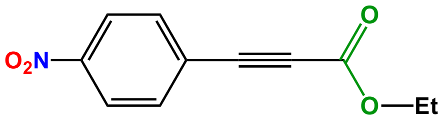

Ethyl 3-(4-nitrophenyl)propiolate

Following the general procedure, the reaction of 1-ethynyl-4-nitrobenzene, CO_2, and iodoethane provided the desired product with an average yield of 88%. ^{1}H NMR (400 MHz, $CDCl_3$): δ 8.28–8.20 (m, 2H), 7.78–7.70 (m, 2H), 4.32 (q, 2H, J = 7.2 Hz), 1.36 (t, 3H, J = 7.2 Hz). ^{13}C NMR (101 MHz, $CDCl_3$): δ 153.42, 148.62, 133.80, 126.45, 123.87, 84.36, 82.83, 62.72, 14.16. GC/MS: retention time = 21.0 min, M/Z = 219.

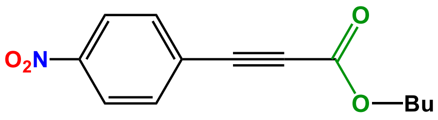

Butyl 3-(4-nitrophenyl)propiolate

Following the general procedure, the reaction of 1-ethynyl-4-nitrobenzene, CO_2, and iodobutane provided the desired product with an average yield of 99%. ^{1}H NMR (400 MHz, $CDCl_3$): δ 8.29–8.20 (m, 2H), 7.79–7.68 (m, 2H), 4.27 (t, 2H, J = 6.7 Hz), 1.77 – 1.65 (m, 2H), 1.51–1.38 (m, 2H), 0.97 (t, 3H, J = 7.4 Hz). ^{13}C NMR (101 MHz, $CDCl_3$): δ 153.58, 148.63, 133.82, 126.51, 123.88, 84.39, 82.87, 66.57, 30.54, 19.17, 13.77. GC/MS: retention time = 23.1 min, M/Z = 246.

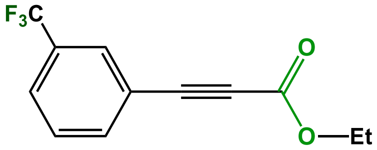

Ethyl 3-(3-(trifluoromethyl)phenyl)propiolate

Following the general procedure, the reaction of 1-ethynyl-3-(trifluoromethyl)benzene, CO_2, and iodoethane provided the desired product with an average yield of 77%. ^{1}H NMR (400 MHz, $CDCl_3$): δ 7.84 (d, 1H, *J* = 2.1 Hz), 7.75 (d, 1H, *J* = 7.7 Hz), 7.69 (ddt, 1H, *J* = 7.9, 1.8, 0.9 Hz), 7.52 (td, 1H, *J* = 7.9, 1.0 Hz), 4.31 (q, 2H, *J* = 7.1 Hz), 1.36 (t, 3H, *J* = 7.1 Hz). ^{13}C NMR (101 MHz, $CDCl_3$): δ 153.74, 136.04, 131.49 (q, *J* = 33.3 Hz), 129.85 (q, *J* = 4.0 Hz), 129.37, 127.27 (q, *J* = 3.7 Hz), 124.84, 122.14, 83.89, 81.87, 62.50, 14.19. GC/MS: retention time = 15.4 min, M/Z = 242.

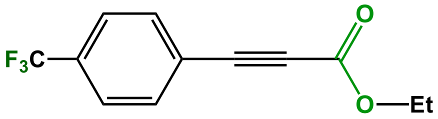

Ethyl 3-(4-(trifluoromethyl)phenyl)propiolate

Following the general procedure, the reaction of 1-ethynyl-4-(trifluoromethyl)benzene, CO_2, and iodoethane provided the desired product with an average yield of 84%. ^{1}H NMR (400 MHz, $CDCl_3$): δ 7.69 (d, 2H, *J* = 8.6 Hz), 7.64 (d, 2H, *J* = 8.3 Hz), 4.31 (q, 2H, *J* = 7.1 Hz), 1.36 (t, 3H, *J* = 7.1 Hz). ^{13}C NMR (101 MHz, $CDCl_3$): δ 153.73, 133.29, 132.31 (q, *J* = 32.9 Hz), 125.68 (q, *J* = 3.7 Hz), 123.63, 122.32, 83.93, 82.44, 62.53, 14.19. GC/MS: retention time = 15.4 min, M/Z = 242.

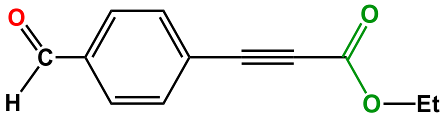

Ethyl 3-(4-formylphenyl)propiolate

Following the general procedure, the reaction of 4-ethynylbenzaldehyde, CO_2, and iodoethane provided the desired product with an average yield of 80%. ^{1}H NMR (400 MHz, $CDCl_3$): δ 10.04 (s, 1H), 7.93–7.86 (m, 2H), 7.77–7.70 (m, 2H), 4.32 (q, 2H, *J* = 7.2 Hz), 1.37 (d, 4H, *J* = 14.3 Hz). ^{13}C NMR (101 MHz, $CDCl_3$): δ 191.27, 153.72, 137.17, 133.52, 129.68, 125.76, 84.23, 83.47, 62.57, 14.20. GC/MS: retention time = 19.8 min, M/Z = 202.

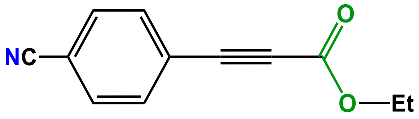

Ethyl 3-(4-cyanophenyl)propiolate

Following the general procedure, the reaction of 4-ethynylbenzonitrile, CO_2, and iodoethane provided the desired product with an average yield of 93%. ^{1}H NMR (400 MHz, $CDCl_3$): δ 7.67 (s, 4H), 4.31 (q, 2H, *J* = 7.1 Hz), 1.36 (t, 3H, *J* = 7.1 Hz). ^{13}C NMR (101 MHz, $CDCl_3$): δ 153.49, 133.39, 132.34, 124.60, 117.97, 114.12, 83.84, 83.21, 62.64, 14.16. GC/MS: retention time = 19.6 min, M/Z = 199.

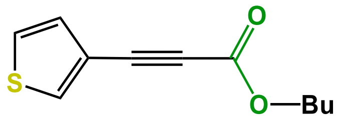

Butyl 3-(thiophen-3-yl)propiolate

Following the general procedure, the reaction of 3-ethynylthiophene, CO_2, and iodobutane provided the desired product with an average yield of 88%. ^{1}H NMR (400 MHz, $CDCl_3$): δ 7.75 (dd, 1H, *J* = 3.0, 1.1 Hz), 7.32 (dd, 1H, *J* = 5.1, 3.0 Hz), 7.23 (dd, 1H, *J* = 5.0, 1.1 Hz), 4.23 (t, 2H, *J* = 6.7 Hz), 1.69 (m, 2H, *J* = 6.9 Hz), 1.43 (m, 2H, *J* = 7.4 Hz), 0.96 (t, 3H, *J* = 7.4 Hz). ^{13}C NMR (101 MHz, $CDCl_3$): δ 154.40, 133.83, 130.34, 126.18, 119.05, 81.66, 80.88, 66.07, 30.61, 19.19, 13.79. GC/MS: retention time = 19.2 min, M/Z = 208.

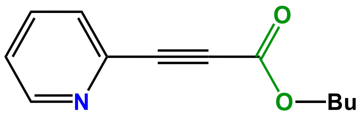

Butyl 3-(pyridin-2-yl)propiolate

Following the general procedure, the reaction of 2-ethynylpyridine, CO_2, and iodoebutane provided the desired product with an average yield of 40%. ^{1}H NMR (400 MHz, $CDCl_3$): δ 8.65 (ddd, 1H, *J* = 4.9, 1.8, 1.0 Hz), 7.72 (td, 1H, *J* = 7.7, 1.8 Hz), 7.59 (dt, 1H, *J* = 7.8, 1.1 Hz), 7.35 (ddd, 1H, *J* = 7.6, 4.8, 1.2 Hz), 4.24 (t, 2H, *J* = 6.7 Hz), 1.73–1.64 (m, 2H), 1.48–1.38 (m, 2H), 0.95 (t, 3H, *J* = 7.4 Hz). ^{13}C NMR (101 MHz, $CDCl_3$): δ 153.75, 150.63, 140.70, 136.48, 128.67, 124.75, 83.85, 79.34, 66.30, 30.50, 19.12, 13.73. GC/MS: retention time = 20.1 min, M/Z = 203.

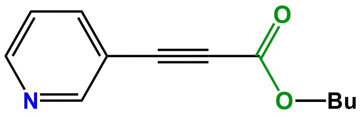

Butyl 3-(pyridin-3-yl)propiolate

Following the general procedure, the reaction of 3-ethynylpyridine, CO_2, and iodobutane provided the desired product with an average yield of 44%. ^1H NMR (400 MHz, $CDCl_3$): δ 8.81 (s, 1H), 8.66 (d, 1H, J = 4.6 Hz), 7.87 (dt, 1H, J = 7.9, 1.8 Hz), 7.33 (dd, 1H, J = 7.9, 4.8 Hz), 4.25 (t, 2H, J = 6.7 Hz), 1.76 – 1.65 (m, 2H), 1.44 (m, 2H, J = 7.4 Hz), 0.96 (t, 3H, J = 7.4 Hz). ^{13}C NMR (101 MHz, $CDCl_3$): δ 153.82, 153.49, 150.82, 139.97, 123.35, 117.25, 83.71, 82.45, 66.38, 30.56, 19.17, 13.78. GC/MS: retention time = 19.2 min, M/Z = 203.

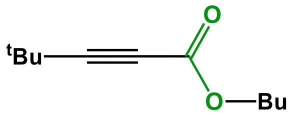

Butyl 4,4-dimethylpent-2-ynoate

Following the general procedure, the reaction of 3,3-dimethyl-1-butyne, CO_2, and iodoebutane provided the desired product with an average yield of 44%. ^1H NMR (400 MHz, $CDCl_3$): δ 4.14 (t, 2H, J = 6.8 Hz), 1.70–1.58 (m, 2H), 1.44–1.34 (m, 2H), 1.28 (s, 9H), 0.93 (t, 4H, J = 7.4 Hz). ^{13}C NMR (101 MHz, $CDCl_3$): δ 154.44, 96.54, 71.96, 65.76, 37.44, 30.60, 30.12, 27.65, 22.55, 19.17, 13.79. GC/MS: retention time = 12.5 min, M/Z = 181.

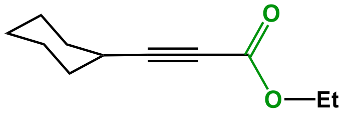

Ethyl 3-cyclohexylpropiolate

Following the general procedure, the reaction of 1-ethynylcyclohexane, CO_2, and iodoethane provided the desired product with an average yield of 55%. ^1H NMR (400 MHz, $CDCl_3$): δ 4.20 (q, 2H, J = 7.1 Hz), 2.50 (tt, 1H, J = 9.3, 3.8 Hz), 1.88–1.77 (m, 2H), 1.70 (qd, 2H, J = 7.3, 2.6 Hz), 1.57–1.50 (m, 2H), 1.50– .42 (m, 1H), 1.29 (t, 6H, J = 7.1 Hz). ^{13}C NMR (101 MHz, $CDCl_3$): δ 154.18, 92.94, 73.20, 61.85, 31.56, 28.98, 25.70, 24.76, 14.17. GC/MS: retention time = 15.4 min, M/Z = 180.

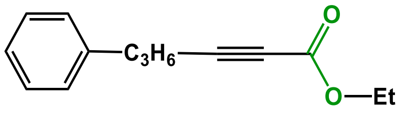

Ethyl 6-phenylhex-2-ynoate

Following the general procedure, the reaction of 5-phenyl-1-pentyne, CO_2, and iodoethane provided the desired product with an average yield of 89%. ^1H NMR (400 MHz, $CDCl_3$): δ 7.34–7.14 (m, 5H), 4.23 (q, 2H, J = 7.1 Hz), 2.74 (t, 2H, J = 7.5 Hz), 2.34 (t, 2H, J = 7.1 Hz), 1.91 (qunitet,

2H, J = 7.3 Hz), 1.32 (t, 3H, J = 7.1 Hz). ^{13}C NMR (101 MHz, $CDCl_3$): δ 153.95, 140.96, 128.62, 128.58, 126.25, 88.93, 73.75, 61.94, 34.78, 29.17, 18.13, 14.17. GC/MS: retention time = 19.9 min, M/Z = 216.

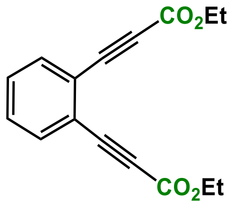

Diethyl 3,3'-(1,2-phenylene)dipropiolate

Following the general procedure, the reaction of 1,2-diethynylbenzene, CO_2, and iodoethane provided the desired product with an average yield of 99%. ^{1}H NMR (400 MHz, $CDCl_3$): δ 7.62 (dd, 1H, J = 5.8, 3.3 Hz), 7.44 (dd, 1H, J = 5.8, 3.3 Hz), 4.31 (q, 2H, J = 7.2 Hz), 1.36 (t, 3H, J = 7.1 Hz). ^{13}C NMR (101 MHz, $CDCl_3$): δ 153.80, 133.57, 130.38, 123.78, 84.89, 82.91, 62.42, 14.20. GC/MS: retention time = 23.9 min, M/Z = 270.

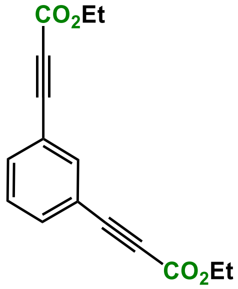

Diethyl 3,3'-(1,3-phenylene)dipropiolate

Following the general procedure, the reaction of 1,3-diethynylbenzene, CO_2, and iodoethane provided the desired product with an average yield of 97%. ^{1}H NMR (400 MHz, $CDCl_3$): δ 7.78 (t, 1H, J = 1.7 Hz), 7.63 (dd, 2H, J = 7.8, 1.7 Hz), 7.40 (t, 1H, J = 7.8 Hz), 4.30 (q, 4H, J = 7.1 Hz), 1.35 (t, 6H, J = 7.1 Hz). ^{13}C NMR (101 MHz, $CDCl_3$): δ 153.80, 136.97, 134.71, 129.15, 120.71, 84.03, 81.68, 62.44, 14.19. GC/MS: retention time = 24.9 min, M/Z = 270.

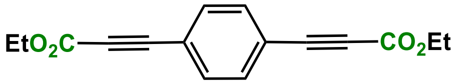

Diethyl 3,3'-(1,4-phenylene)dipropiolate

Following the general procedure, the reaction of 1,4-diethynylbenzene, CO_2, and iodoethane provided the desired product with an average yield of 97%. ^{1}H NMR (400 MHz, $CDCl_3$): δ 7.58 (s,

2H), 4.30 (q, 2H, *J* = 7.1 Hz), 1.36 (t, 3H, *J* = 7.1 Hz). ^{13}C NMR (101 MHz, $CDCl_3$): δ 153.81, 133.01, 121.94, 84.52, 83.01, 62.46, 14.19. GC/MS: retention time = 24.8 min, M/Z = 270.

Single-crystal X-ray crystallography. A summary of crystal data and refinement details for all compounds is given in Table S1.

General Data Collection

Data were collected on a Bruker D8 VENTURE DUO diffractometer equipped with a IµS 3.0 microfocus source operated at 75 W (50kV, 1.5 mA) to generate Mo Kα radiation (λ = 0.71073 Å) and a PHOTON III detector. Crystals were transferred from the vial and placed on a glass slide in type NVH immersion oil by Cargille. A Zeiss Stemi 305 microscope was used to identify a suitable specimen for X-ray diffraction from a representative sample of the material. The crystal and a small amount of the oil were collected on a MiTeGen 100 micron MicroLoop and transferred to the instrument where it was placed under a cold nitrogen stream (Oxford 800 series) maintained at 100K throughout the duration of the experiment. The sample was optically centered using a video camera to ensure no translations were observed as the crystal was rotated through all positions. A unit cell collection was then carried out. After it was determined that the unit cell was not present in the CCDC database, a data collection strategy was calculated by *APEX4*.[3]

Refinement Details

(a) *Refined structure of [Cu-**L3**]PF_6.* After data collection, the unit cell was re-determined using a subset of the full data collection. Intensity data were corrected for Lorentz, polarization, and background effects using the *APEX4*[3]. A semi-empirical correction for adsorption was applied using *SADABS*[3]. The program *SHELXT*[4] was used for the initial structure solution, and *SHELXL*[5] was used for the refinement of the structure. Both programs were utilized within the OLEX2 software[6]. Hydrogen atoms bound to carbon atoms were located in the difference Fourier map and were geometrically constrained using the appropriate AFIX commands.

(b) *Refined structure of [Cu-**L4**]$PF_6.CH_3CN$.* After data collection, the unit cell was re-determined using a subset of the full data collection. Intensity data were corrected for Lorentz, polarization, and background effects using the *APEX4*[3]. A numerical absorption correction was applied based on a Gaussian integration over a multifaceted crystal, followed by a semi-empirical correction for adsorption using *SADABS*[3]. The program *SHELXT*[4] was used for the initial structure solution, and *SHELXL*[5] for the structure refinement. Both programs were utilized within the OLEX2

software[6]. A methyl moiety was positionally disordered and modeled as split sites (C33). To help maintain reasonable ADP and bond length values for the disordered sites, SIMU, RIGU, and free variable DFIX restraints were applied. Hydrogen atoms bound to carbon atoms were located in the difference Fourier map and were geometrically constrained using the appropriate AFIX commands.

(c) *Refined structure of [Cu-**L3***]*. After data collection, the unit cell was re-determined using a subset of the full data collection. Intensity data were corrected for Lorentz, polarization, and background effects using the *APEX6*[3]. A numerical absorption correction was applied based on a Gaussian integration over a multifaceted crystal, followed by a semi-empirical correction for adsorption using *SADABS*[3]. The program *SHELXT*[4] was used for the initial structure solution, and *SHELXL*[5] for structure refinement. Both programs were utilized within the OLEX2 software[6]. In this structure, there were multiple disordered sites. Due to this, a large number of atom sites were modeled as positionally disordered over 2 sites. To help maintain reasonable ADP values, SIMU 0.01 and RIGU 0.001 were applied to all disordered sites. Hydrogen atoms bound to carbon atoms were located in the difference Fourier map and were geometrically constrained using the appropriate AFIX commands.

(d) Refined structure of [Cu-L1].* After data collection, the unit cell was re-determined using a subset of the full data collection. Intensity data were corrected for Lorentz, polarization, and background effects using the *APEX6*[3]. A semi-empirical correction for adsorption was applied using *SADABS*[3]. The program *SHELXT*[4] was used for the initial structure solution and *SHELXL*[5] was used for refinement of the structure. Both programs were utilized within the OLEX2 software[6]. In this structure, all three crystallographically unique metal-ligand complexes were positionally disordered and modeled as split sites. Each molecular position was allowed to refine its split occupancies independently. To help maintain reasonable bond length and ADP values, SADI, SIMU, and RIGU restraints were used for every atom in the model. Hydrogen atoms bound to carbon atoms were located in the difference Fourier map and were geometrically constrained using the appropriate AFIX commands. The Z’ value for this structure is 3.

(e) Refined structure of [L3Cu(PA)]$_2$.2CH$_3$CN. After data collection, the unit cell was re-determined using a subset of the full data collection. Intensity data were corrected for Lorentz, polarization, and background effects using the APEX6[3]. A numerical absorption correction was applied based on a Gaussian integration over a multifaceted crystal and followed by a semi-empirical correction for adsorption applied using SADABS[3]. The program SHELXT[4] was used for the initial structure solution and SHELXL[5] was used for refinement of the structure. Both programs were utilized within the OLEX2 software[6]. In this structure, the interstitial acetonitrile molecule was positionally disordered and modeled over two sites. Hydrogen atoms bound to carbon atoms were located in the difference Fourier map and were geometrically constrained using the appropriate AFIX commands.

CCDC numbers 2415975, 2415955, 2488390, 2494324, and 2514575 contain the supplementary crystallographic data for this paper. These data can be obtained free of charge from The Cambridge Crystallographic Data Centre via www.ccdc.cam.ac.uk/data_request/cif

Table S1. Summary of X-ray crystallographic data collection parameters for the new Cu-CNC complexes

complex	[Cu-**L3**]PF_6	[Cu-**L4**]$PF_6.CH_3CN$	[Cu-**L3***]	[Cu-**L1***]	[**L3**-Cu(PA)]$_2$.2CH_3CN
formula	$C_{31}H_{33}CuN_5F_6P$	$C_{39}H_{48}CuF_6N_6P$	$C_{31}H_{32}CuN_5$	$C_{19}H_{24}CuN_5$	$C_{82}H_{82}Cu_2N_{12}$
Mr (g mol^{-1})	684.13	809.34	538.15	385.97	1362.67
T (K)	100	100	100	100	100
λ (Å)	0.71073	0.71073	0.71073	0.71073	0.71073
crystal system	triclinic	monoclinic	triclinic	hexagonal	triclinic
space group	P -1	P 1 21/n 1	P -1	P 31	P -1
a (Å)	8.9525(5)	11.0022(4)	9.1454(8)	10.6182(2)	8.3559(4)
b (Å)	12.2544(8)	15.0039(5)	11.5360(11)	10.6182(2)	11.2732(6)
c (Å)	14.4861(7)	23.1511(8)	14.1953(15)	43.2847(14)	19.0233(8)
α (deg)	78.930(2)	90	69.605(3)	90	90.952(2)
β (deg)	83.833(2)	93.337(2)	74.381(3)	90	94.577(2)
γ (deg)	83.018(2)	90	80.865(3)	120	95.280(2)
V ($Å^3$)	1542.32(15)	3815.2(2)	1348.4(2)	4226.4(2)	1778.14(15)
Z	2	4	2	9	1
ρ_{calc} (g cm^{-3})	1.473	1.409	1.325	1.365	1.273
μ (mm^{-1})	0.827	0.681	0.839	1.174	0.652
F (000)	704.0	1688.0	564.0	1818.0	716.0
2ϑ max (deg)	30.539	28.282	27.877	26.430	27.102
h,k,l max	12,17,20	14,20,30	12,15,18	13,13,54	10,14,24
no. of unique reflns	9456	9463	6386	11553	7847
final R indices (I > 2σ)	0.0345	0.0300	0.0774	0.0527	0.0403
R (wF^2)^a	0.0845	0.0753	0.1892	0.1140	0.0985
Goodness-of-fit on F^2	1.031	1.023	1.313	1.067	1.024

$^a R(\omega F^2) = \{\Sigma[\omega(F_o^2 - F_c^2)^2]/\Sigma[\omega(F_o^2)^2]\}^{1/2}$; $\omega = 1/[\sigma^s(F_o^2) + (aP)^2 + bP]$, $P = [2F_c^2 + \max(F_o,0)]/3$.

Table S2. Selected bond lengths and angles for the Cu-CNC complexes

complex	bonds	distance (Å)	bonds	angle (°)
[Cu-**L3**]PF_6	Cu(1)–N(3)	2.221(1)	C(1)–Cu(1)–C(11)	173.97(6)
	Cu(1)–C(1)	1.891(1)	C(1)–Cu(1)–N(3)	94.06(6)
	Cu(1)–C(11)	1.894(1)	C(11)–Cu(1)–N(3)	91.97(6)
	C(4)-C(5)	1.510(2)	N(2)-C(4)-C(5)	115.0(1)
	C(6)-C(10)	1.512(2)	N(4)-C(10)-C(6)	118.2(1)
	C(2)–C(3)	1.353(3)		
	C(12)–C(13)	1.351(2)		
	C(6)-C(7)	1.393(2)		
	C(7)-C(8)	1.388(2)		
	C(8)-C(9)	1.389(2)		
	C(9)-C(5)	1.387(2)		
	N(3)-C(5)	1.348(2)		
	N(3)-C(6)	1.341(2)		
[Cu-**L4**]$PF_6.CH_3CN$	Cu(1)–N(3)	2.269(1)	C(1)–Cu(1)–C(11)	178.04(6)
	Cu(1)–C(1)	1.904(1)	C(1)–Cu(1)–N(3)	90.43(5)
	Cu(1)–C(11)	1.904(1)	C(11)–Cu(1)–N(3)	89.68(5)
	C(2)–C(3)	1.351(2)		
	C(12)–C(13)	1.352(2)		
[Cu-**L3***]	Cu(1)–N(3)	2.095(4)	C(1)–Cu(1)–C(11)	168.0(2)
	Cu(1)–C(1)	1.905(4)	C(1)–Cu(1)–N(3)	96.4(2)
	Cu(1)–C(11)	1.896(4)	C(11)–Cu(1)–N(3)	95.6(2)
	C(4)-C(5)	1.36(1)	N(2)-C(10)-C(9)	113(1)
	C(9)-C(10)	1.52(2)	N(4)-C(10)-C(6)	128(1)
	C(2)–C(3)	1.344(6)		
	C(12)–C(13)	1.350(9)		
	C(5)-C(6)	1.48(1)		
	C(6)-C(7)	1.31(2)		
	C(7)-C(8)	1.43(1)		
	C(8)-C(9)	1.42(1)		
	N(3)-C(5)	1.372(6)		
	N(3)-C(9)	1.364(6)		
[Cu-**L1***]	Cu(2)–N(8)	2.10(1)	C(20)–Cu(2)–C(30)	164.1(7)
	Cu(2)–C(20)	1.86(1)	C(20)–Cu(2)–N(8)	95.6(6)

	Cu(2)–C(30)	1.92(1)	C(30)–Cu(2)–N(8)	97.6(6)
	C(23)-C(24)	1.35(3)	N(7)-C(23)-C(24)	129(2)
	C(25)-C(29)	1.55(2)	N(9)-C(29)-C(25)	114(1)
	Cu(1)–N(3A)	2.12(1)	C(1A)–Cu(1)–C(11A)	170(1)
	Cu(1)–C(1A)	2.01(2)	C(1A)–Cu(1)–N(3A)	98.9(9)
	Cu(1)–C(11A)	1.82(2)	C(11A)–Cu(1)–N(3A)	95.6(8)
	C(5A)-C(4A)	1.43(2)	N(2A)-C(5A)-C(4A)	127(1)
	C(6A)-C(10A)	1.51(2)	N(4A)-C(10A)-C(6A)	111(1)
	Cu(3A)–N(13A)	2.128(7)	C(39A)–Cu(3A)–C(49A)	165.3(7)
	Cu(3A)–C(39A)	1.86(2)	C(39A)–Cu(3A)–N(13A)	96.0(6)
	Cu(3A)–C(49A)	1.93(2)	C(49A)–Cu(3A)–N(13A)	95.9(5)
	C(42A)-C(43A)	1.39(2)	N(12A)-C(42A)-C(43A)	129(2)
	C(44A)-C(48A)	1.46(2)	N(14A)-C(48A)-C(44A)	114(1)

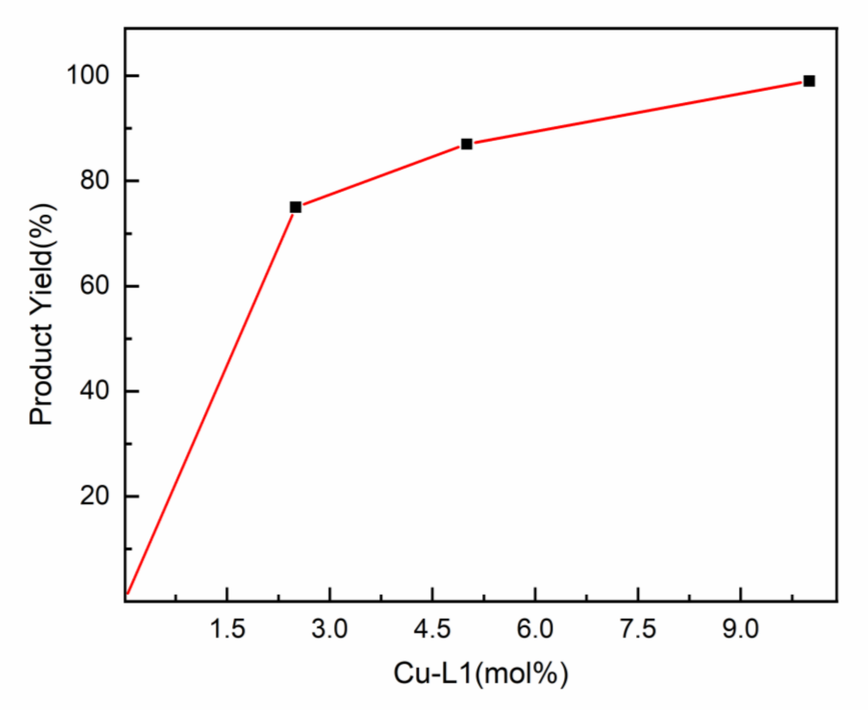

Figure S1. The isolated yields of ethyl 3-phenylpropiolate from the direct carboxylation of phenylacetylene in CH_3CN after 12 hours at room temperature and 12 hours at 80 °C. Reaction conditions: phenylacetylene (1.37 mmol), Cs_2CO_3 (2.74 mmol), iodoethane (4.00 mmol), Cu-**L1**, solvent (5 mL), CO_2 (1 atm).

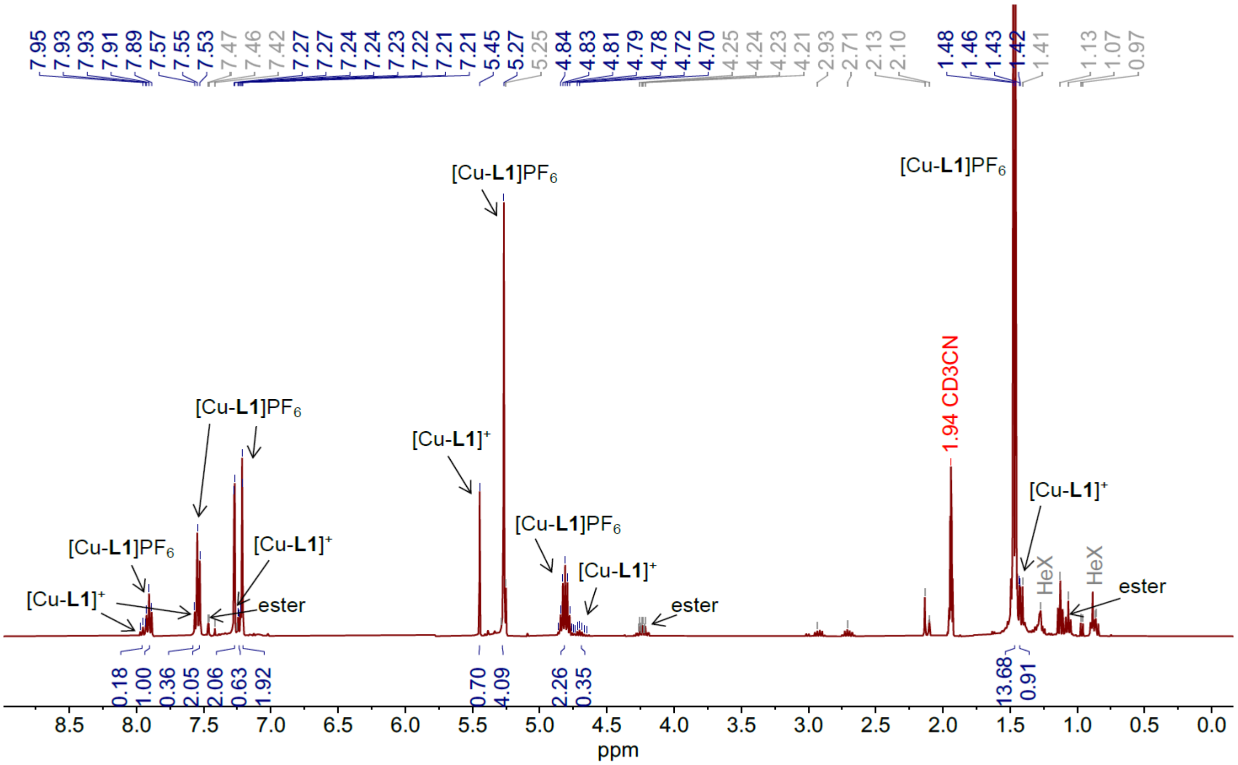

Figure S2. ^{1}H NMR spectrum of the copper catalyst fully recovered from the catalytic reaction after trituration and recrystalization, in CD_3CN (400 MHz).

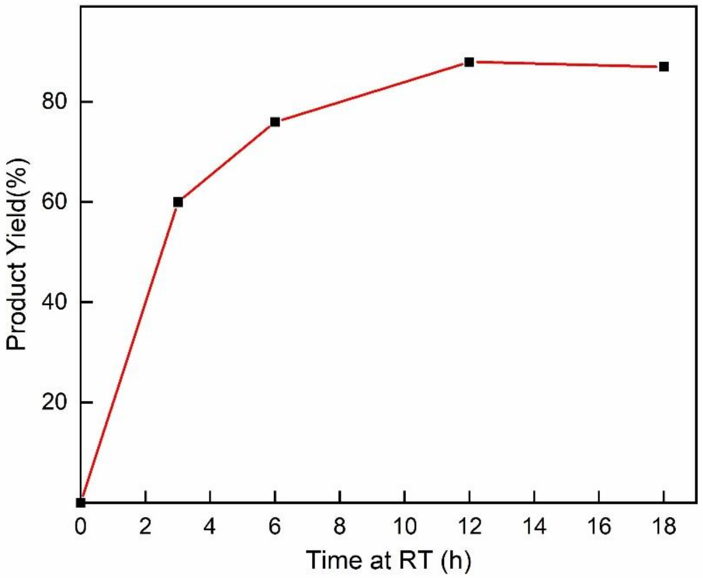

Figure S3. The isolated yields of ethyl 3-phenylpropiolate from the direct carboxylation-esterification of phenylacetylene with CO_2 catalyzed by [Cu-**L1**]PF_6 after 3, 6, and 12 hours of stirring at room temperature, followed by 12 h stirring at 80 °C. Reaction conditions: [Cu] (10 mol%), terminal alkyne (1.0 equiv.), Cs_2CO_3 (2.0 equiv.), EtI (2.9 equiv.), CO_2 (1 atm), CH_3CN (5mL).

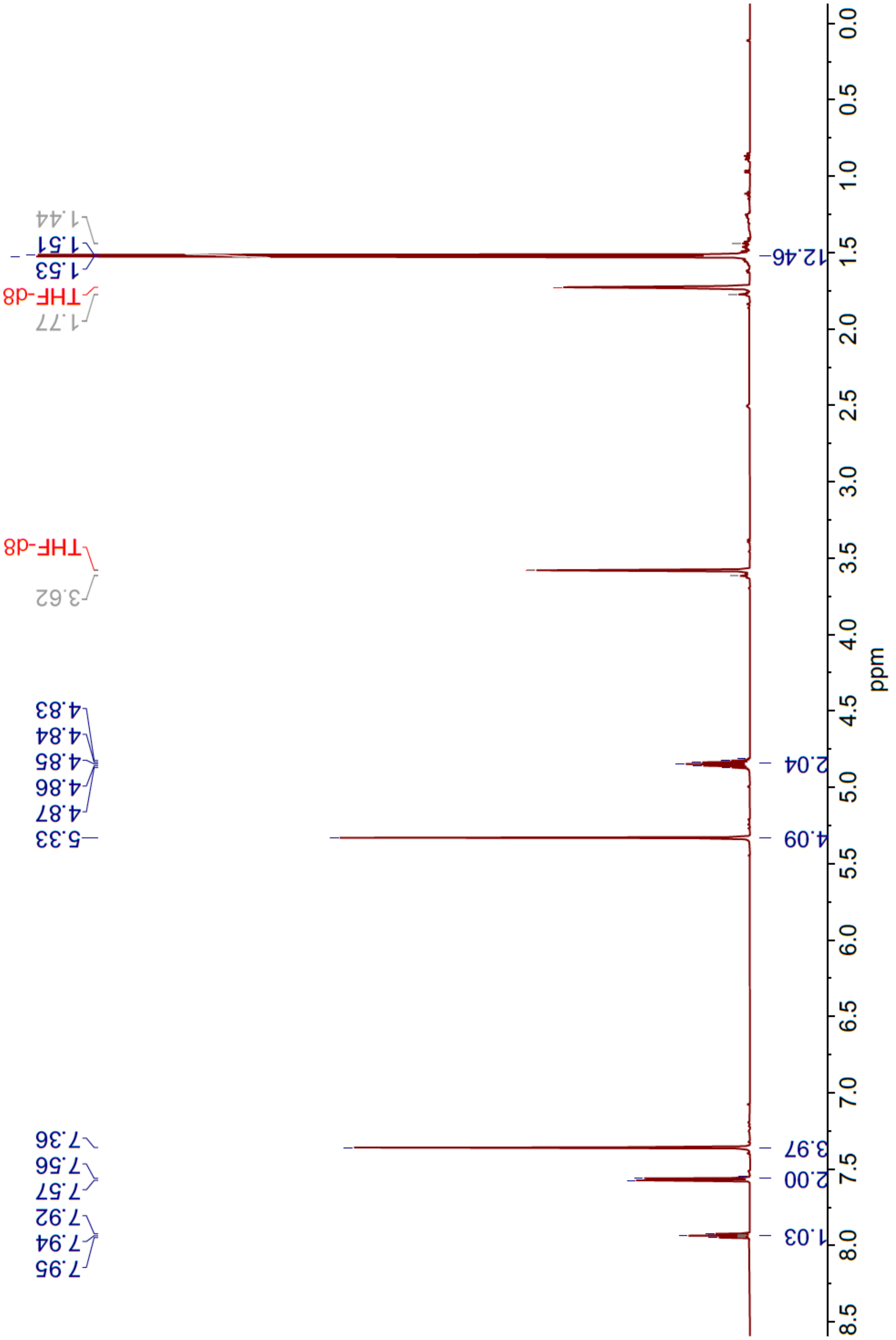

Figure S4. ^{1}H NMR spectrum for [Cu(I^{i}Pr$^{C\wedge N\wedge C}$)]BArF, [Cu-**L1**]BArF, in THF-d_8 at room temperature (400 MHz).

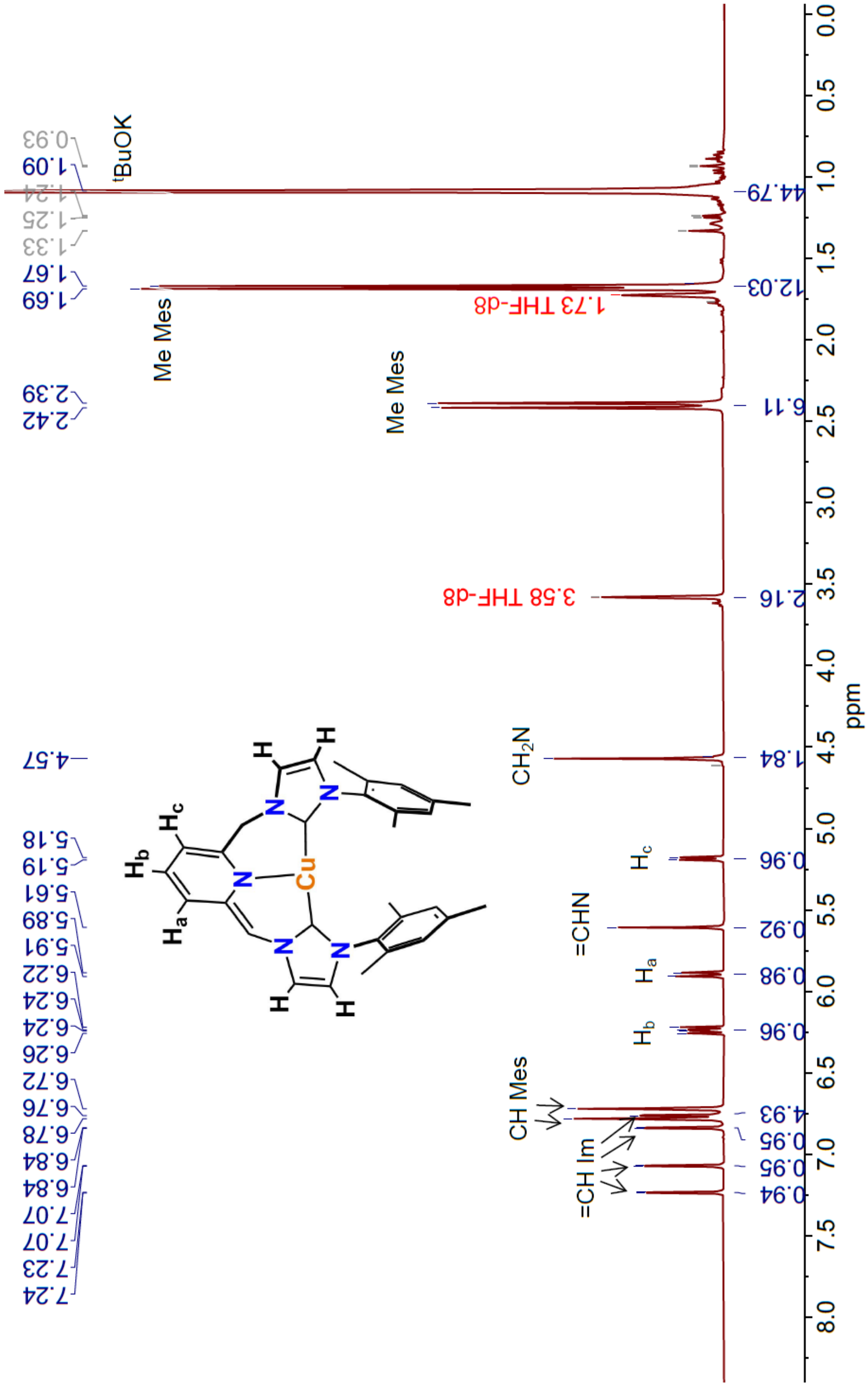

Figure S5. ^{1}H NMR spectrum for [Cu(IMes$^{C\wedge N\wedge C}$)*] (Cu-**L3***) in THF-d_8 at room temperature (600 MHz).

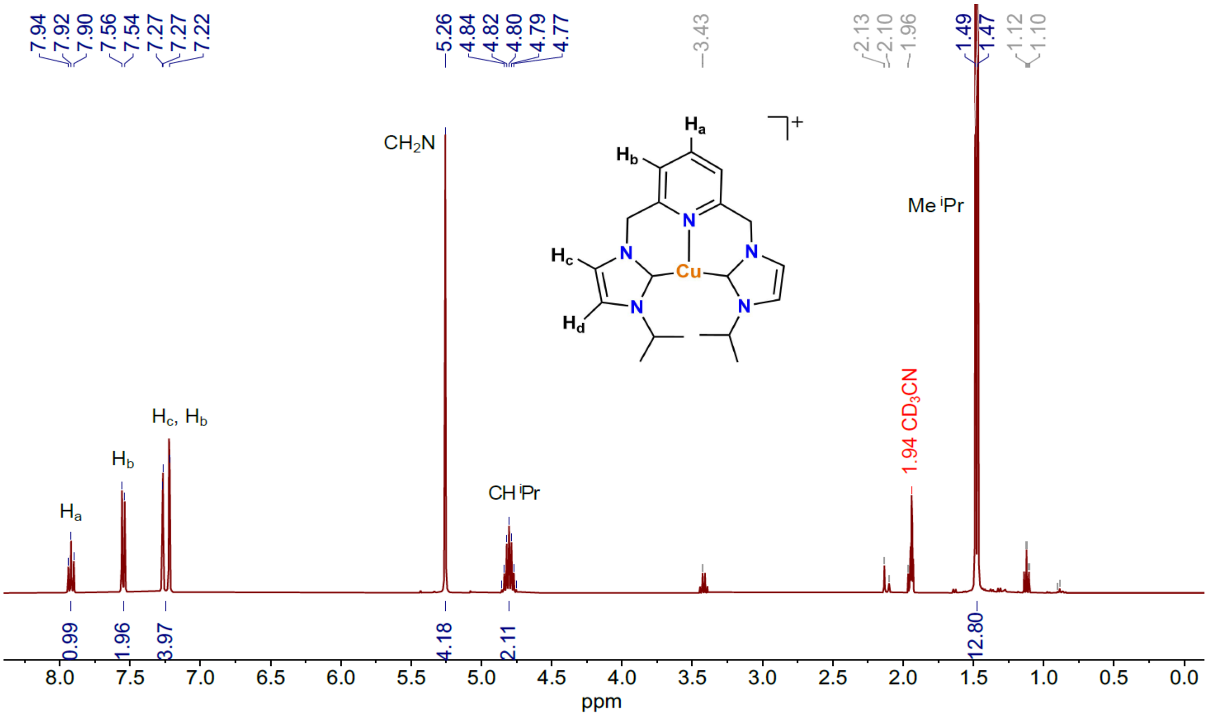

Figure S6. ^{1}H NMR spectrum for [Cu(I^{i}Pr$^{C\wedge N\wedge C}$)]PF_6, [Cu-**L1**]PF_6, in CD_3CN at RT (400 MHz).

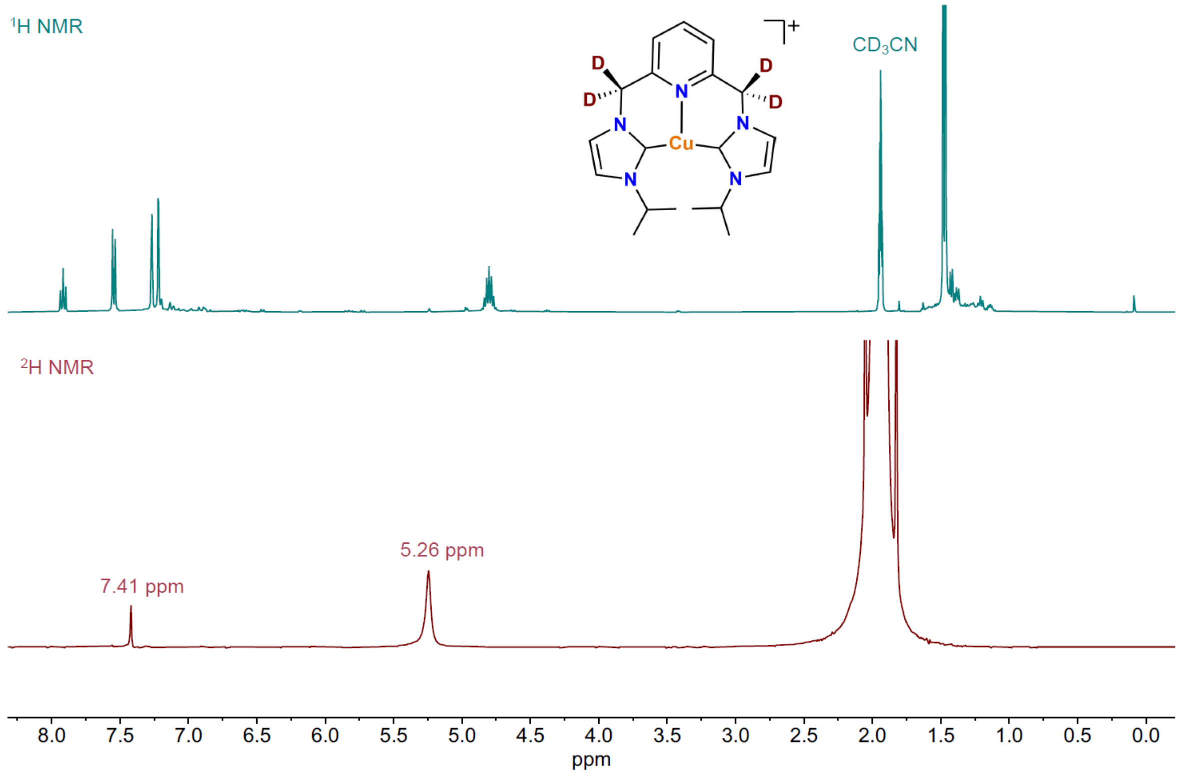

Figure S7. ^{1}H NMR (top) and ^{2}H NMR (bottom) spectra for the reaction of [Cu(I^{i}Pr$^{C\wedge N\wedge C}$)]PF_6, [Cu-**L1**]$^+$ with Cs_2CO_3 in a 1:5 molar ratio in CD_3CN after 24 hours of stirring at room temperature, yielding the original complex with deuterated methylene linkers, [Cu-**L1^D**]$^+$.

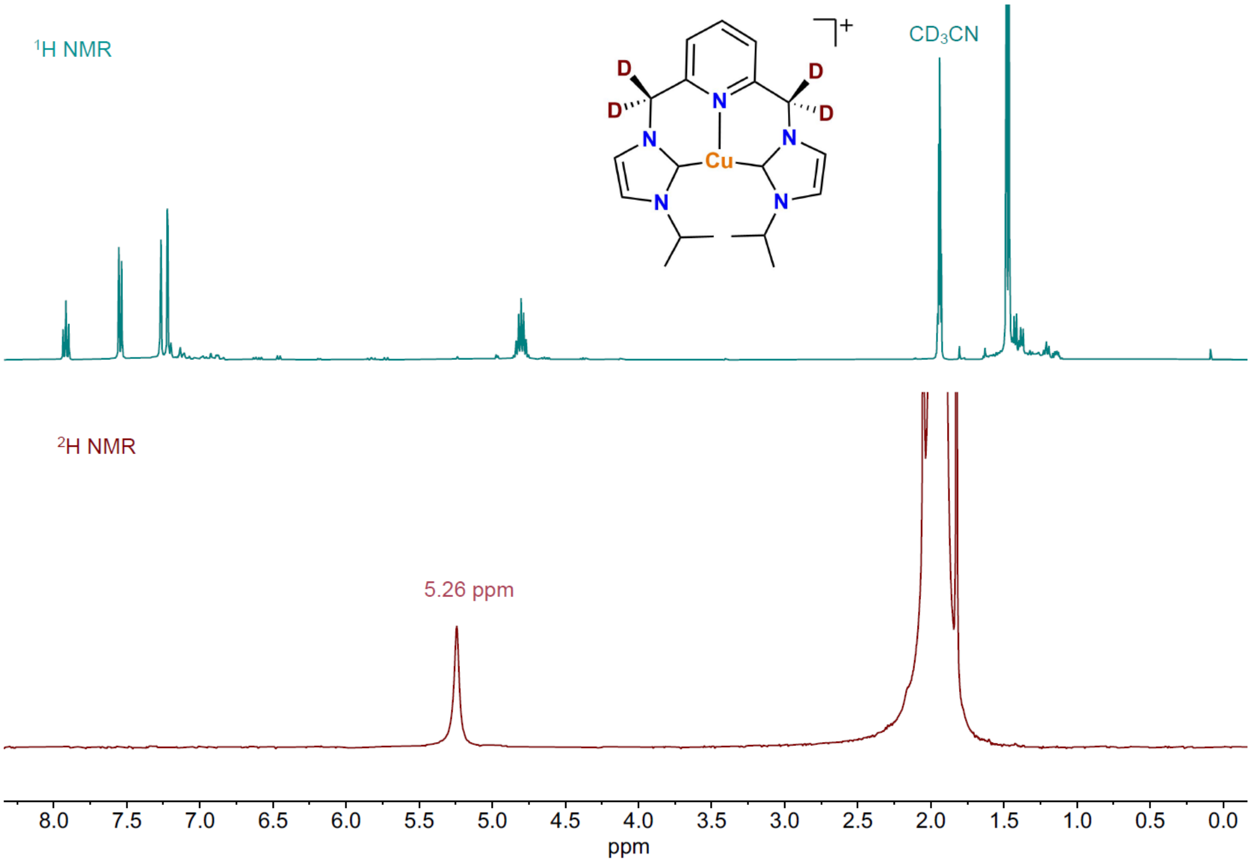

Figure S8. ^{1}H NMR (top) and ^{2}H NMR (bottom) spectra for the reaction of [Cu(I^{i}Pr$^{C\text{^}N\text{^}C}$)]PF$_6$, [Cu-**L1**]$^+$ with Cs_2CO_3 in a 1:1 molar ratio in CD_3CN after 24 hours of stirring at room temperature, yielding the original complex with deuterated methylene linkers, [Cu-^{D}**L1**]$^+$.

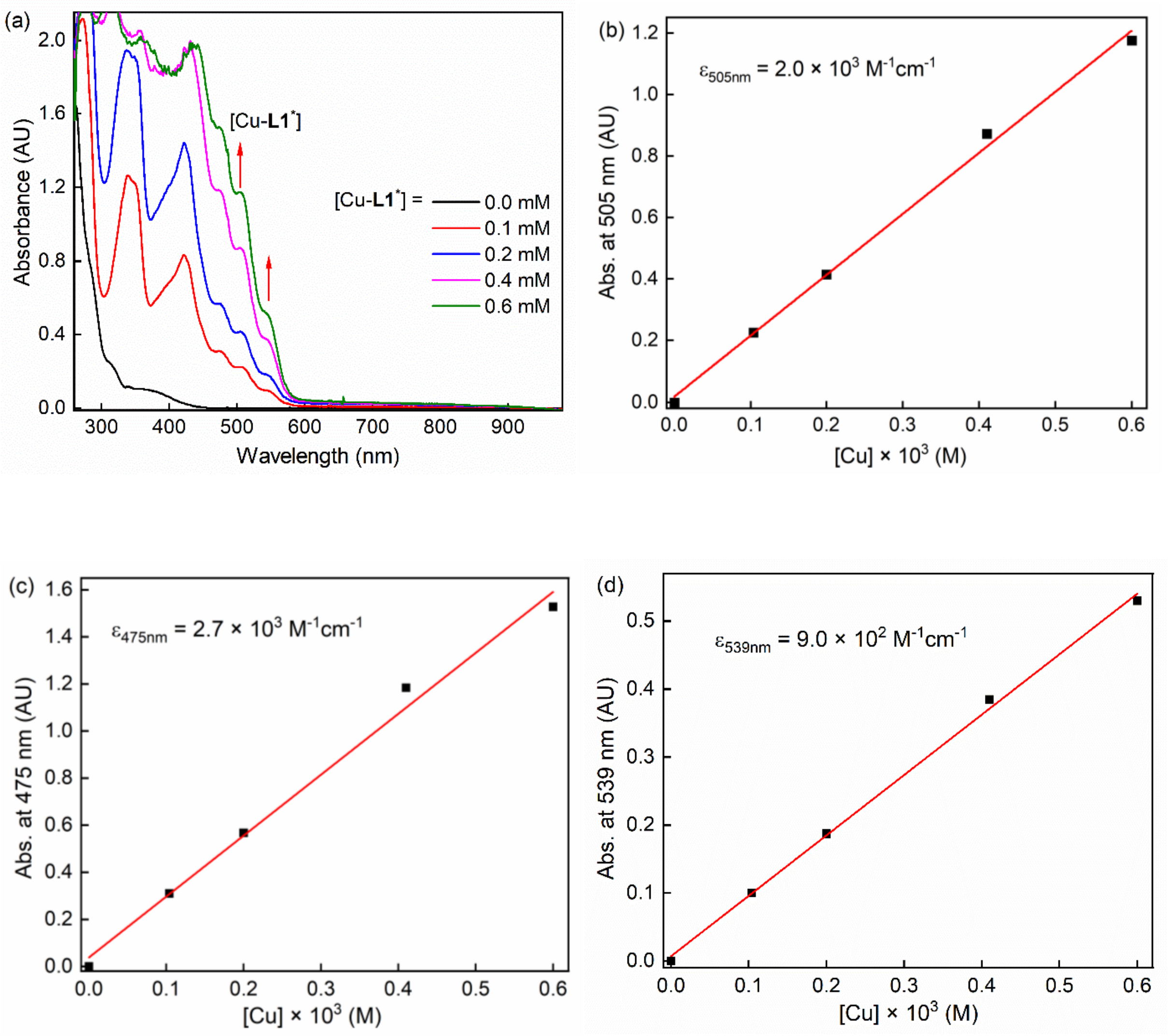

Figure S9. (a) Spectral titration for the formation of the dearomatized complex [Cu-**L1***] upon addition of an excess amount of KO^{t}Bu into the solution of [Cu-**L1**]BArF in THF at 223 K. (b, c, d) Plots of the absorbance at 505 nm, 475 nm, and 539 nm due to [Cu-**L1***] generated from the reaction of [Cu-**L1**]$^+$ and five-fold excess amount of KO^{t}Bu vs. the concentration of [Cu-**L1**]$^+$ (0.0 – 0.6 mM) at 223 K to determine the extinction coefficient of [Cu-**L1***] in THF.

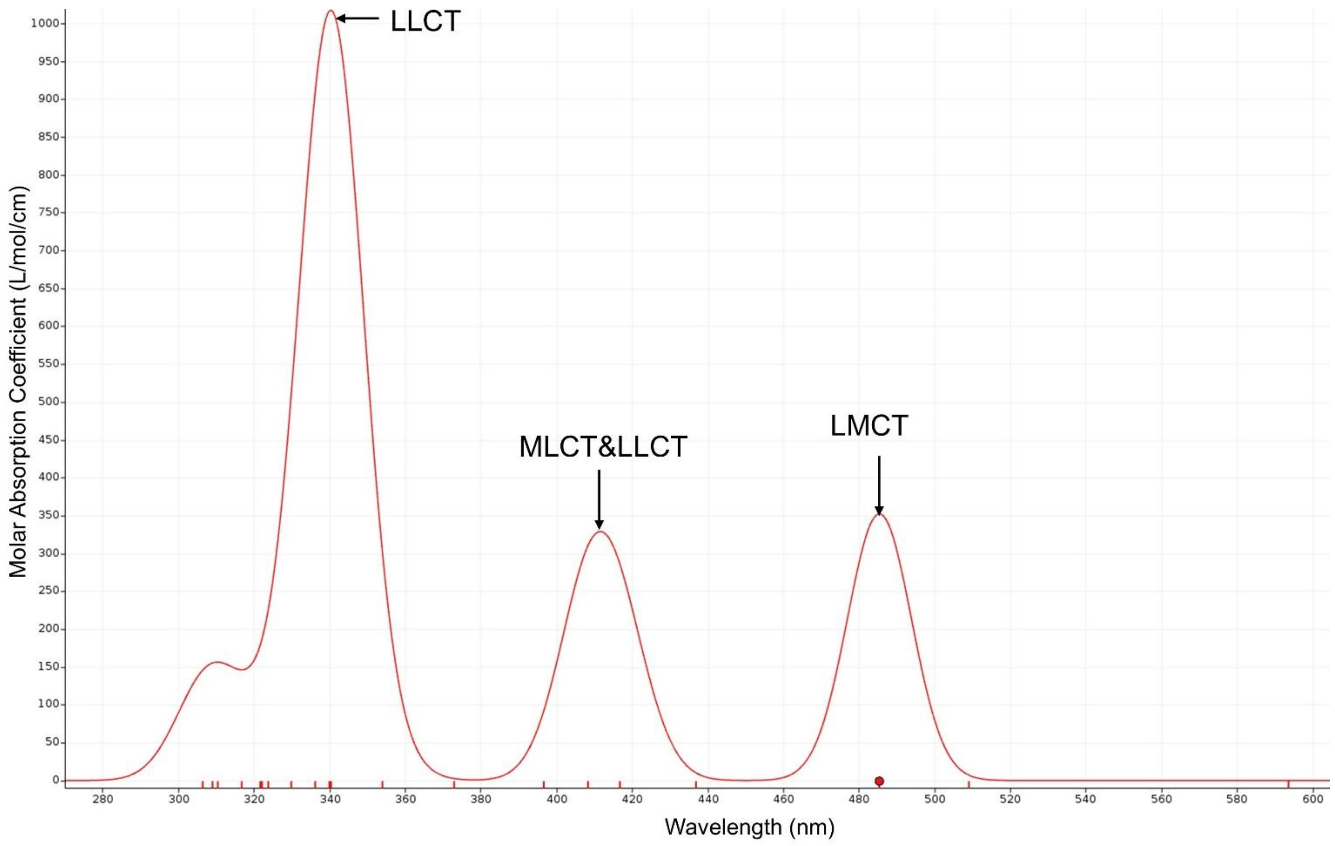

Figure S10. The UV-Vis spectrum of the dearomatized complex, [Cu-**L1***]. The peaks represent the Gaussian area of the calculated electronic transitions.

Note: The spectrum is calculated by the TD-DFT function of the AMS program (ADF 2023 package).[7, 8]

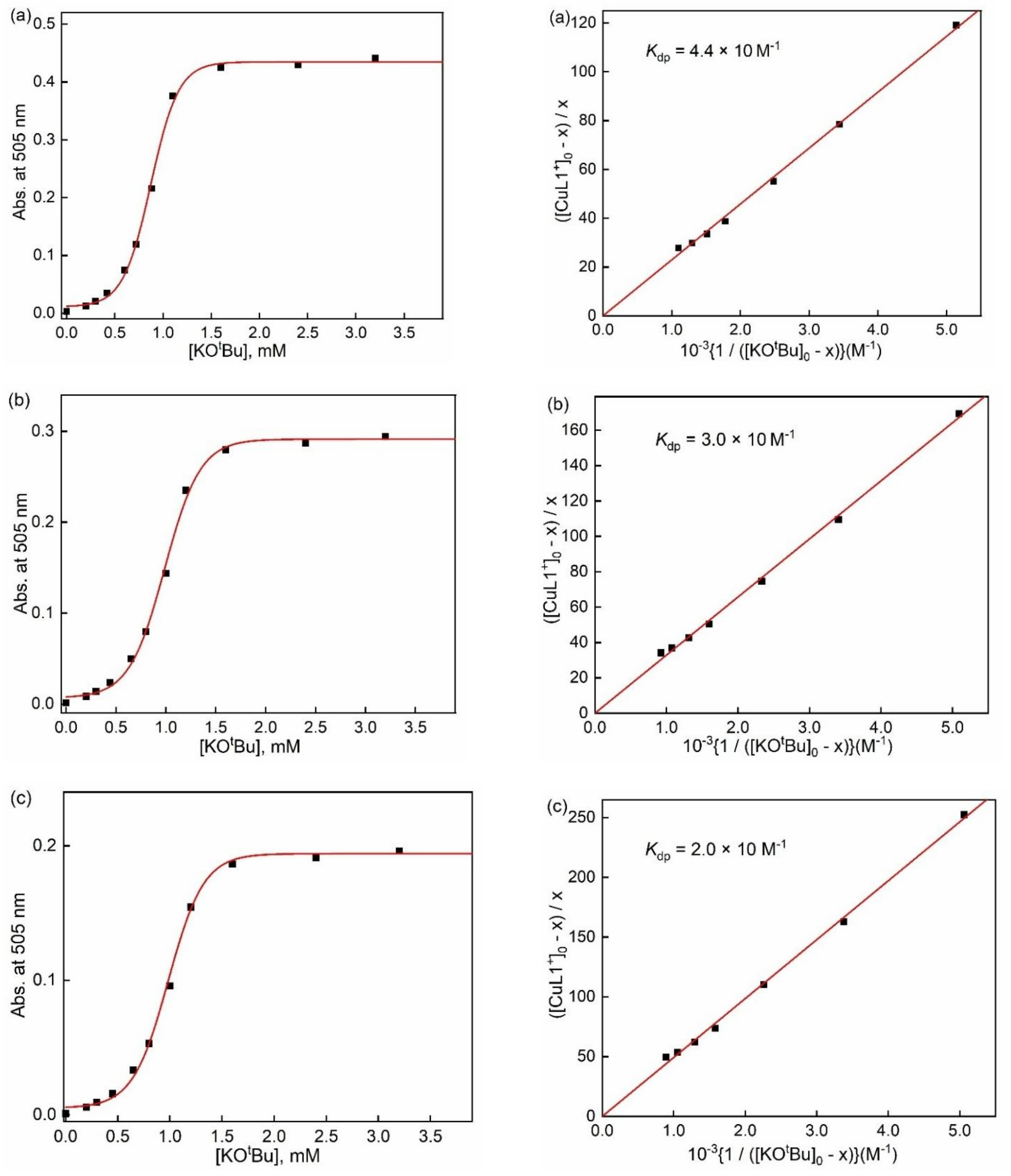

Figure S11. Absorbance changes of [Cu-**L1***] monitored at 505 nm upon addition of KO^tBu into the solution of [Cu-**L1***]BArF (0.50 mM) in THF at 213 K (a), 233 K (b), and 253 K (c) (left panels). Right panels show the plot of $([CuL1^+]_0 – x)/x$ vs $1/([KO^tBu]_0 – x)$ to determine the deprotonation constant of [Cu**L1**]BArF upon addition of KO^tBu (0.0 – 4.0 mM) into the solution of [Cu**L1**]BArF (0.50 mM) in THF.

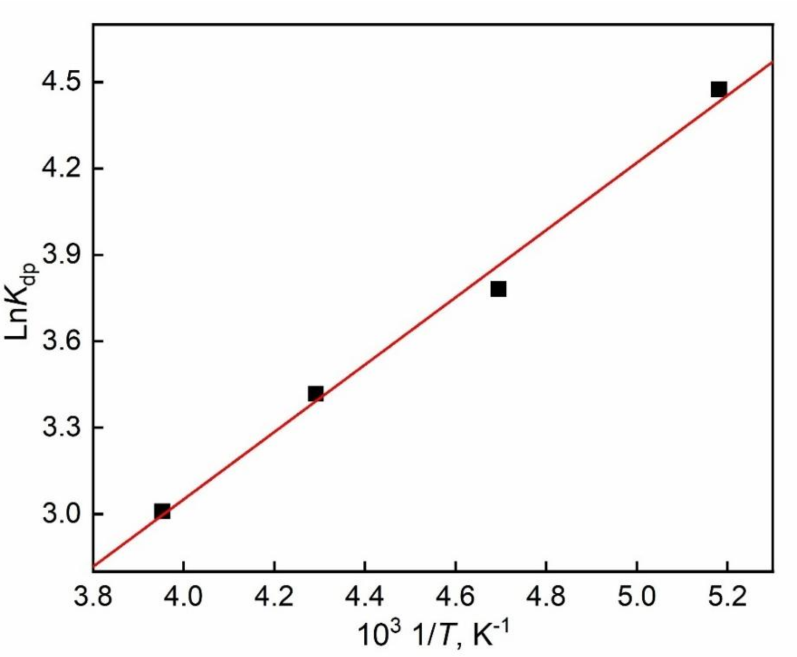

Figure S12. van’t Hoff plot to determine the enthalpy and entropy changes in the deprotonation of [Cu**L1**]BArF upon addition of KO^tBu (0.0 – 4.0 mM) into the solution of [Cu**L1**]BArF (0.50 mM) in THF.

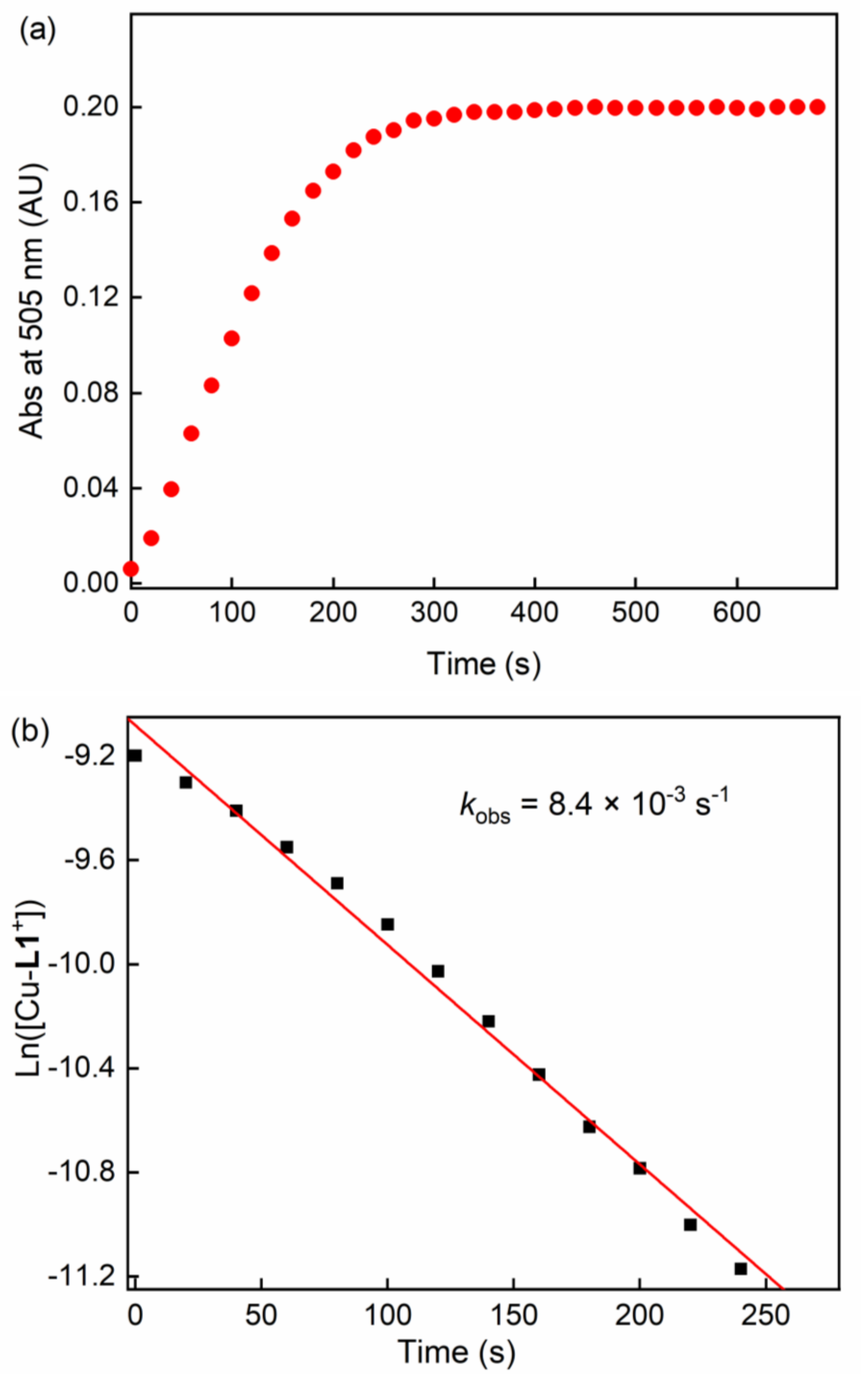

Figure S13. (a) Time profile of the absorbance at 505 nm due to [Cu-**L1***] and (b) the first-order plot of the formation of [Cu-**L1***] from the reaction of [Cu-**L1**]BArF (0.10 mM) with KO^{t}Bu (1.0 mM) in THF at 193 K.

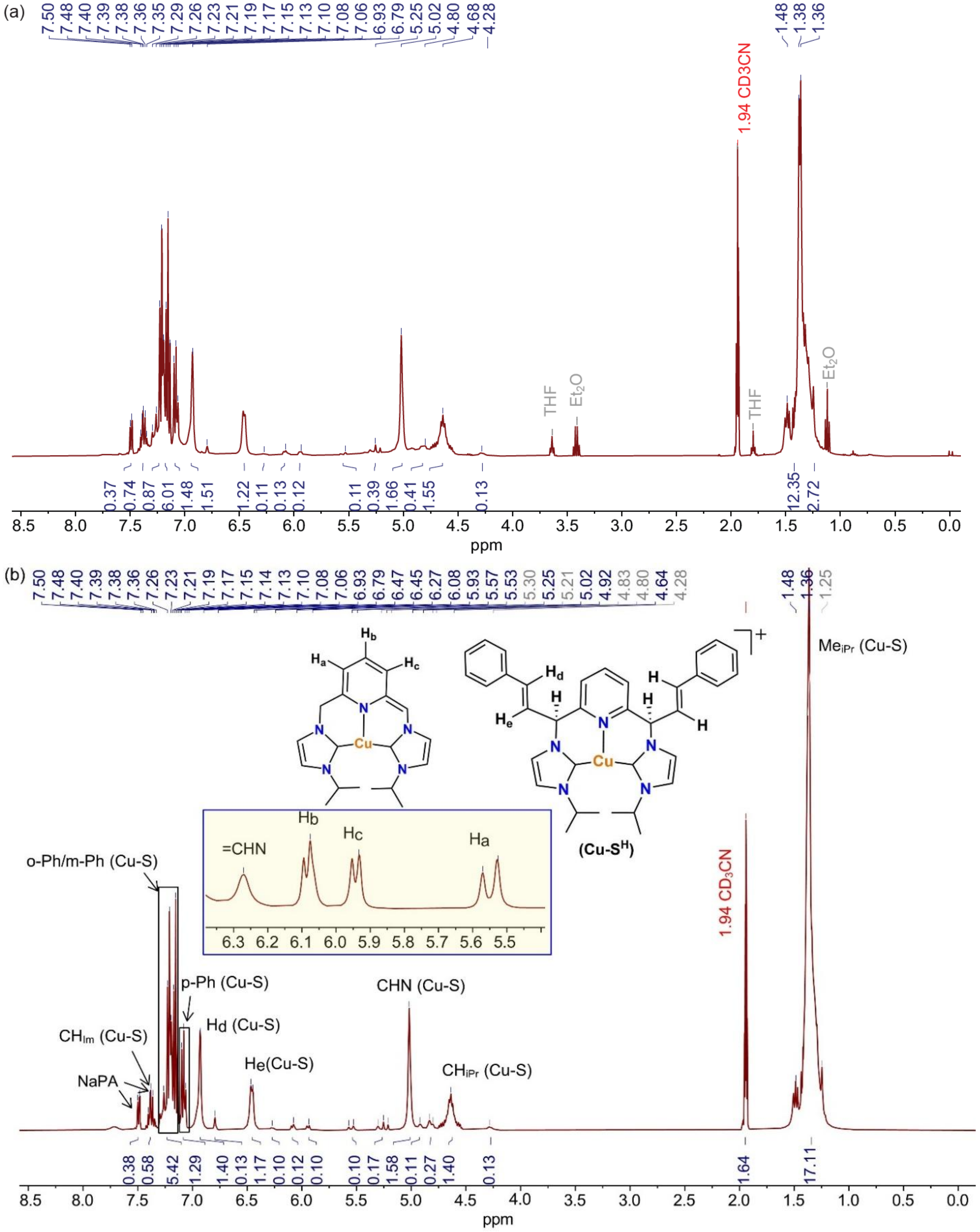

Figure S14. (a) ^{1}H NMR spectrum of the reaction of [Cu-**L1**]PF_6 and sodium phenylacetylide (1 equiv.) in CD_3CN upon mixing at RT (400 MHz). (b) Fitted spectrum after line fitting analysis and removal of solvent peaks for clarity.

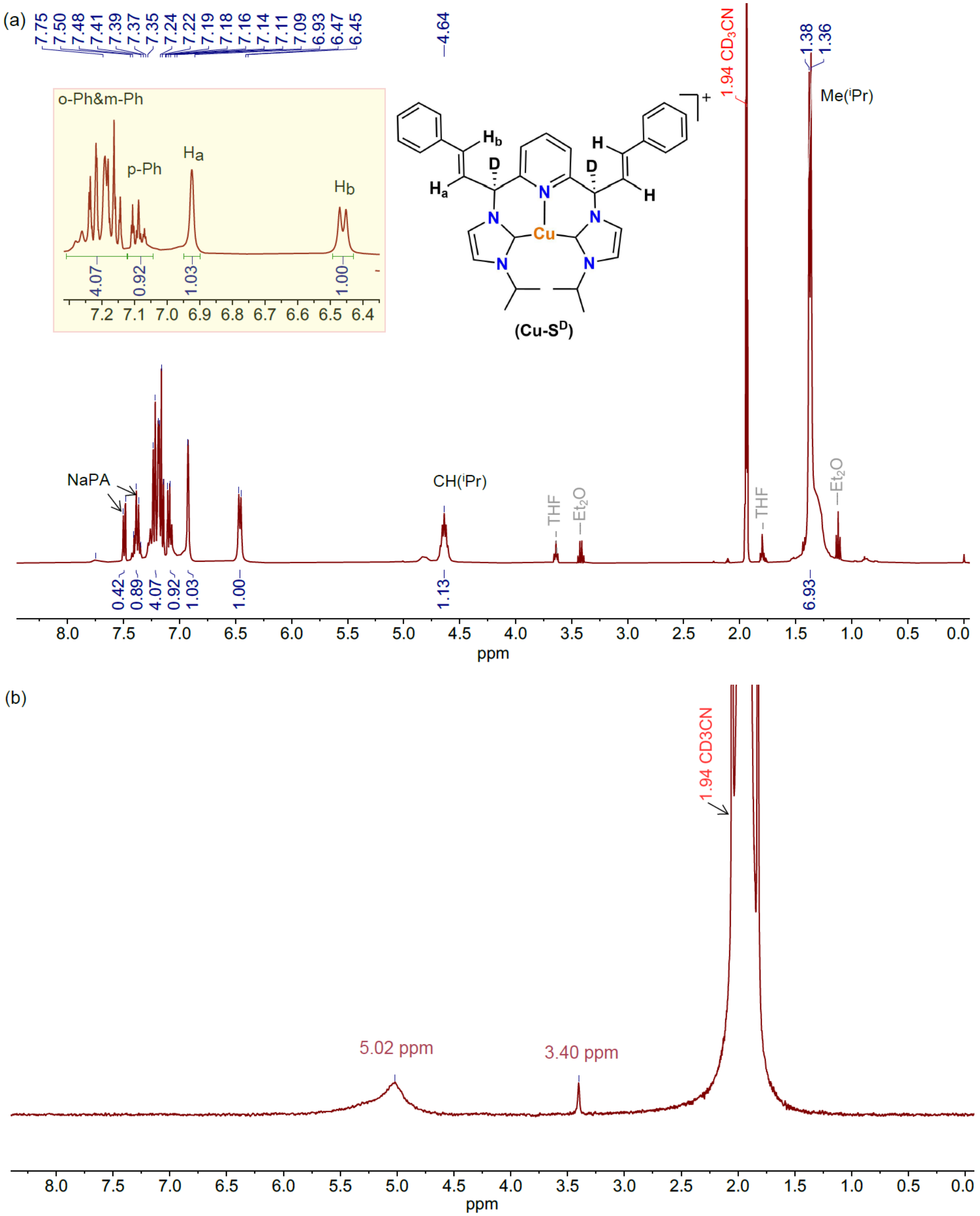

Figure S15. (a) ^{1}H NMR and (b) ^{2}H NMR spectra of the reaction of [Cu-**L1**]PF_6 and sodium phenylacetylide (1 equiv.) in CD_3CN after 24 hours of stirring at RT (400 MHz).

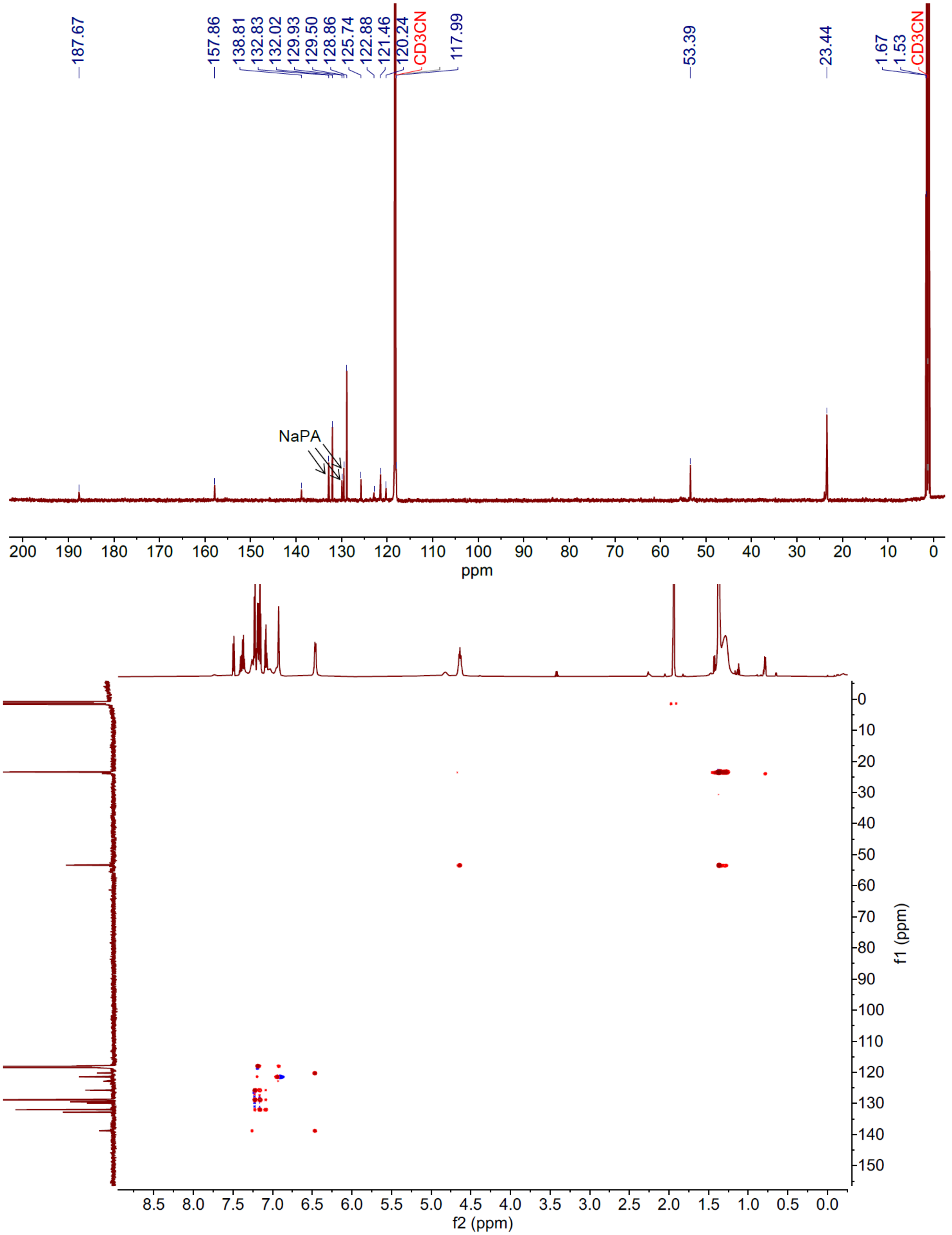

Figure S16. $^{13}C\{^{1}H\}$ NMR (top) and ^{1}H-^{13}C HSQC (bottom) spectra of **Cu-S** formed in situ from [Cu-**L1**]PF_6 and sodium phenylacetylide (1 equiv.) reaction in CD_3CN after 24 h of stirring at RT.

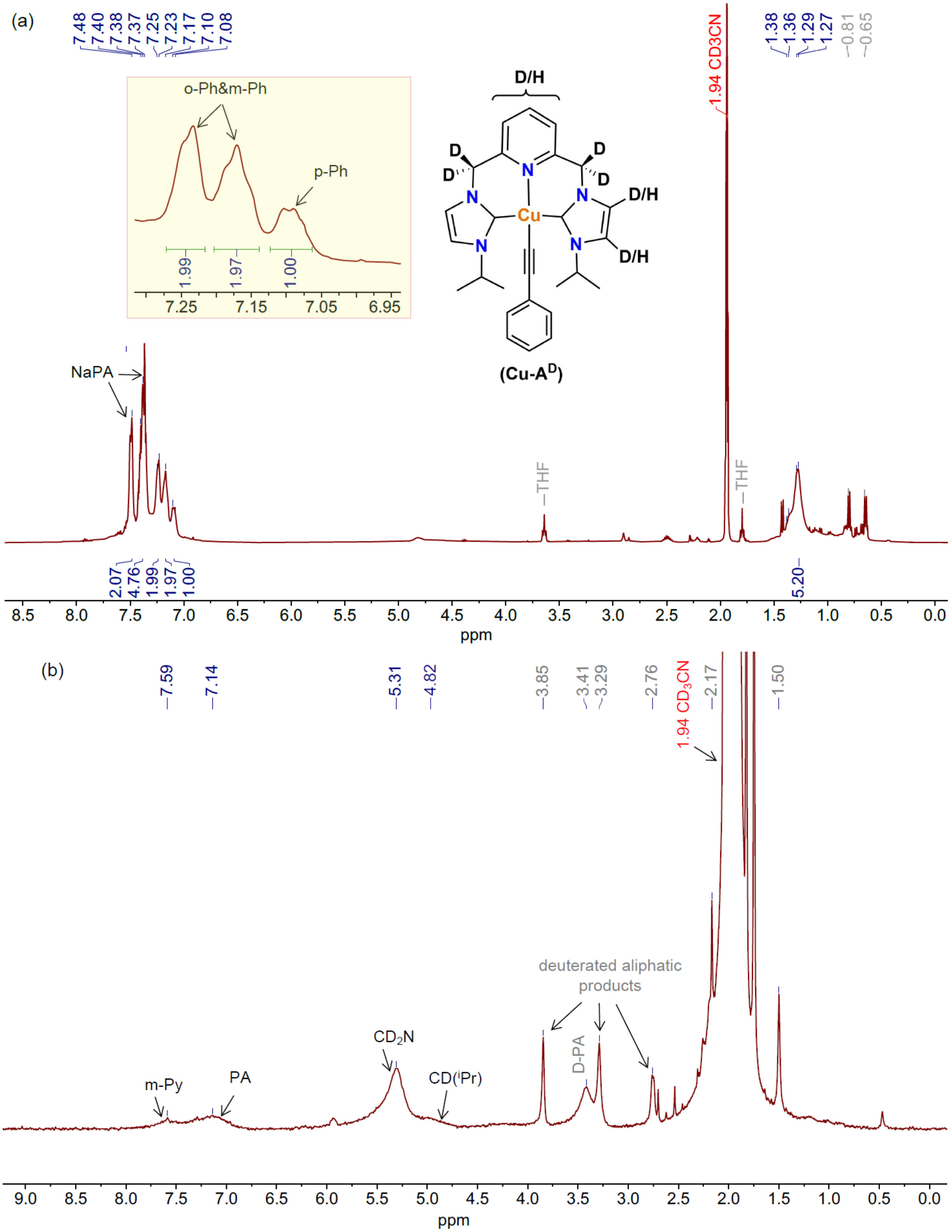

Figure S17. (a) ^{1}H NMR and (b) ^{2}H NMR spectra of the reaction of [Cu-**L1**]PF_6 and sodium phenylacetylide (5 equiv.) in CD_3CN after stirring at RT for 24 hours (400 MHz).

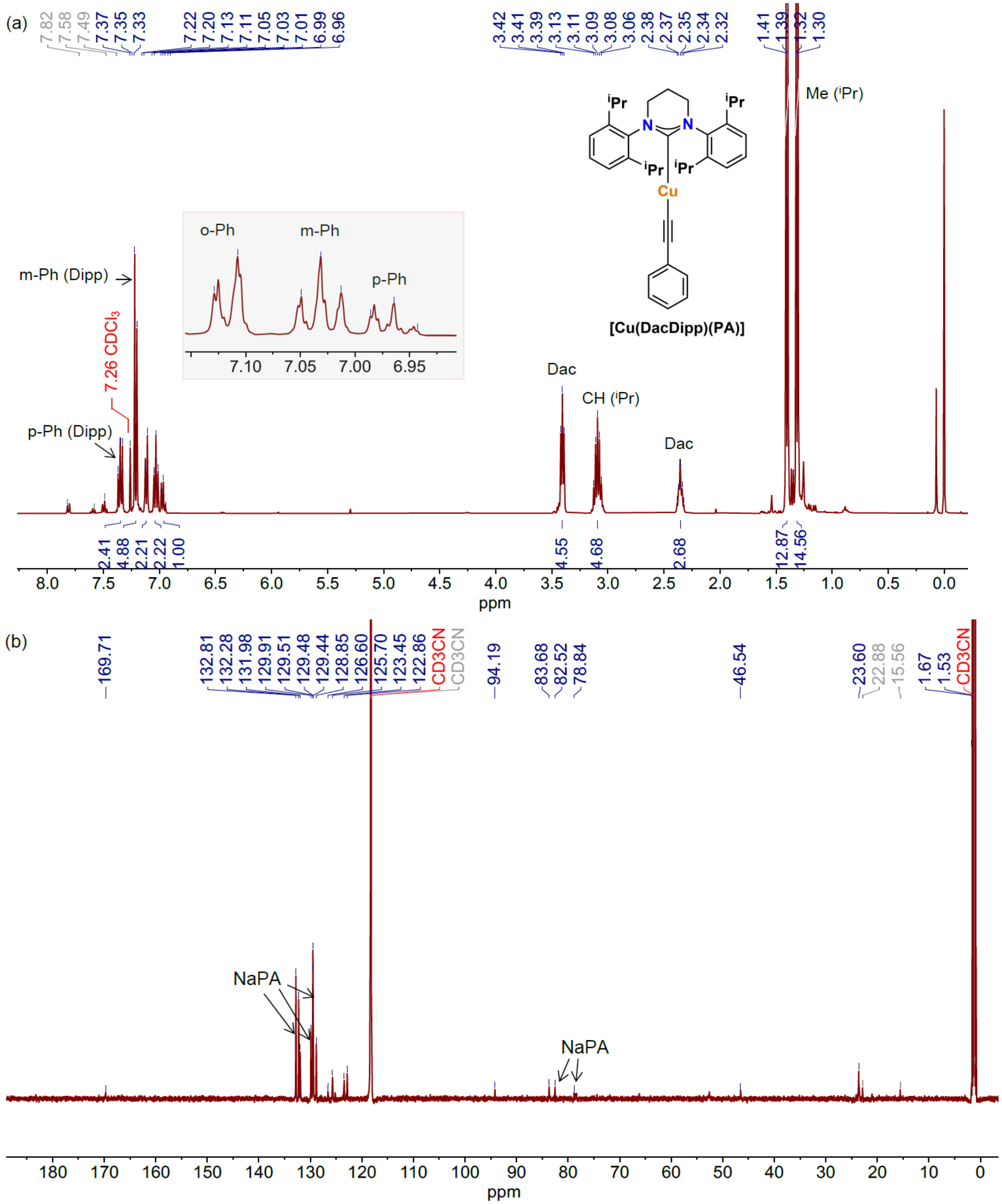

Figure S18. (a) ^{1}H NMR spectrum of [Cu(DacDipp)(PA)] complex in $CDCl_3$ displaying the resonance signals of the Cu-acetylide species. (b) $^{13}C\{^1H\}$ NMR spectrum of **Cu-A** formed in situ from [Cu-**L1**]PF_6 and sodium phenylacetylide (5 equiv.) reaction in CD_3CN after 24 h of stirring at RT.

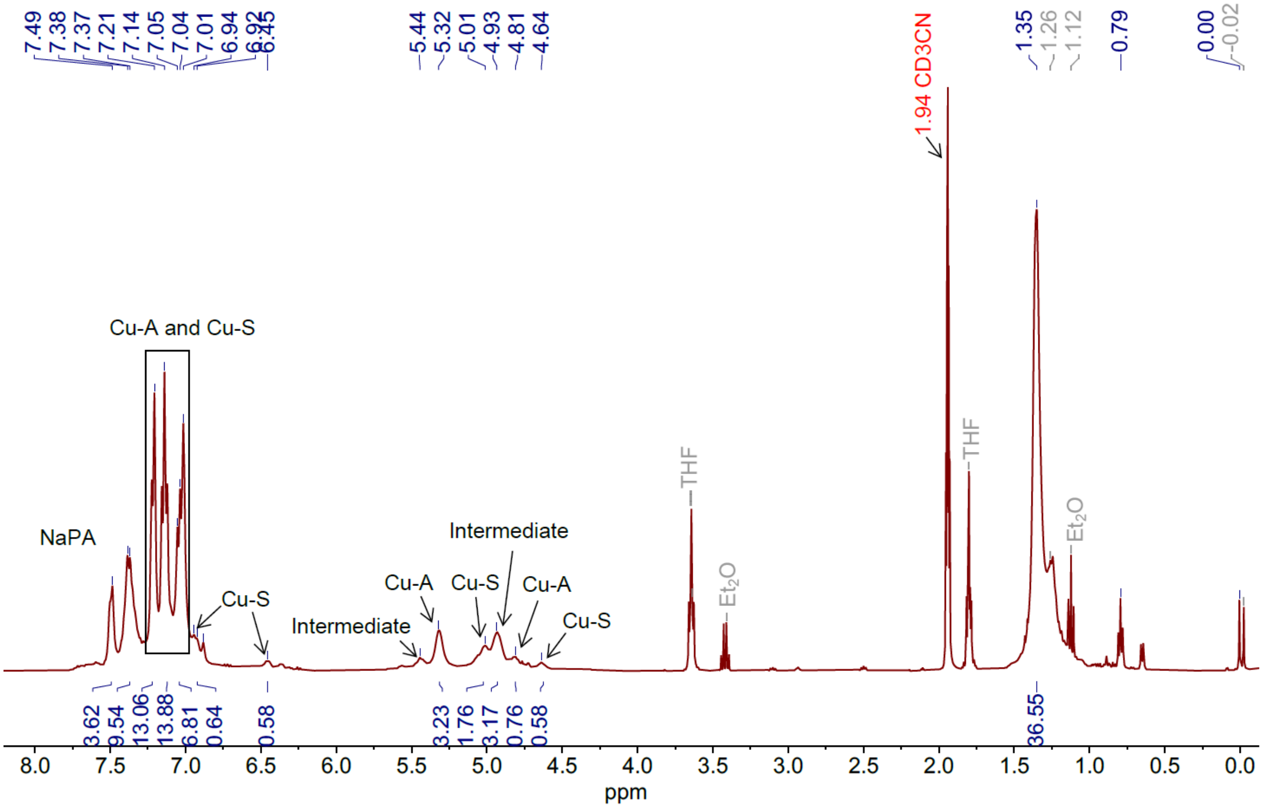

Figure S19. (a) ^{1}H NMR spectrum of the reaction of [Cu-**L1**]PF_6 and sodium phenylacetylide (5 equiv.) in CD_3CN upon mixing at RT (400 MHz).

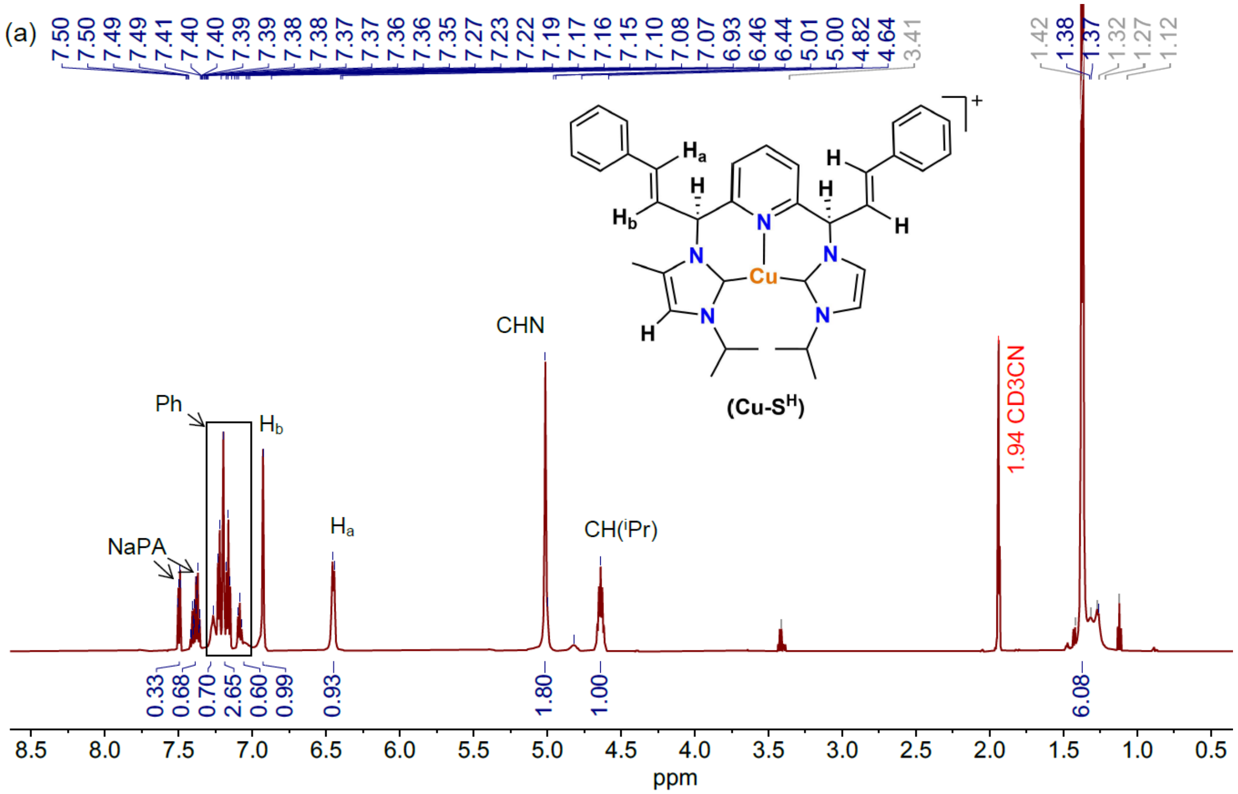

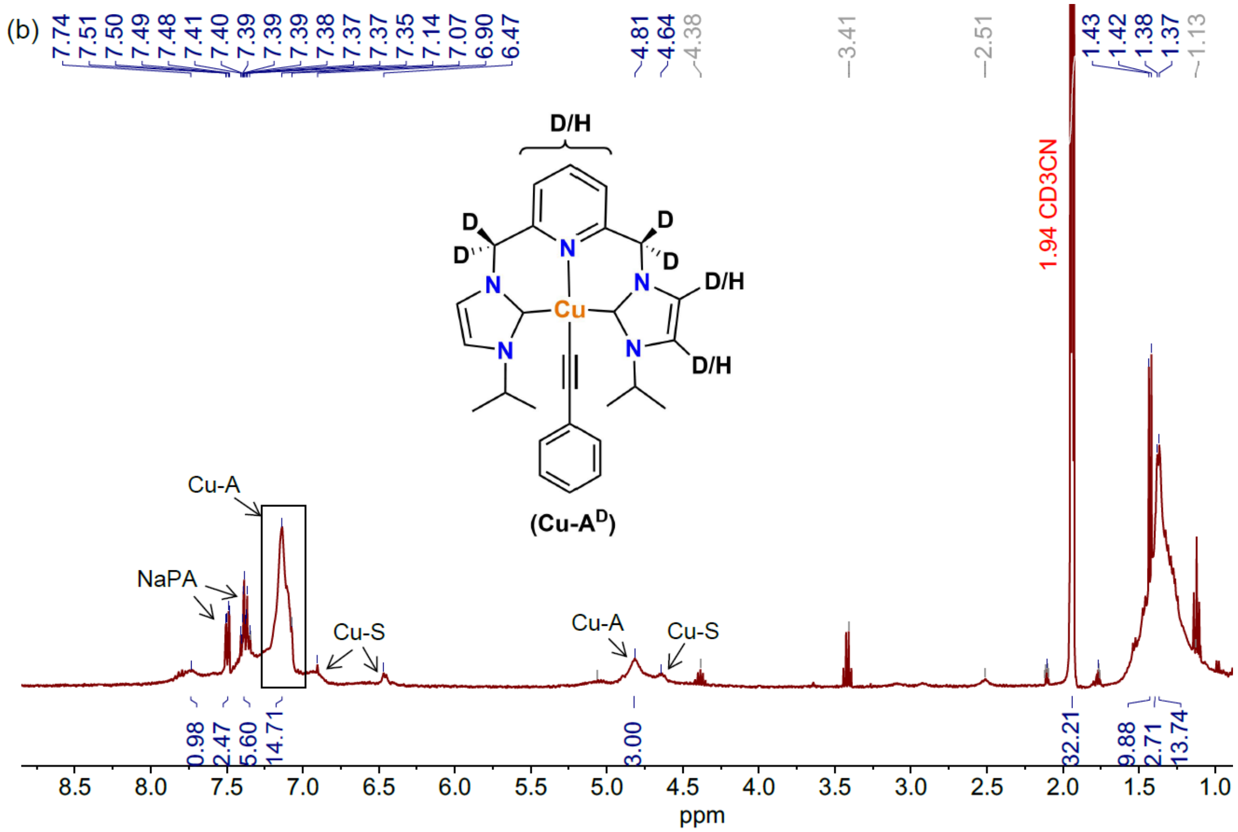

Figure S20. ^{1}H NMR spectra of the reaction of [Cu-**L1**]PF_6 and sodium phenylacetylide (1 equiv.) in CD_3CN after stirring for (a) 1 hour at room temperature and (b) 12 hours at 80 °C (400 MHz).

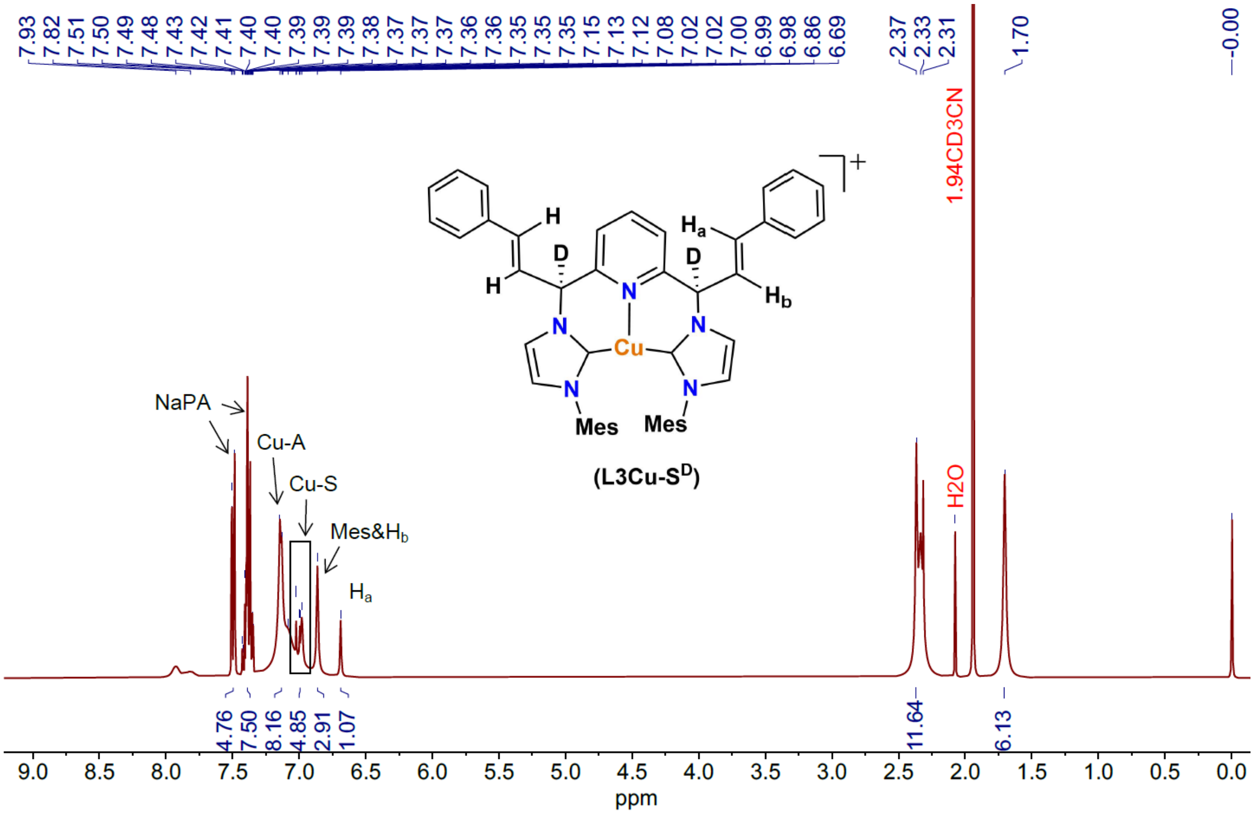

Figure S21. ^{1}H NMR spectrum of the reaction of [Cu-**L3**]PF_6 and sodium phenylacetylide (1 equiv.) in CD_3CN upon mixing at RT (400 MHz). Solvent impurity peaks are removed for clarity.

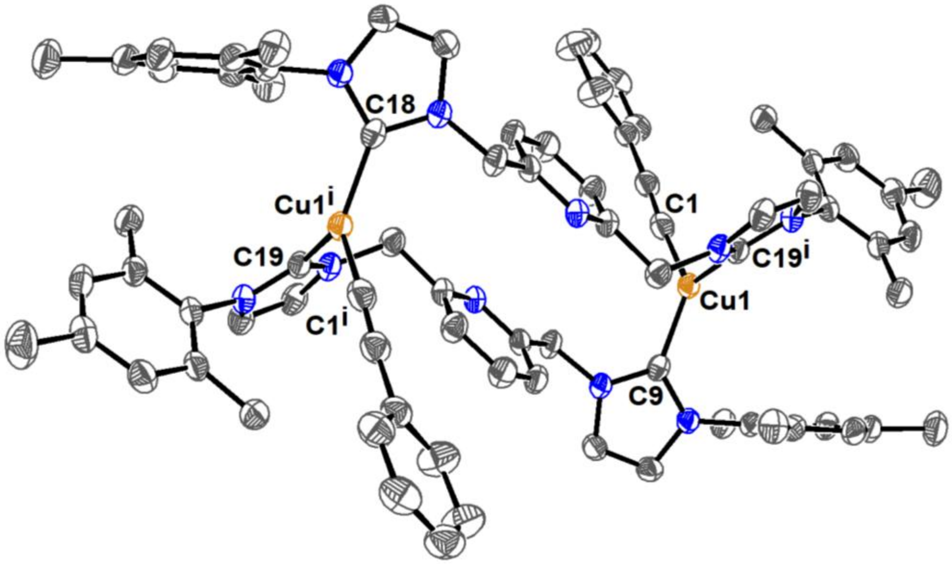

Figure S22. (a) ORTEP diagrams of **L3**-bound **Cu-A** complex. Hydrogen atoms have been omitted for clarity. Selected distances (Å) and angles (°); Cu(1)–C(1) = 1.928(2), Cu(1)–C(9) = 1.957(2), Cu(1)–C(19) = 1.978(2), C(9)–Cu(1)–C(19) = 123.19(9), C(9)–Cu(1)–C(1) = 124.0(1), C(1)–Cu(1)–C(19) = 111.5(1). Ellipsoids are shown at the 60% probability level.

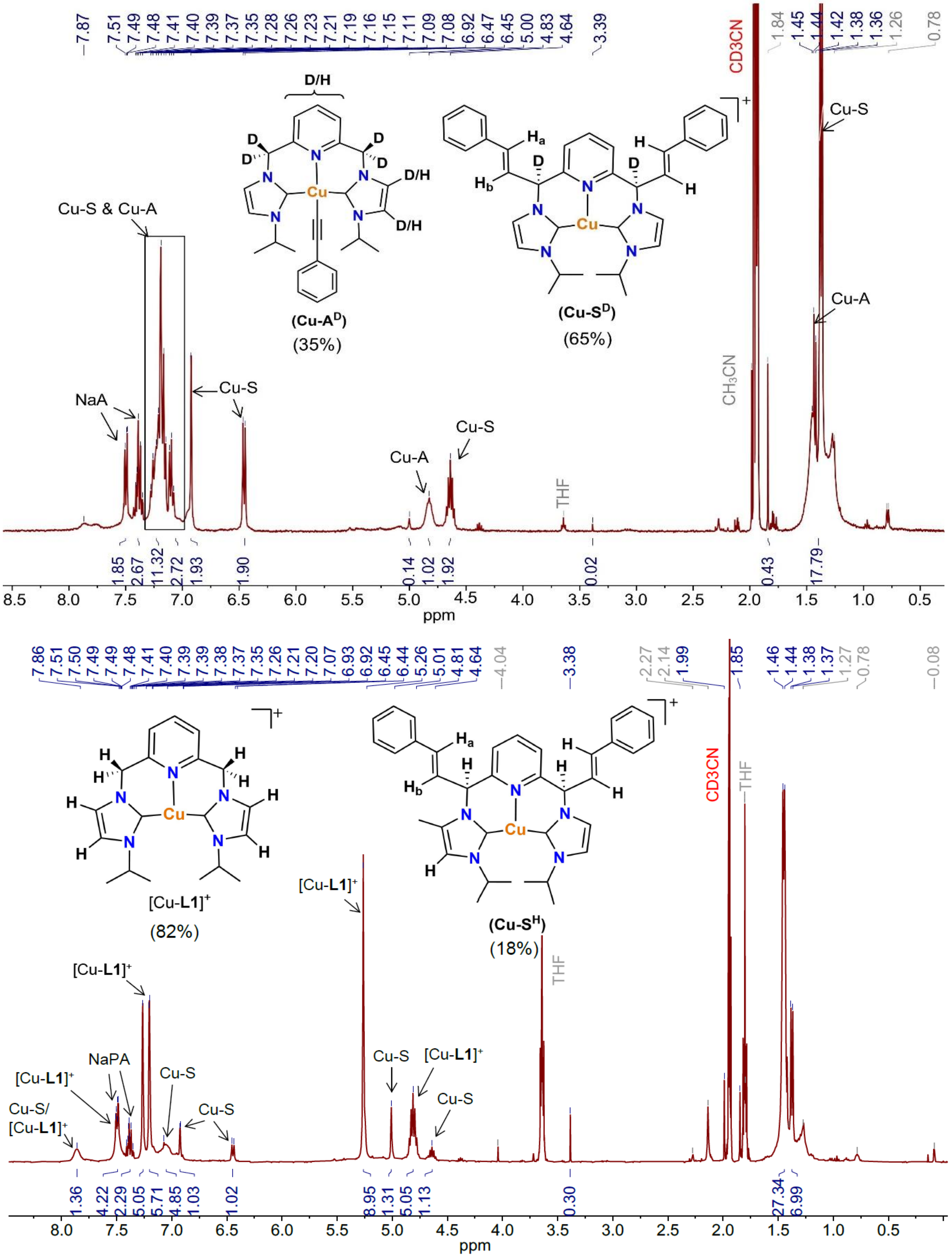

Figure S23. ^{1}H NMR spectrum of the crude solid from the reaction of [Cu-**L1**]PF_6 and sodium phenylacetylide (1 equiv.) after solvent removal and redissolving in CD_3CN (top) and after THF trituration, solvent removal, and redissolving in CD_3CN (bottom) at RT (400 MHz).

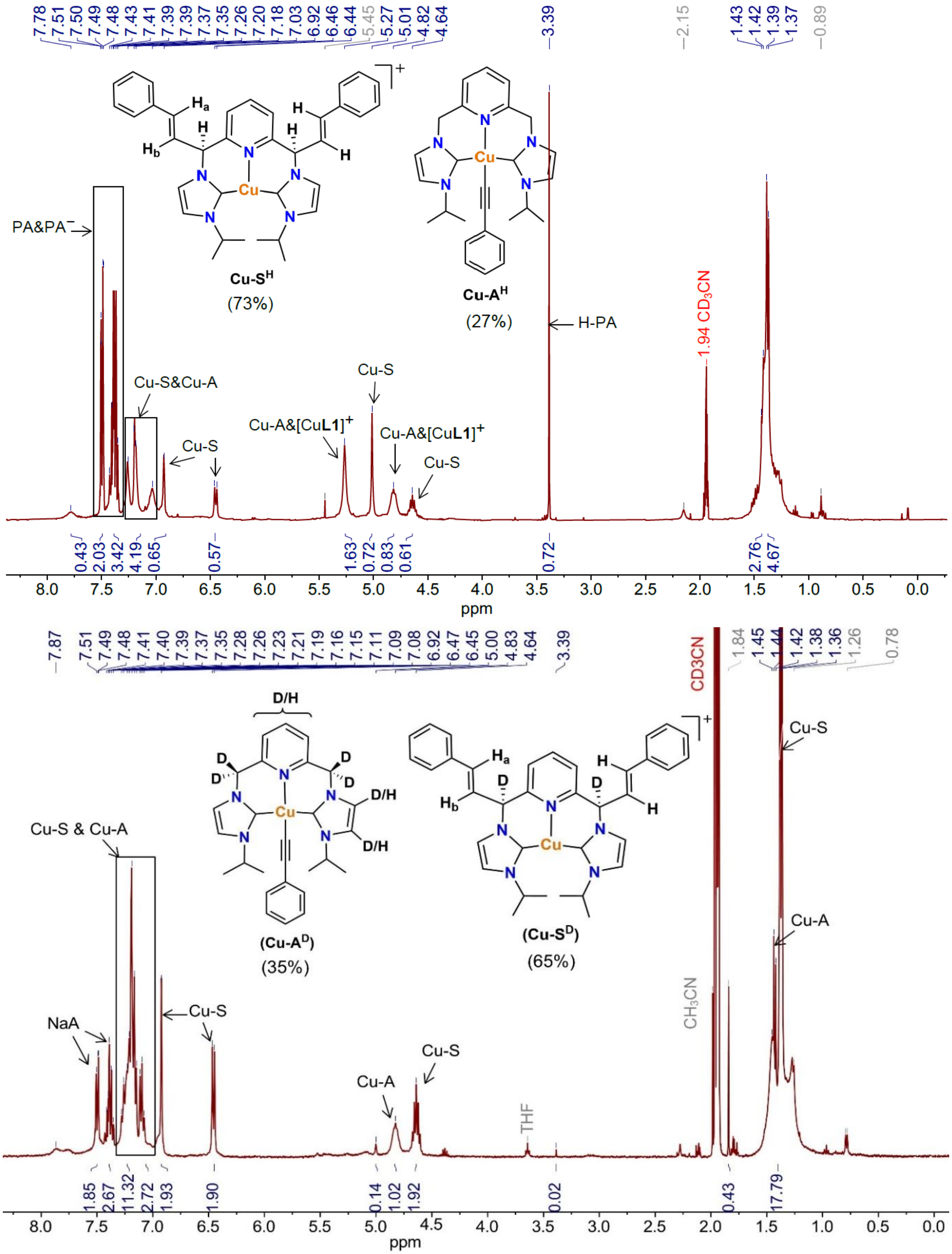

Figure S24. ^{1}H NMR spectra of the reaction of [Cu-**L1**]PF_6 and phenylacetylene (1 equiv.) in the presence of (a) Cs_2CO_3 (1 equiv.) and (b) Cs_2CO_3 (5 equiv.) in CD_3CN after 24 hours of stirring at RT (400 MHz).

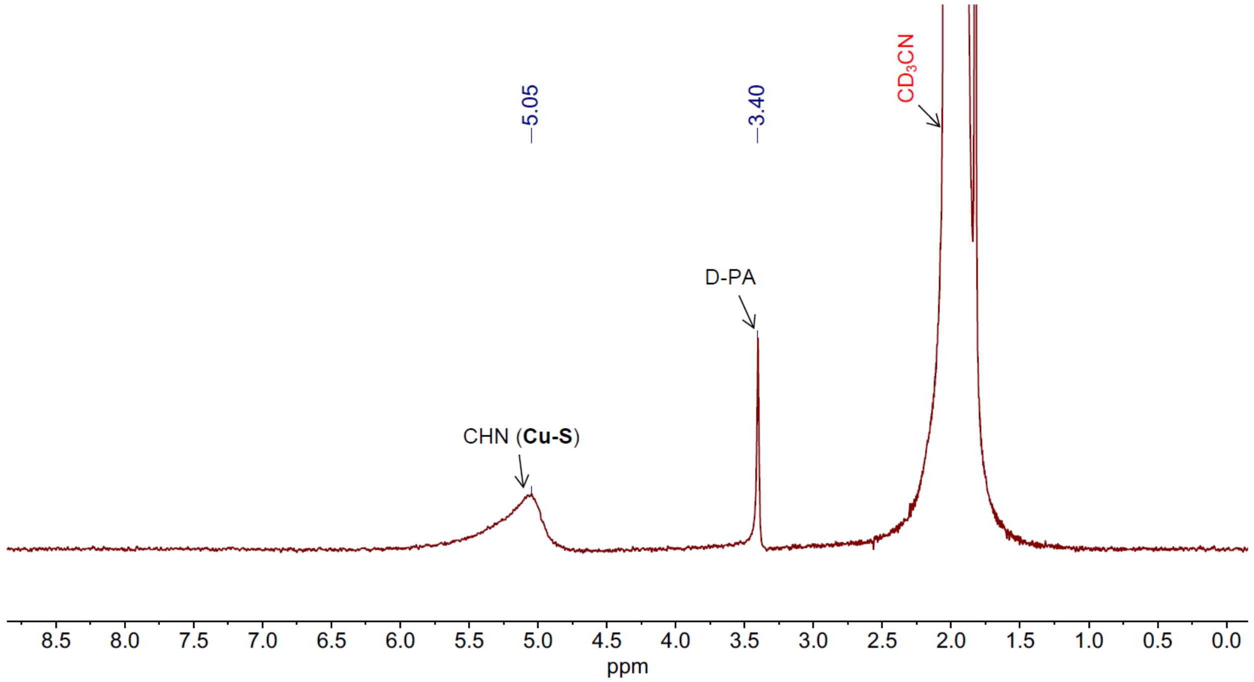

Figure S25. ^{2}H NMR spectra of the reaction of [Cu-**L1**]PF_6 and phenylacetylene (1 equiv.) in the presence of Cs_2CO_3 (5 equiv.) in CD_3CN after 24 hours of stirring at RT (400 MHz).

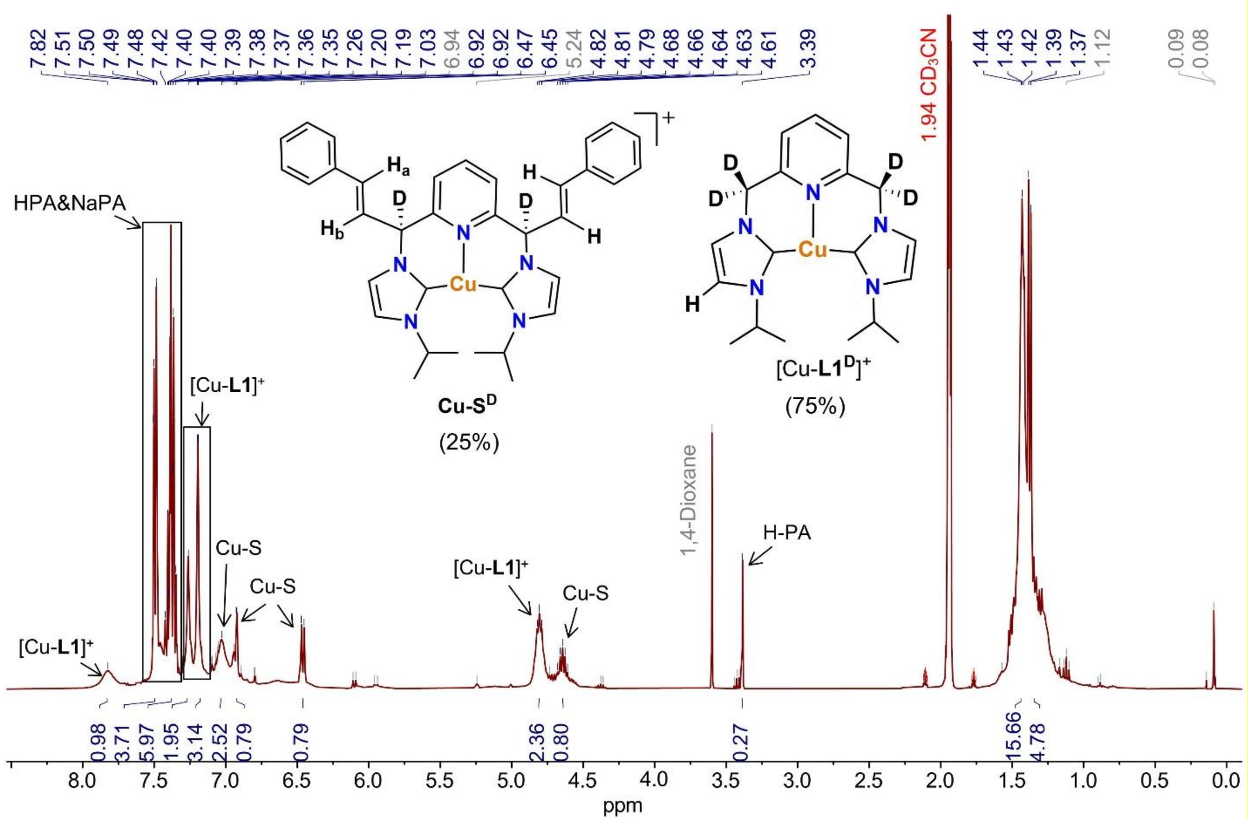

Figure S26. ^{1}H NMR spectrum of the reaction of [Cu-**L1**]PF_6, Cs_2CO_3 (2 equiv.), and phenylacetylene (1 equiv.) in CD_3CN after stirring for 12 h at RT and 12 h at 80 °C.

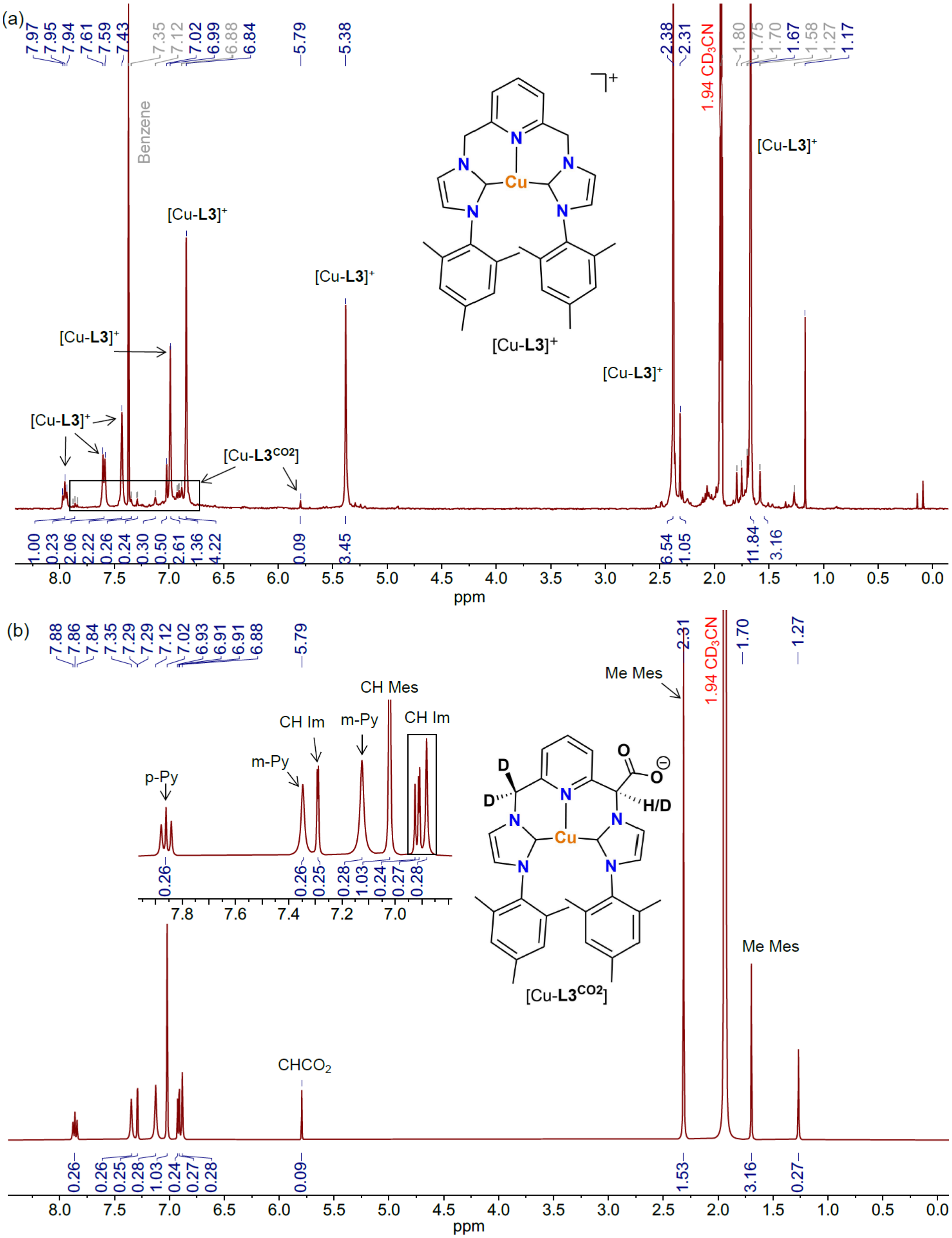

Figure S27. (a) ^{1}H NMR spectrum of the solid collected from the reaction of [Cu-**L3**]PF_6 (1 equiv.), and KO^tBu (5 equiv.) after stirring for 30 min at RT under CO_2 (1 atm). (b) ^{1}H NMR spectrum of the solid after removing the peaks of the starting complex and impurities.

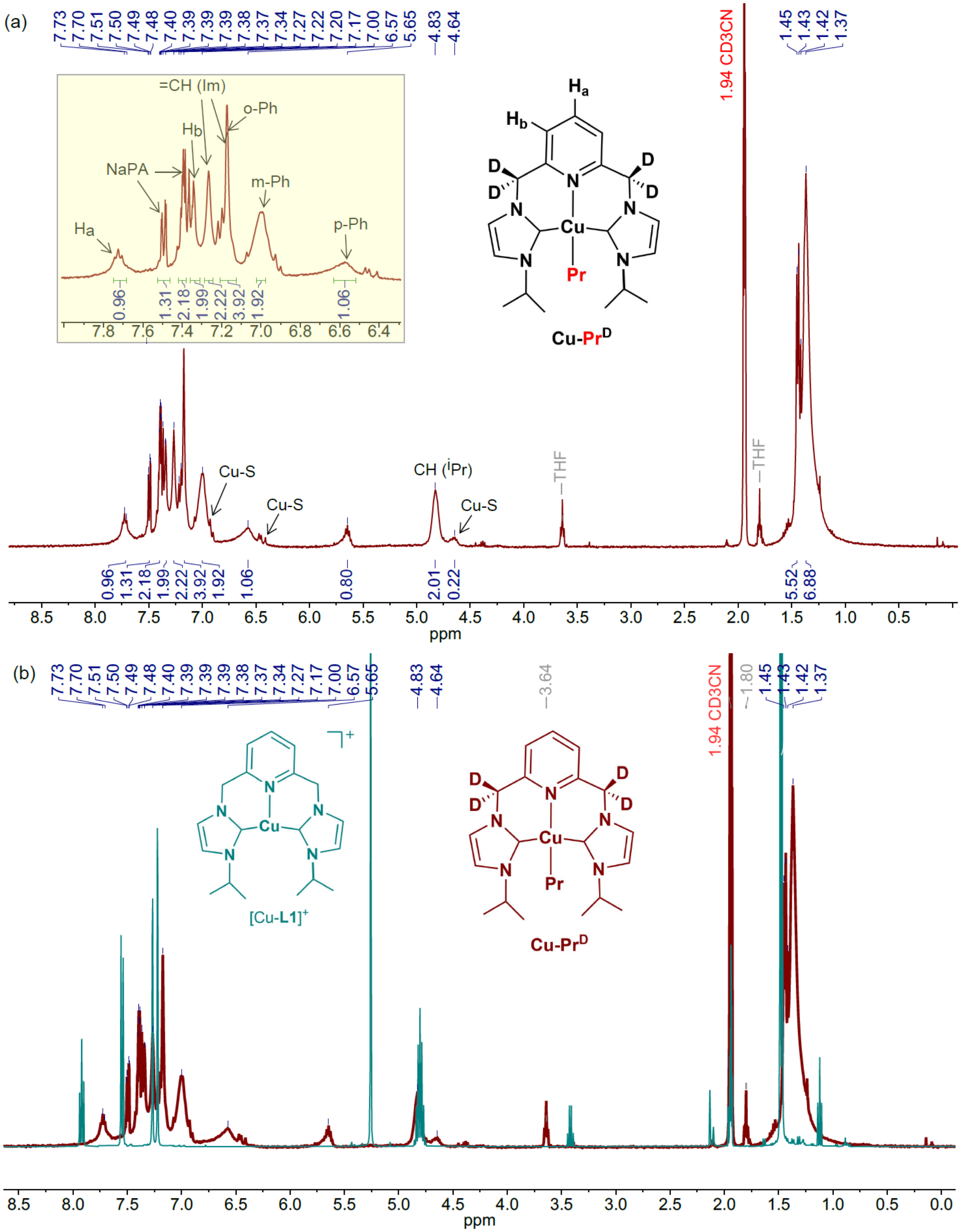

Figure S28. (a) ^{1}H NMR spectrum of the reaction of [Cu-**L1**]PF_6, sodium phenylacetylide (1 equiv.), and CO_2 (1 atm) in CD_3CN at RT. (b) Superimposed ^{1}H NMR spectra of **Cu-Pr** and the starting [Cu-**L1**]PF_6 complex in CD_3CN.

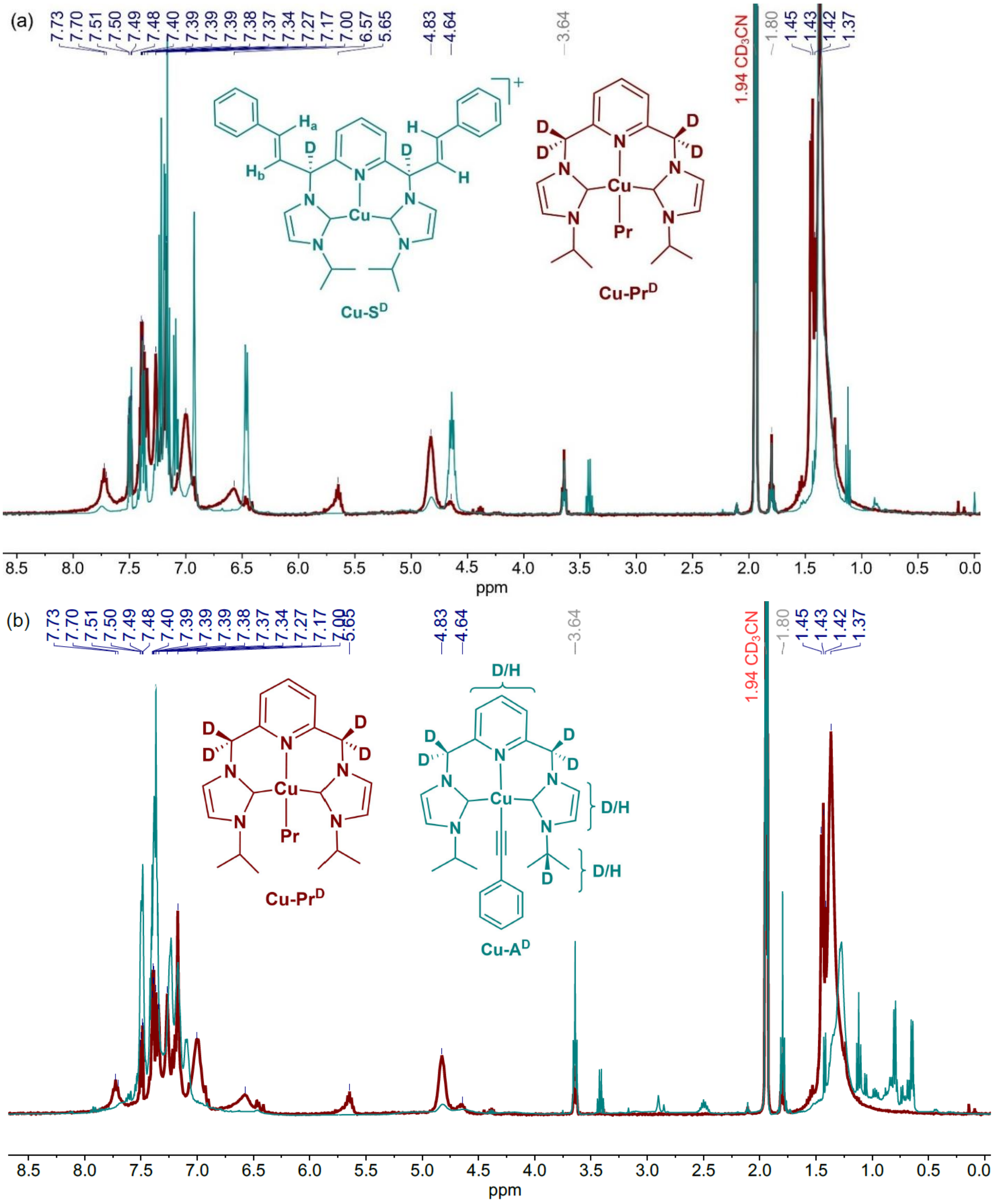

Figure S29. (a) Superimposed ^{1}H NMR spectra of the **Cu-Pr** and **Cu-S** complexes formed in situ from the corresponding reactions of [Cu-**L1**]PF_6 (1 equiv.) and sodium phenylacetylide in the presence and absence of CO_2 (1 atm) in CD_3CN, respectively. (b) Superimposed ^{1}H NMR spectra of the **Cu-Pr** and **Cu-A** complexes formed in situ in CD_3CN, respectively.

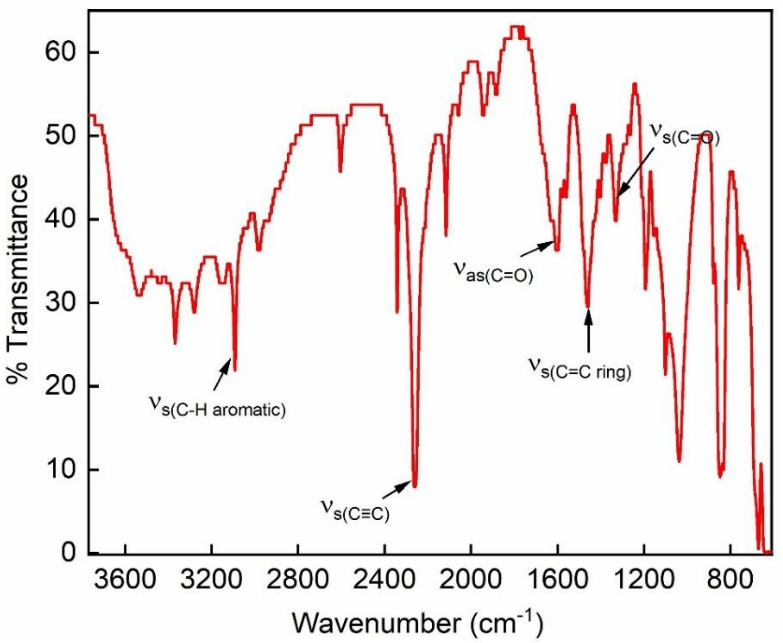

Figure S30. IR spectrum of the reaction of [Cu-**L1**]PF_6, sodium phenylacetylide (1 equiv.), and CO_2 (1 atm) in CD_3CN at RT.

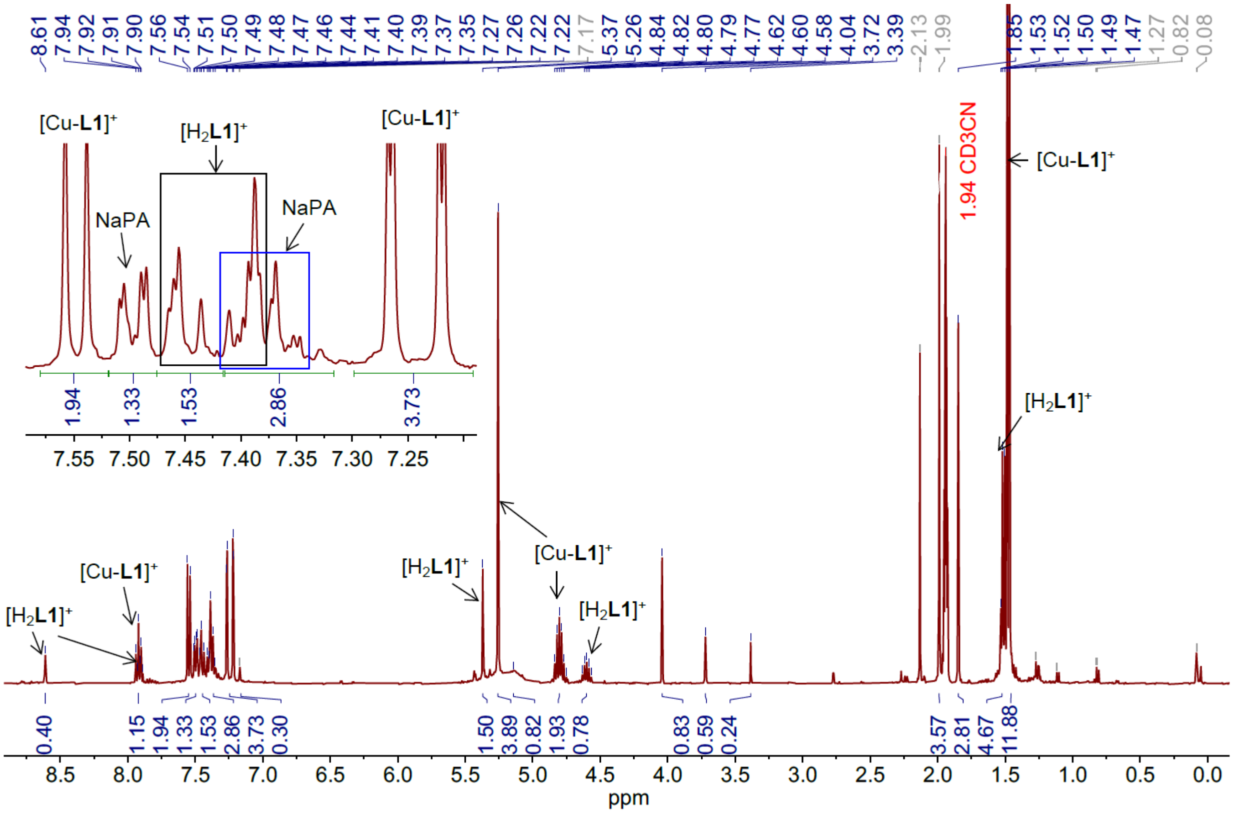

Figure S31. (a) ^{1}H NMR spectrum of the solid collected from the reaction of [Cu-**L1**]PF_6 (1 equiv.), sodium phenylacetylide, and CO_2 (1 atm) in CD_3CN after solvent removal under vacuum.

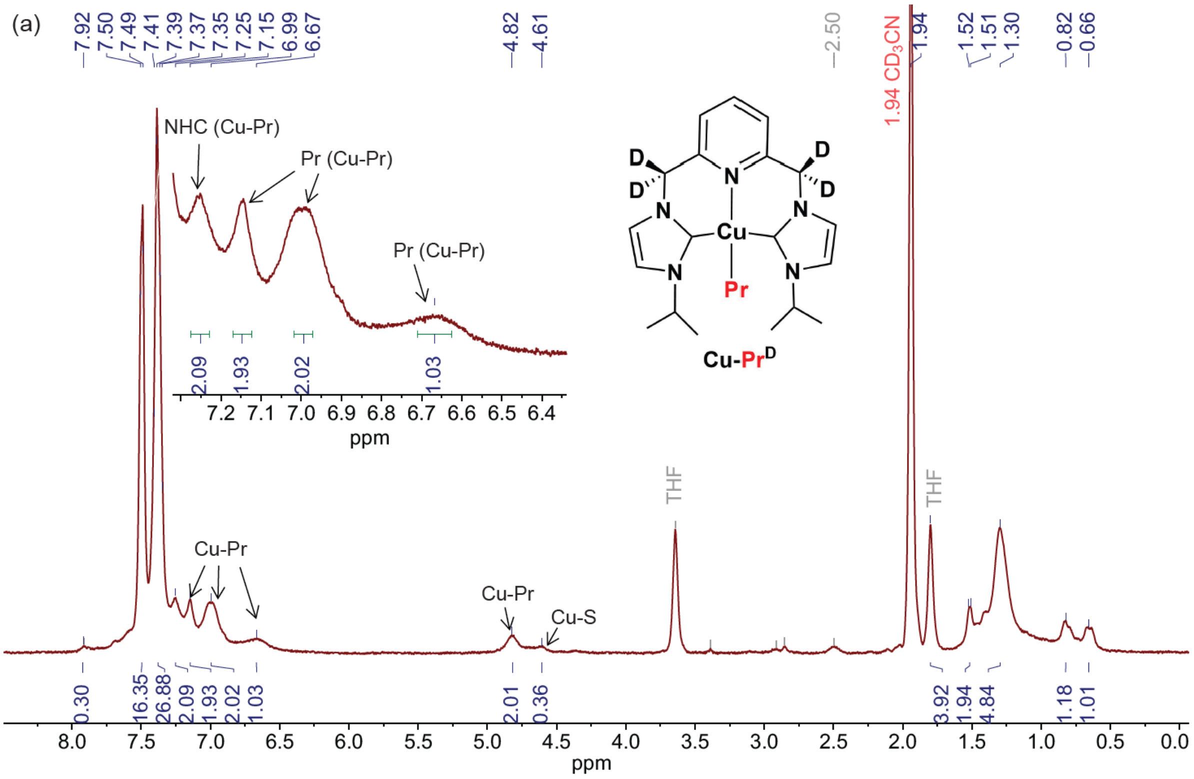

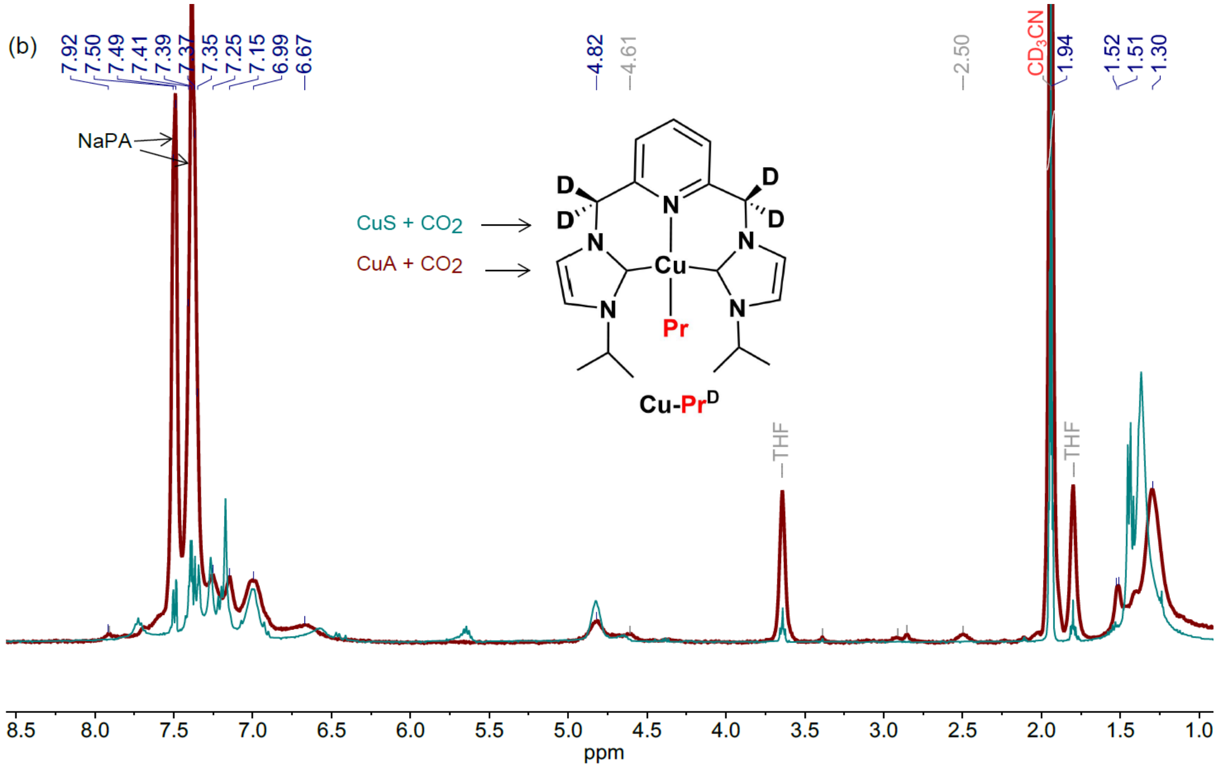

Figure S32. (a) ^{1}H NMR spectrum of the reaction of [Cu-**L1**]PF_6, sodium phenylacetylide (5 equiv.), and CO_2 (1 atm) in CD_3CN after stirring for 24 h at RT. (b) Superimposed ^{1}H NMR spectra of the **Cu-Pr** formed from the reactions of **Cu-S** and **Cu-A** complexes with CO_2 in CD_3CN at RT.

^{1}H NMR and ^{13}C NMR spectroscopy data of bis-imidazolium salts and Cu-CNC complexes

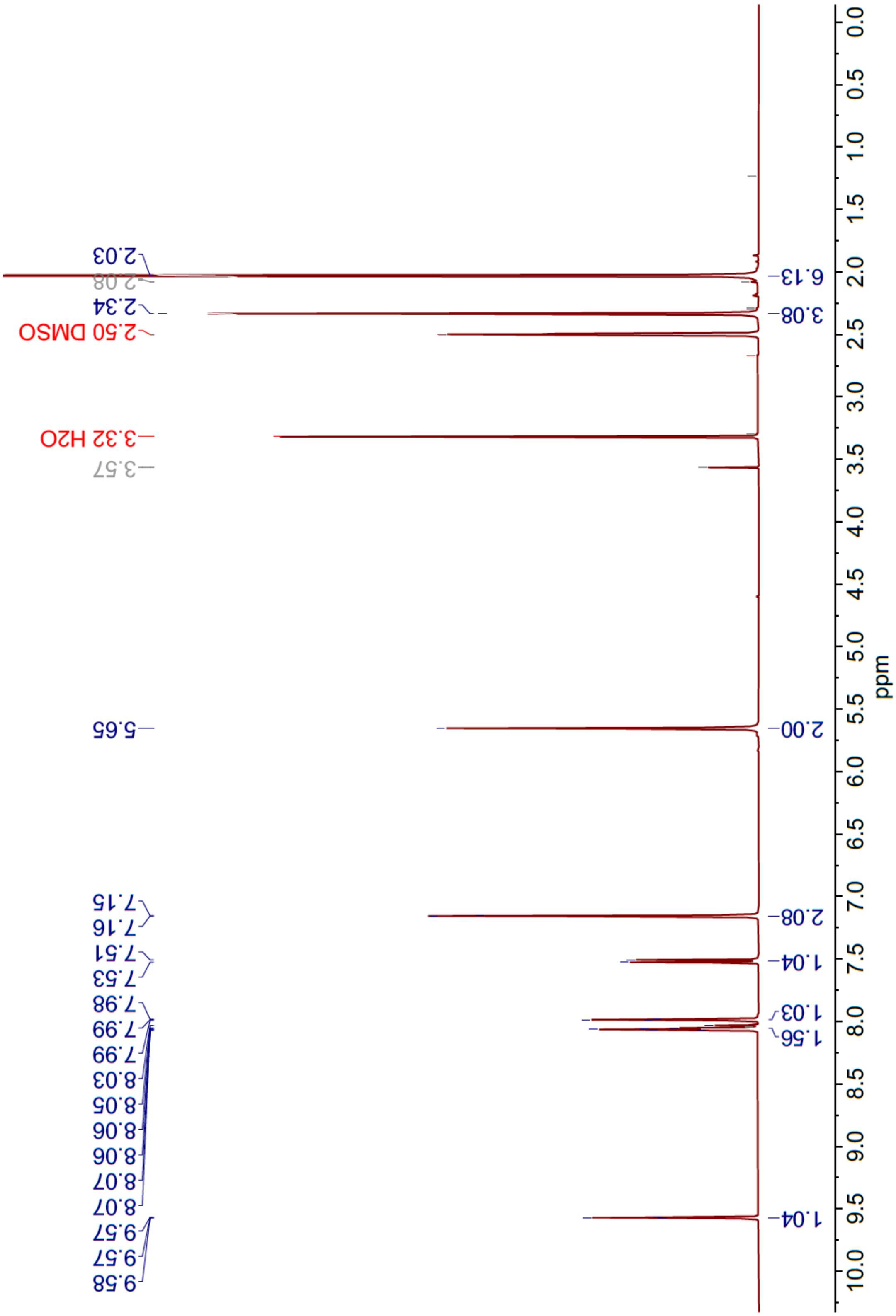

Figure S33. ^{1}H-NMR spectrum of *I(Mes)$^{C\wedge N\wedge C}$·2HBr*, **L3.**2HBr in DMSO-d_6 (400 MHz)

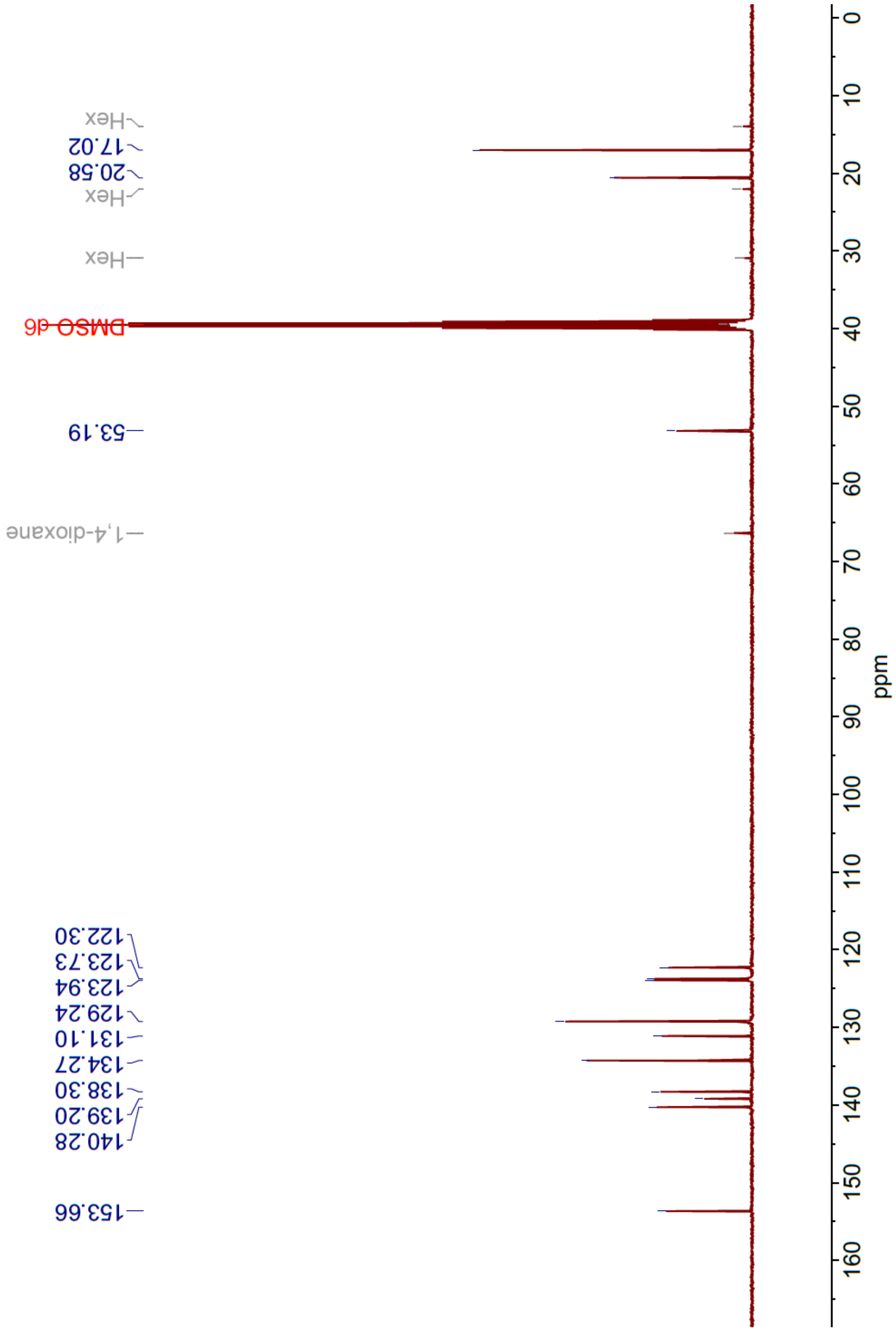

Figure S34. ^{13}C-NMR spectrum of *I(Mes)$^{C\text{^}N\text{^}C}$•2HBr*, **L3.**2HBr in DMSO-d_6 (101 MHz)

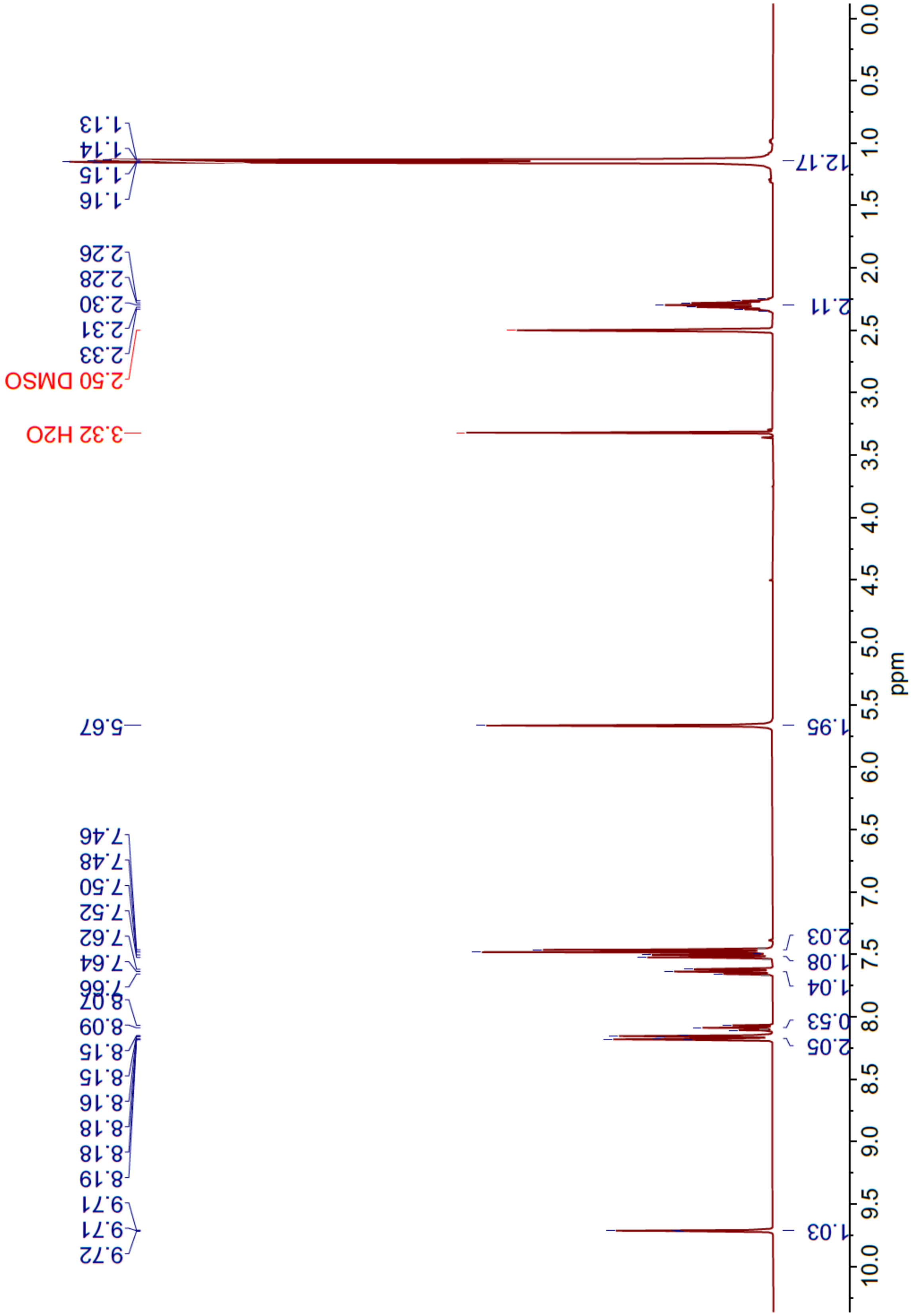

Figure S35. ^{1}H-NMR spectrum of *I(Dipp)*$^{C^\wedge N^\wedge C}$*·2HBr*, **L4.**2HBr in DMSO-d_6 (400 MHz)

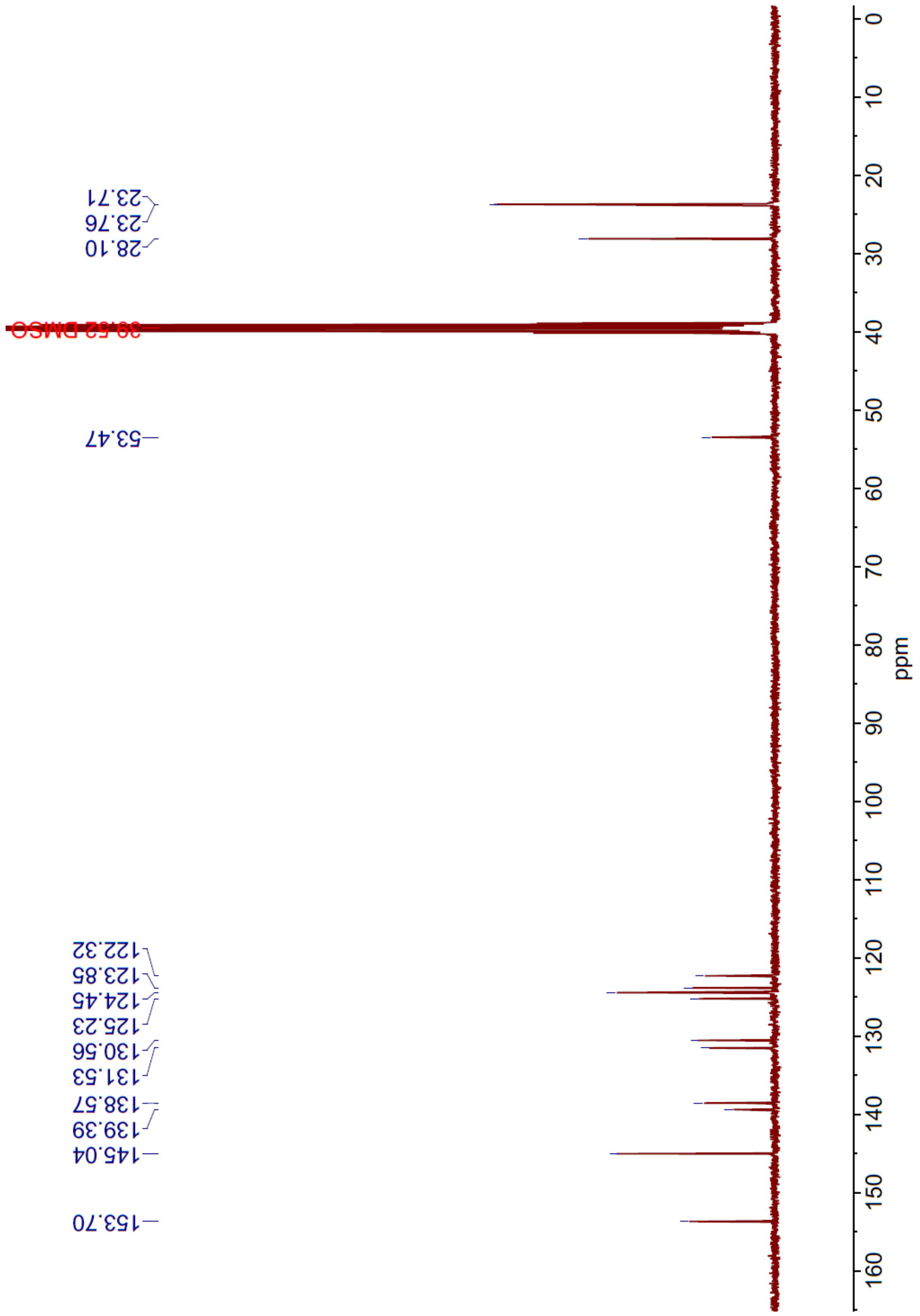

Figure S36. ^{13}C-NMR spectrum of *I(Dipp)*$^{C^\wedge N^\wedge C}$*·2HBr*, **L4.**2HBr in DMSO-*d*$_6$ (101 MHz)

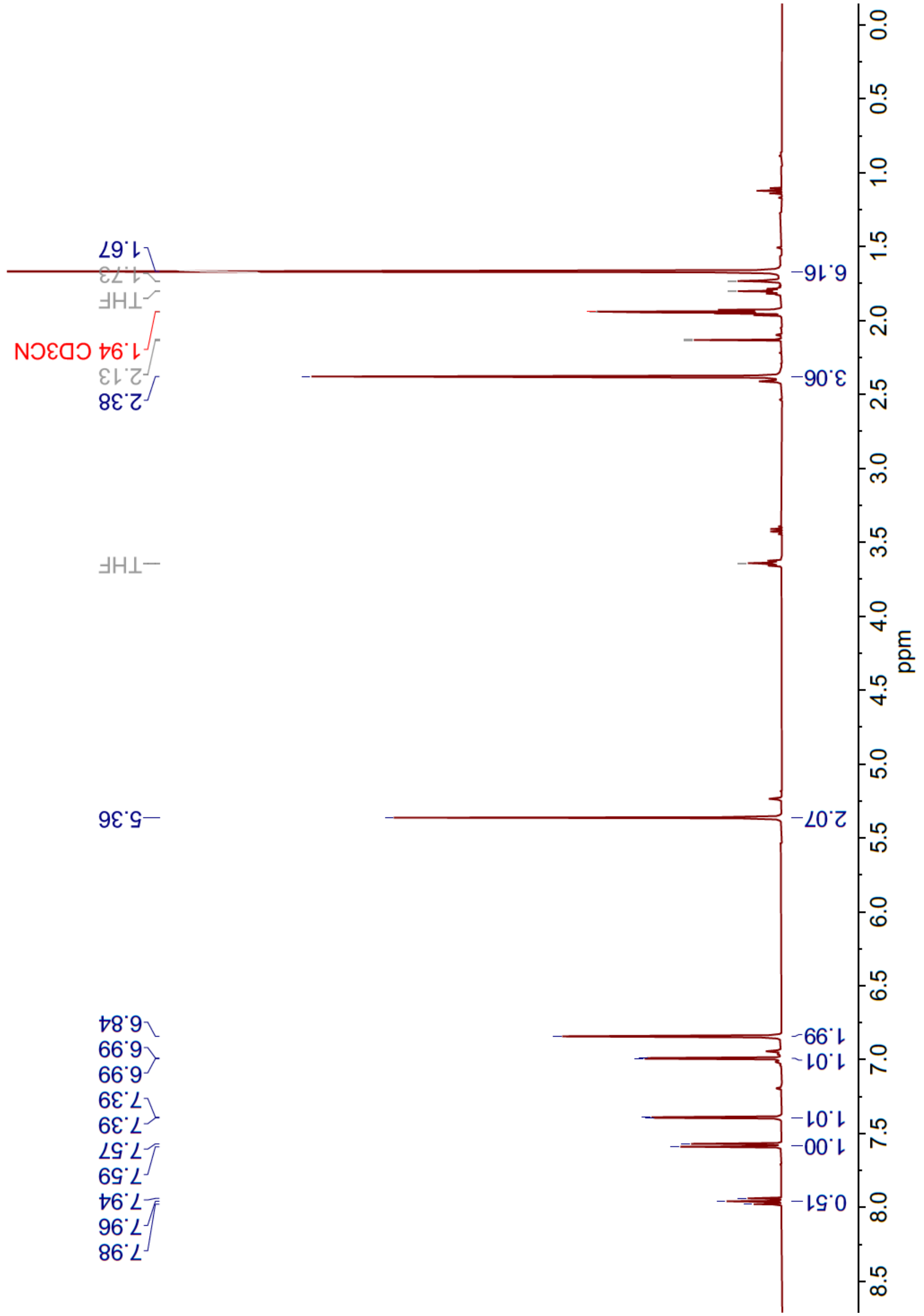

Figure S37. ^{1}H-NMR spectrum of [*Cu(IMes$^{C\wedge N\wedge C}$)]PF$_6$*, [Cu-**L3**]PF_6 in CD_3CN (400 MHz)

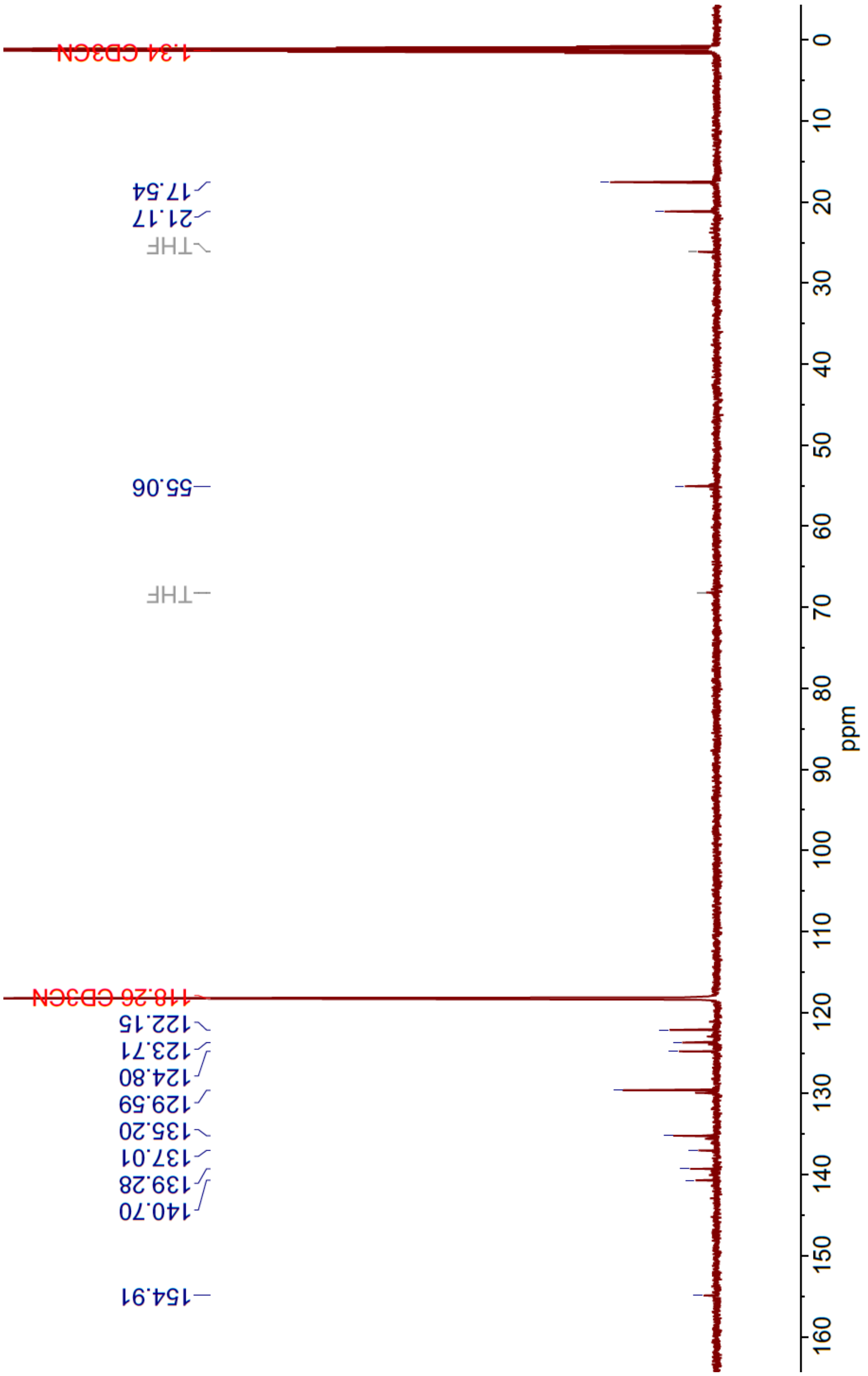

Figure S38. ^{13}C-NMR spectrum of [*Cu(IMes$^{C\wedge N\wedge C}$)]PF$_6$*, [Cu-**L3**]PF_6 in CD_3CN (150 MHz)

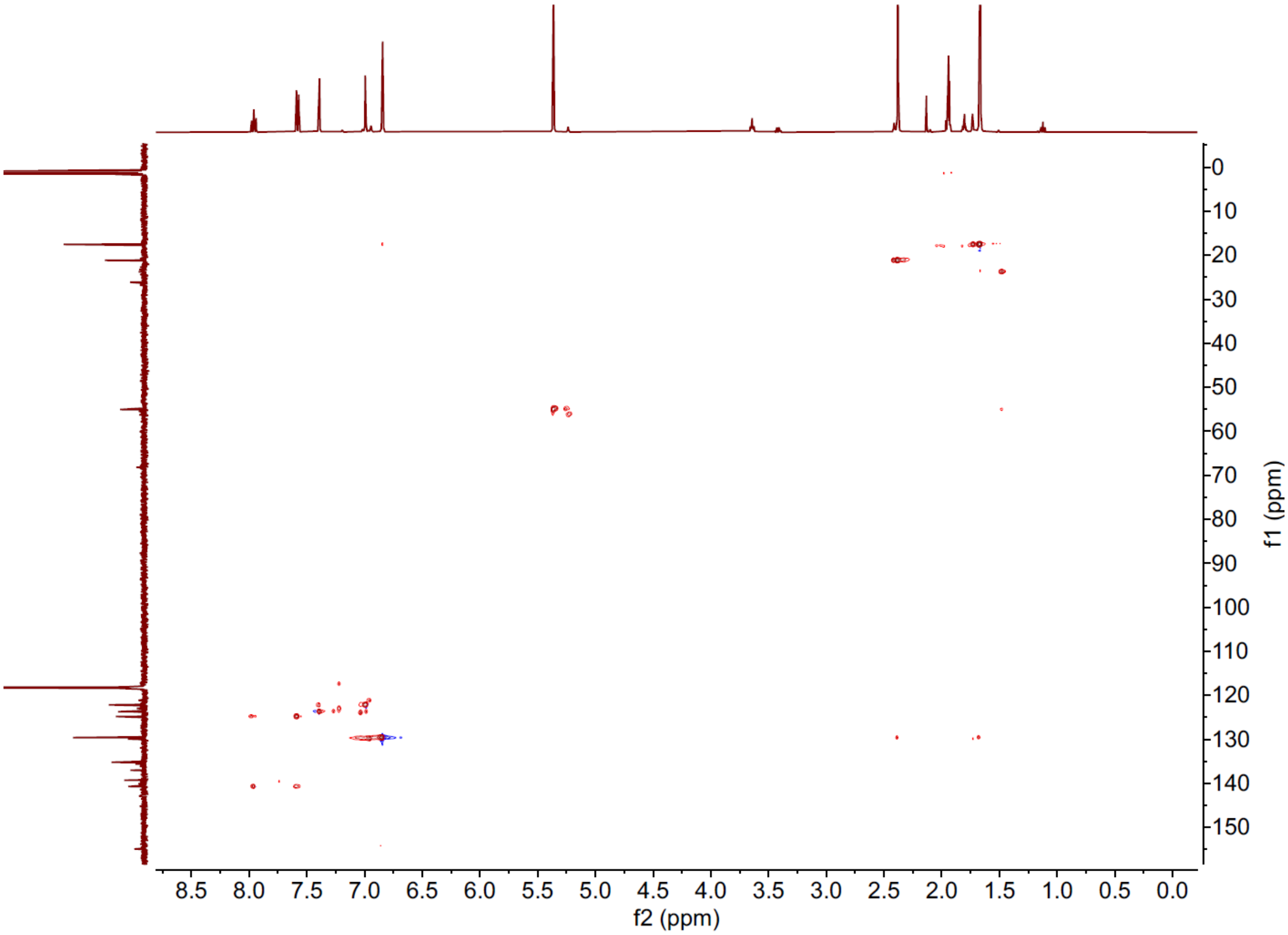

Figure S39. ^{1}H-^{13}C HSQC spectrum of [*Cu(IMes$^{C\wedge N\wedge C}$)]PF$_6$*, [Cu-**L3**]PF$_\mathbf{6}$ in CD_3CN.

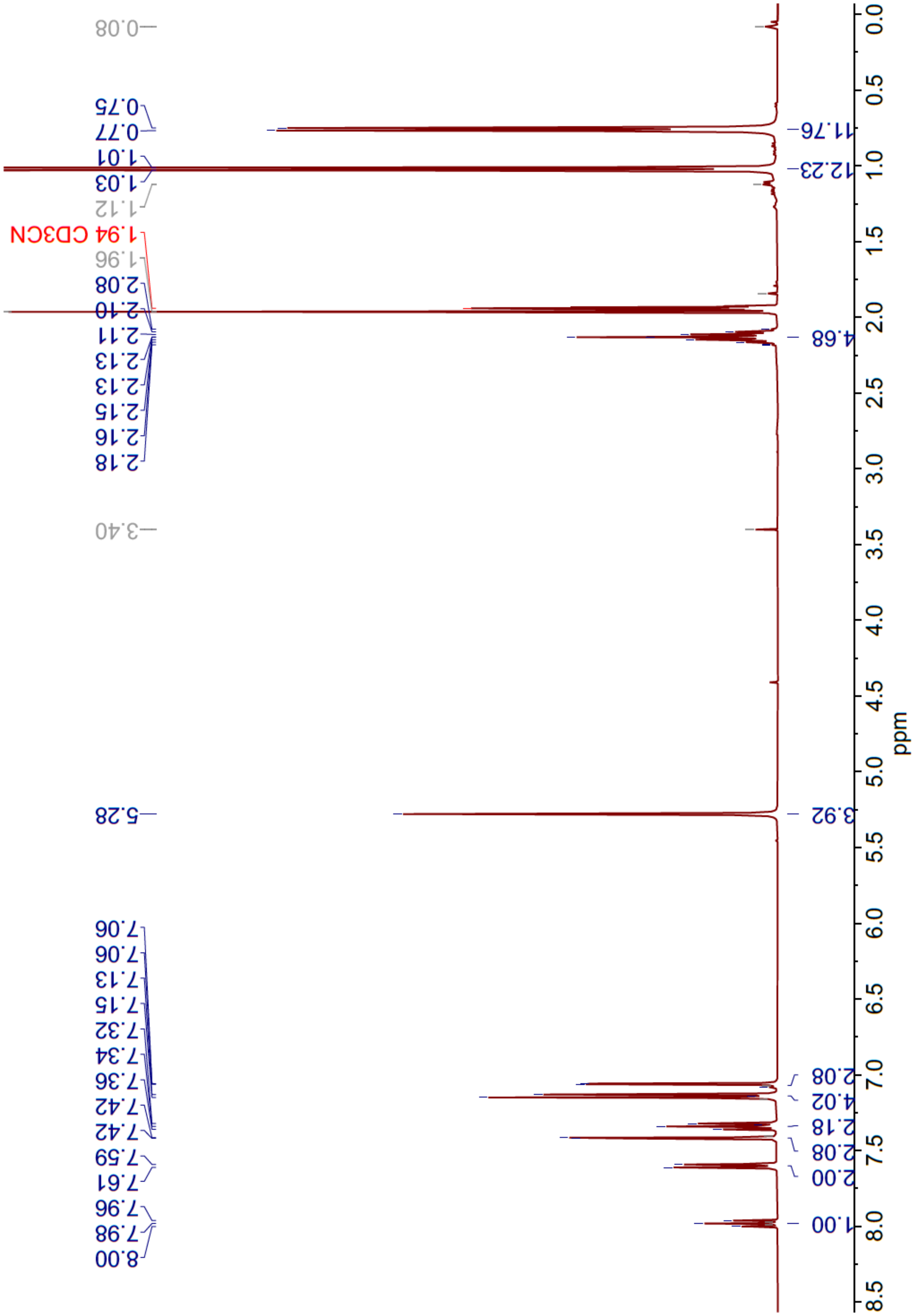

Figure S40. ^{1}H-NMR spectrum of *[Cu(IDipp$^{C^\wedge N^\wedge C}$)]PF$_6$*, [Cu-**L4**]PF$_6$ in CD_3CN (400 MHz)

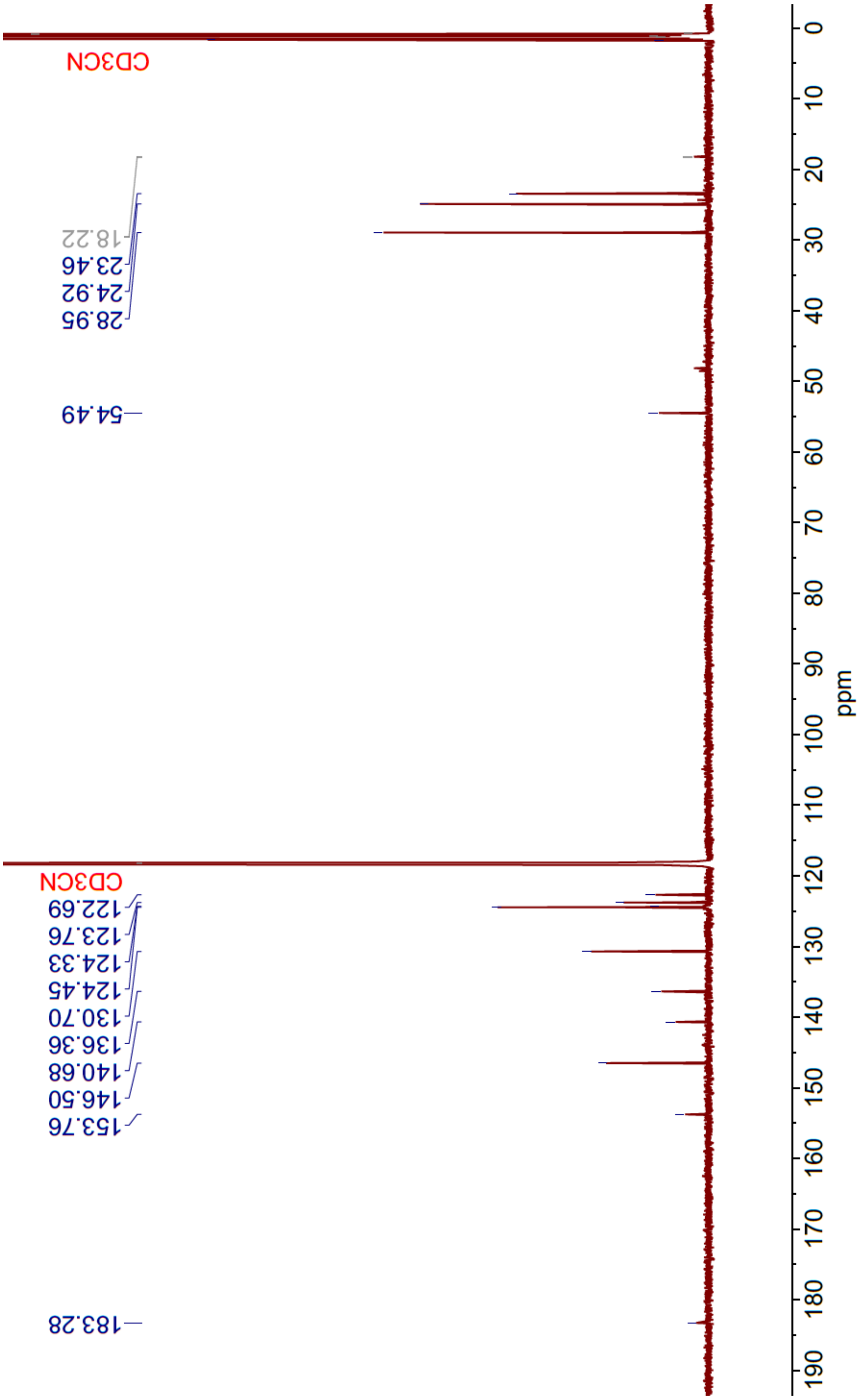

Figure S41. ^{13}C-NMR spectrum of *[Cu(IDipp$^{C\wedge N\wedge C}$)]PF$_6$*, [Cu-**L4**]PF$_6$ in CD_3CN (101 MHz)

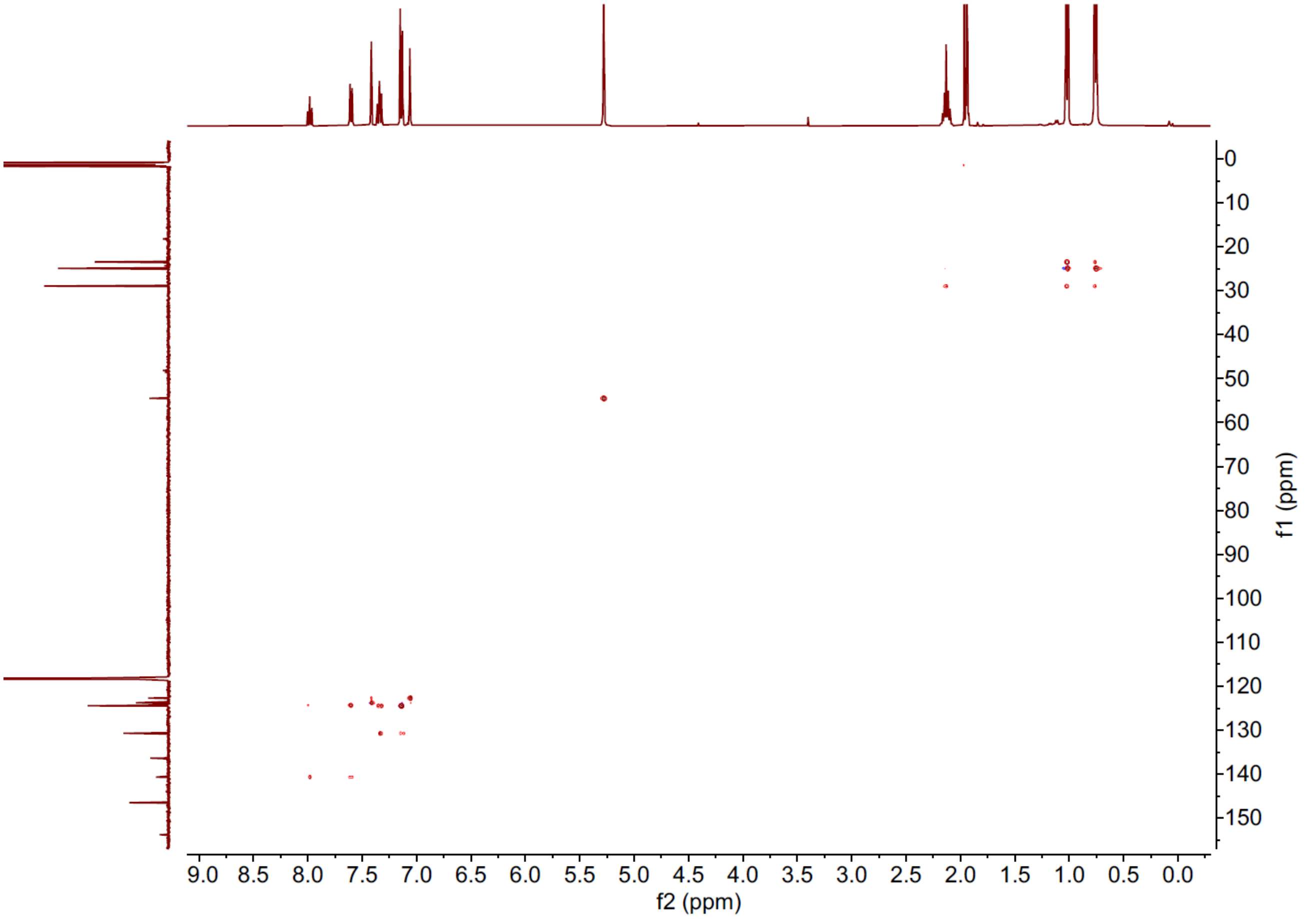

Figure S42. ^{1}H-^{13}C HSQC spectrum of [*Cu(IDipp*$^{C^\wedge N^\wedge C}$*)]PF*$_6$, [Cu-**L4**]PF_6 in CD_3CN.

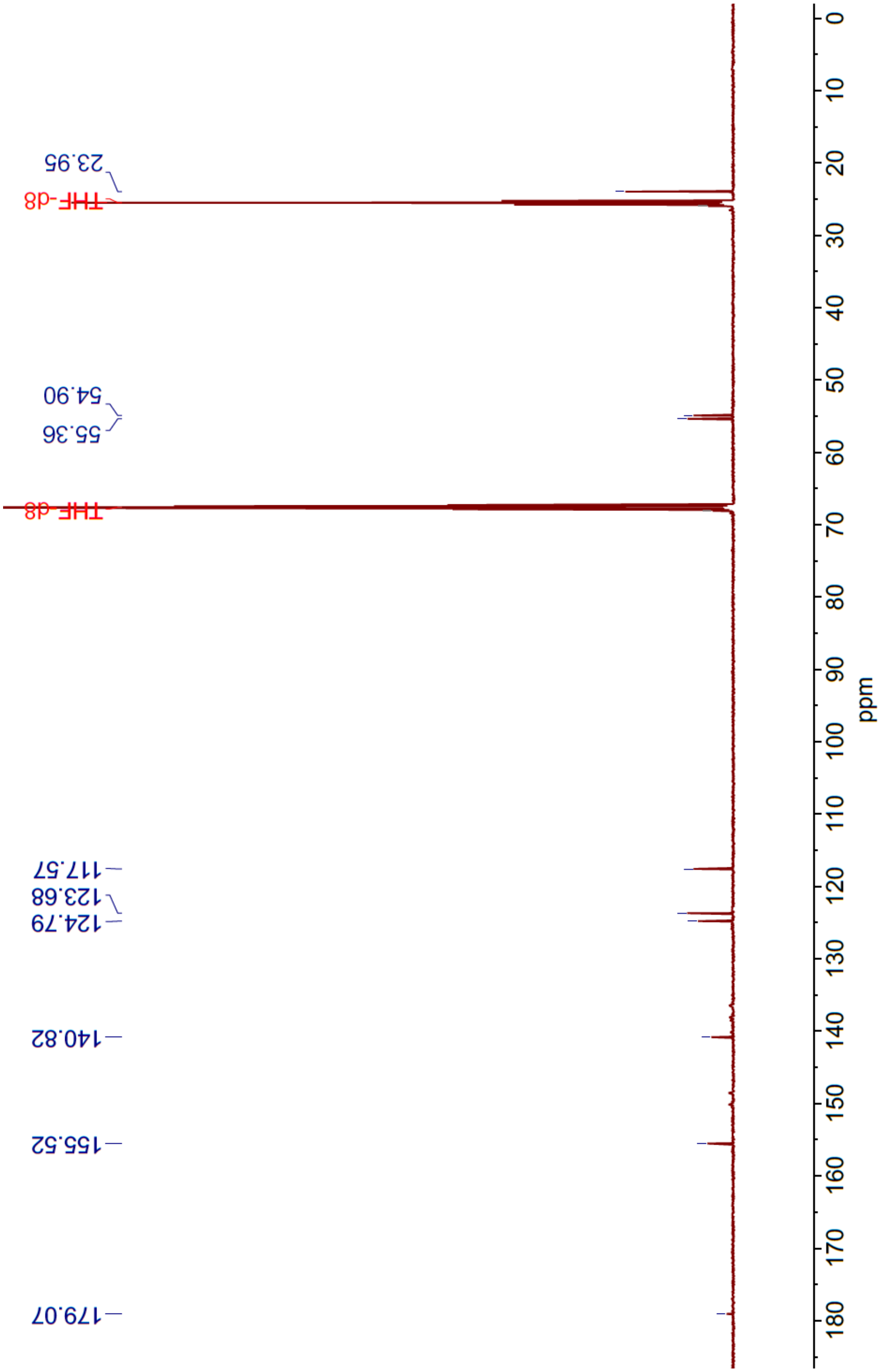

Figure S43. ^{13}C-NMR spectrum of *[Cu(I^{i}Pr$^{C\^{}N\^{}C}$)]BArF*, [Cu-**L1**]BArF in THF-d_8 (150 MHz)

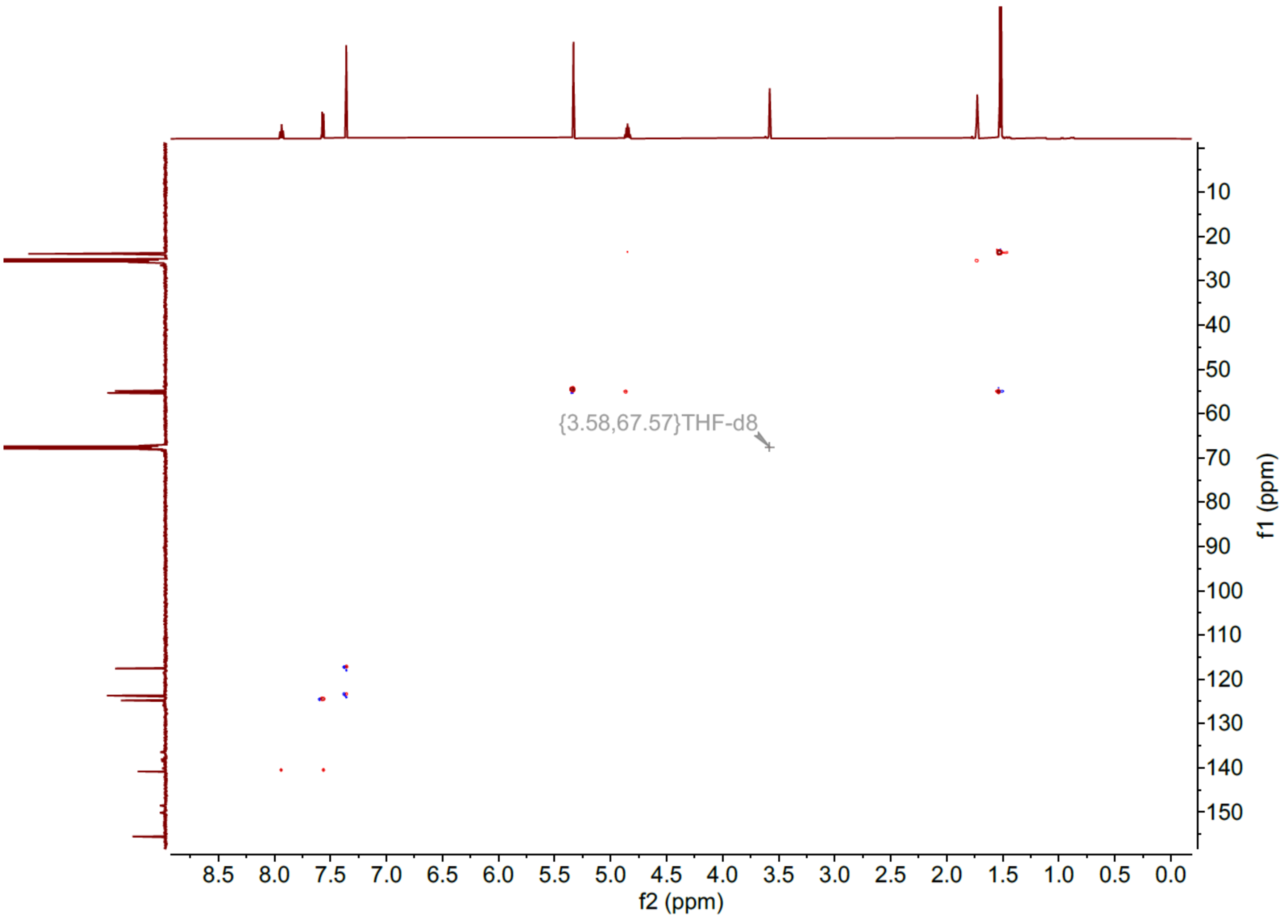

Figure S44. ^{1}H-^{13}C HSQC spectrum of *[Cu(I^{i}Pr$^{C\wedge N\wedge C}$)]BArF*, [Cu-**L1**]BArF in in THF-d_8

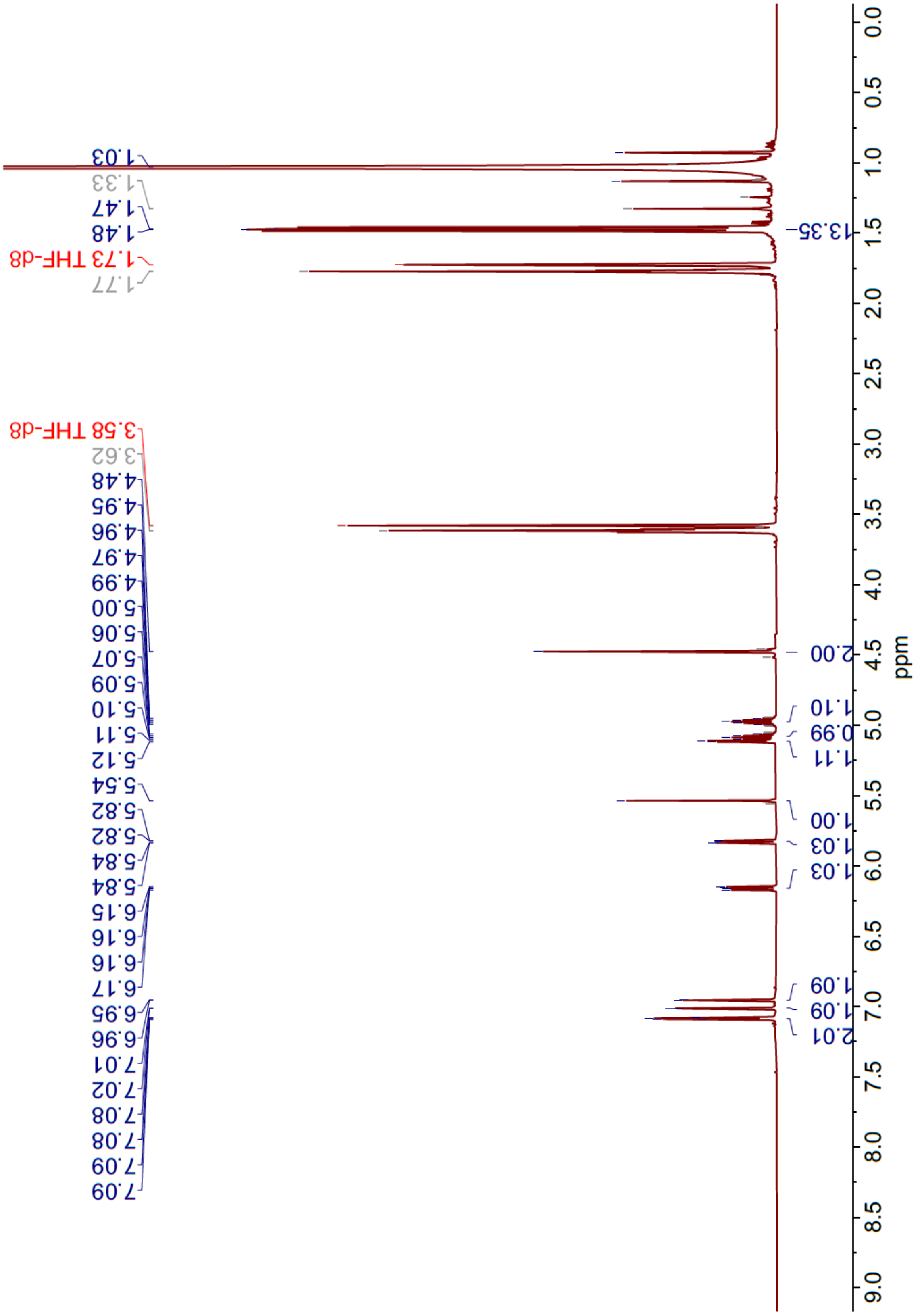

Figure S45. ^{1}H-NMR spectrum of *[Cu(I^{i}Pr$^{C\wedge N\wedge C}$)*]*, [Cu-**L1***] in THF-d_8 (600 MHz)

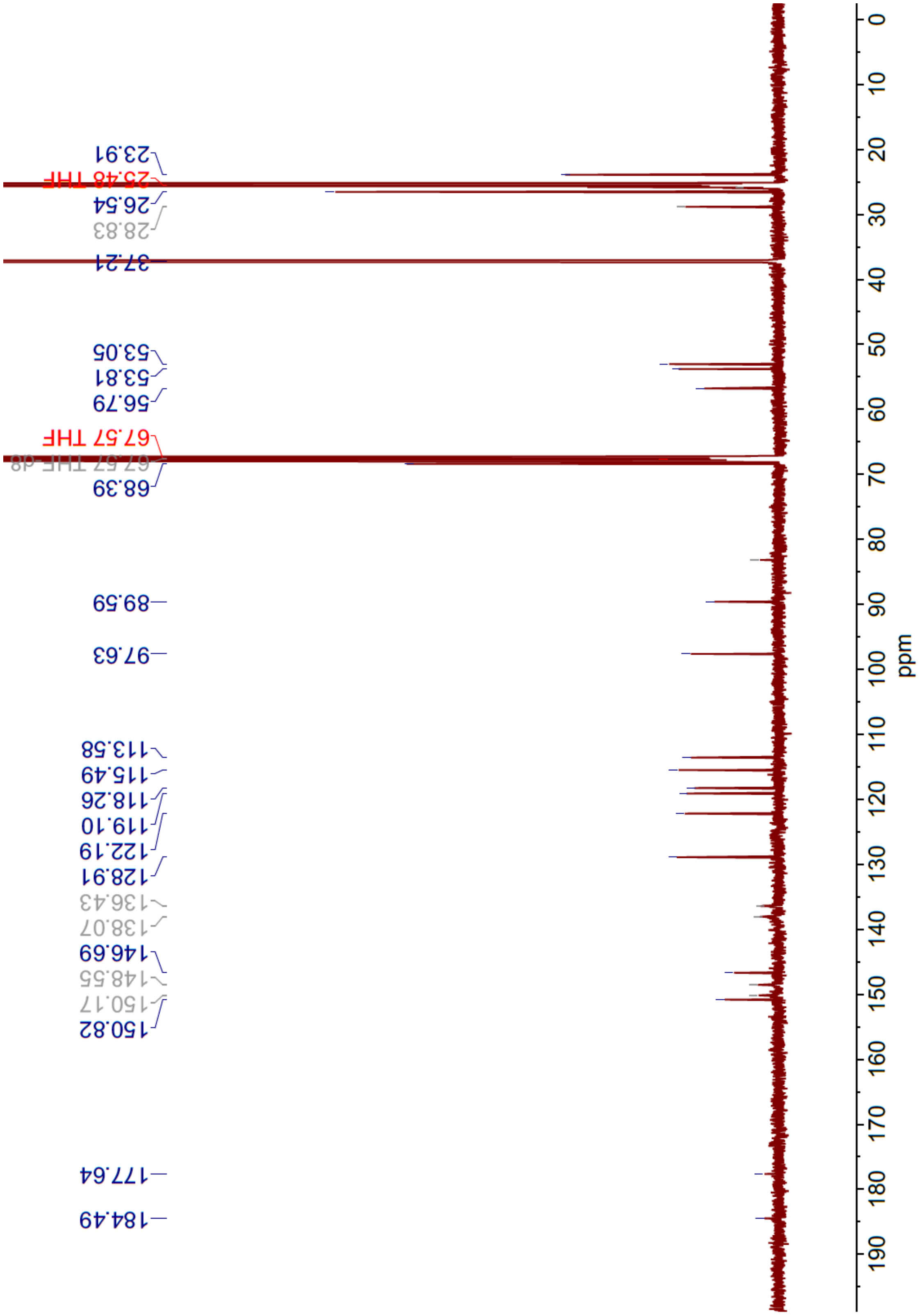

Figure S46. ^{13}C-NMR spectrum of *[Cu(I^{i}Pr$^{C\wedge N\wedge C}$)*]*, [Cu-**L1***] in THF-d_8 (150 MHz)

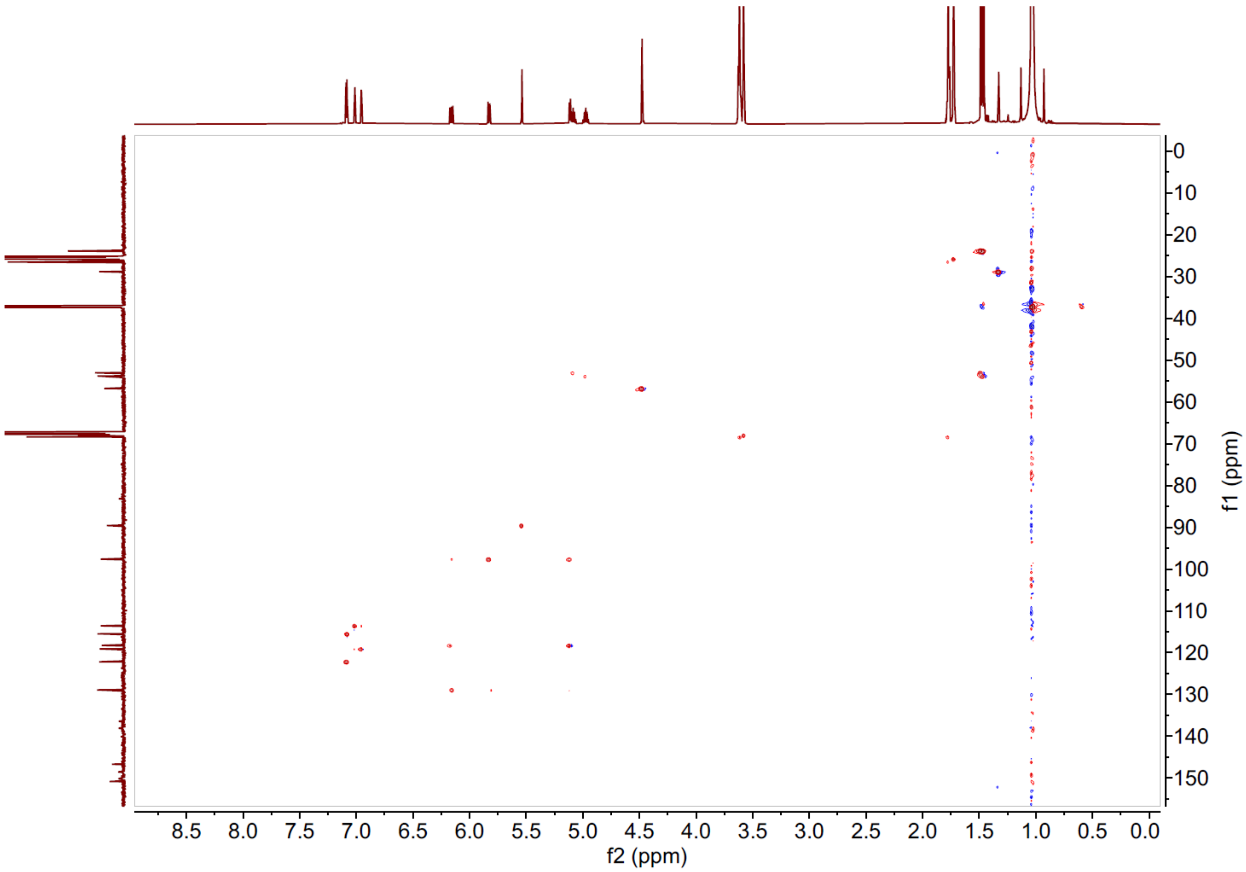

Figure S47. ^{1}H-^{13}C HSQC spectrum of *[Cu(I^{i}Pr$^{C\wedge N\wedge C}$)*]*, [Cu-**L1***] in THF-*d_8*

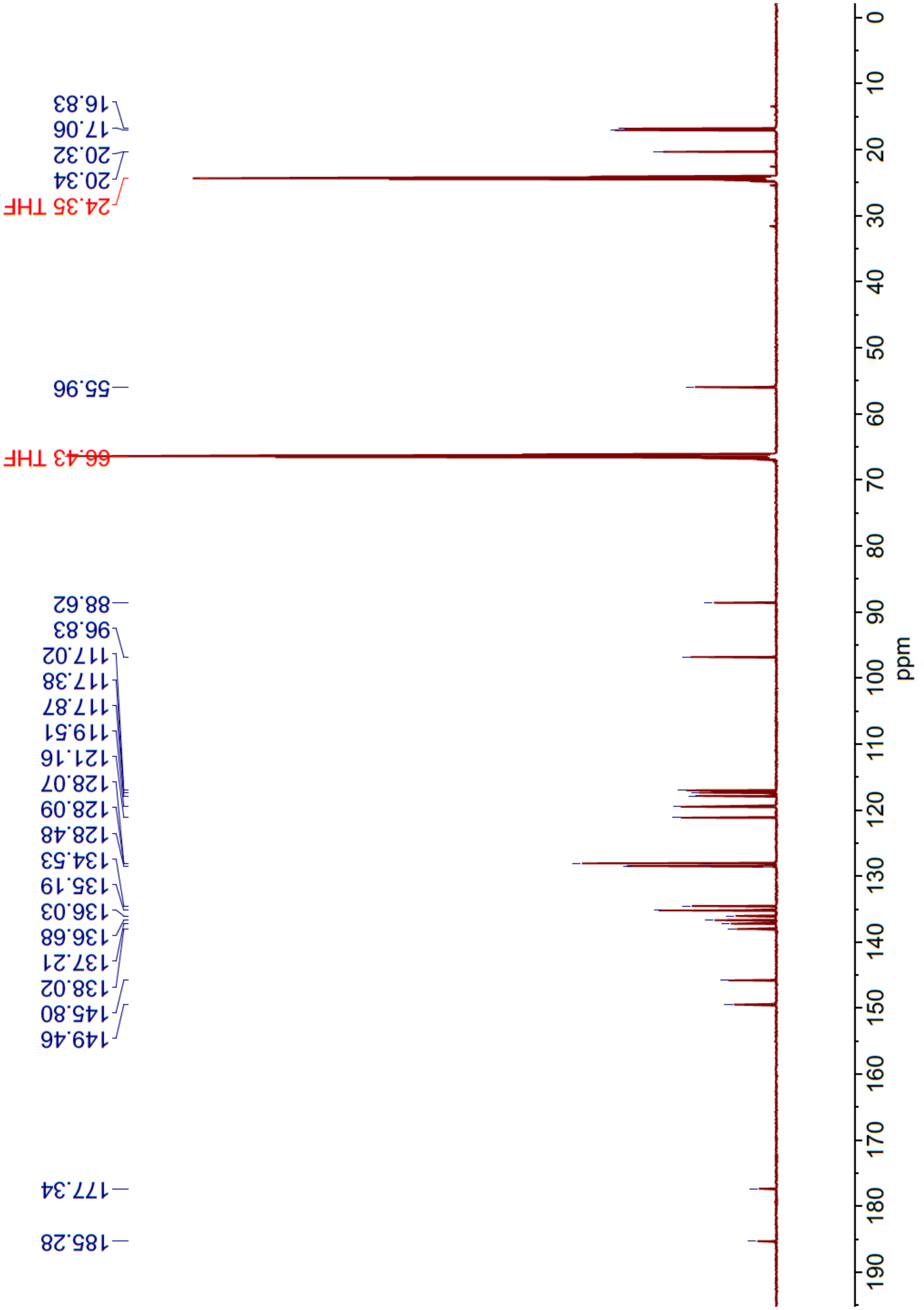

Figure S48. ^{13}C-NMR spectrum of *[Cu(IMes$^{C\wedge N\wedge C}$)*]*, [Cu-**L3***] in THF-d_8 (150 MHz)

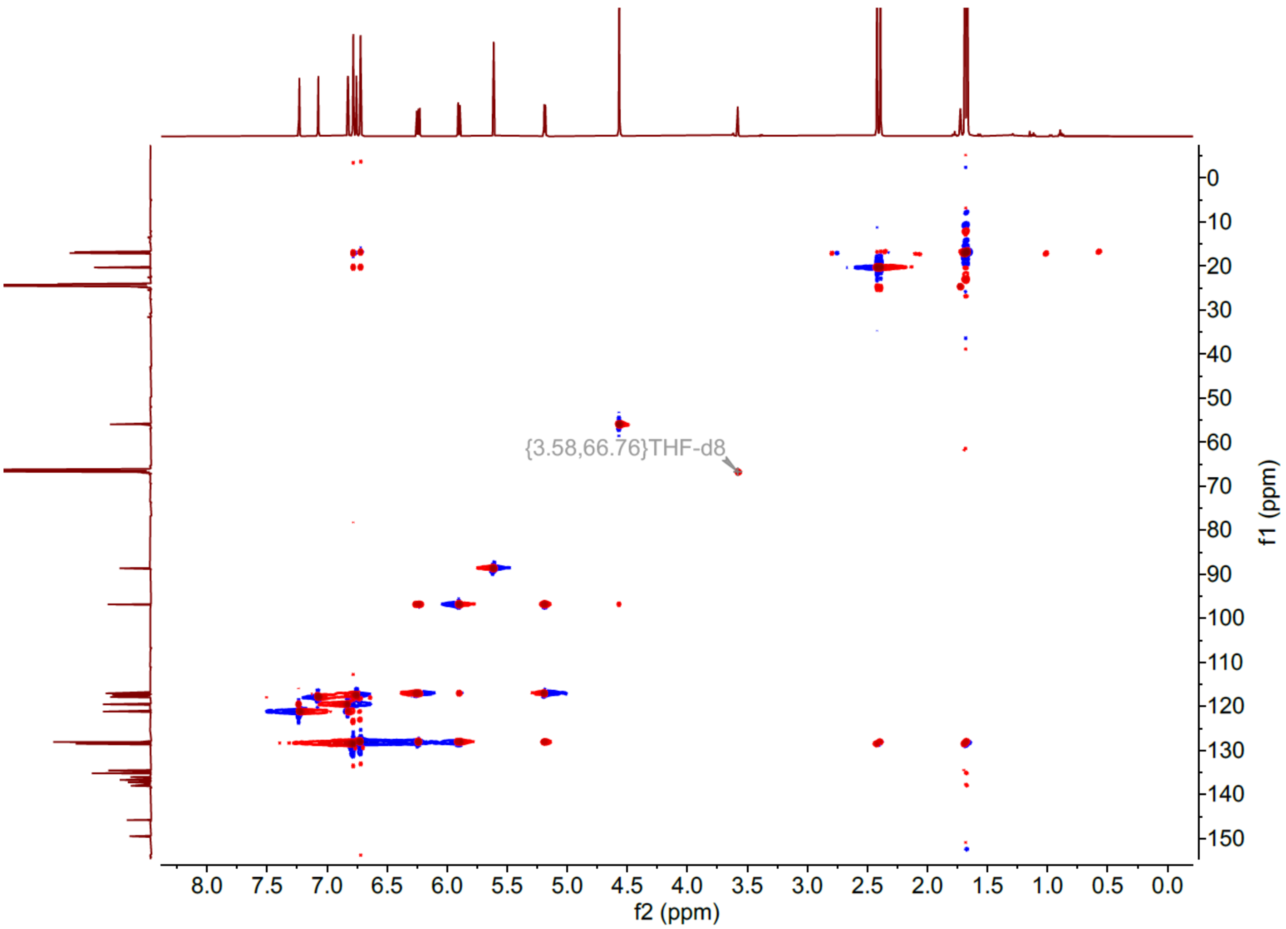

Figure S49. ^{1}H-^{13}C HSQC spectrum of *[Cu(IMes$^{C\wedge N\wedge C}$)*]*, [Cu-**L3***] in THF-*d*$_8$

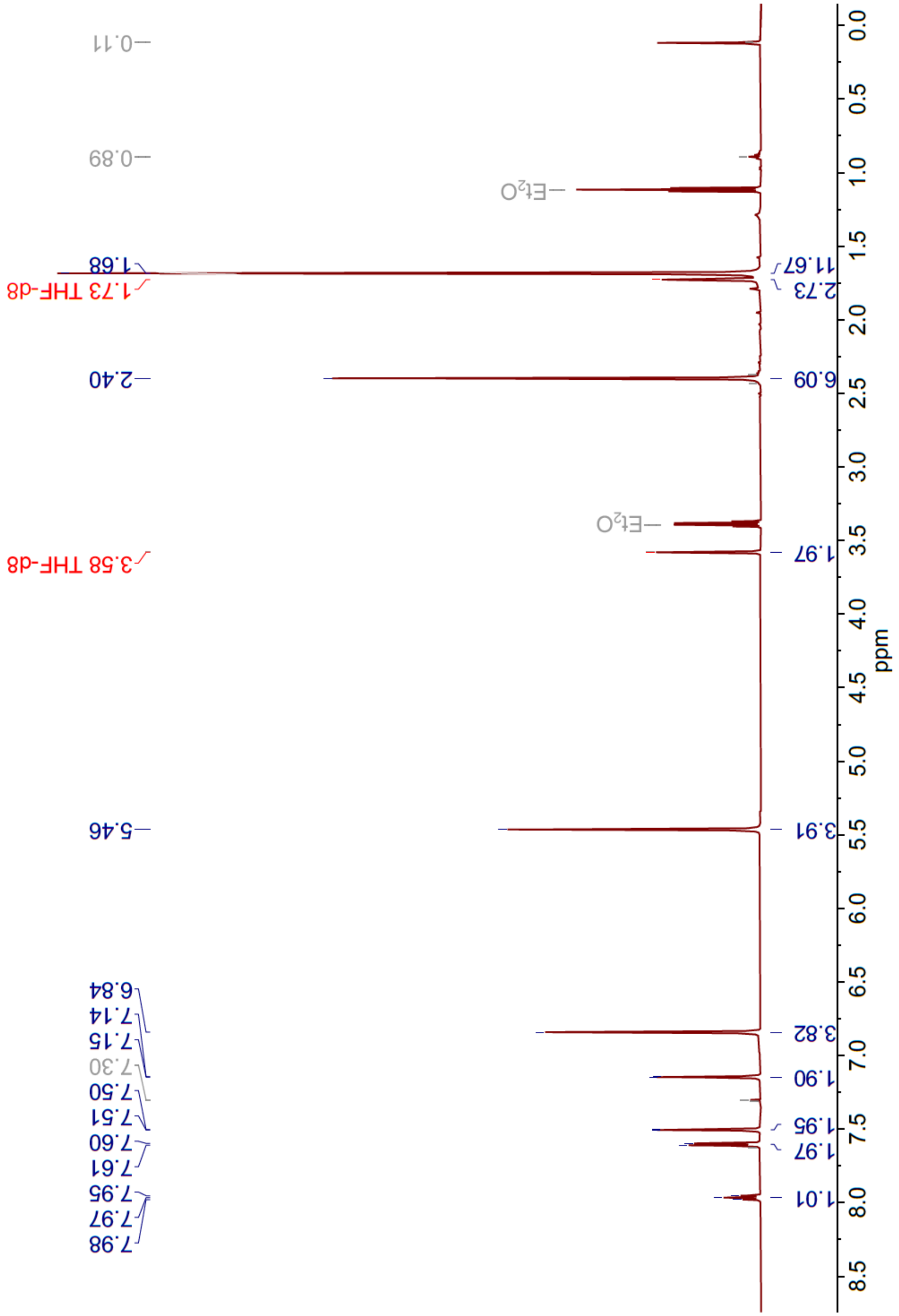

Figure S50. ^{1}H-NMR spectrum of [*Cu(IMes$^{C^\wedge N^\wedge C}$)]BArF*, [Cu-**L3**]BArF in THF-d_8 (600 MHz)

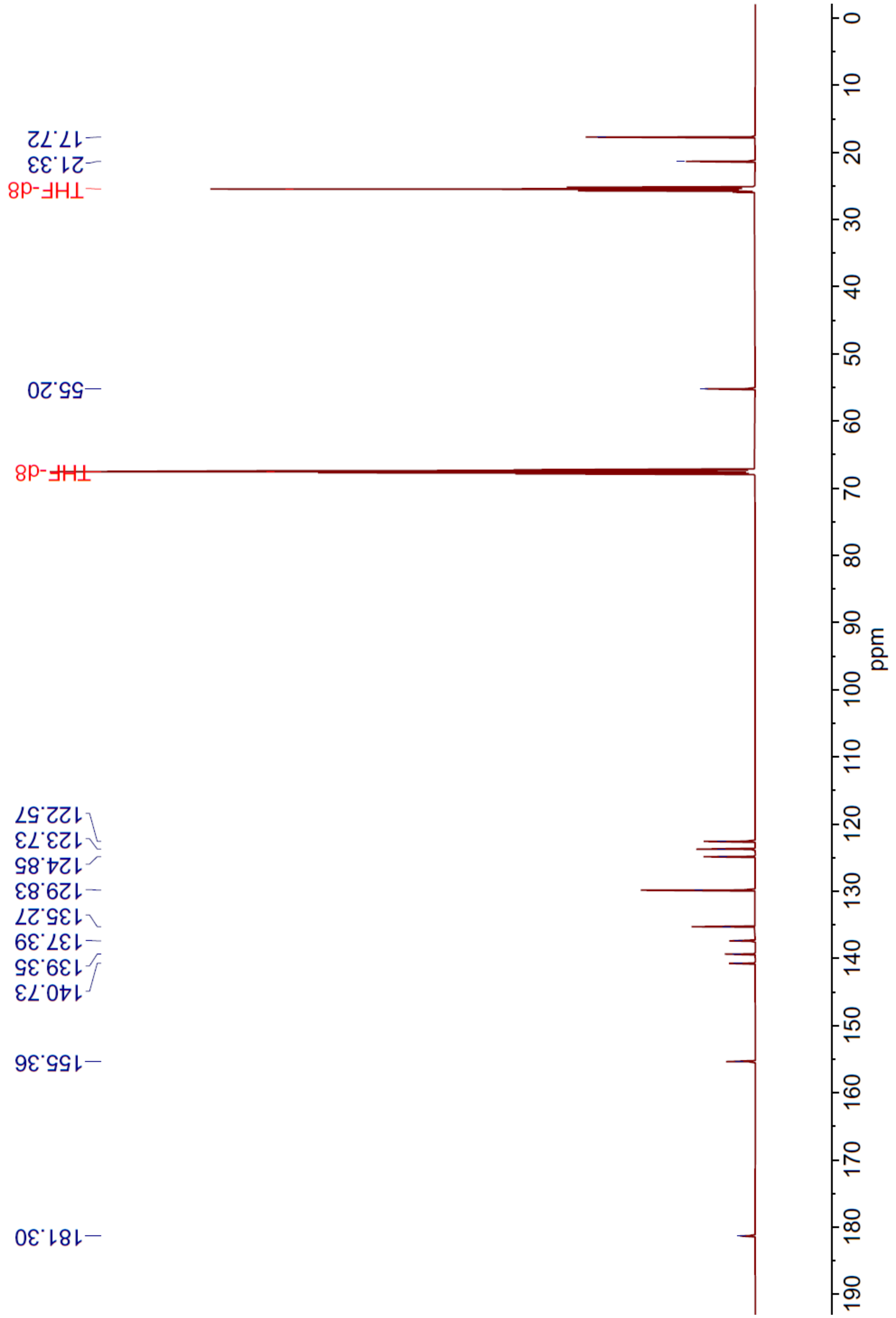

Figure S51. ^{13}C-NMR spectrum of [*Cu(IMes$^{C\wedge N\wedge C}$)]BArF*, [Cu-**L3**]BArF in THF-d_8 (150 MHz)

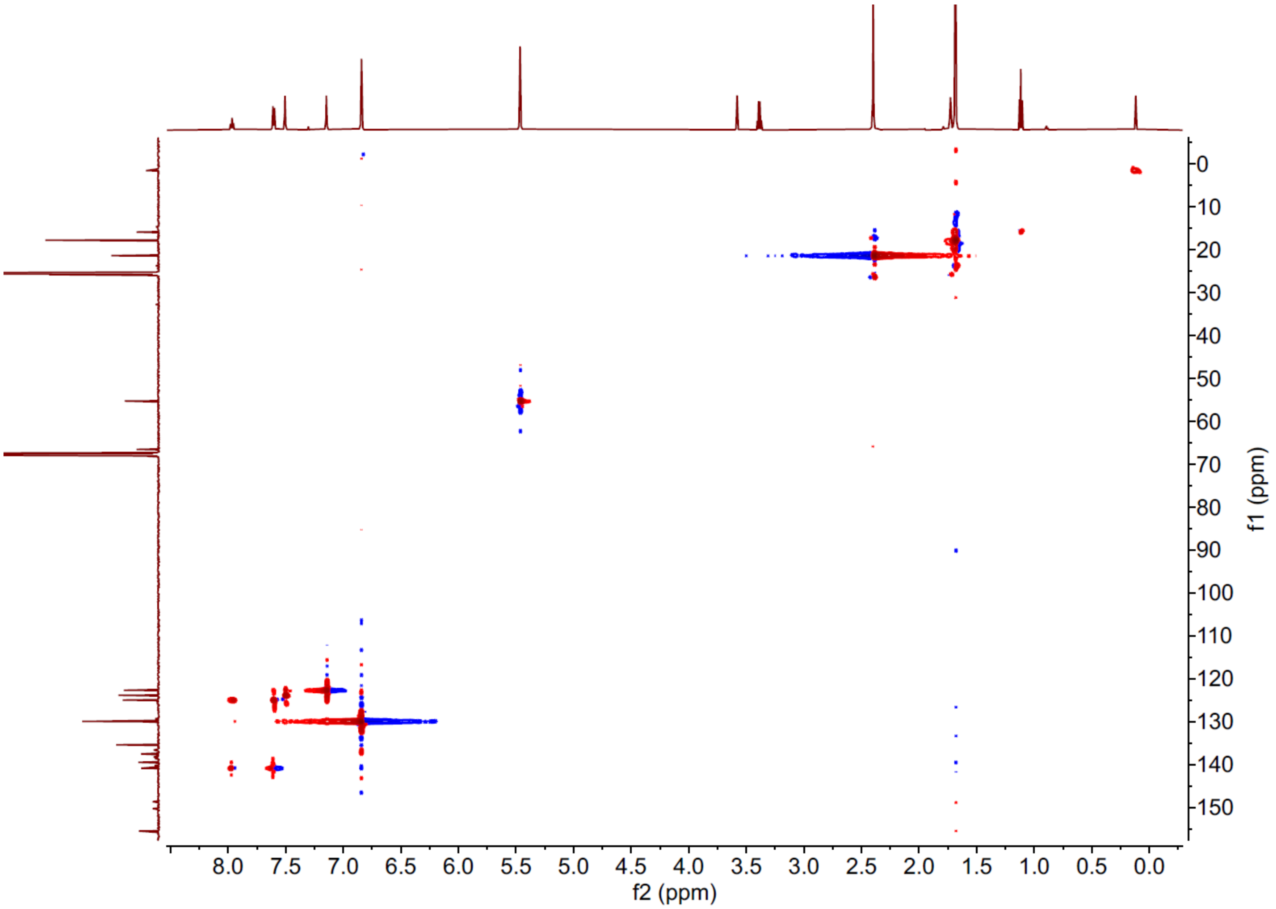

Figure S52. ^{1}H-^{13}C HSQC spectrum of [*Cu(IMes$^{C\wedge N\wedge C}$)]BArF*, [Cu-**L3**]BArF in THF-*d$_8$*

^{1}H NMR and ^{13}C NMR spectroscopy data of propiolate ester products

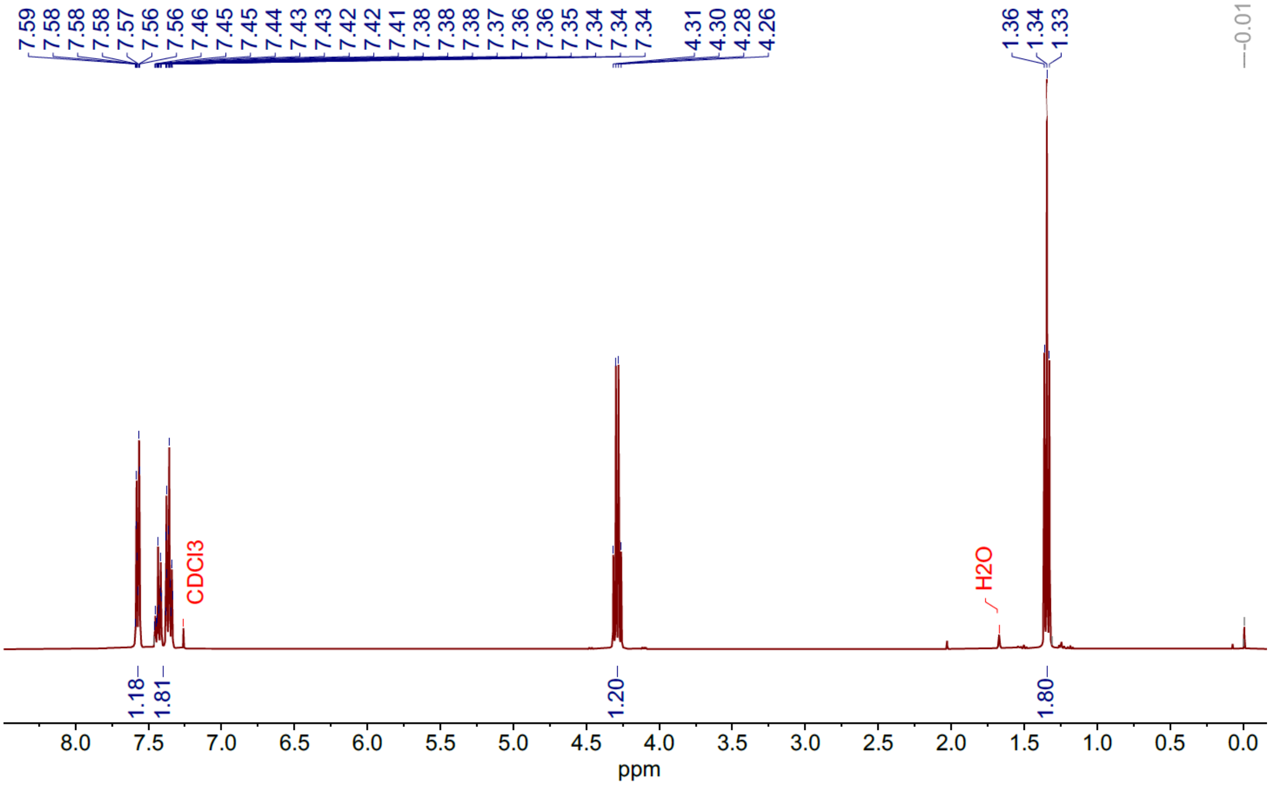

Figure S53. ^{1}H-NMR spectrum of *ethyl 3-phenylpropiolate* in $CDCl_3$ (400 MHz)

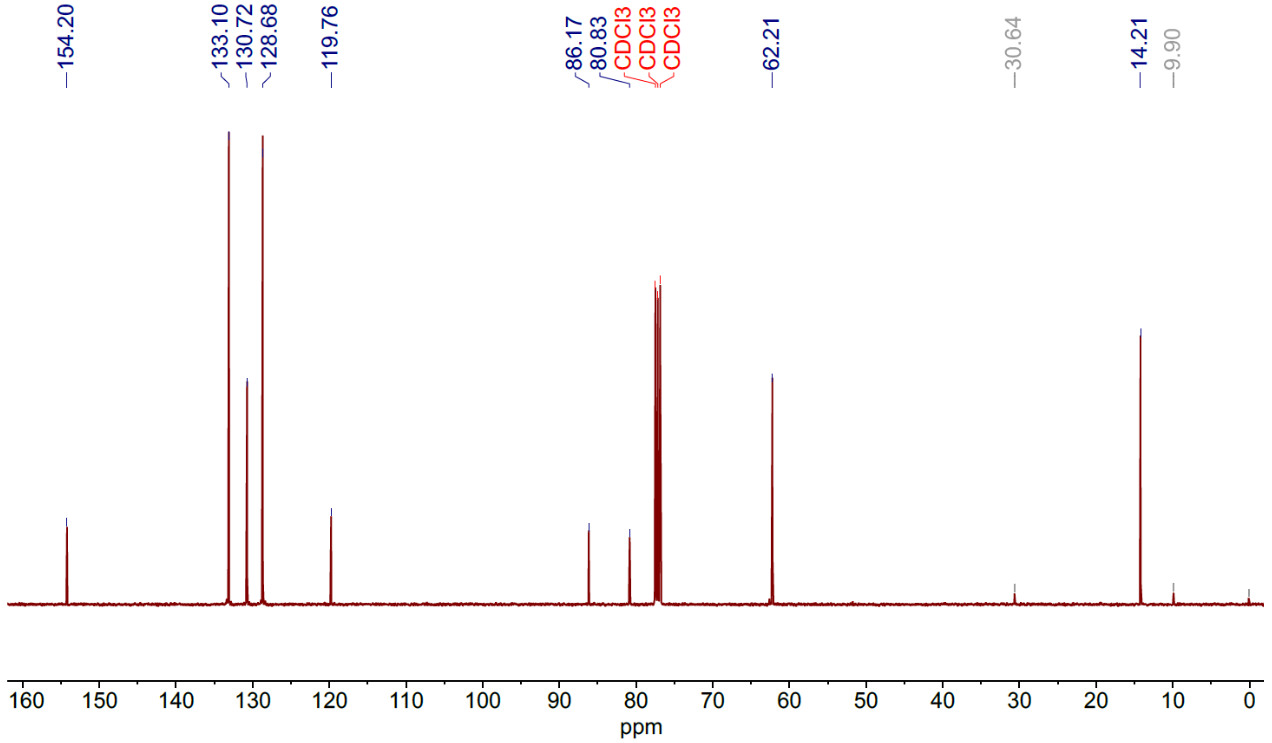

Figure S54. ^{13}C-NMR spectrum of *ethyl 3-phenylpropiolate* in $CDCl_3$ (101 MHz)

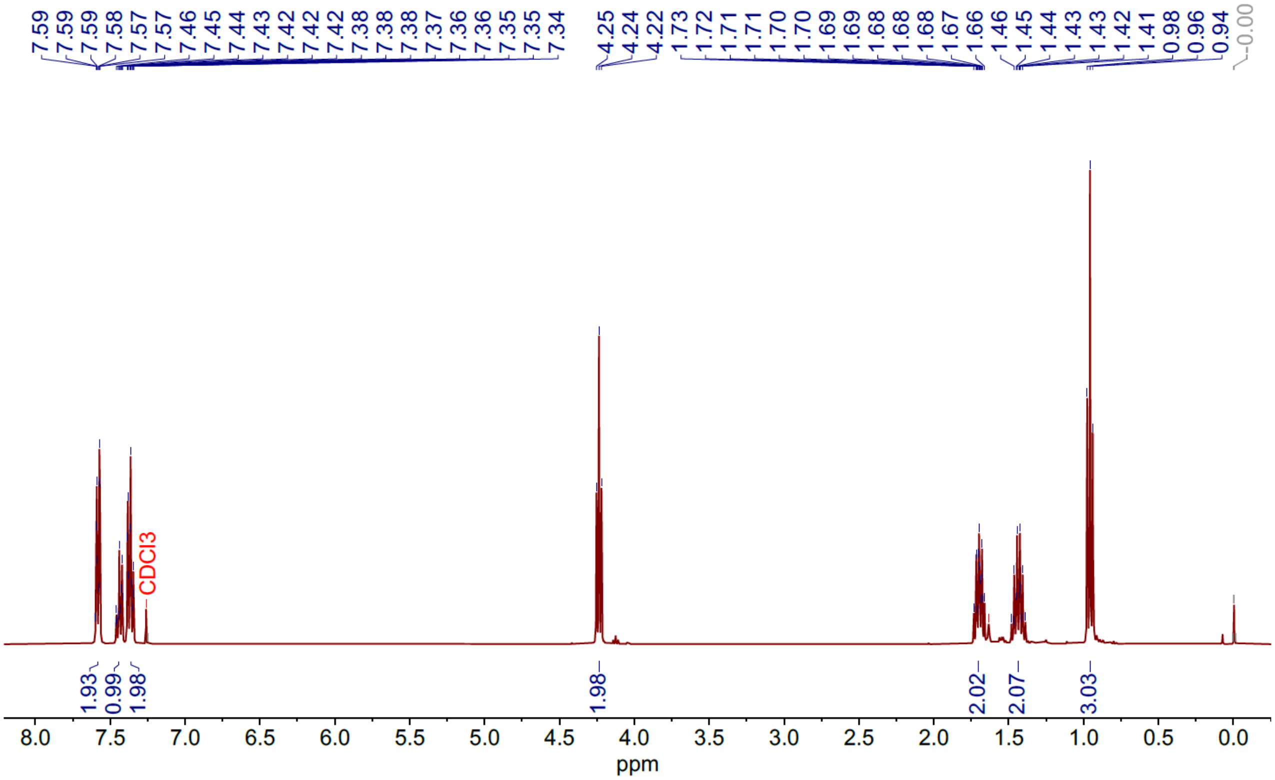

Figure S55. ^{1}H-NMR spectrum of *butyl 3-phenylpropiolate* in $CDCl_3$ (400 MHz)

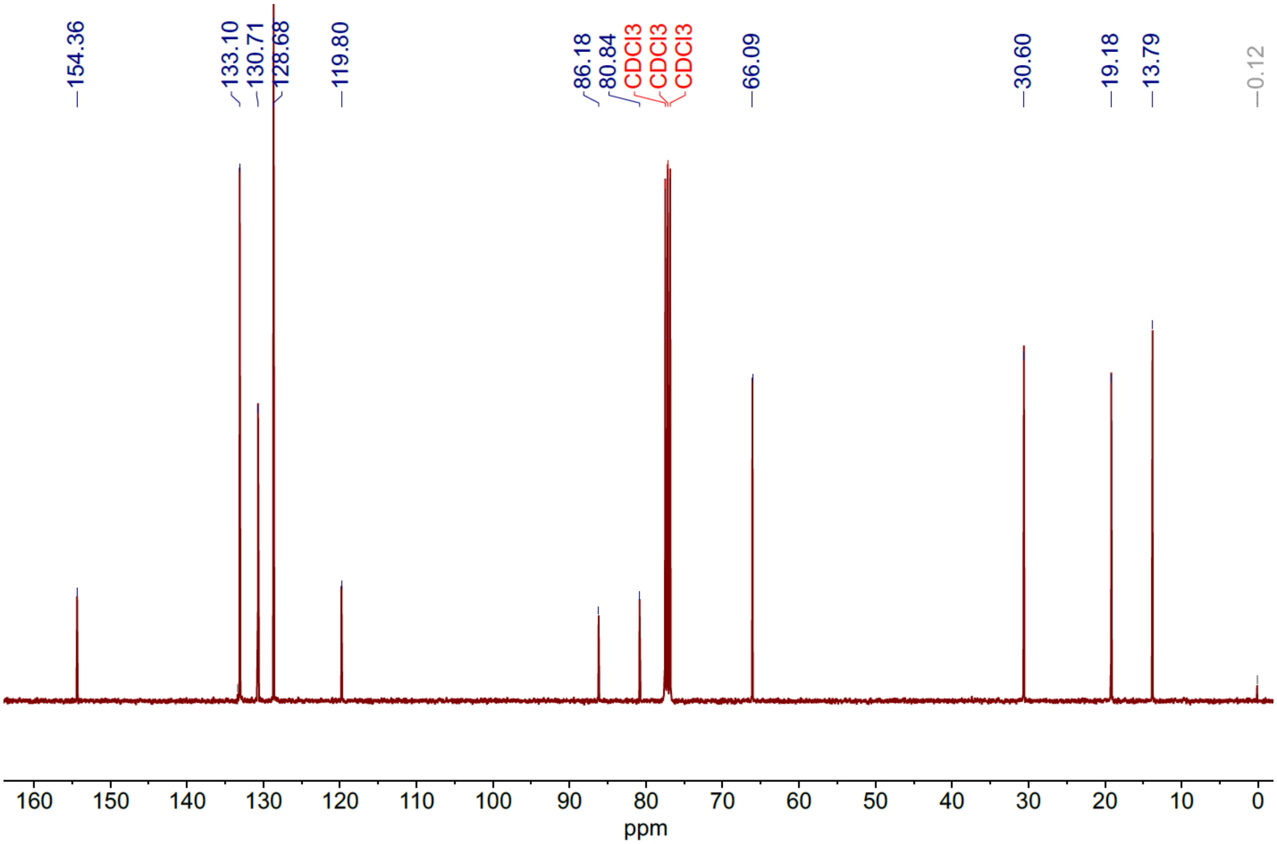

Figure S56. ^{13}C-NMR spectrum of *butyl 3-phenylpropiolate* in $CDCl_3$ (101 MHz)

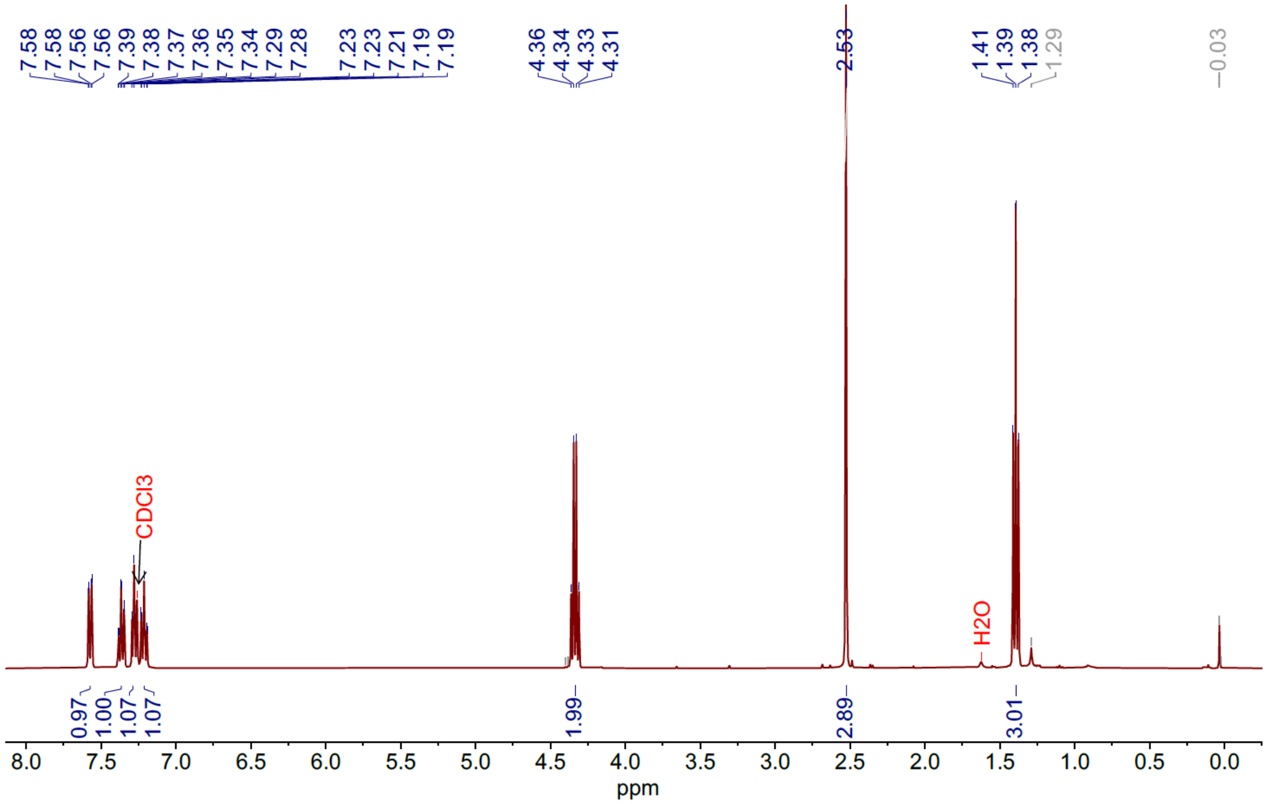

Figure S57. ^{1}H-NMR spectrum of *ethyl 3-(o-tolyl)propiolate* in $CDCl_3$ (400 MHz)

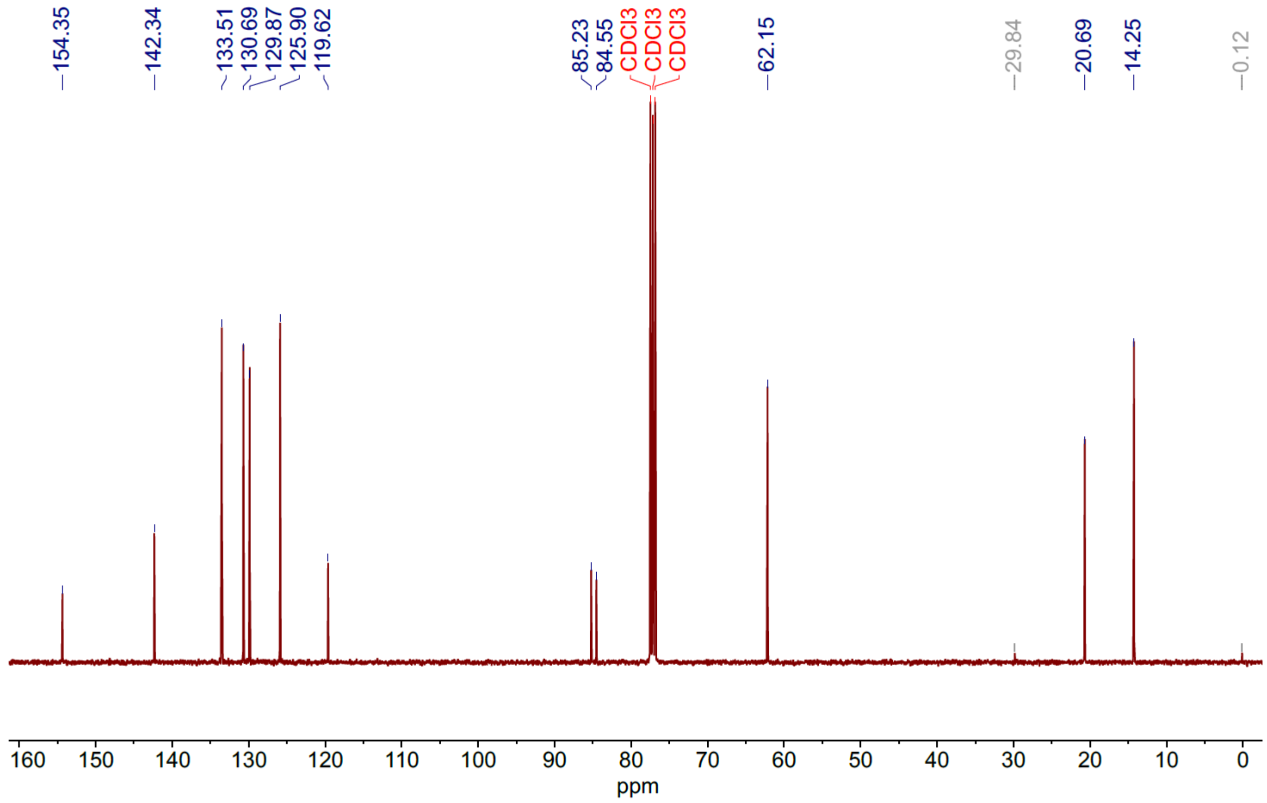

Figure S58. ^{13}C-NMR spectrum of *ethyl 3-(o-tolyl)propiolate* in $CDCl_3$ (101 MHz)

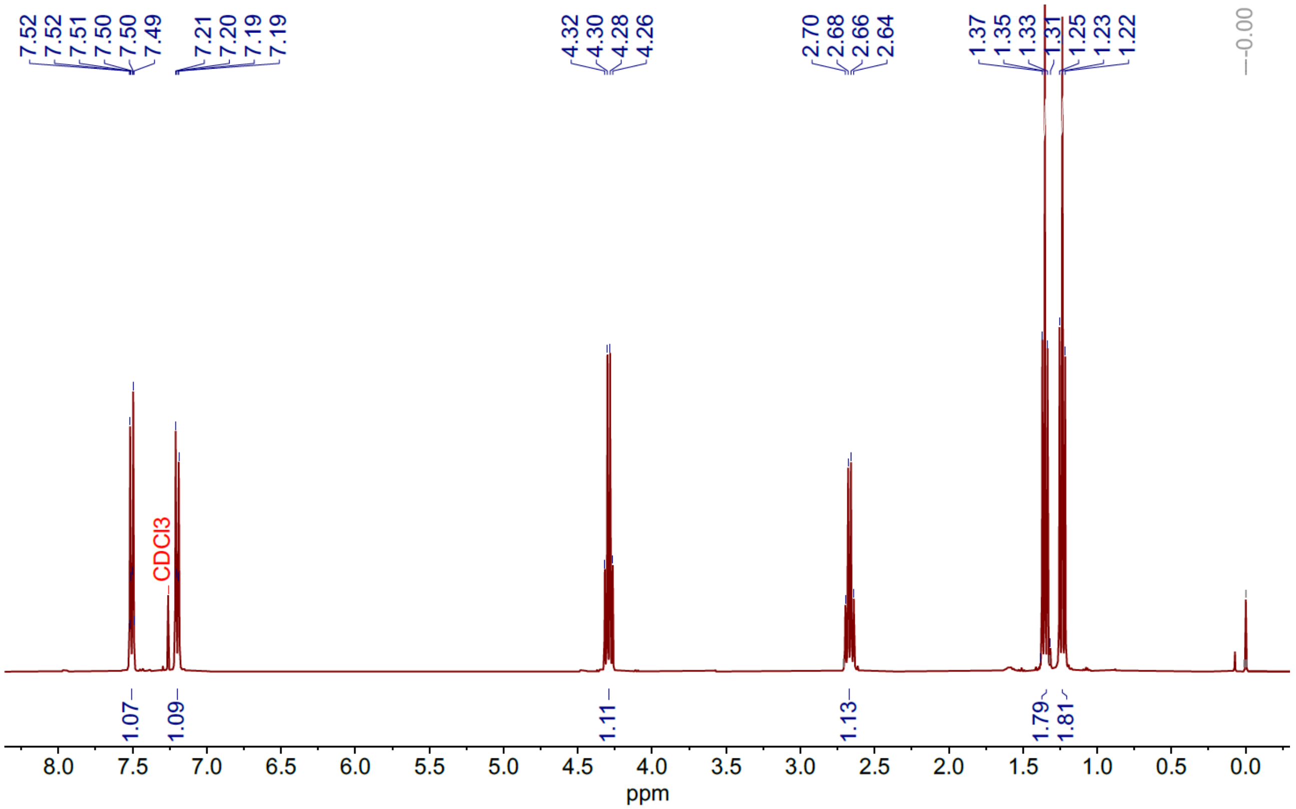

Figure S59. ^{1}H-NMR spectrum of *ethyl 3-(4-ethylphenyl)propiolate* in $CDCl_3$ (400 MHz)

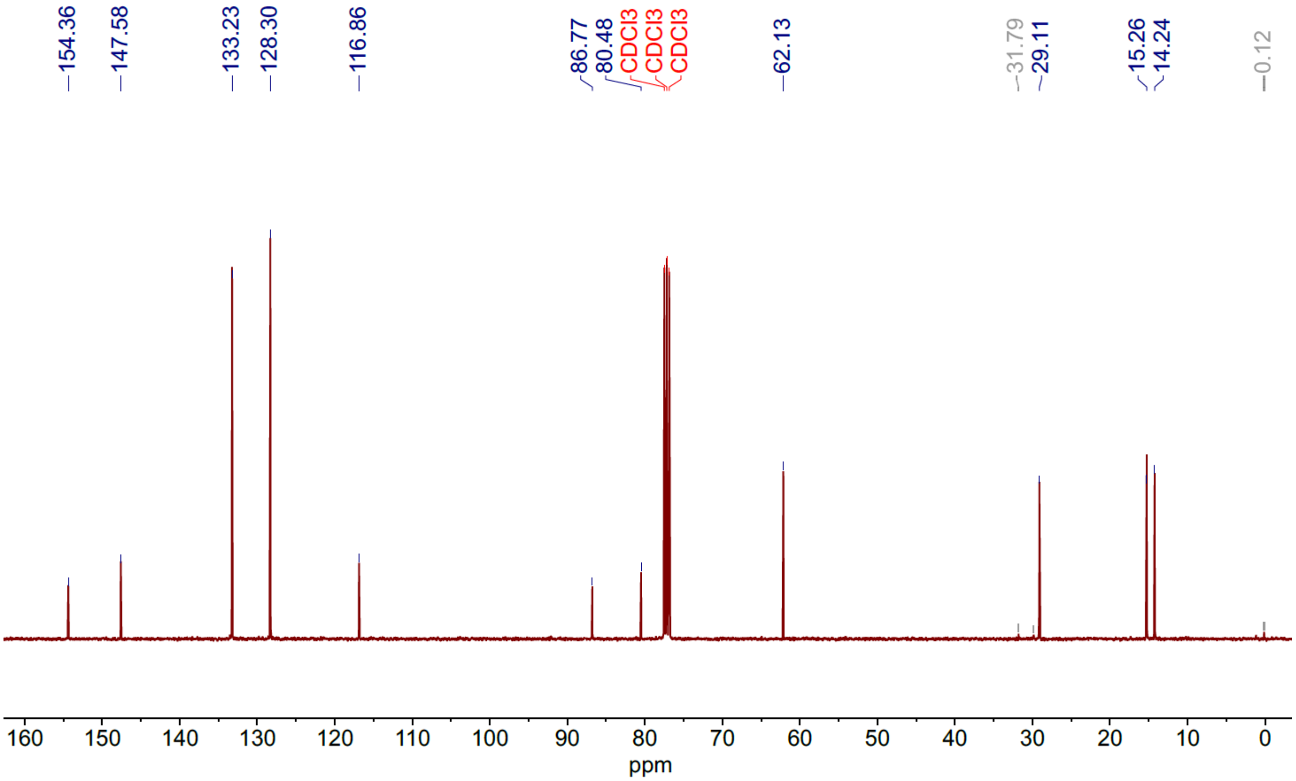

Figure S60. ^{13}C-NMR spectrum of *ethyl 3-(4-ethylphenyl)propiolate* in $CDCl_3$ (101 MHz)

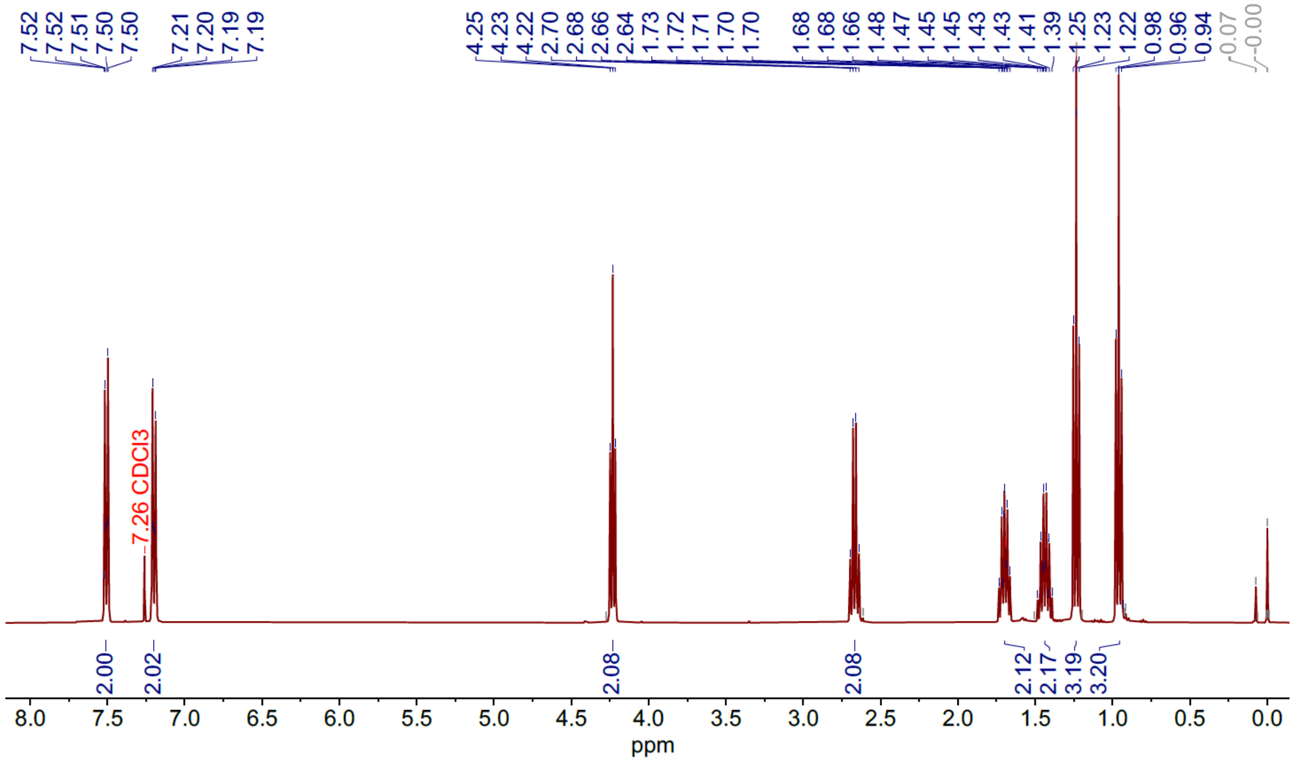

Figure S61. ^{1}H-NMR spectrum of *butyl 3-(4-ethylphenyl)propiolate* in $CDCl_3$ (400 MHz)

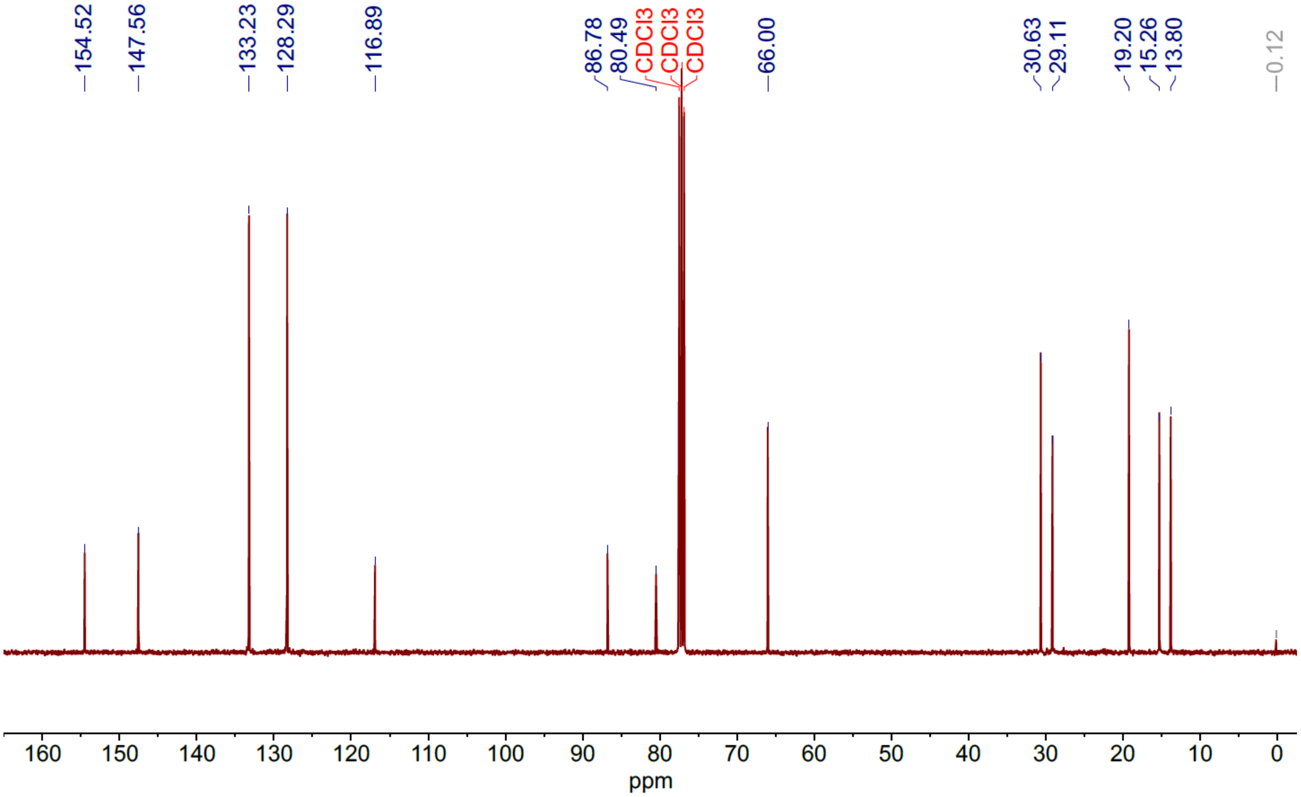

Figure S62. ^{13}C-NMR spectrum of *butyl 3-(4-ethylphenyl)propiolate* in $CDCl_3$ (101 MHz)

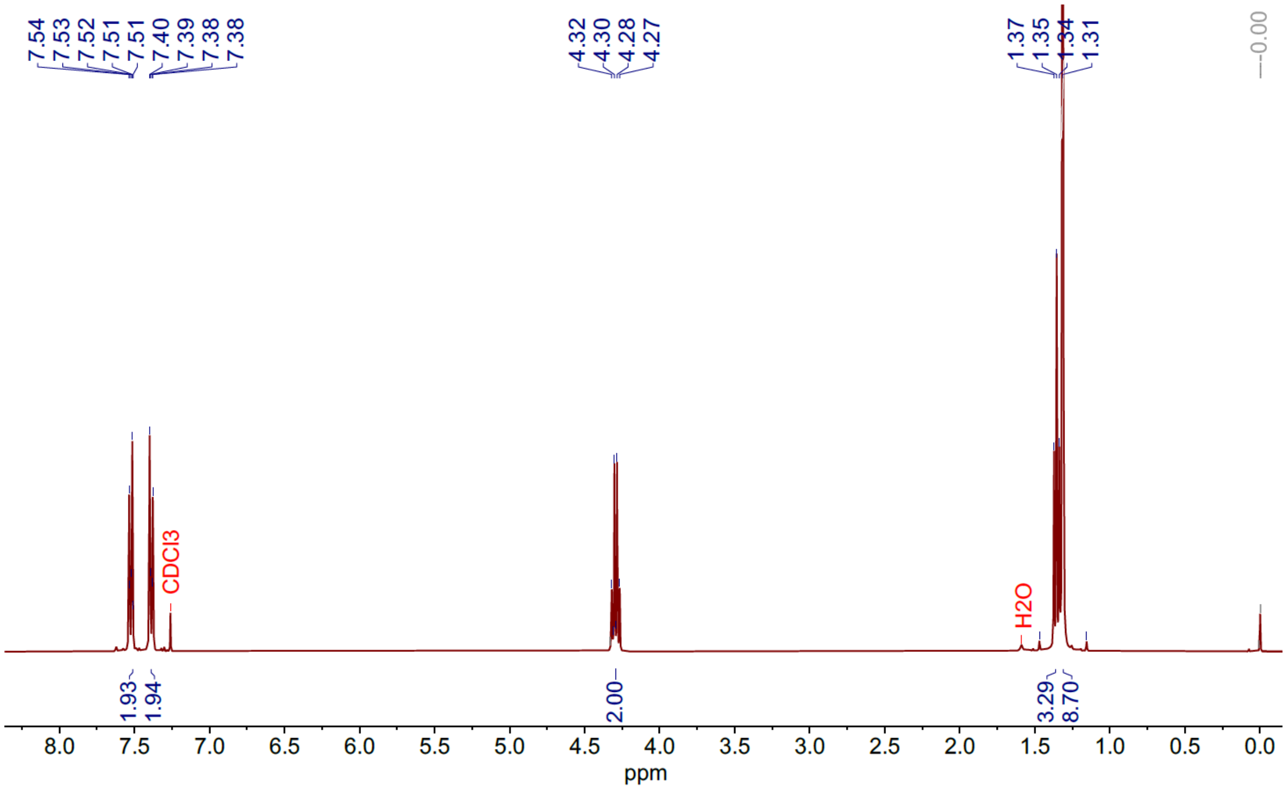

Figure S63. ^{1}H-NMR spectrum of *ethyl 3-(4-(tert-butyl)phenyl)propiolate* in $CDCl_3$ (400 MHz)

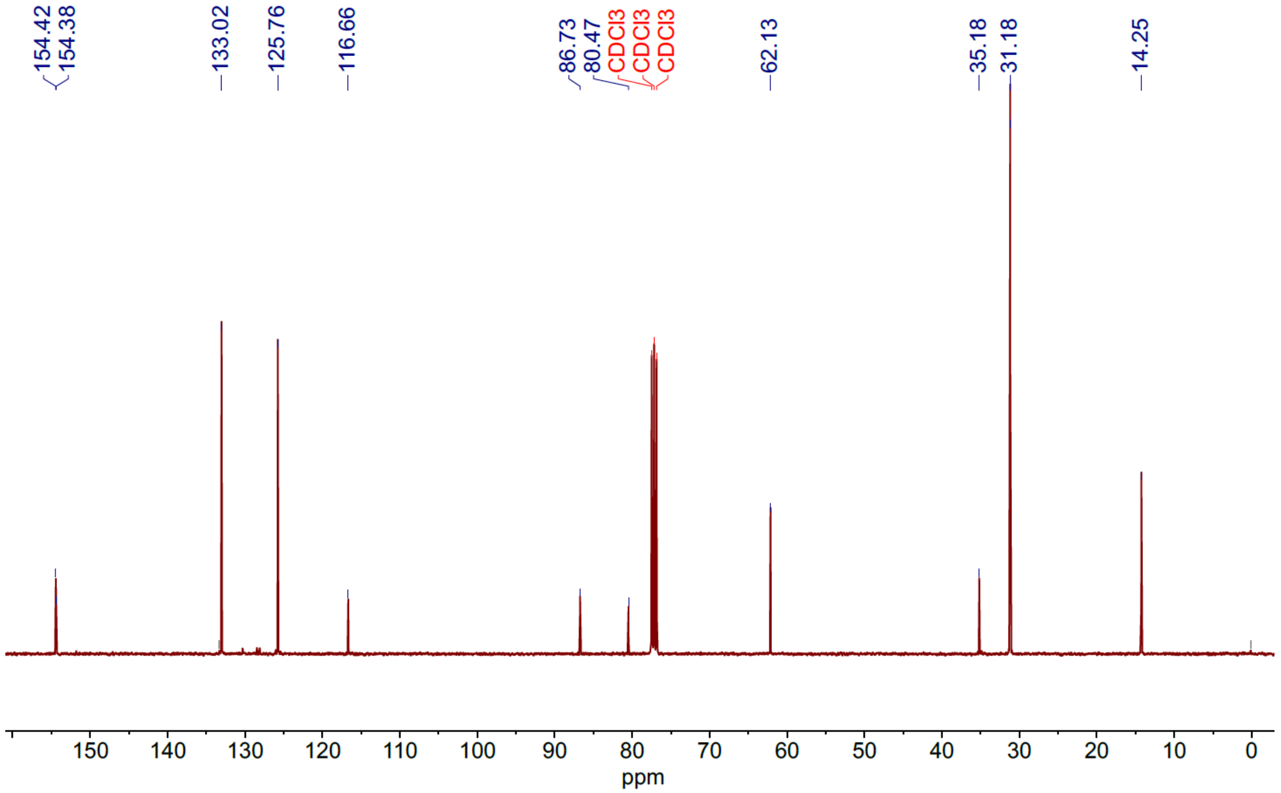

Figure S64. ^{13}C-NMR spectrum of *ethyl 3-(4-(tert-butyl)phenyl)propiolate* in $CDCl_3$ (101 MHz)

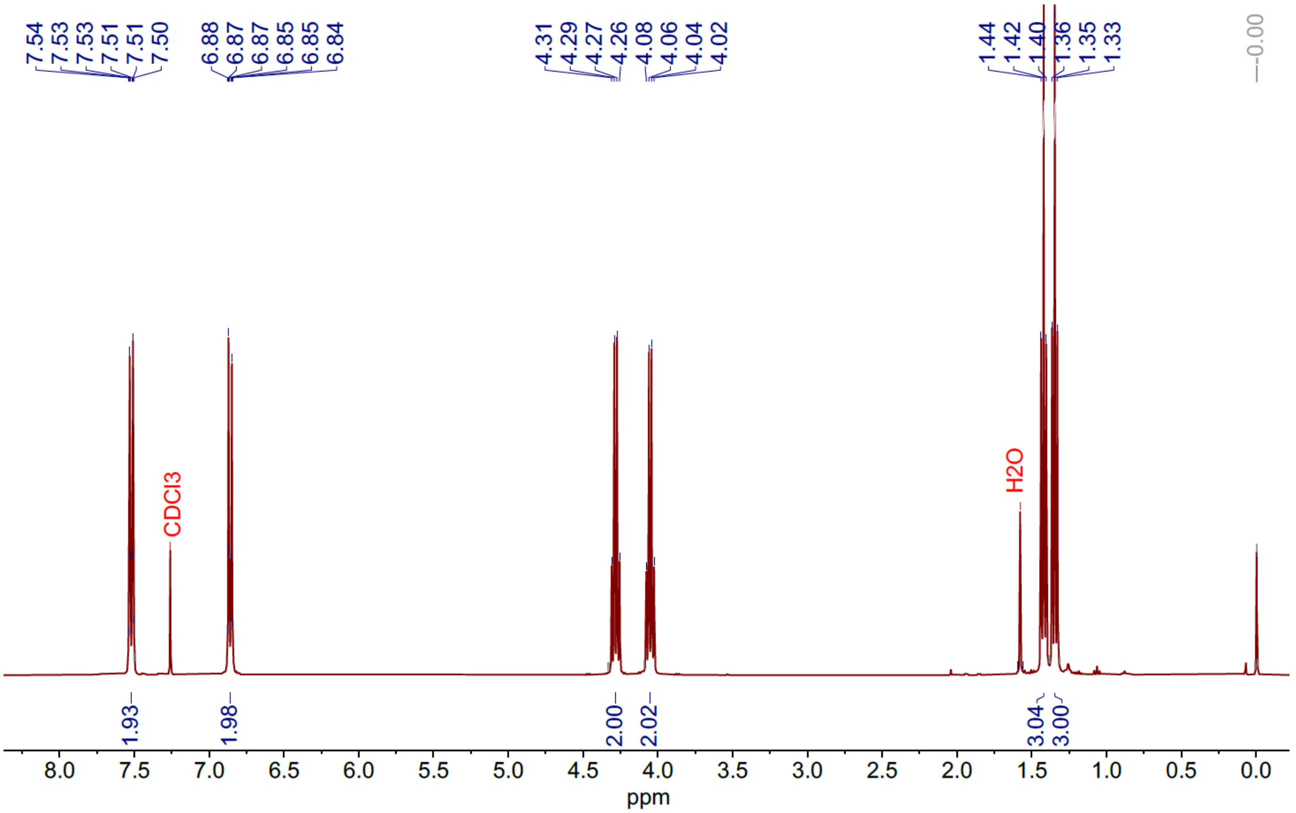

Figure S65. ^{1}H-NMR spectrum of *ethyl 3-(4-ethoxyphenyl)propiolate* in $CDCl_3$ (400 MHz)

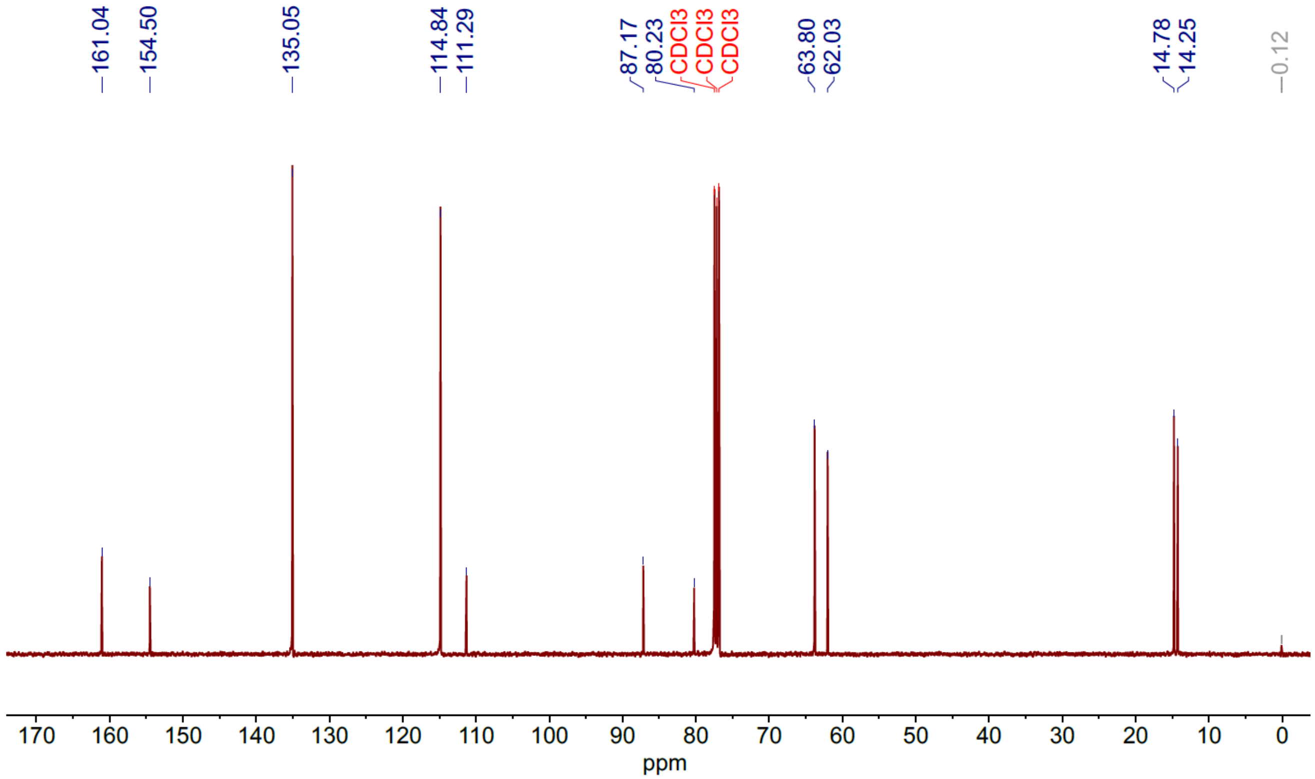

Figure S66. ^{13}C-NMR spectrum of *ethyl 3-(4-ethoxyphenyl)propiolate* in $CDCl_3$ (101 MHz)

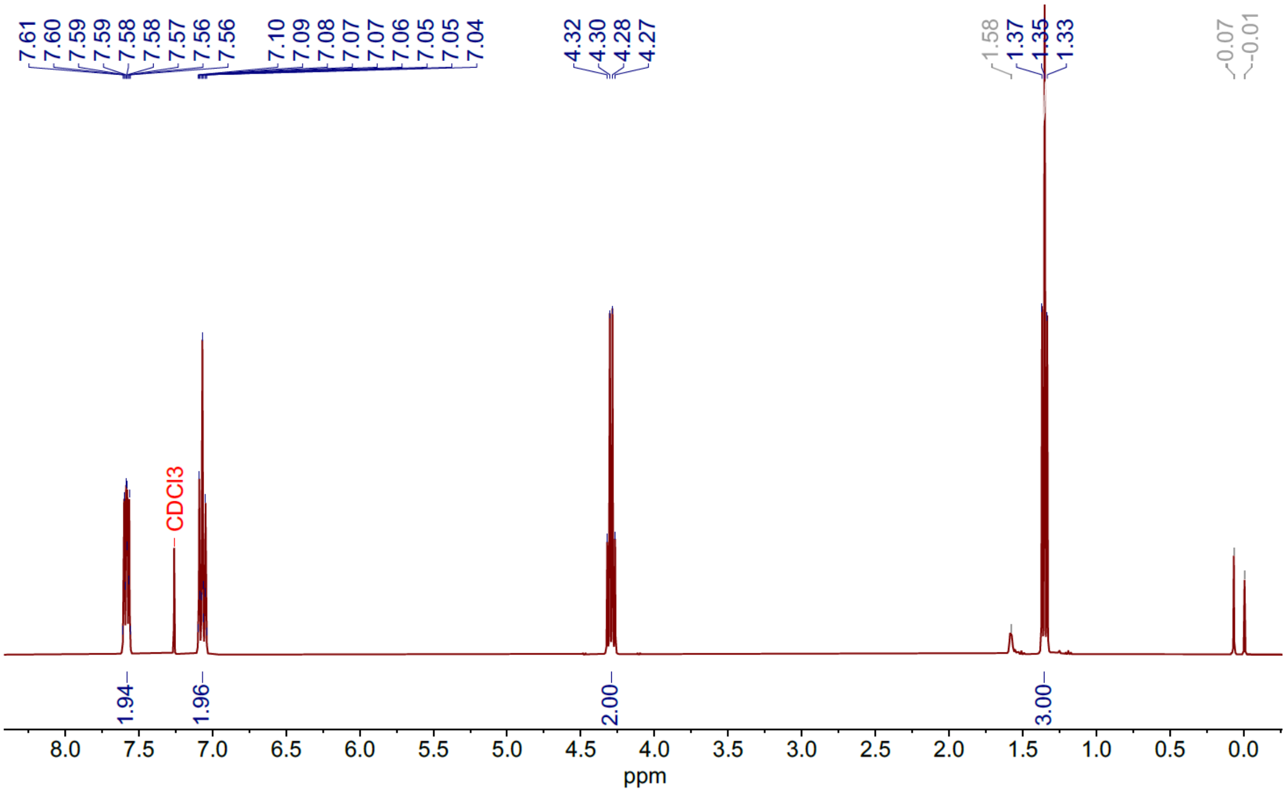

Figure S67. ^{1}H-NMR spectrum of *ethyl 3-(4-fluorophenyl)propiolate* in $CDCl_3$ (400 MHz)

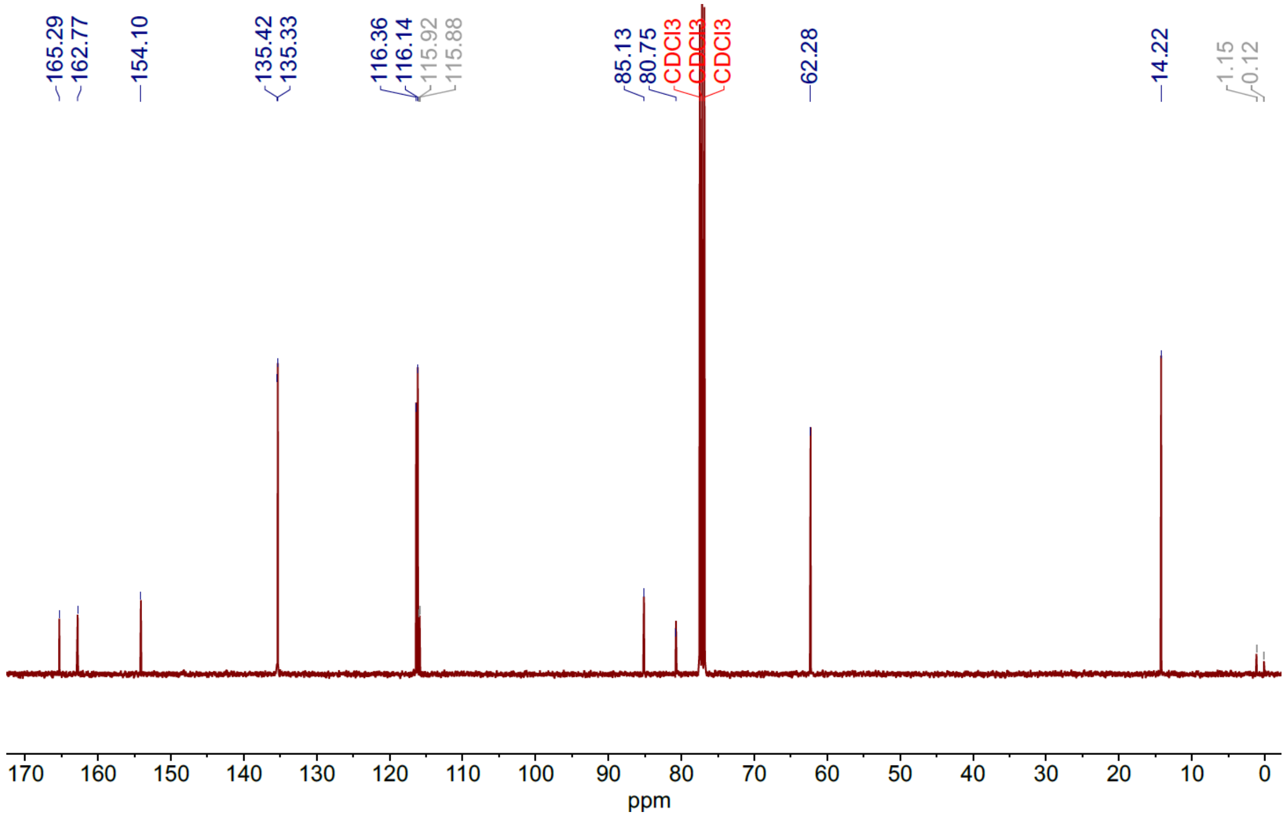

Figure S68. ^{13}C-NMR spectrum of *ethyl 3-(4-fluorophenyl)propiolate* in $CDCl_3$ (101 MHz)

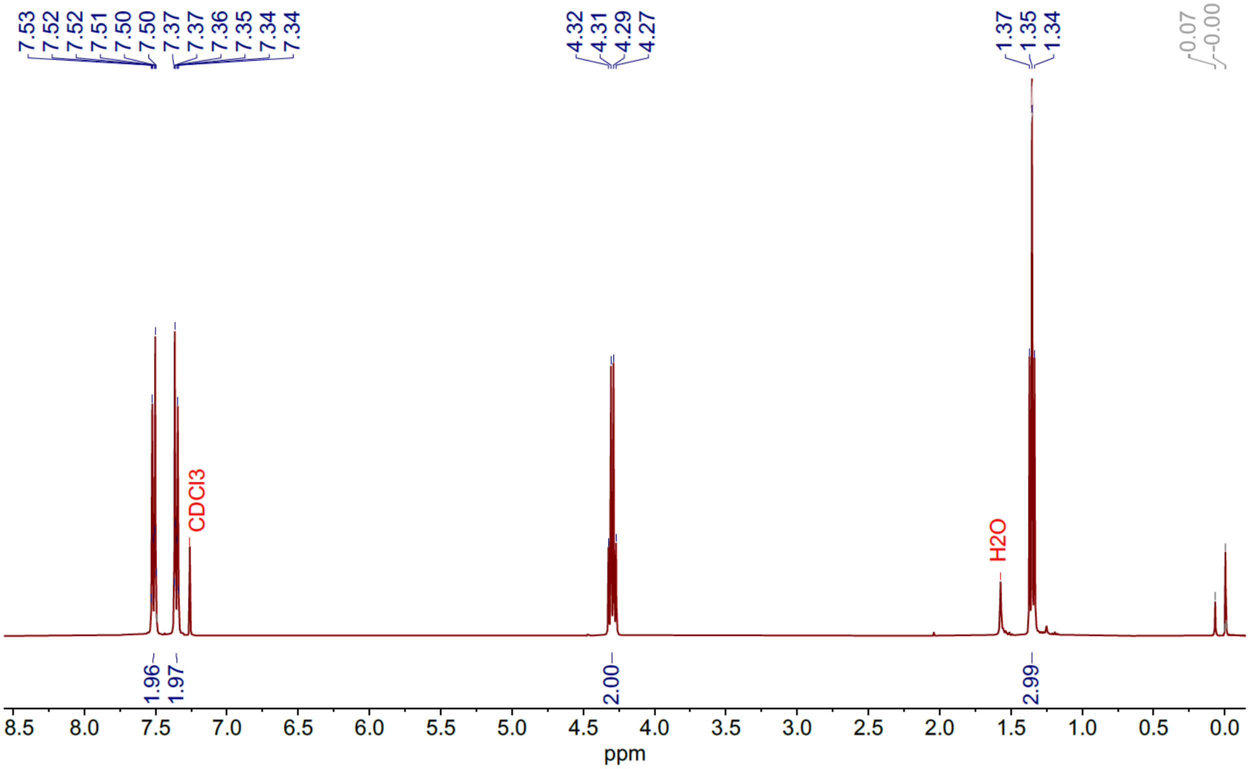

Figure S69. ^{1}H-NMR spectrum of *ethyl 3-(4-chlorophenyl)propiolate* in $CDCl_3$ (400 MHz)

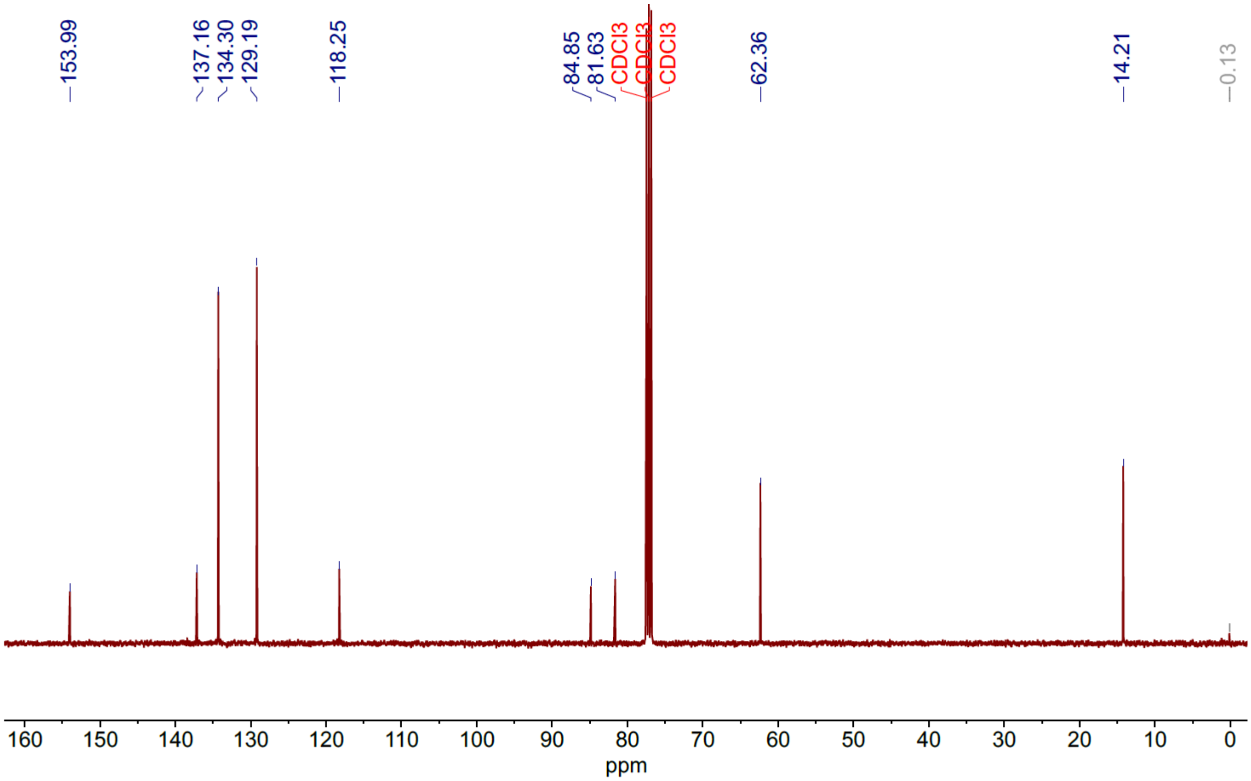

Figure S70. ^{13}C-NMR spectrum of *ethyl 3-(4-chlorophenyl)propiolate* in $CDCl_3$ (101 MHz)

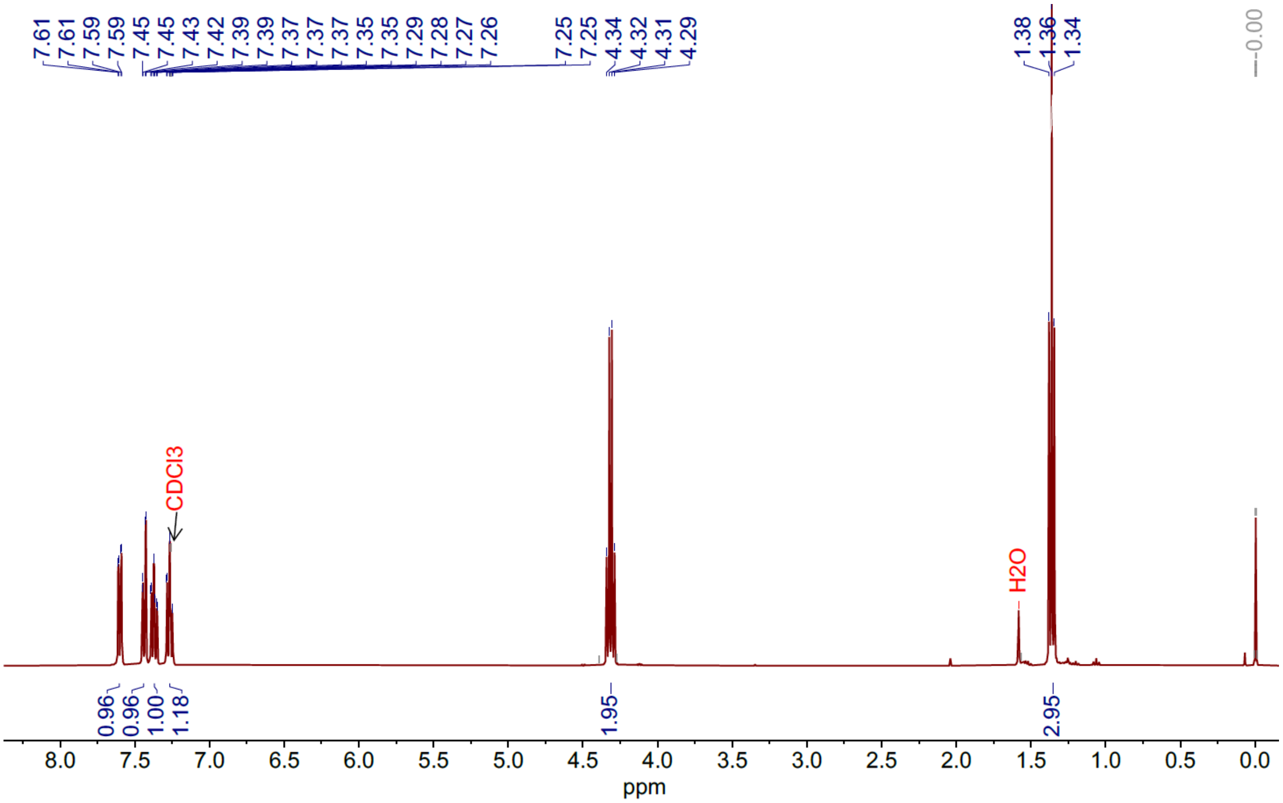

Figure S71. ^{1}H-NMR spectrum of *ethyl 3-(2-chlorophenyl)propiolate* in $CDCl_3$ (400 MHz)

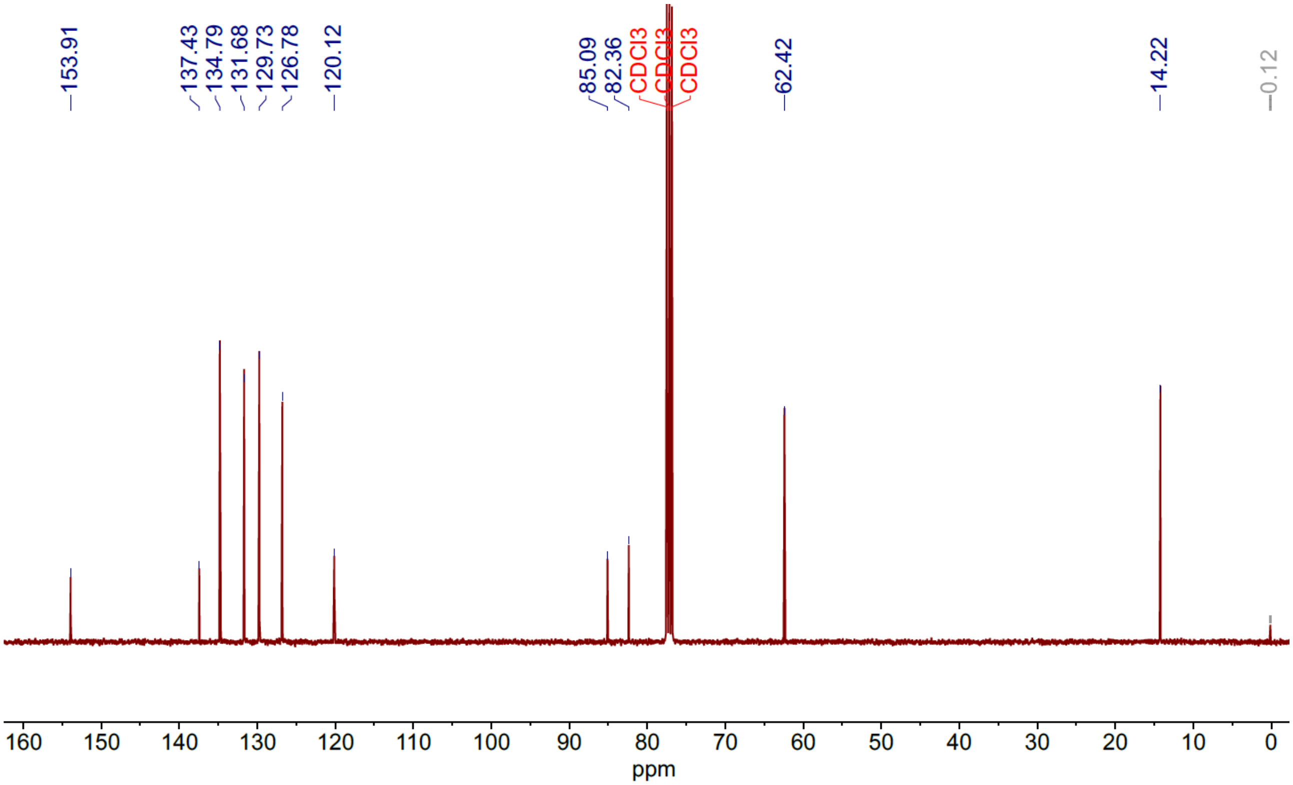

Figure S72. ^{13}C-NMR spectrum of *ethyl 3-(2-chlorophenyl)propiolate* in $CDCl_3$ (101 MHz)

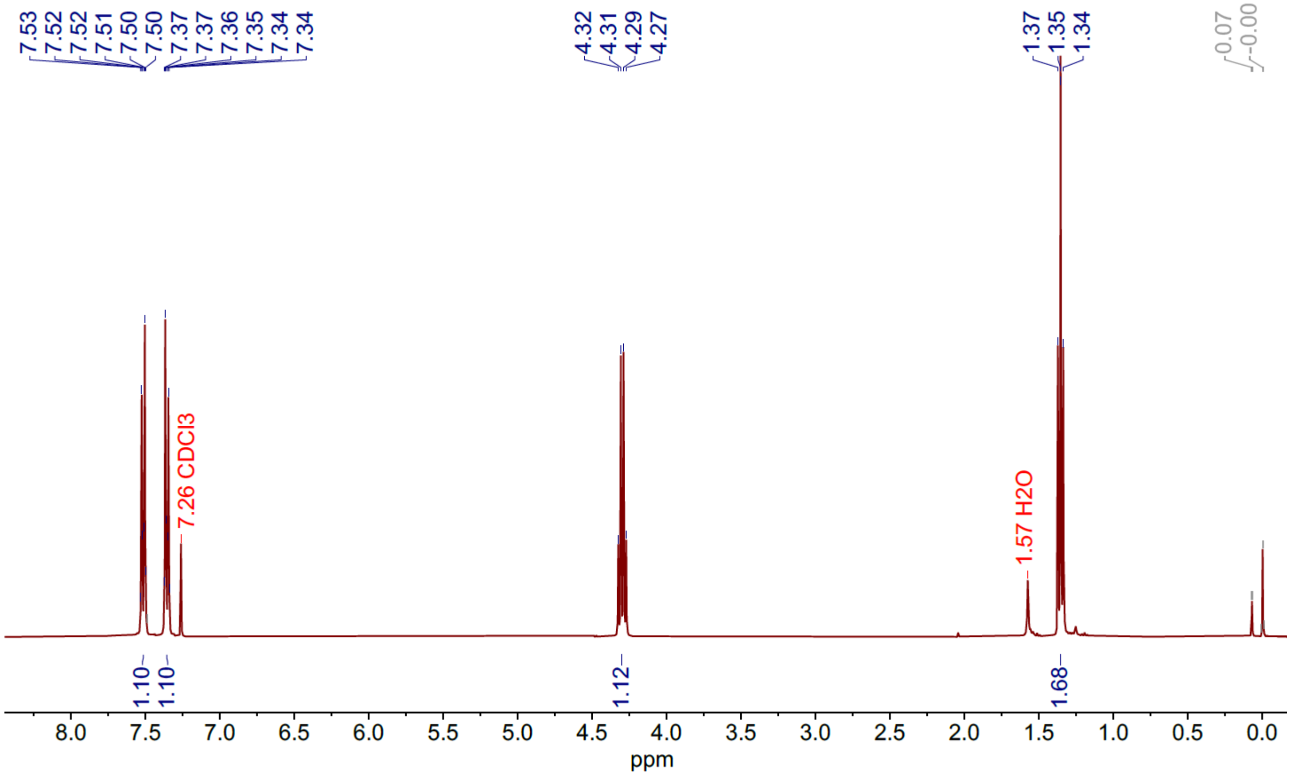

Figure S73. ^{1}H-NMR spectrum of *ethyl 3-(4-bromophenyl)propiolate* in $CDCl_3$ (400 MHz)

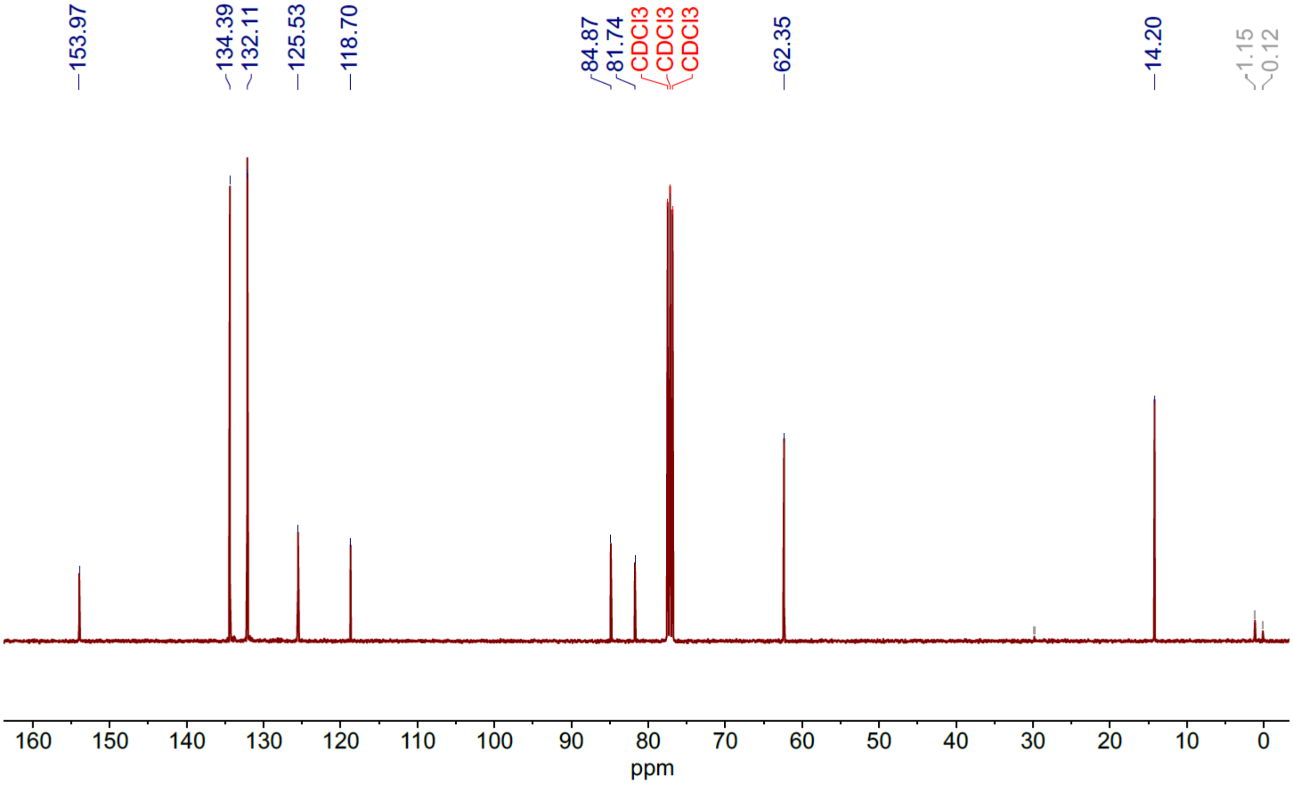

Figure S74. ^{13}C-NMR spectrum of *ethyl 3-(4-bromophenyl)propiolate* in $CDCl_3$ (101 MHz)

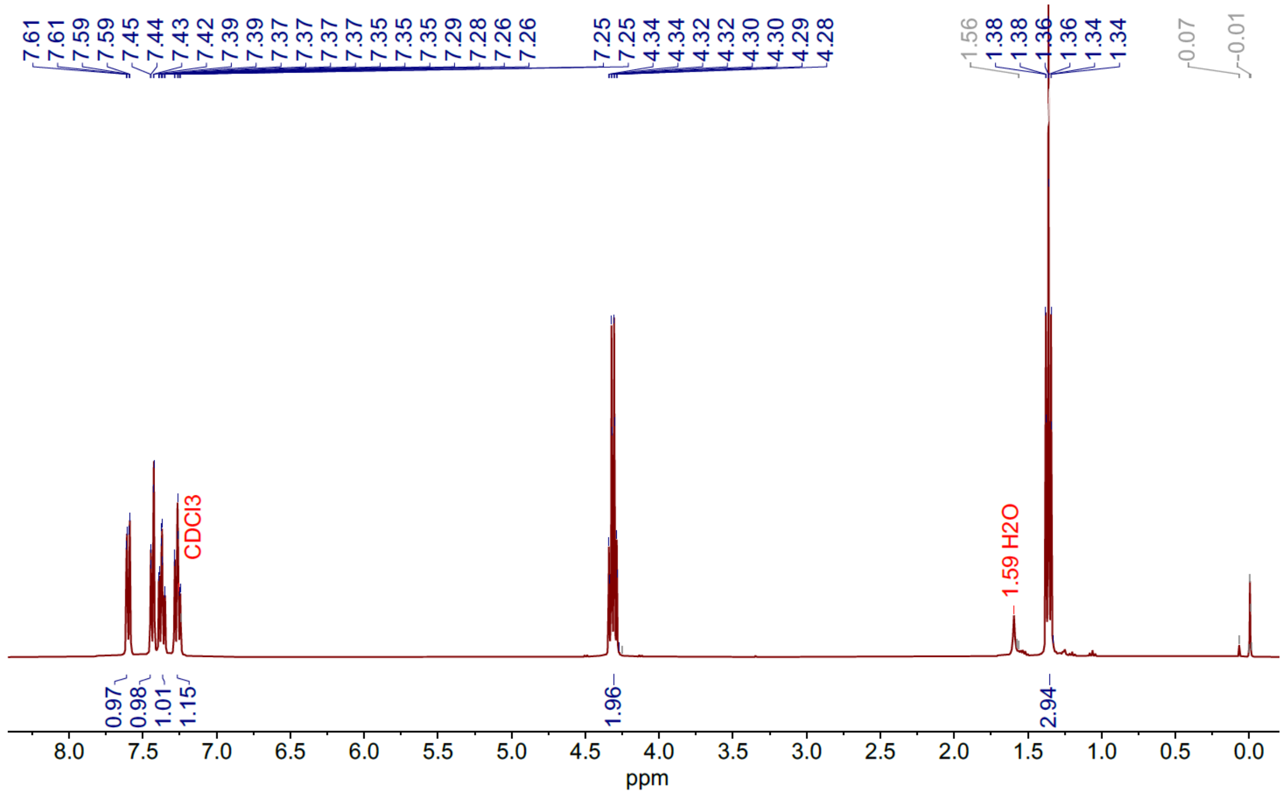

Figure S75. ^{1}H-NMR spectrum of *ethyl 3-(2-bromophenyl)propiolate* in $CDCl_3$ (400 MHz)

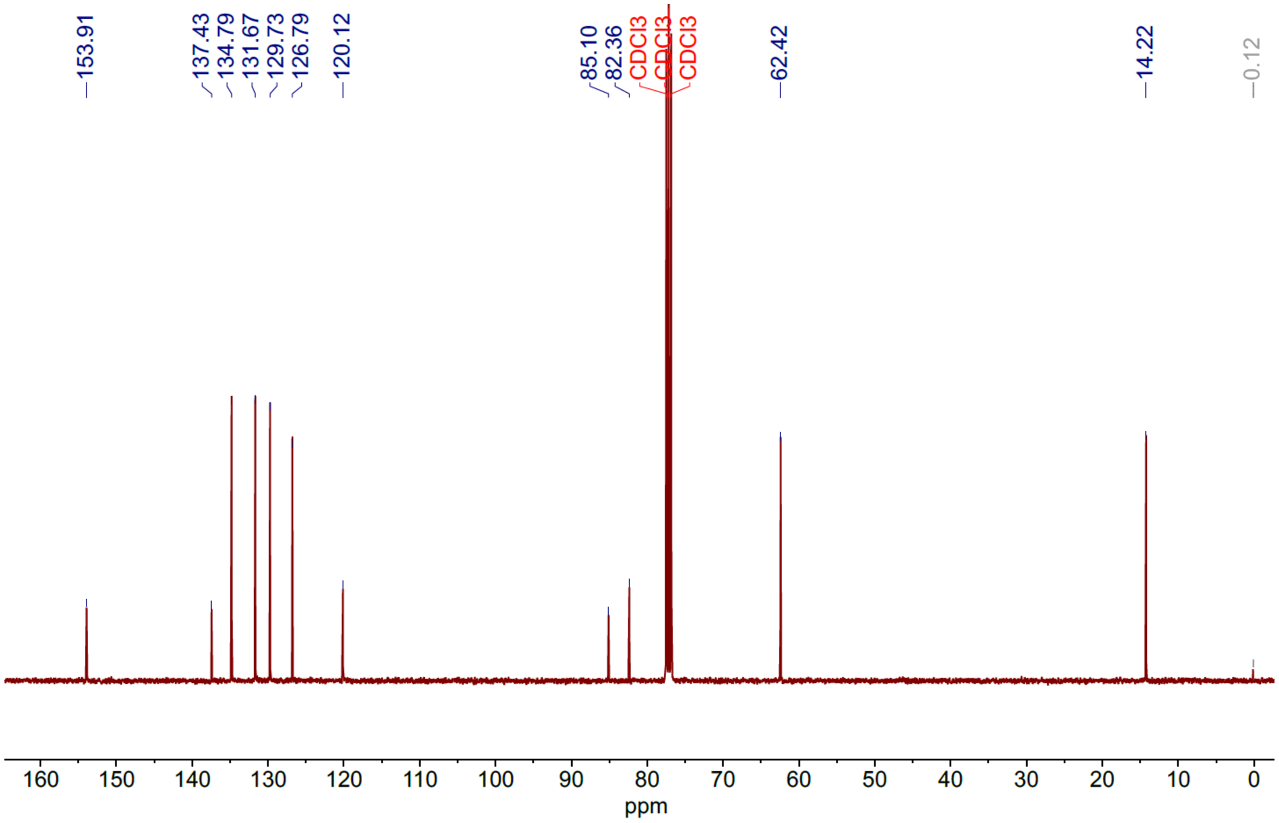

Figure S76. ^{13}C-NMR spectrum of *ethyl 3-(2-bromophenyl)propiolate* in $CDCl_3$ (101 MHz)

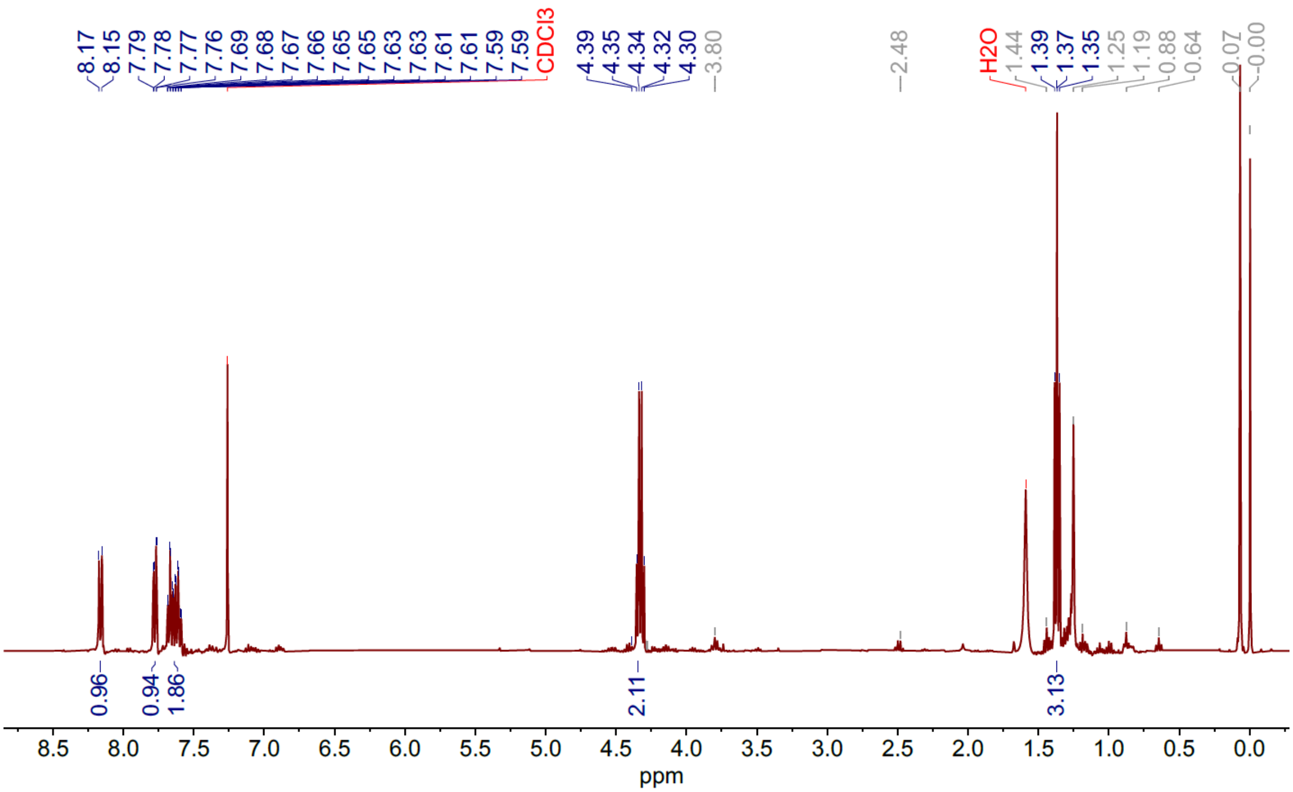

Figure S77. ^{1}H-NMR spectrum of *ethyl 3-(2-nitrophenyl)propiolate* in $CDCl_3$ (400 MHz)

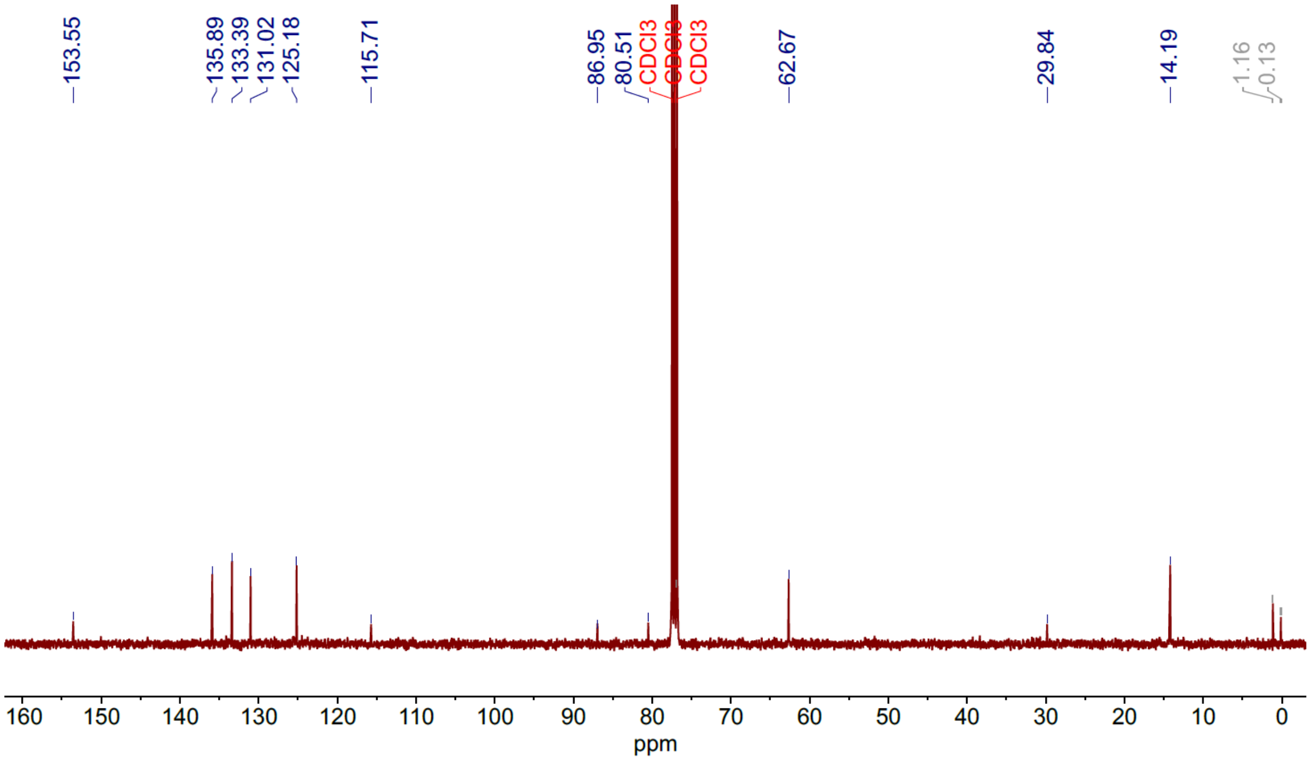

Figure S78. ^{13}C-NMR spectrum of *ethyl 3-(2-nitrophenyl)propiolate* in $CDCl_3$ (400 MHz)

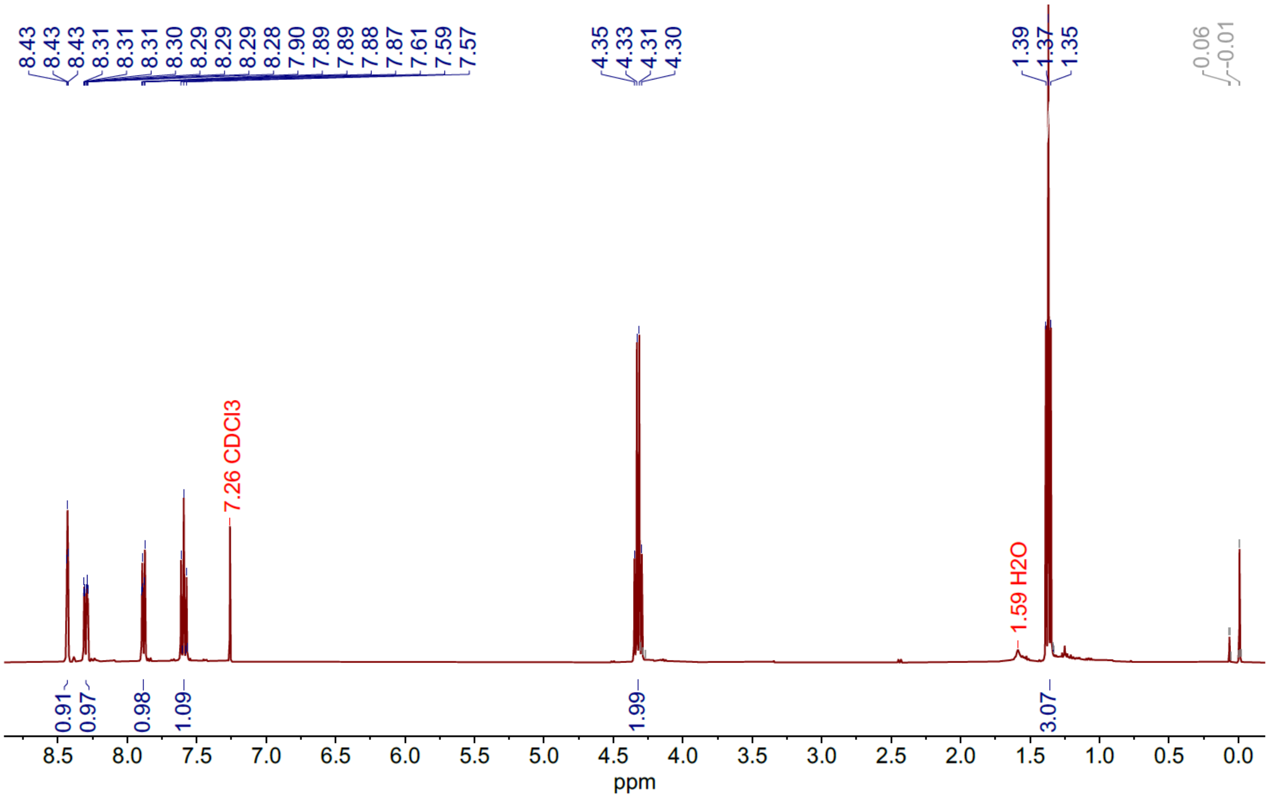

Figure S79. ^{1}H-NMR spectrum of *ethyl 3-(3-nitrophenyl)propiolate* in $CDCl_3$ (400 MHz)

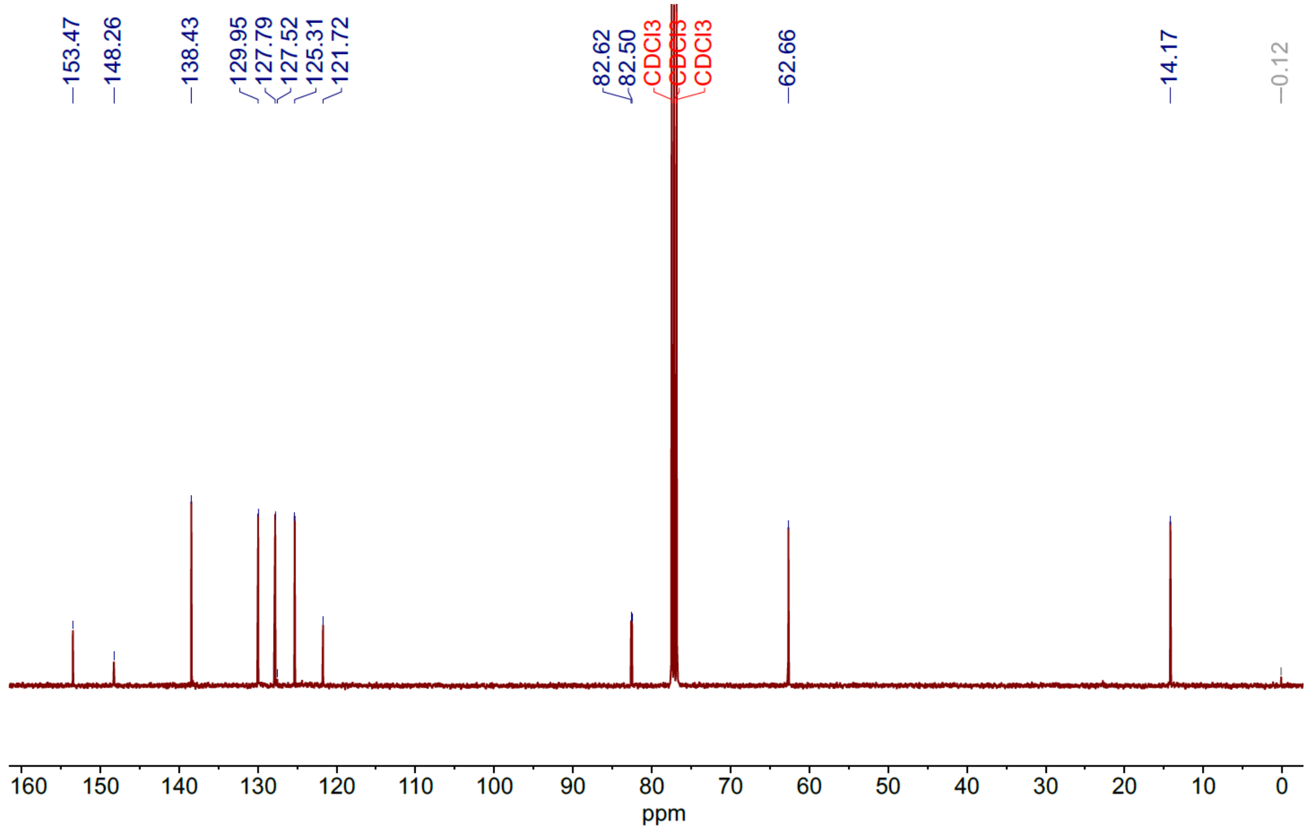

Figure S80. ^{13}C-NMR spectrum of *ethyl 3-(3-nitrophenyl)propiolate* in $CDCl_3$ (101 MHz)

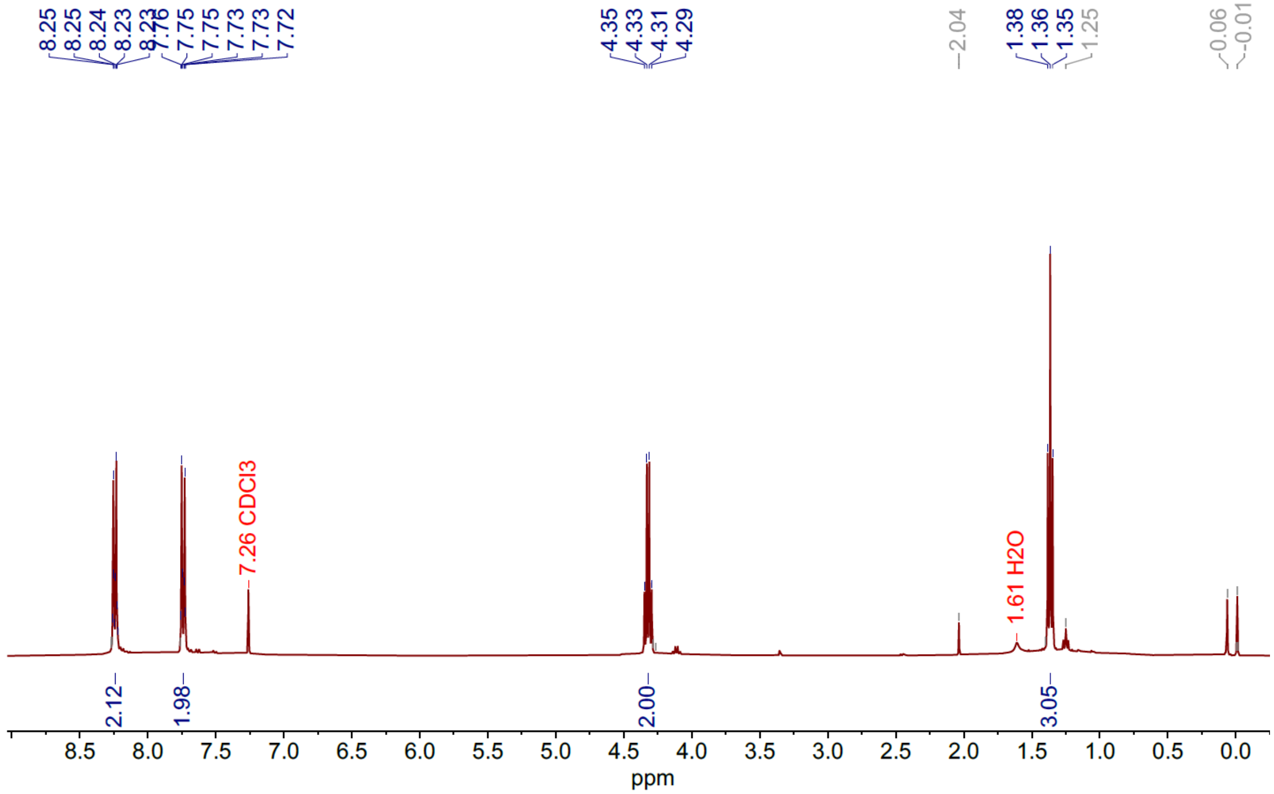

Figure S81. ^{1}H-NMR spectrum of *ethyl 3-(4-nitrophenyl)propiolate* in $CDCl_3$ (400 MHz)

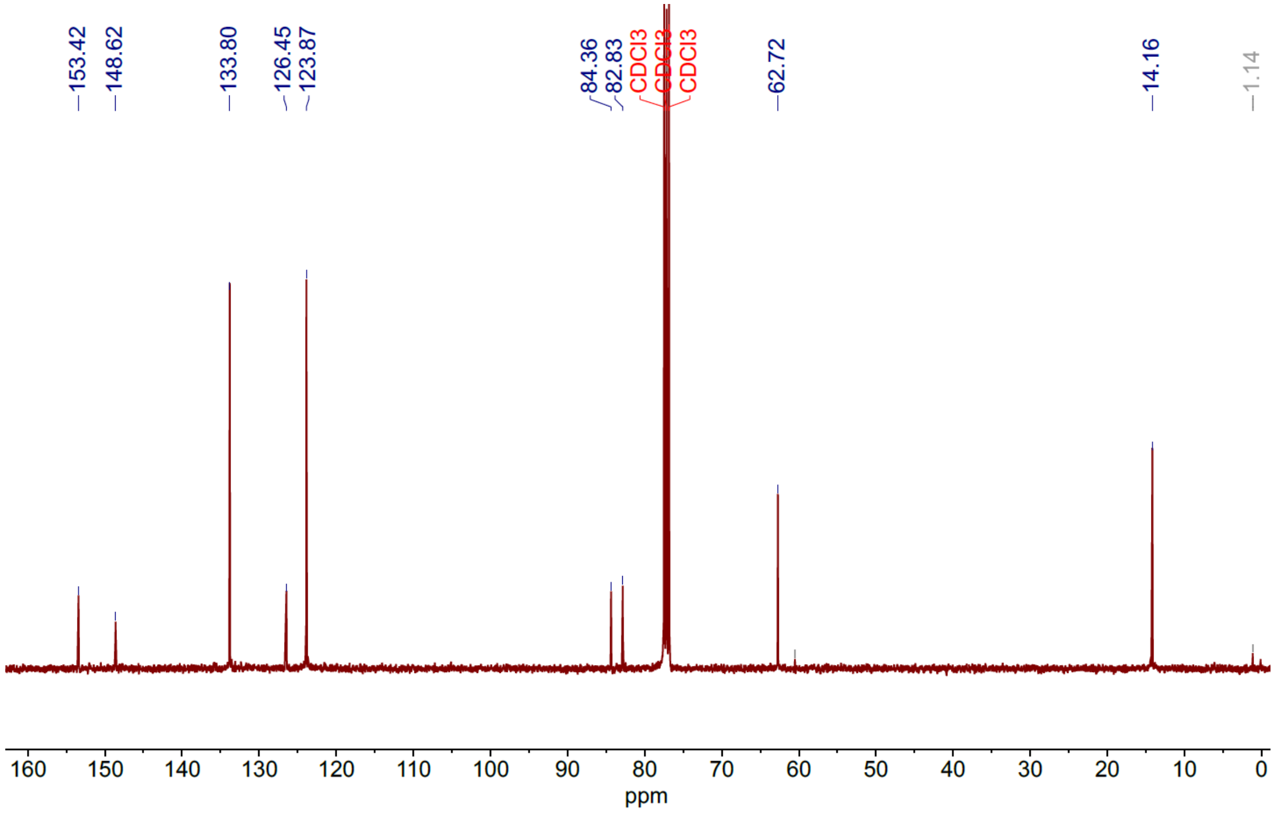

Figure S82. ^{13}C-NMR spectrum of *ethyl 3-(4-nitrophenyl)propiolate* in $CDCl_3$ (101 MHz)

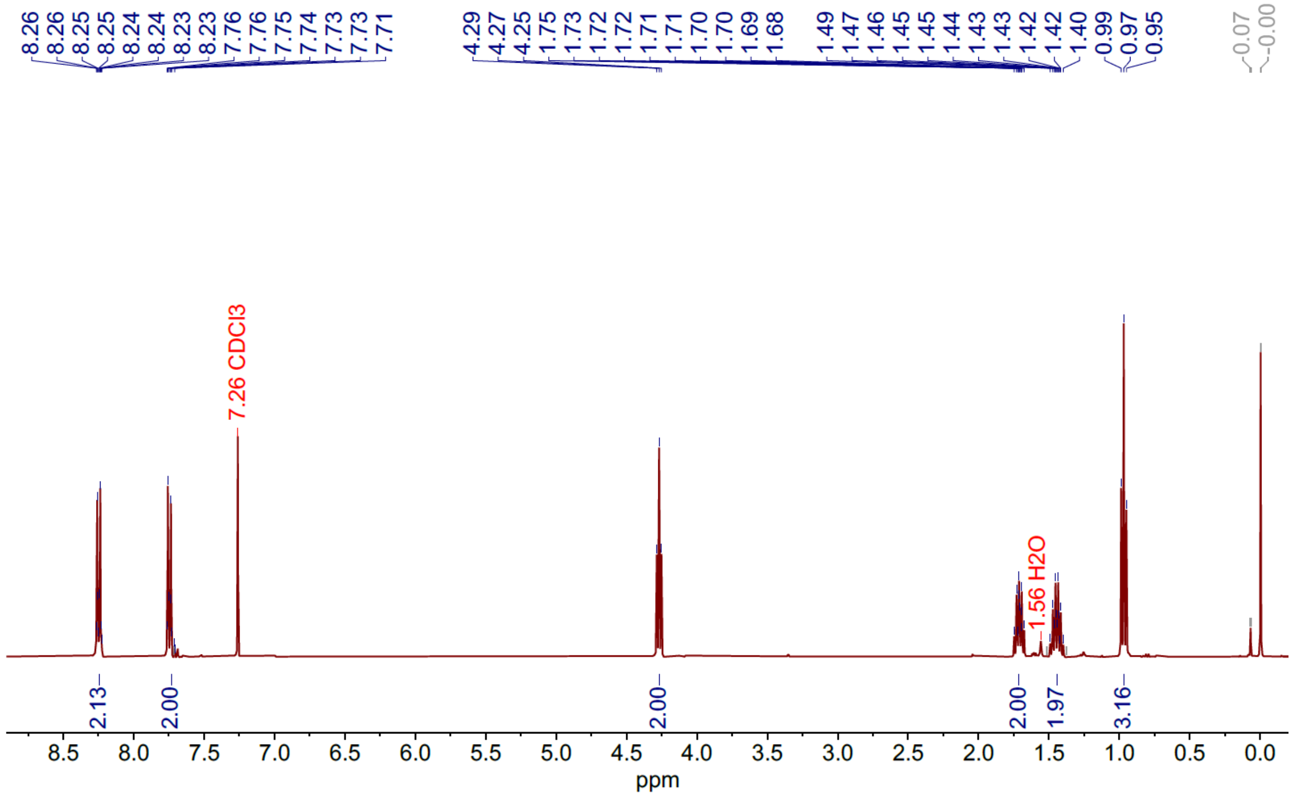

Figure S83. ^{1}H-NMR spectrum of *butyl 3-(4-nitrophenyl)propiolate* in $CDCl_3$ (400 MHz)

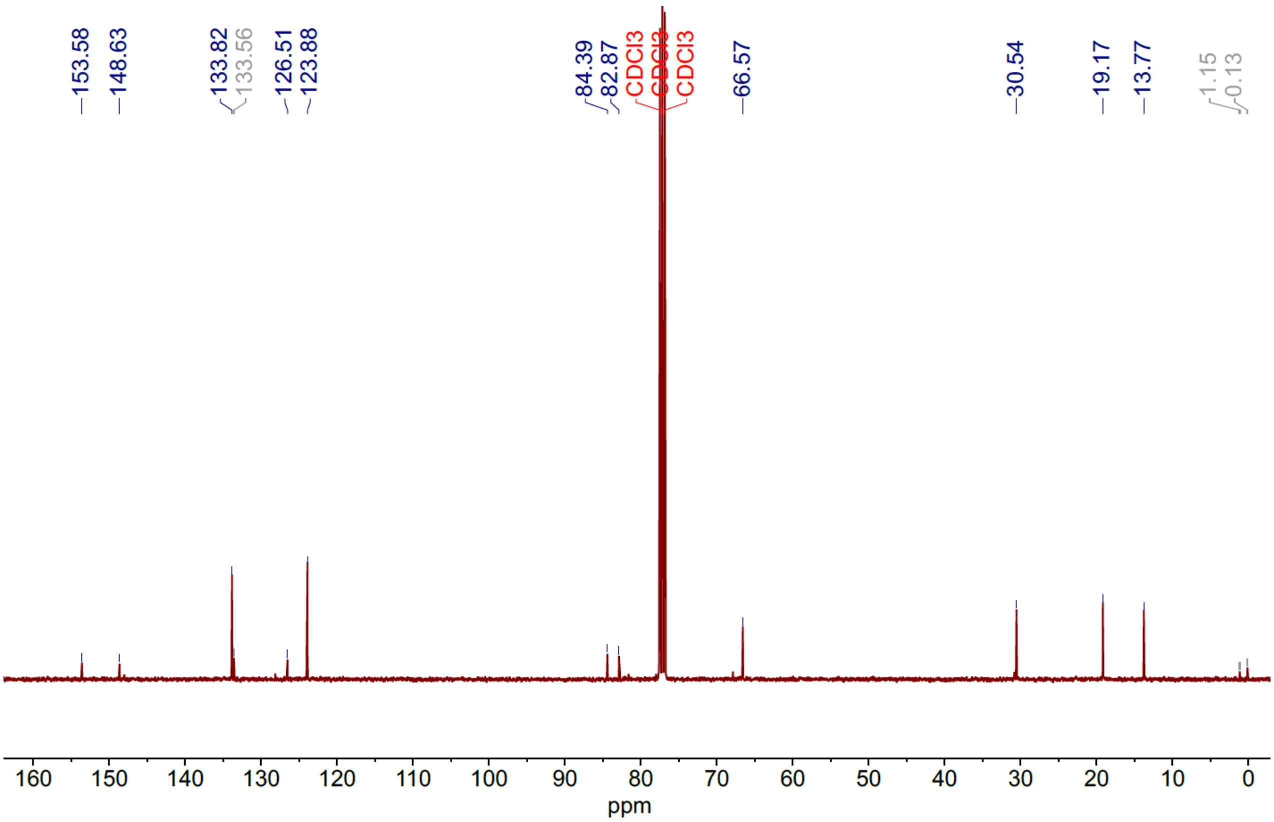

Figure S84. ^{13}C-NMR spectrum of *butyl 3-(4-nitrophenyl)propiolate* in $CDCl_3$ (101 MHz)

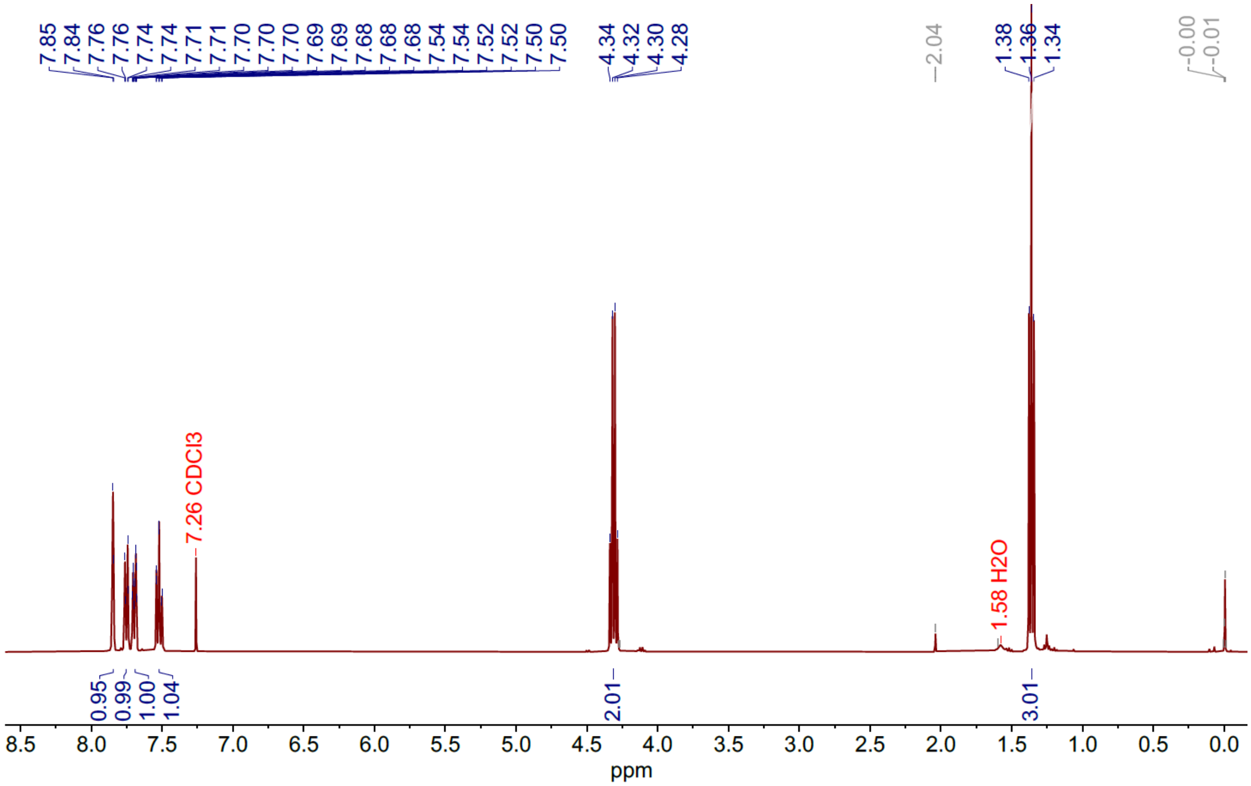

Figure S85. ^{1}H-NMR spectrum of *ethyl 3-(3-(trifluoromethyl)phenyl)propiolate* in $CDCl_3$

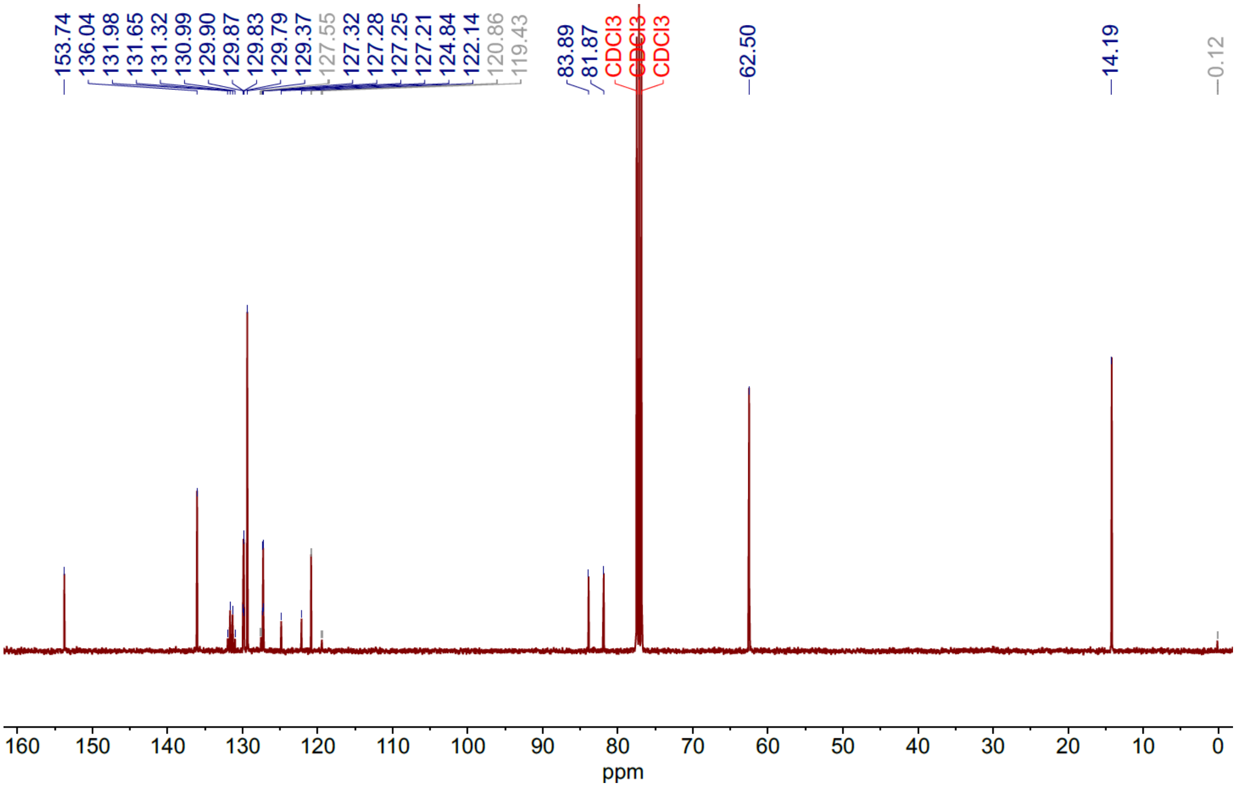

Figure S86. ^{13}C-NMR spectrum of *ethyl 3-(3-(trifluoromethyl)phenyl)propiolate* in $CDCl_3$ (101 MHz)

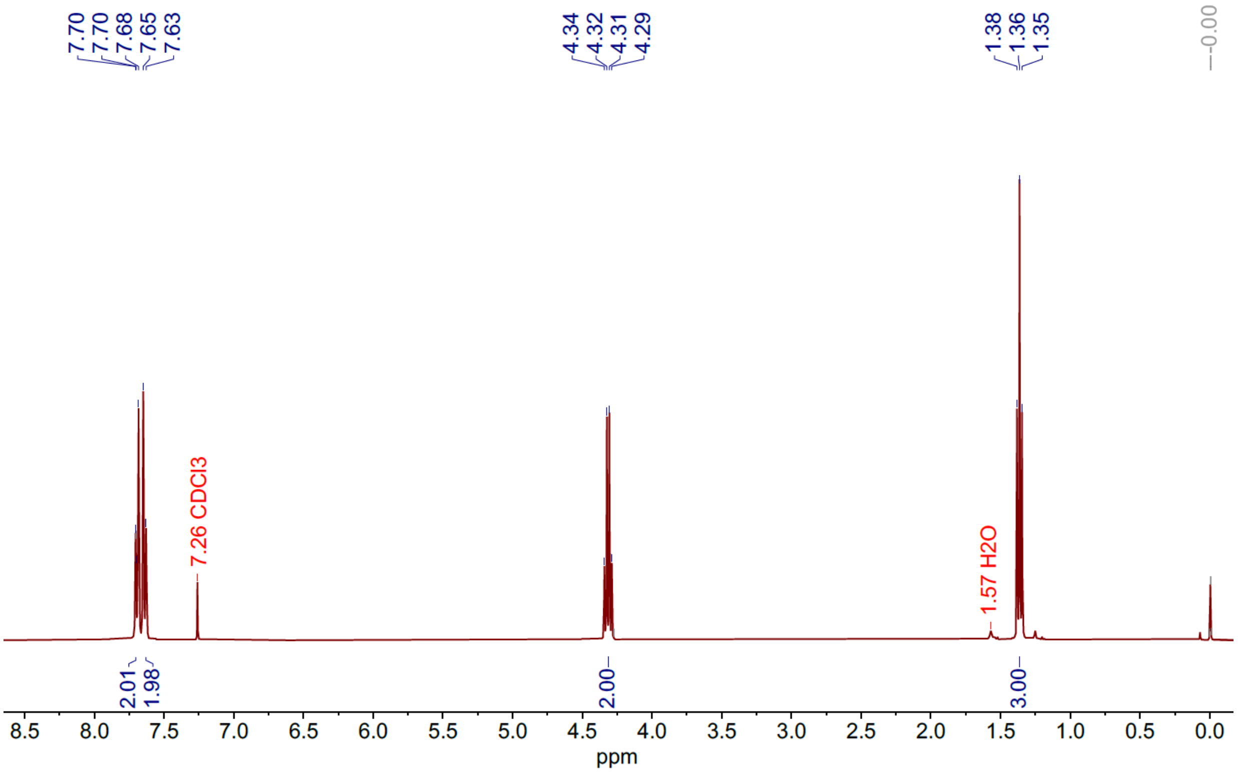

Figure S87. ^{1}H-NMR spectrum of *ethyl 3-(4-(trifluoromethyl)phenyl)propiolate* in $CDCl_3$ (400 MHz)

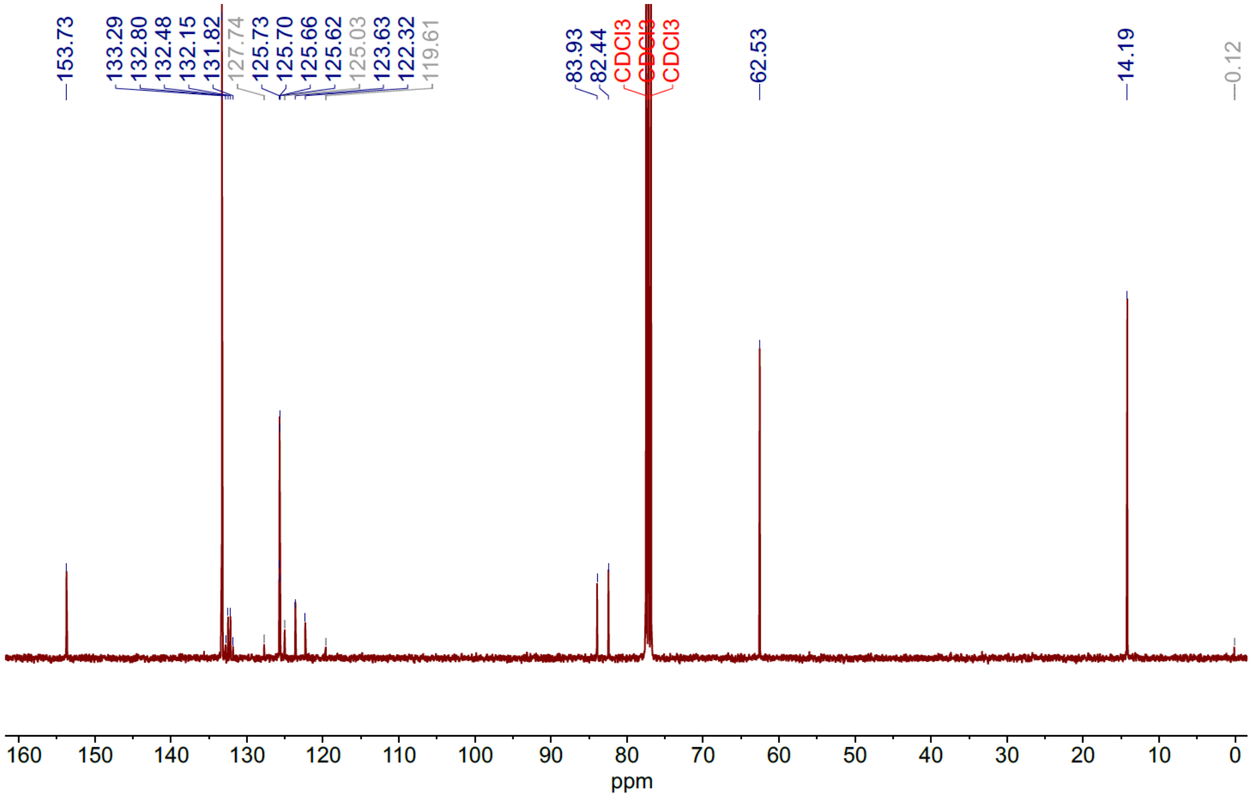

Figure S88. ^{1}H-NMR spectrum of *ethyl 3-(4-(trifluoromethyl)phenyl)propiolate* in $CDCl_3$ (101 MHz)

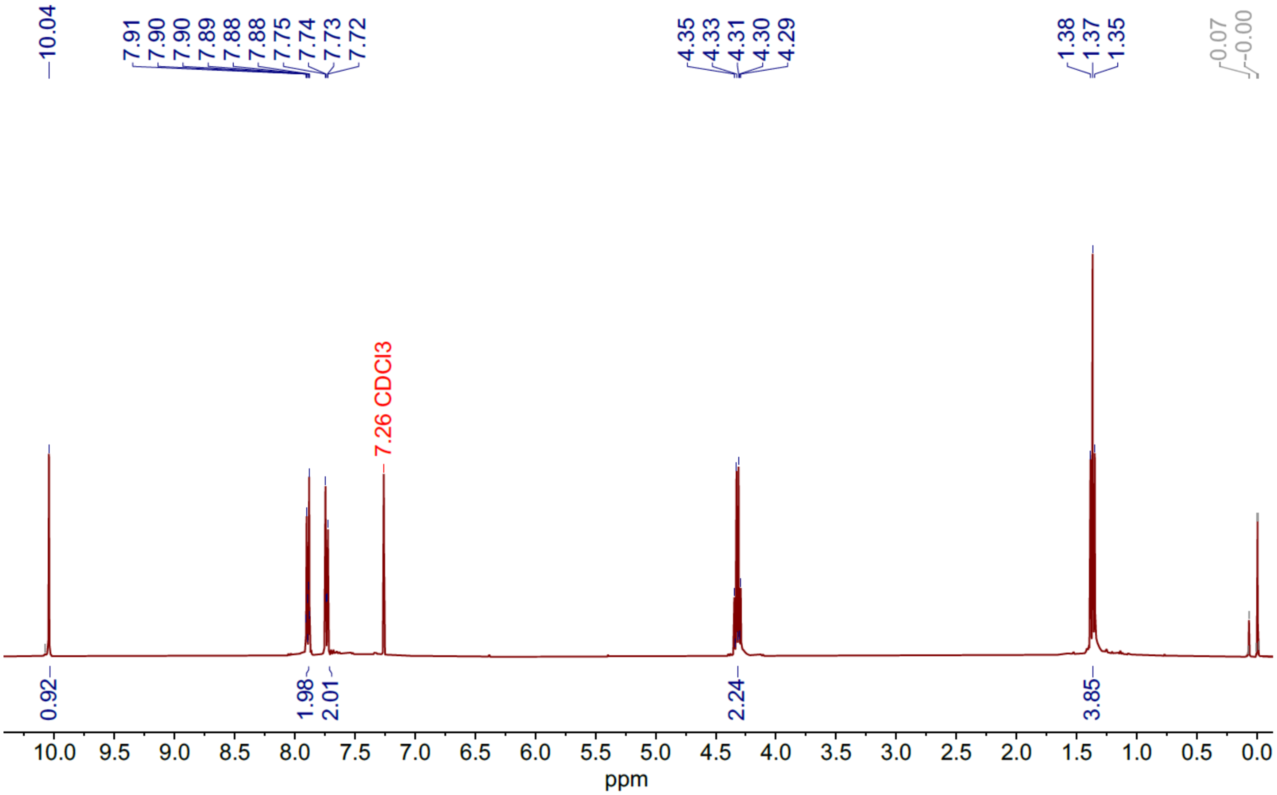

Figure S89. ^{1}H-NMR spectrum of *ethyl 3-(4-formylphenyl)propiolate* in $CDCl_3$ (400 MHz)

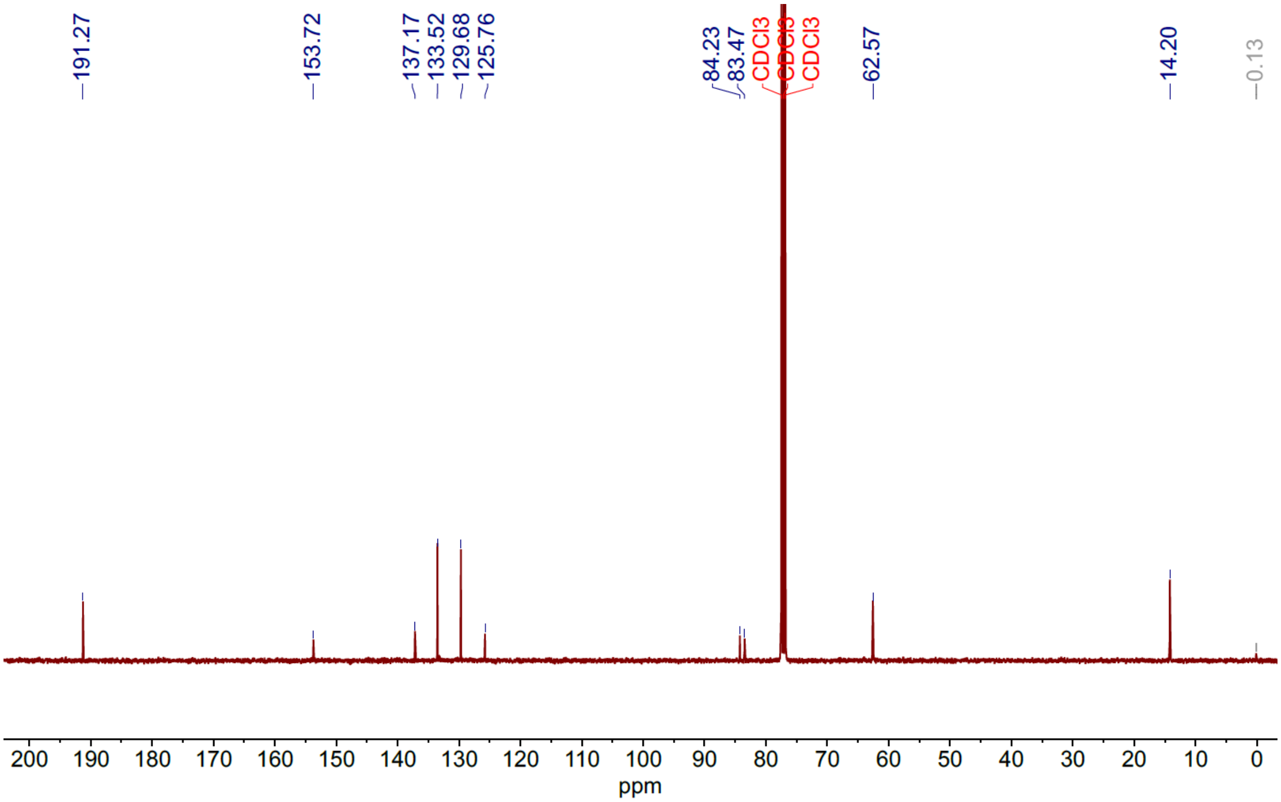

Figure S90. ^{13}C-NMR spectrum of *ethyl 3-(4-formylphenyl)propiolate* in $CDCl_3$ (101 MHz)

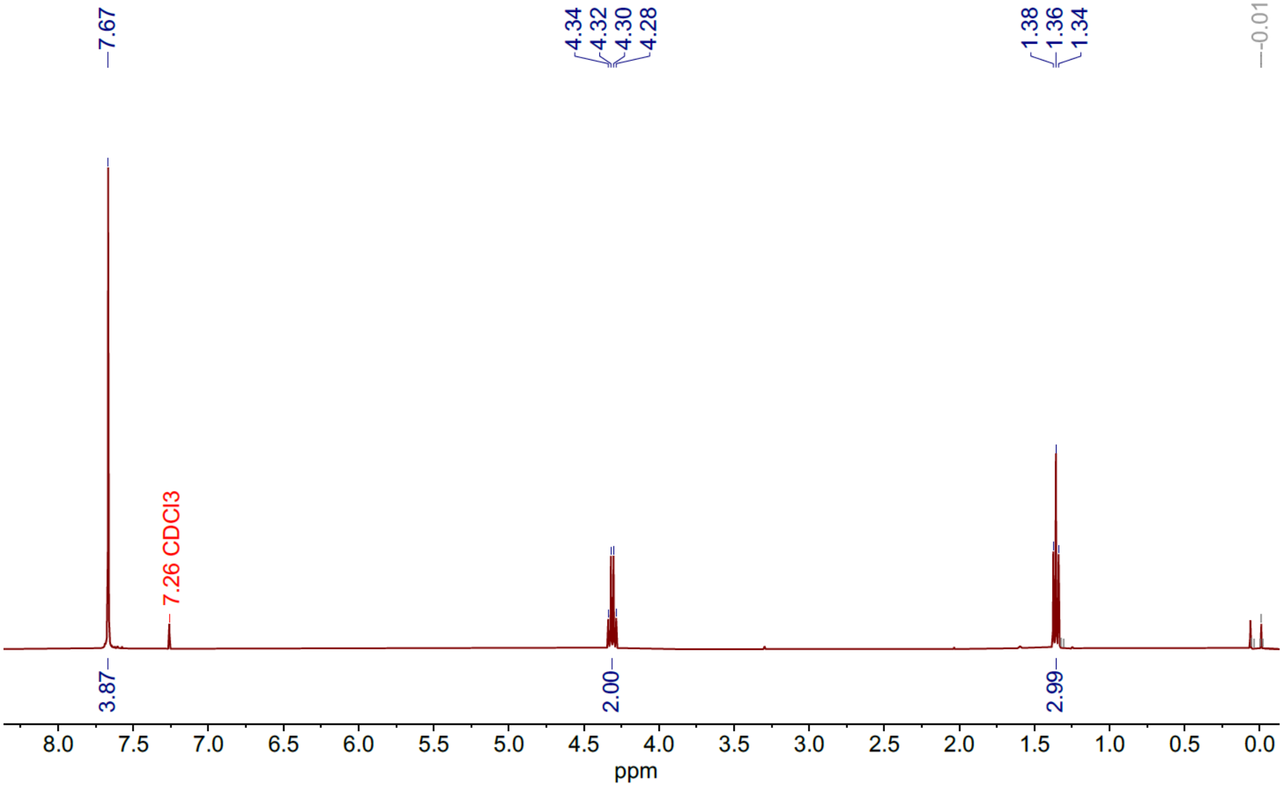

Figure S91. ^{1}H-NMR spectrum of *ethyl 3-(4-cyanophenyl)propiolate* in $CDCl_3$ (400 MHz)

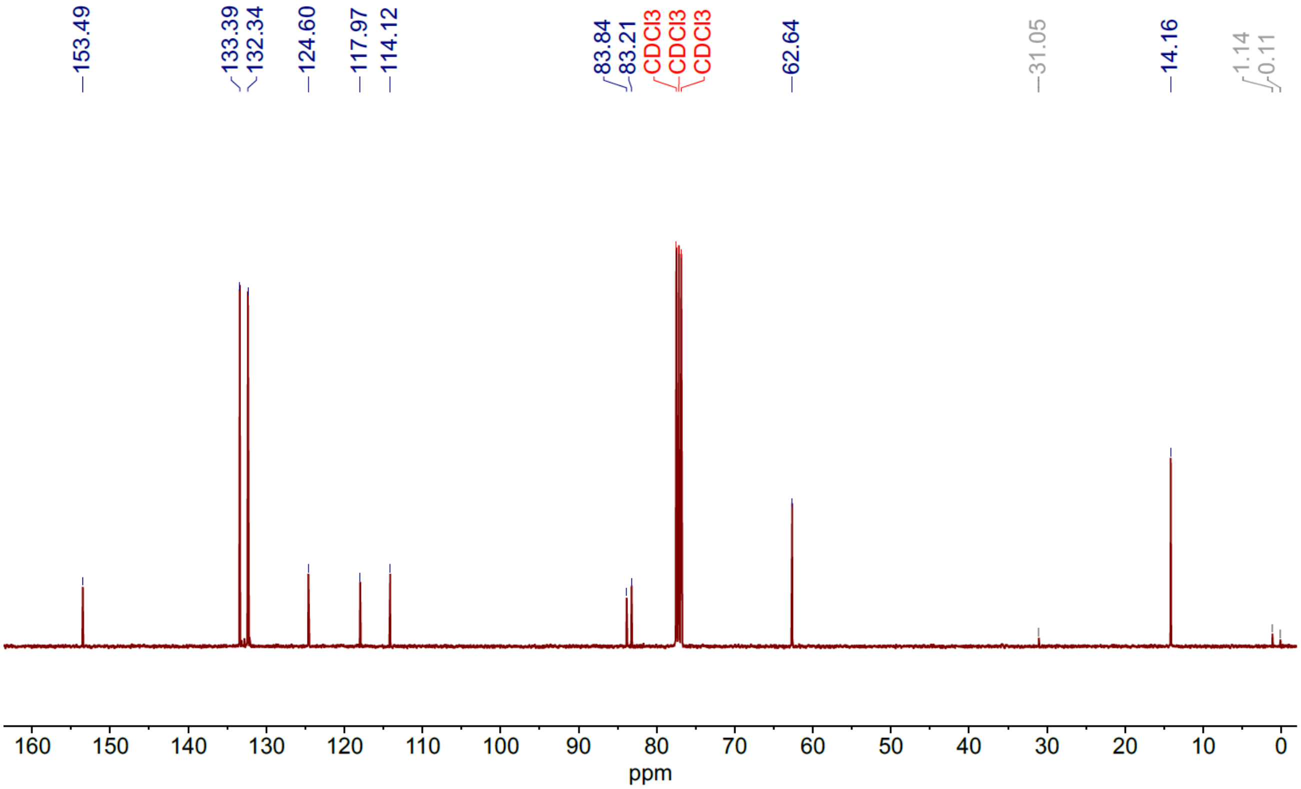

Figure S92. ^{13}C-NMR spectrum of *ethyl 3-(4-cyanophenyl)propiolate* in $CDCl_3$ (101 MHz)

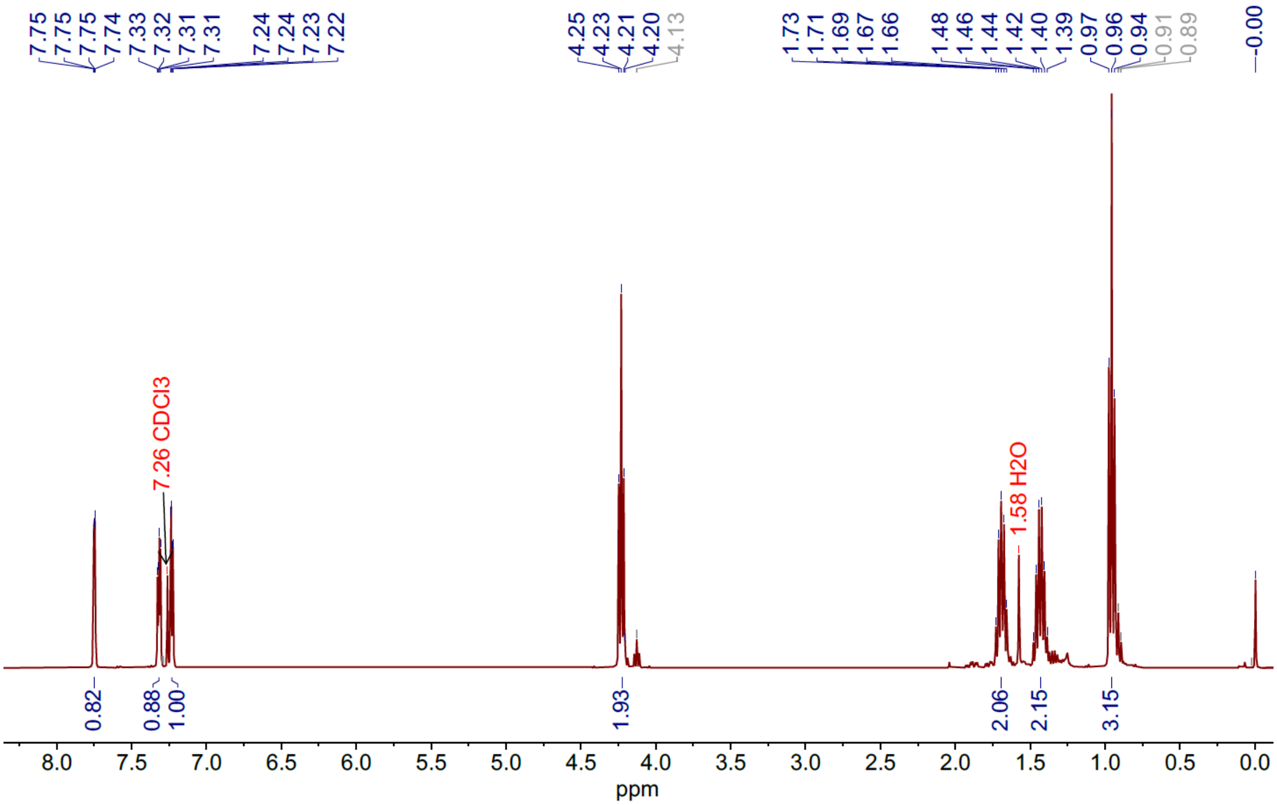

Figure S93. ^{1}H-NMR spectrum of *butyl 3-(thiophen-3-yl)propiolate* in $CDCl_3$ (400 MHz)

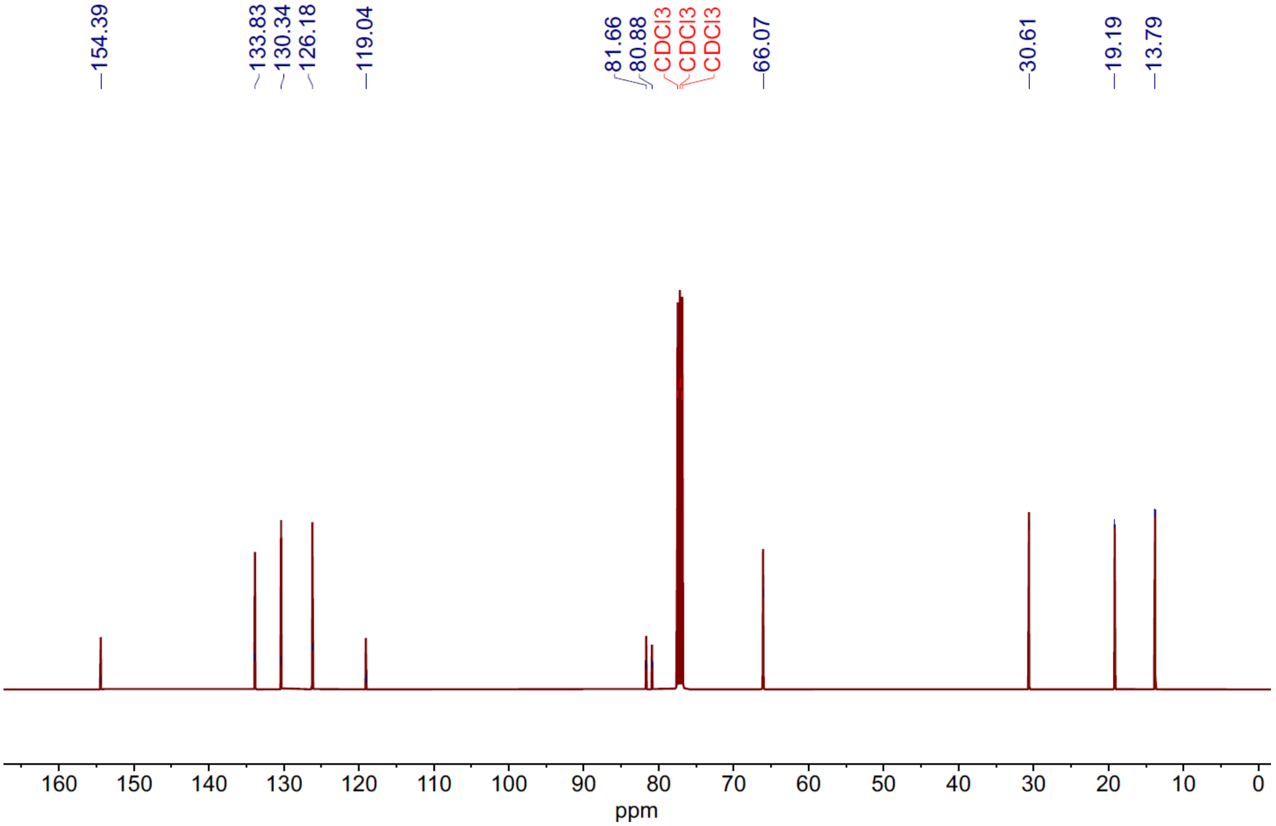

Figure S94. ^{13}C-NMR spectrum of *butyl 3-(thiophen-3-yl)propiolate* in $CDCl_3$ (101 MHz)

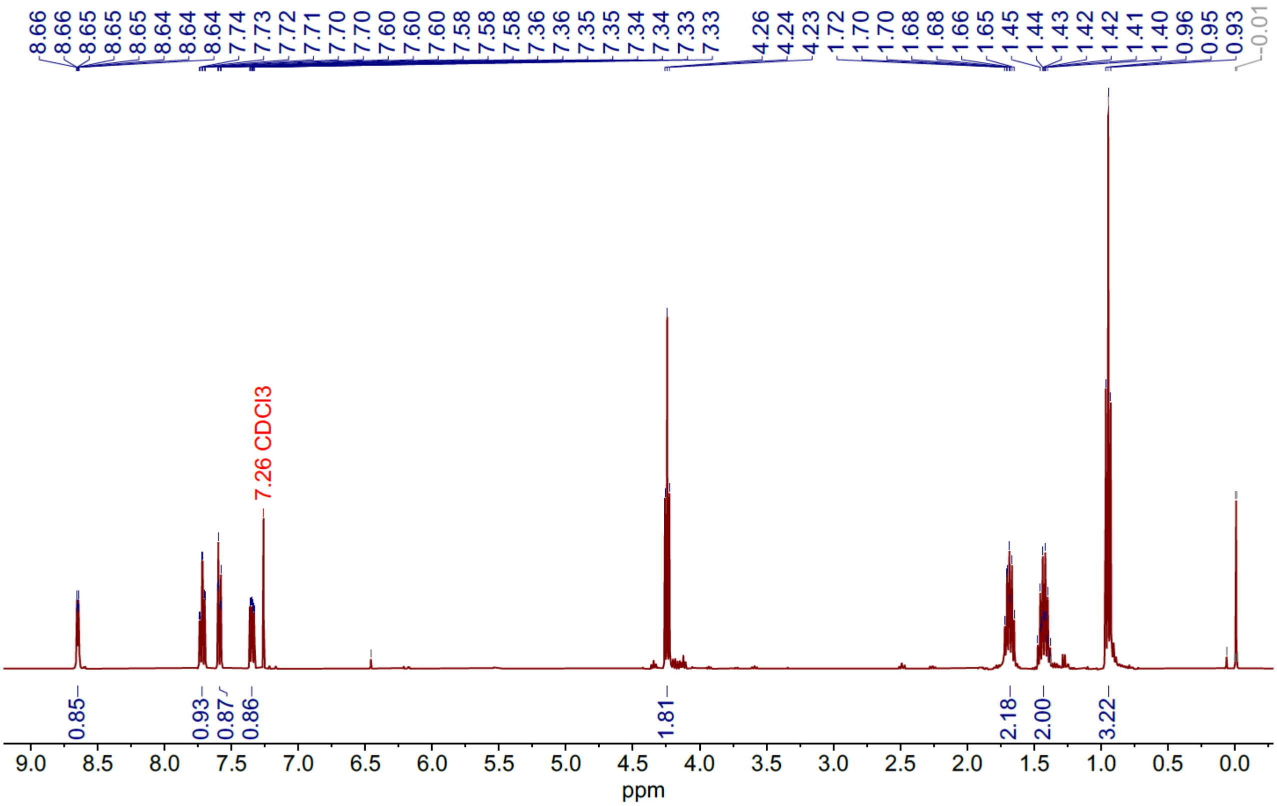

Figure S95. ^{1}H-NMR spectrum of *butyl 3-(pyridin-2-yl)propiolate* in $CDCl_3$ (400 MHz)

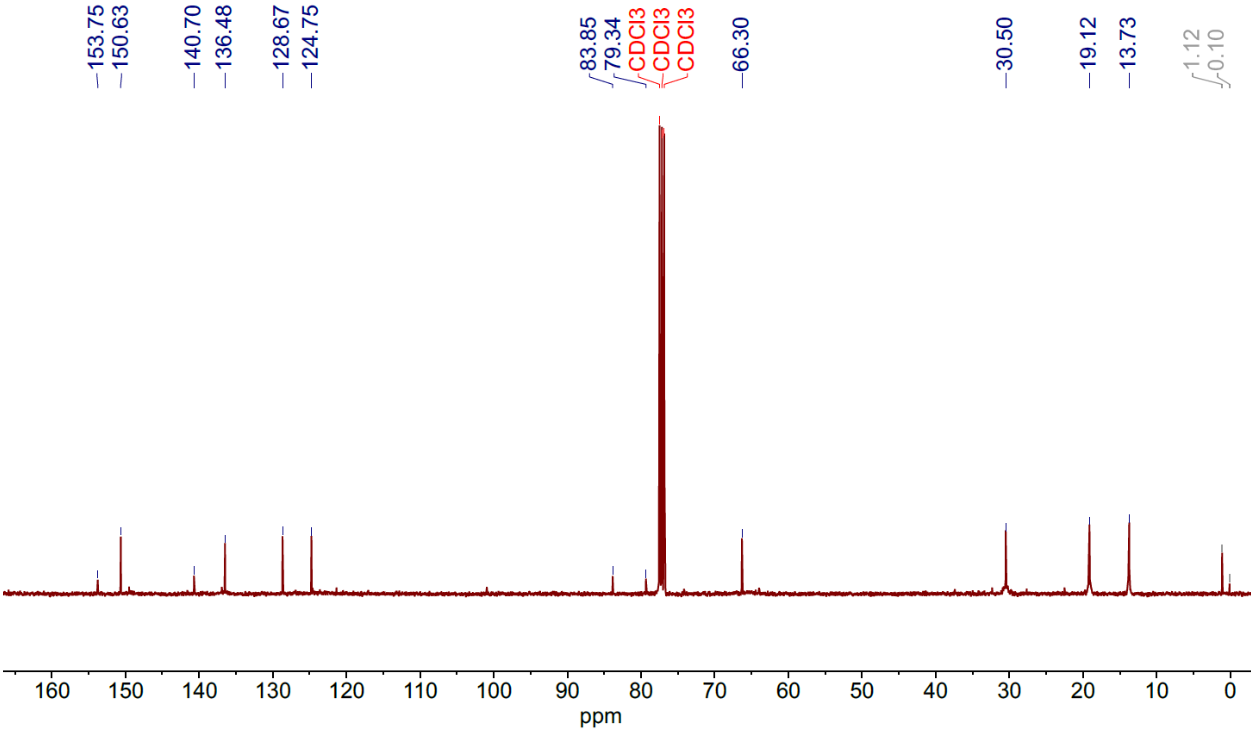

Figure S96. ^{13}C-NMR spectrum of *butyl 3-(pyridin-2-yl)propiolate* in $CDCl_3$ (101 MHz)

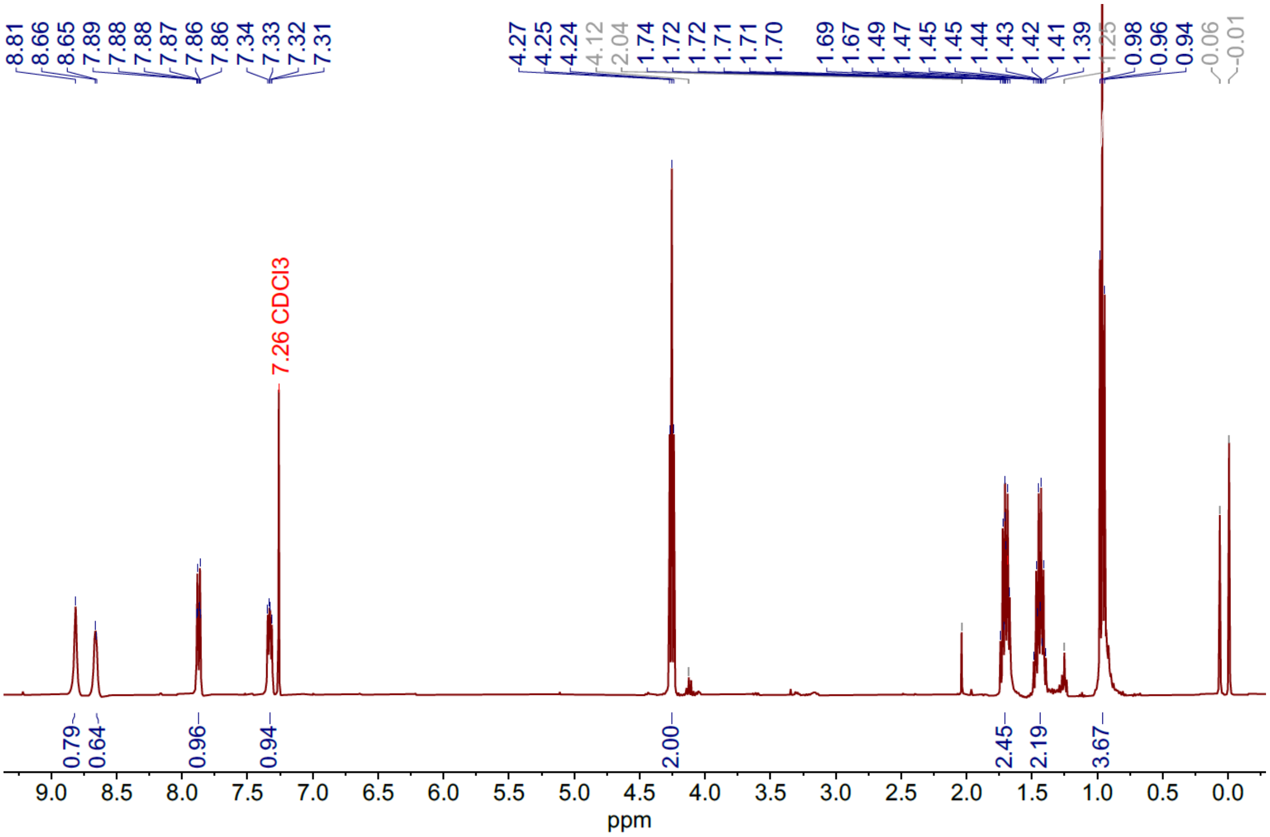

Figure S97. ^{1}H-NMR spectrum of *butyl 3-(pyridin-3-yl)propiolate* in $CDCl_3$ (400 MHz)

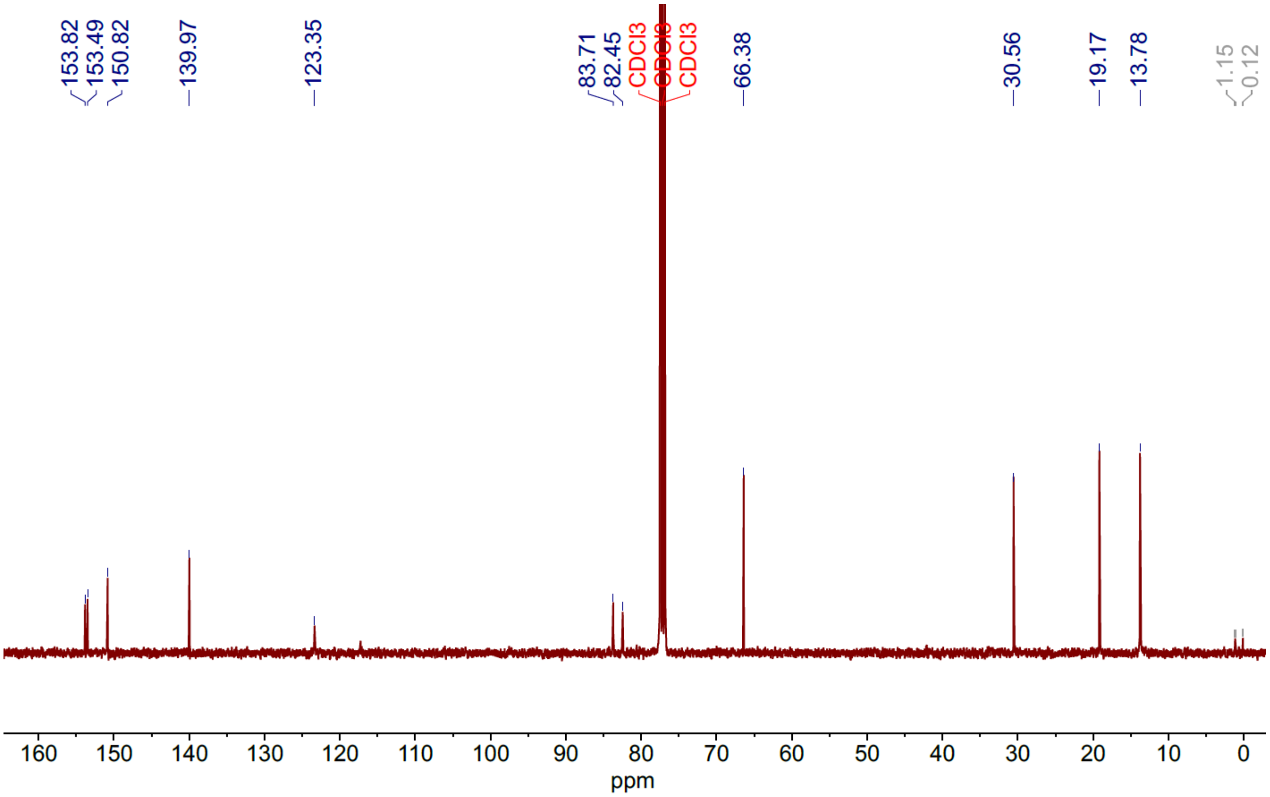

Figure S98. ^{13}C-NMR spectrum of *butyl 3-(pyridin-3-yl)propiolate* in $CDCl_3$ (101 MHz)

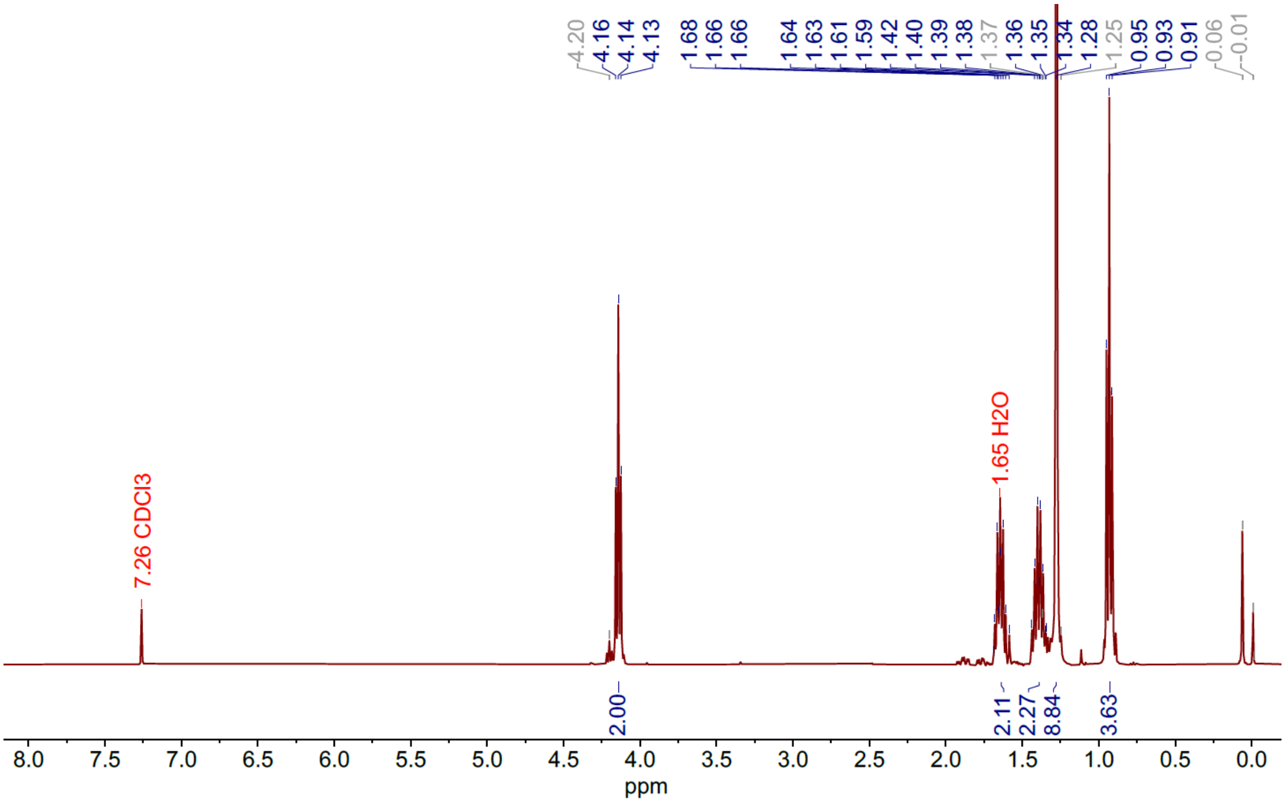

Figure S99. ^{1}H-NMR spectrum of *butyl 4,4-dimethylpent-2-ynoate* in $CDCl_3$ (400 MHz)

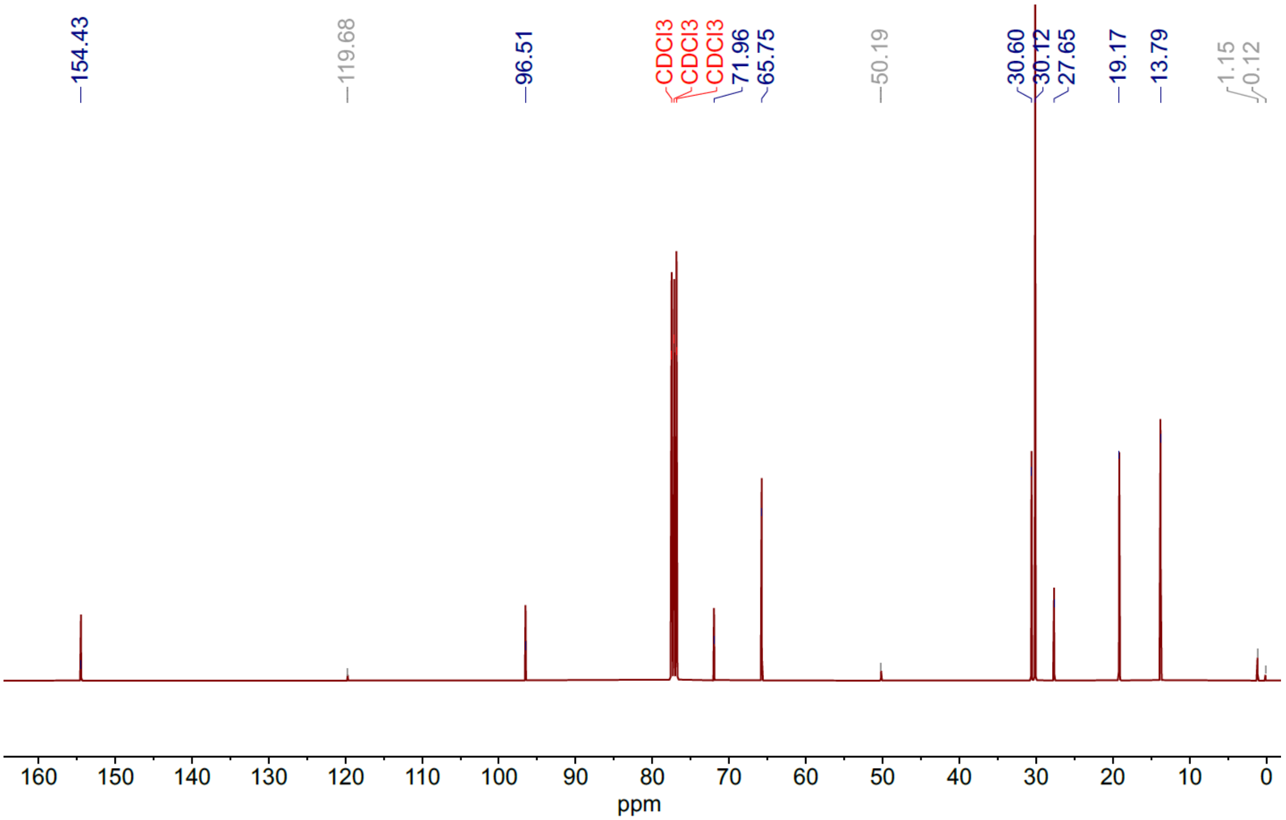

Figure S100. ^{13}C-NMR spectrum of *butyl 4,4-dimethylpent-2-ynoate* in $CDCl_3$ (101 MHz)

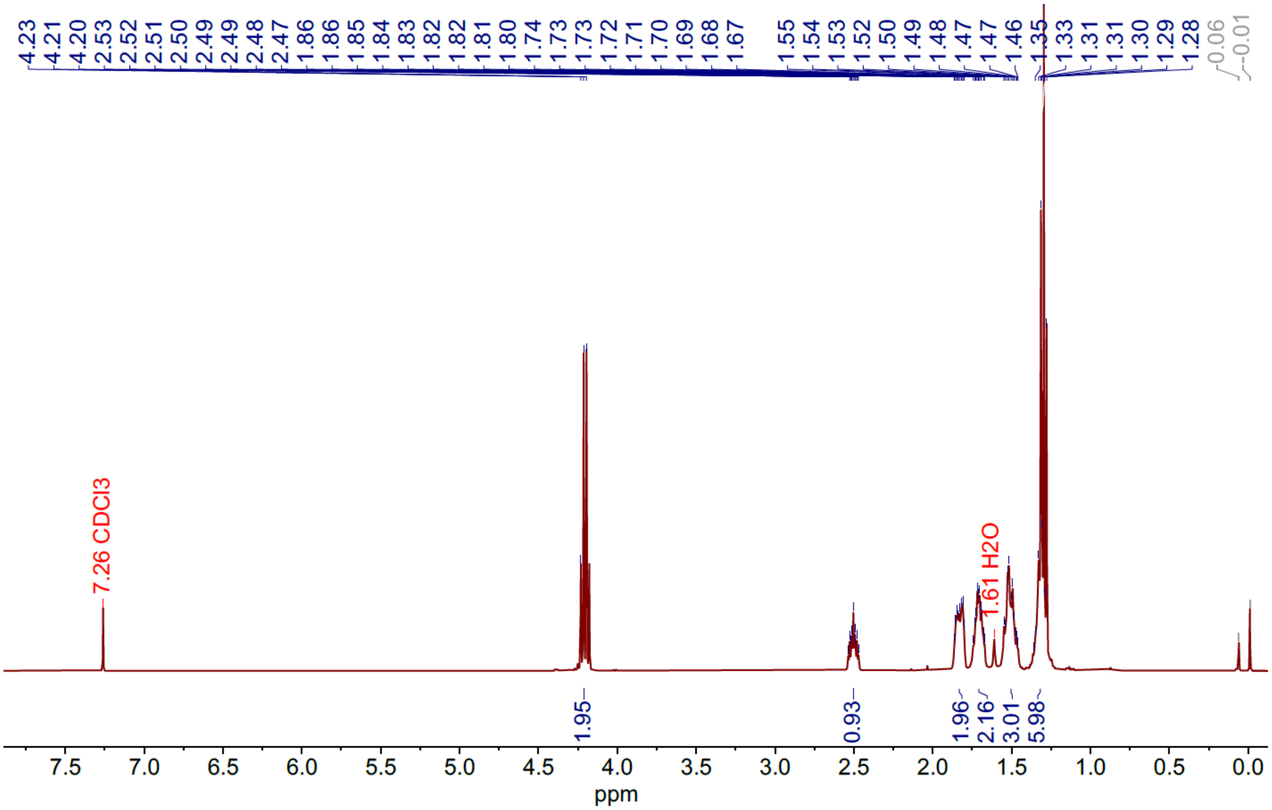

Figure S101. ^{1}H-NMR spectrum of *ethyl 3-cyclohexylpropiolate* in $CDCl_3$ (400 MHz)

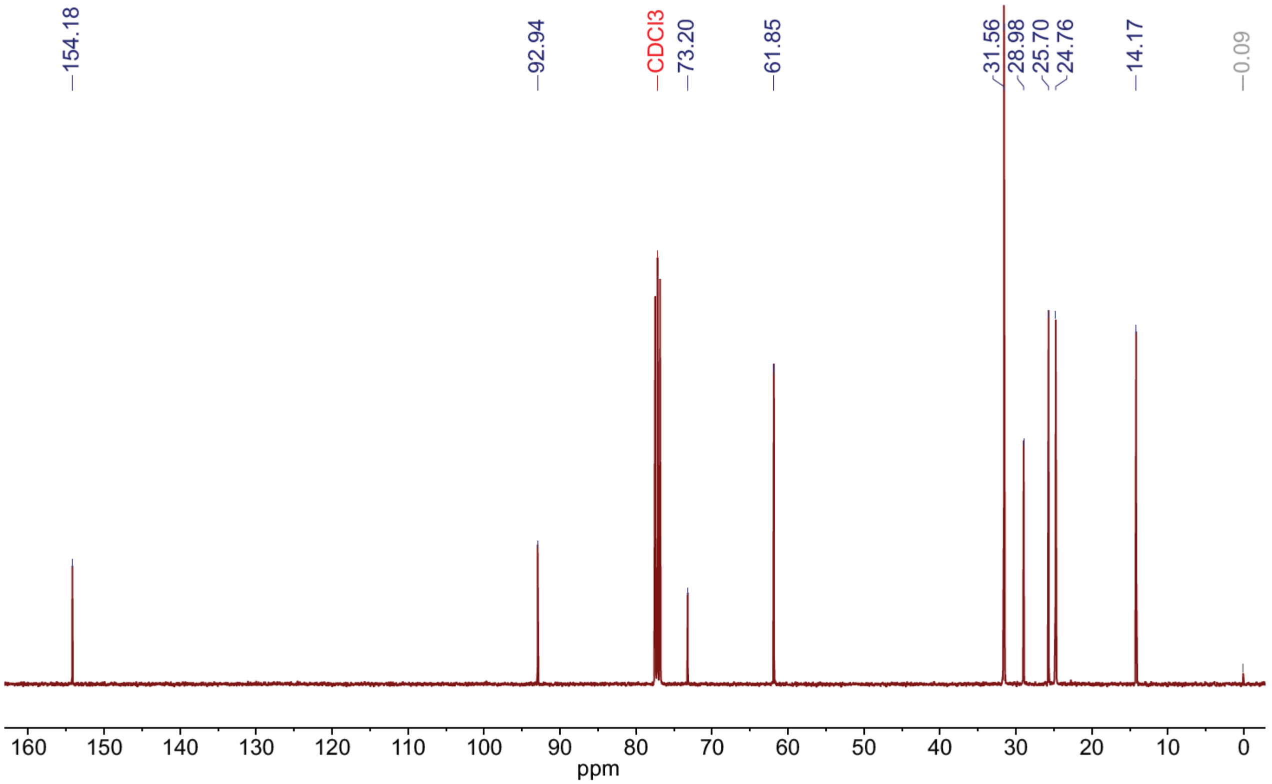

Figure S102. ^{13}C-NMR spectrum of *ethyl 3-cyclohexylpropiolate* in $CDCl_3$ (101 MHz)

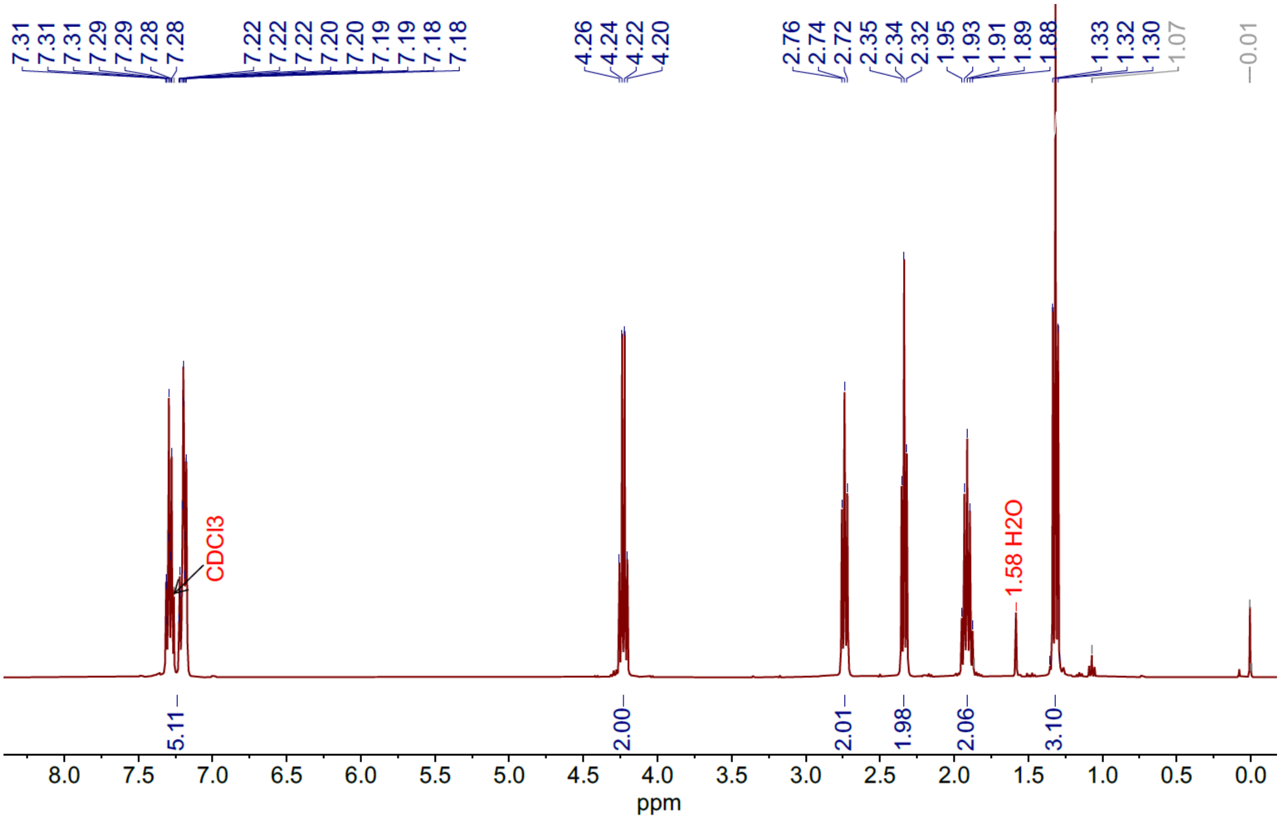

Figure S103. ^{1}H-NMR spectrum of *ethyl 6-phenylhex-2-ynoate* in $CDCl_3$ (400 MHz)

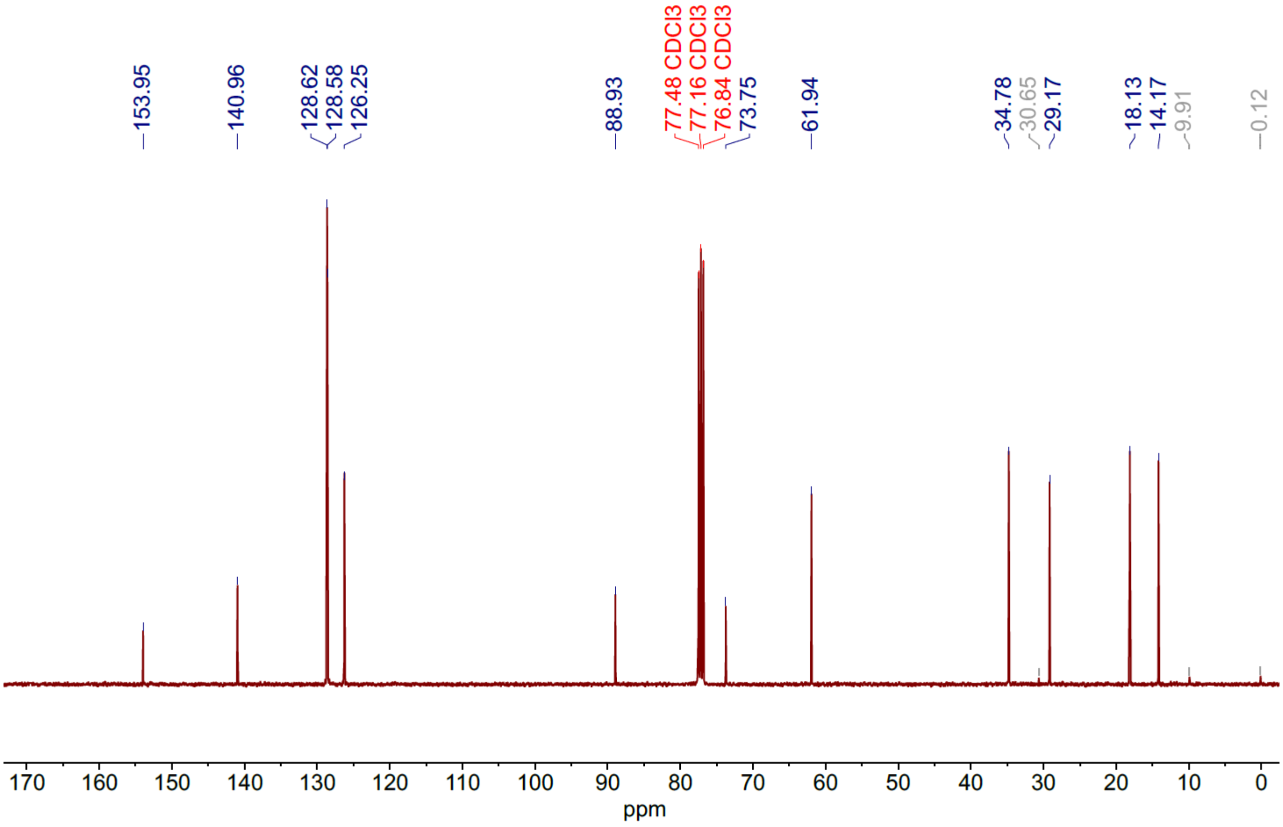

Figure S104. ^{13}C-NMR spectrum of *ethyl 6-phenylhex-2-ynoate* in $CDCl_3$ (101 MHz)

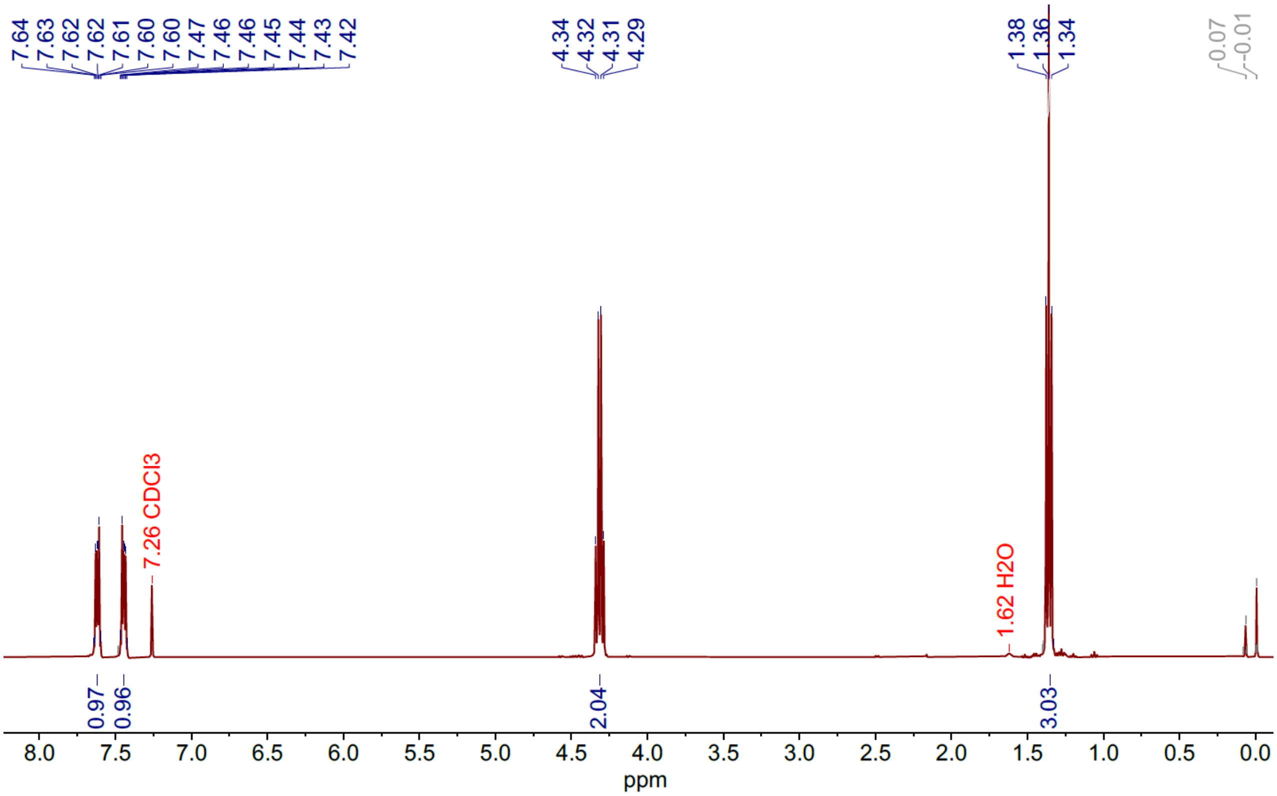

Figure S105. ^{1}H-NMR spectrum of *diethyl 3,3'-(1,2-phenylene)dipropiolate* in $CDCl_3$ (400 MHz)

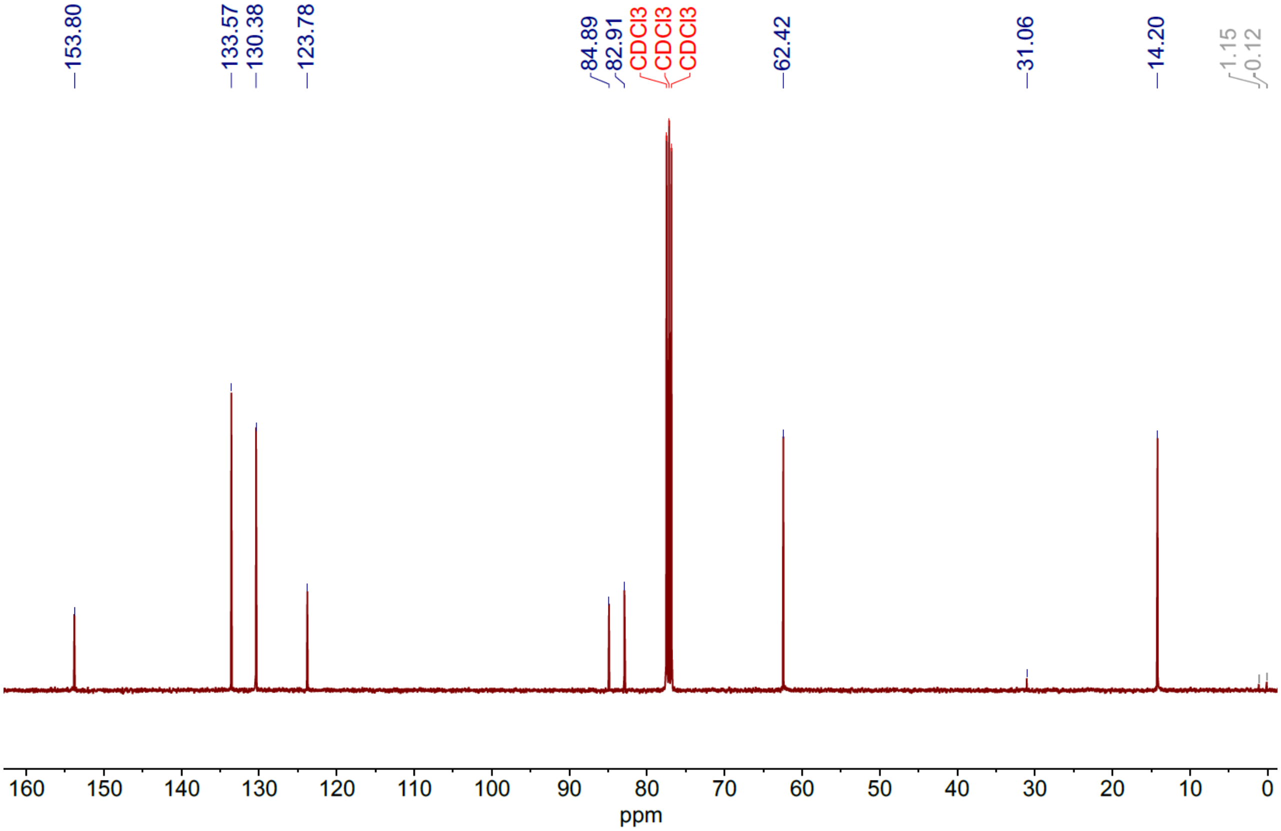

Figure S106. ^{13}C-NMR spectrum of *diethyl 3,3'-(1,2-phenylene)dipropiolate* in $CDCl_3$ (101 MHz)

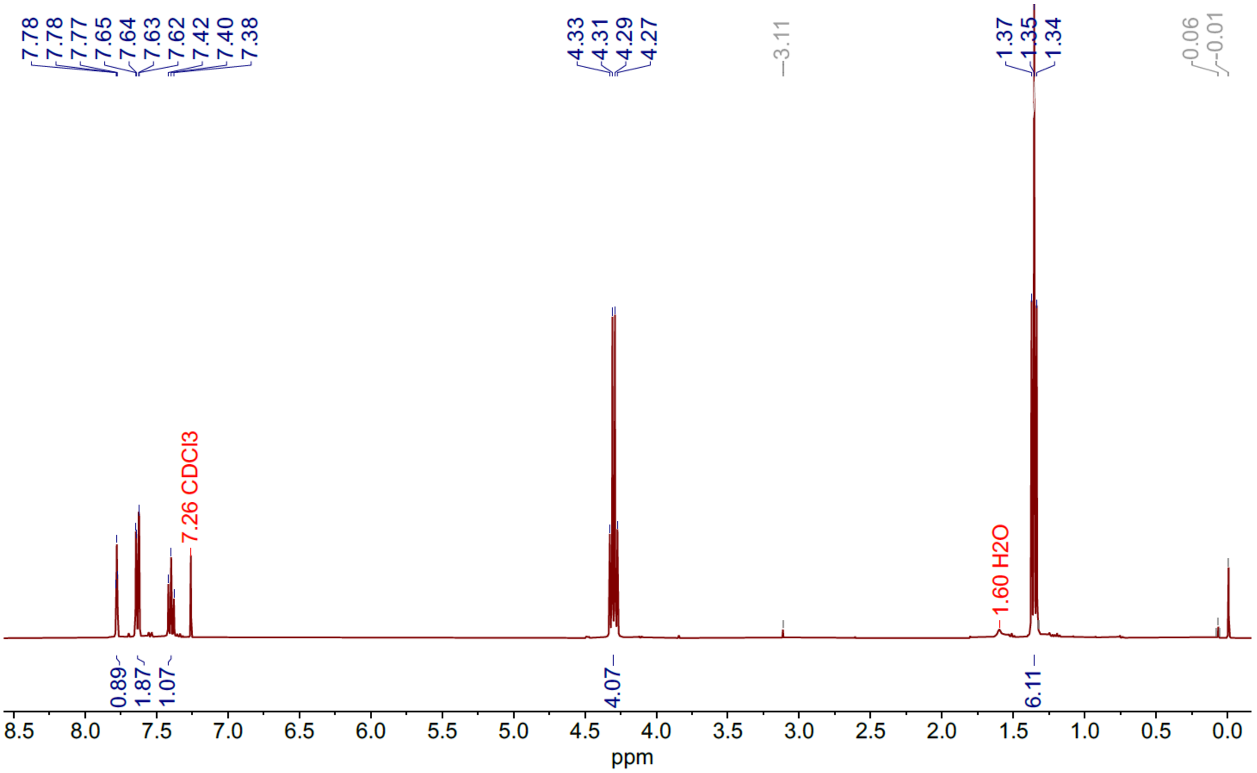

Figure S107. ^{1}H-NMR spectrum of *diethyl 3,3'-(1,3-phenylene)dipropiolate* in $CDCl_3$ (400 MHz)

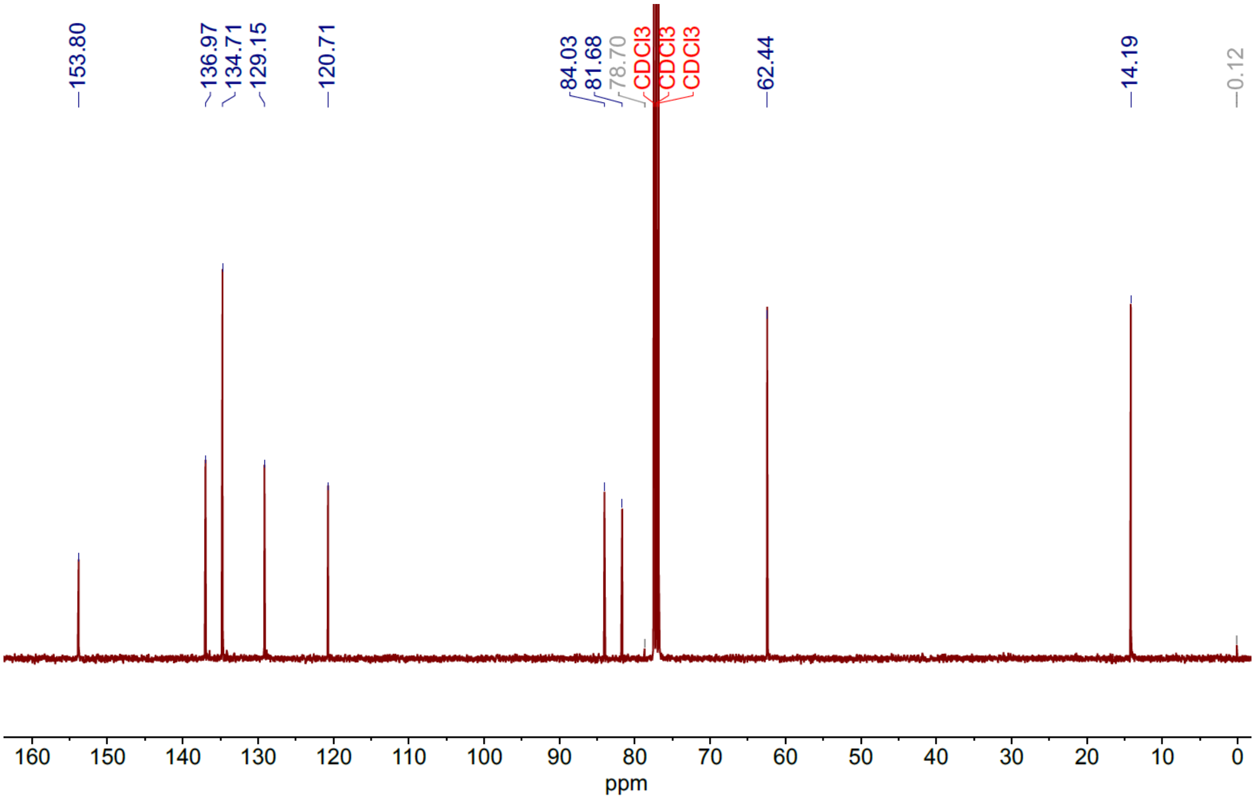

Figure S108. ^{13}C-NMR spectrum of *diethyl 3,3'-(1,3-phenylene)dipropiolate* in $CDCl_3$ (101MHz)

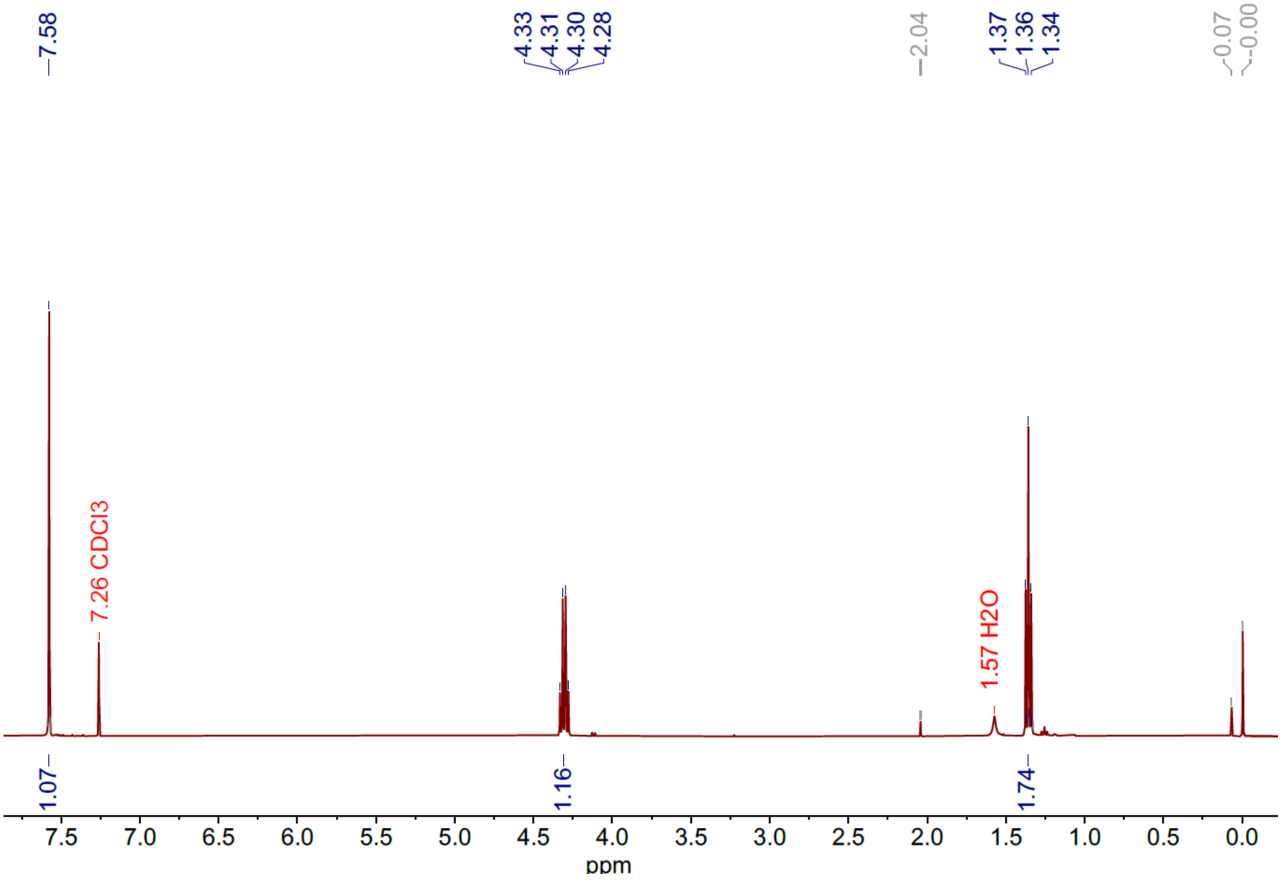

Figure S109. ^{1}H-NMR spectrum of *diethyl 3,3'-(1,4-phenylene)dipropiolate* in $CDCl_3$ (400 MHz)

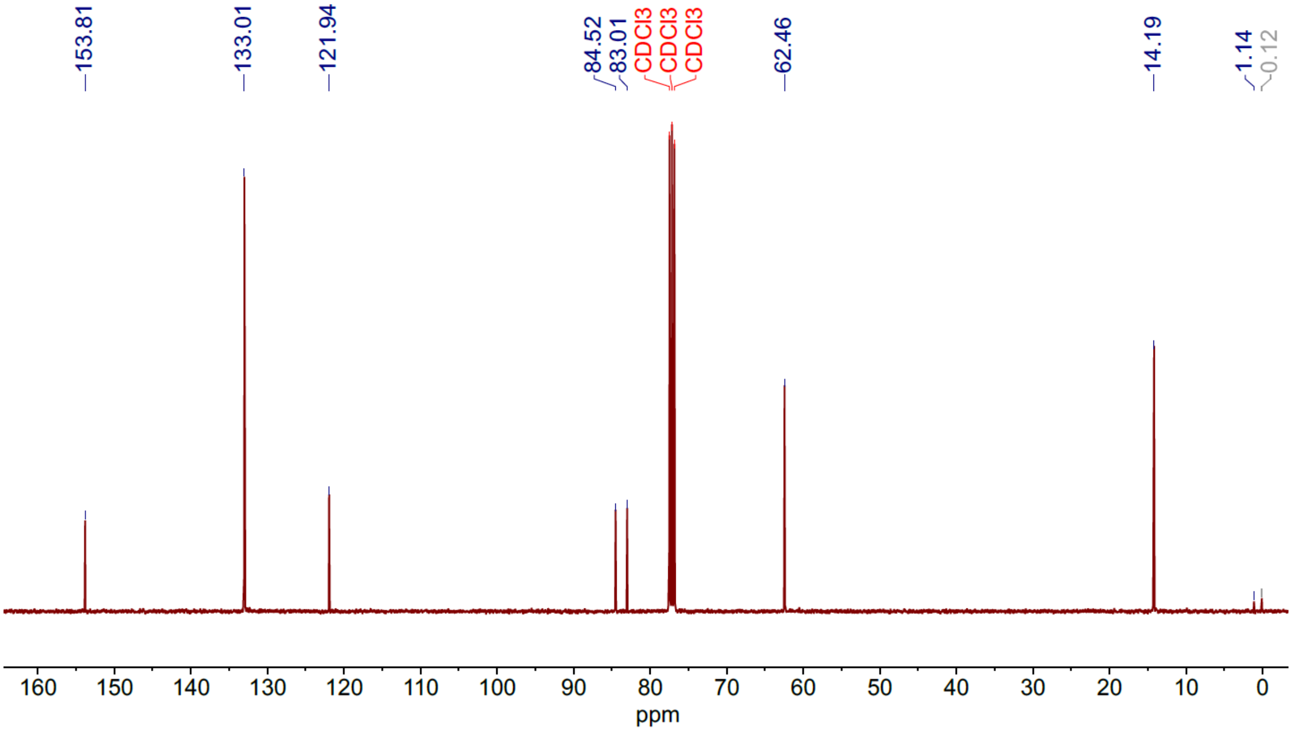

Figure S110. ^{13}C-NMR spectrum of *diethyl 3,3'-(1,3-phenylene)dipropiolate* in $CDCl_3$ (101 MHz)

Summary of DFT calculations.

Methods:

DFT calculations were performed in Gaussian-09 using the unrestricted B3LYP method with dispersion corrections at the GD3 level with Becke-Johnson damping included. The basis set used LANL2TZ+ with core potential on Cu and 6-311+G* on the rest of the atoms. The conductor-type polarized continuum model (CPCM) with a dielectric constant mimicking acetonitrile was used during geometry optimizations and frequency calculations.

DFT calculations show that in **Cu-Pr**, the substrate binds the copper complex through a weak interaction of the π-cloud of the triple bond with copper. This weak interaction will cost little free energy to break, and consequently, the stability of **Cu-Pr** will be limited. Furthermore, the relative free energy of **Cu-S** and **Cu-A** supports the endergonic nature of the **Cu-S** to **Cu-A** conversion.

Table S3: Absolute energies and free energies (au) for the optimized geometries.

	E	ZPE	G
^{1}[Cu-L*]^{0}	-1208.76081	0.405813	-1208.4099
^{1}[Cu-A]$^{-}$	-1516.76891	0.503402	-1516.3333
^{1}[Cu-Pr]$^{-}$	-1705.46713	0.518803	-1705.018
^{1}[Cu-S]^{0}	-1825.87615	0.633651	-1825.3188
^{1}[Phenylpropiolate]$^{-}$	-496.685728	0.111951	-496.61051
^{1}[Phenylacetylide]^{0}	-308.494975	0.109077	-308.41631

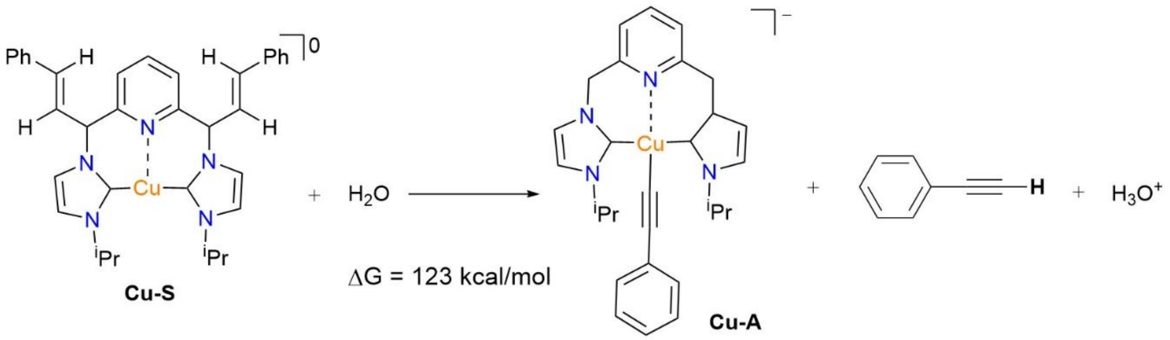

Fig. S111. Calculated dissociation free energy for the conversion of **Cu-S** into **Cu-A**, phenylacetylene, and a hydrogen ion.

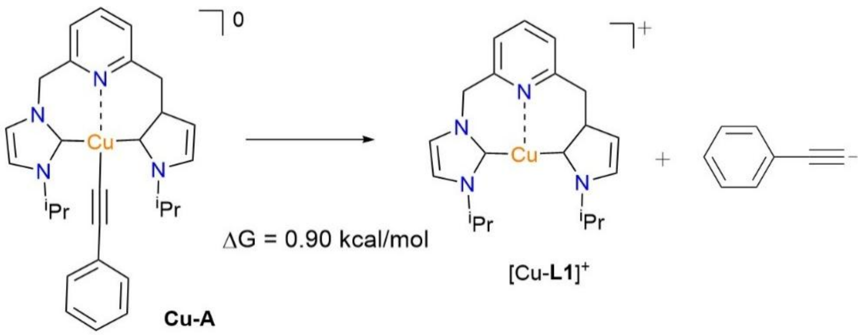

Fig. S112. Calculated dissociation free energy for the conversion of **Cu-A** into the starting complex and acetylide ion.

Cartesian coordinates:

^{1}[Cu-L*]^{0}:

29	2.878573000	4.914437000	2.810878000
7	1.342764000	3.473298000	3.273002000
7	4.016332000	2.274598000	2.523599000
7	5.630010000	3.722402000	2.452767000
7	0.428879000	6.353253000	3.171122000
7	1.671605000	7.516593000	1.862881000
6	4.273541000	3.618573000	2.610495000
6	1.692726000	6.425524000	2.672038000
6	6.589456000	5.409431000	0.934413000
1	7.273356000	4.705804000	0.451741000
1	7.038447000	6.404563000	0.887136000
1	5.655763000	5.426610000	0.367723000
6	6.326730000	5.016475000	2.387976000
1	5.614079000	5.722516000	2.818558000
6	7.594377000	5.006437000	3.237473000
1	7.376181000	4.703173000	4.263847000
1	8.027913000	6.008510000	3.261380000
1	8.352195000	4.330904000	2.832108000
6	6.206156000	2.486049000	2.256008000
1	7.262936000	2.344786000	2.107061000
6	5.200757000	1.573165000	2.305507000
1	5.220155000	0.499991000	2.215520000
6	2.769994000	1.611650000	2.595002000
1	2.841595000	0.561259000	2.353792000
6	1.537793000	2.145429000	2.925193000
6	0.387969000	1.262962000	2.920851000
1	0.534347000	0.220339000	2.656216000
6	-0.849822000	1.731563000	3.241500000
1	-1.705805000	1.063419000	3.229901000
6	-1.018208000	3.098224000	3.604656000
1	-1.988337000	3.492536000	3.881515000
6	0.104889000	3.896720000	3.612299000
6	-0.004749000	5.322226000	4.120102000
1	-1.036416000	5.542730000	4.386854000
1	0.603228000	5.426585000	5.022458000
6	-0.366546000	7.376175000	2.682444000
1	-1.397972000	7.496268000	2.969063000
6	0.417493000	8.110315000	1.851285000
1	0.188899000	8.988342000	1.272385000

6	2.826152000	7.935296000	1.047831000
1	3.661673000	7.371834000	1.466829000
6	2.628011000	7.515653000	-0.407224000
1	2.457939000	6.439029000	-0.476731000
1	3.516585000	7.762244000	-0.992910000
1	1.773633000	8.030369000	-0.855241000
6	3.097155000	9.428639000	1.204357000
1	2.296024000	10.035607000	0.774643000
1	4.021224000	9.688653000	0.683953000
1	3.209041000	9.699158000	2.256524000

^{1}[Cu-A]$^{-}$:

29	3.656054000	5.638083000	3.038540000
7	2.185281000	4.414209000	4.387854000
7	3.841911000	2.640716000	2.610041000
7	4.887911000	3.681513000	1.031844000
7	0.987398000	6.802624000	3.342221000
7	1.294267000	6.747000000	1.223653000
6	4.151352000	3.901490000	2.168085000
6	1.950771000	6.568195000	2.403594000
6	5.286992000	4.636585000	-1.216059000
1	5.803939000	3.768677000	-1.634289000
1	5.662166000	5.522872000	-1.732640000
1	4.222532000	4.537381000	-1.434985000
6	5.529153000	4.770348000	0.285249000
1	5.032128000	5.670659000	0.651661000
6	7.015346000	4.860409000	0.632656000
1	7.150491000	4.976641000	1.710150000
1	7.470990000	5.719817000	0.134202000
1	7.550282000	3.961508000	0.313066000
6	5.050420000	2.331732000	0.776578000
1	5.602024000	1.949853000	-0.065711000
6	4.387141000	1.675727000	1.762162000
1	4.245736000	0.620951000	1.931270000
6	3.103034000	2.254471000	3.763946000
1	3.154246000	1.191294000	3.952627000
6	2.309726000	3.049265000	4.568818000
6	1.562820000	2.439677000	5.654717000
1	1.699077000	1.380958000	5.856462000
6	0.682617000	3.180885000	6.390588000
1	0.115613000	2.710365000	7.189626000

6	0.496454000	4.561687000	6.105532000
1	-0.217149000	5.167339000	6.651532000
6	1.291792000	5.101926000	5.106695000
6	1.214066000	6.579997000	4.769362000
1	0.412833000	7.067235000	5.324520000
1	2.162273000	7.057988000	5.023495000
6	-0.242371000	7.081285000	2.763035000
1	-1.126257000	7.286786000	3.344432000
6	-0.048894000	7.053831000	1.420808000
1	-0.736657000	7.236425000	0.612520000
6	1.923429000	6.517050000	-0.084223000
1	2.981643000	6.399125000	0.148743000
6	1.402982000	5.218282000	-0.698996000
1	1.598699000	4.374010000	-0.034435000
1	1.894090000	5.026043000	-1.655493000
1	0.326371000	5.272289000	-0.882403000
6	1.746324000	7.720978000	-1.005926000
1	0.698536000	7.872043000	-1.279269000
1	2.309273000	7.566333000	-1.929141000
1	2.112220000	8.633614000	-0.530132000
6	4.872560000	6.697401000	4.160666000
6	5.607104000	7.394138000	4.869068000
6	6.443035000	8.203526000	5.689346000
6	6.394725000	8.115768000	7.098274000
6	7.350691000	9.127810000	5.126371000
6	7.210101000	8.910574000	7.897761000
6	8.163856000	9.919961000	5.930715000
6	8.100939000	9.818947000	7.322086000
1	5.705963000	7.413647000	7.555967000
1	7.407515000	9.214989000	4.046532000
1	7.149935000	8.820825000	8.977977000
1	8.851426000	10.621629000	5.468684000
1	8.735546000	10.437852000	7.947277000

^{1}[Cu-Pr]$^{-}$:

29	-2.485741000	0.904275000	0.504731000
7	-3.376365000	-0.167988000	2.151414000
7	-2.542989000	-1.893616000	-0.203706000
7	-1.374395000	-0.844712000	-1.699949000
7	-3.493219000	2.844776000	2.357678000
7	-3.435065000	3.782189000	0.427819000

6	-2.069598000	-0.650822000	-0.534668000
6	-3.060230000	2.651366000	1.082961000
6	-1.838047000	0.901026000	-3.376612000
1	-2.168356000	0.192666000	-4.141828000
1	-1.424395000	1.779175000	-3.878973000
1	-2.707969000	1.213995000	-2.797011000
6	-0.786286000	0.269698000	-2.464322000
1	-0.491689000	0.999773000	-1.710153000
6	0.450521000	-0.177373000	-3.235055000
1	1.175633000	-0.660710000	-2.577762000
1	0.930694000	0.694565000	-3.680827000
1	0.199082000	-0.865633000	-4.046348000
6	-1.424654000	-2.164080000	-2.097509000
1	-0.944507000	-2.531462000	-2.987599000
6	-2.149172000	-2.828711000	-1.159688000
1	-2.407702000	-3.871277000	-1.079668000
6	-3.343171000	-2.267399000	0.901020000
1	-3.659055000	-3.299413000	0.853762000
6	-3.741491000	-1.493249000	1.976701000
6	-4.578384000	-2.111809000	2.985424000
1	-4.853625000	-3.155103000	2.864626000
6	-5.011702000	-1.398402000	4.062083000
1	-5.643644000	-1.869390000	4.809489000
6	-4.625184000	-0.037380000	4.211874000
1	-4.942101000	0.548144000	5.066359000
6	-3.804246000	0.501793000	3.242455000
6	-3.245647000	1.896241000	3.450241000
1	-3.672698000	2.330816000	4.352311000
1	-2.167013000	1.821245000	3.597864000
6	-4.127075000	4.068574000	2.496613000
1	-4.528084000	4.410810000	3.436104000
6	-4.096589000	4.660633000	1.274531000
1	-4.477736000	5.613934000	0.951884000
6	-3.214098000	3.975209000	-1.015866000
1	-2.420177000	3.266930000	-1.258193000
6	-4.468756000	3.598428000	-1.801093000
1	-4.765580000	2.571163000	-1.578968000
1	-4.280281000	3.678532000	-2.874263000
1	-5.302634000	4.260492000	-1.552123000
6	-2.735161000	5.392287000	-1.316289000
1	-3.524558000	6.131981000	-1.160427000

1	-2.426392000	5.458404000	-2.361401000
1	-1.884045000	5.665347000	-0.689209000
6	0.316936000	0.687402000	2.782043000
6	0.635891000	1.268329000	1.768352000
6	1.017117000	1.922100000	0.561191000
6	0.362704000	3.093117000	0.139777000
6	2.044631000	1.392328000	-0.238721000
6	0.721131000	3.705011000	-1.056185000
6	2.406680000	2.018412000	-1.426489000
6	1.743052000	3.171998000	-1.842987000
1	-0.436817000	3.496965000	0.746894000
1	2.547703000	0.485270000	0.076271000
1	0.202318000	4.600729000	-1.378510000
1	3.197106000	1.595310000	-2.036424000
1	2.017196000	3.651145000	-2.776519000
6	-0.080697000	-0.032394000	4.011235000
8	-0.419724000	0.685395000	4.985400000
8	-0.035216000	-1.286561000	3.953462000

^{1}[Cu-S]^{0}:

29	2.788235000	4.770085000	2.469996000
7	1.273533000	3.308758000	3.654163000
7	3.733054000	2.119203000	3.131912000
7	5.348864000	3.530687000	3.375710000
7	0.371834000	6.196400000	3.088815000
7	1.053551000	6.886952000	1.173195000
6	4.090612000	3.412257000	2.878471000
6	1.413041000	6.101220000	2.215377000
6	7.501966000	4.608652000	2.815073000
1	8.119096000	3.972040000	3.454155000
1	7.999959000	5.577226000	2.736010000
1	7.461168000	4.161638000	1.819489000
6	6.099989000	4.798512000	3.387333000
1	5.530100000	5.452964000	2.725018000
6	6.106274000	5.399135000	4.791926000
1	5.085771000	5.532801000	5.157833000
1	6.598705000	6.374210000	4.781178000
1	6.643704000	4.757188000	5.495084000
6	5.778049000	2.340446000	3.941272000
1	6.745082000	2.223243000	4.399108000
6	4.759490000	1.455818000	3.791190000

1	4.674945000	0.423461000	4.084667000
6	2.505824000	1.464527000	2.721631000
6	1.269206000	2.044577000	3.133752000
6	0.015916000	1.369508000	3.014604000
1	-0.019422000	0.332881000	2.717976000
6	-1.153376000	2.025939000	3.332535000
1	-2.102068000	1.506444000	3.239516000
6	-1.123774000	3.356384000	3.782083000
1	-2.032287000	3.903010000	3.995665000
6	0.127730000	3.931000000	3.950115000
6	0.333436000	5.393179000	4.337820000
1	1.352173000	5.458805000	4.730419000
6	-0.633767000	7.003336000	2.584772000
1	-1.549785000	7.196260000	3.115977000
6	-0.199865000	7.446495000	1.376566000
1	-0.669850000	8.104398000	0.666147000
6	1.875806000	7.056001000	-0.040191000
1	2.819226000	6.566753000	0.208365000
6	1.231949000	6.329077000	-1.218769000
1	1.072853000	5.274768000	-0.982365000
1	1.880942000	6.391691000	-2.094926000
1	0.268166000	6.773231000	-1.481420000
6	2.135641000	8.533725000	-0.318903000
1	1.217035000	9.063585000	-0.583469000
1	2.827207000	8.632334000	-1.158108000
1	2.578983000	9.023783000	0.550474000
6	-0.588854000	6.011748000	5.347838000
1	-0.579115000	7.097926000	5.353545000
6	2.718436000	0.333143000	1.898587000
1	3.778341000	0.124985000	1.767582000
6	-1.289400000	5.342565000	6.268845000
1	-1.262783000	4.257063000	6.254813000
6	1.879543000	-0.508196000	1.204752000
1	0.810532000	-0.339082000	1.192097000
6	-2.117516000	5.923026000	7.334173000
6	-2.801379000	5.053789000	8.198555000
6	-2.267144000	7.306466000	7.532267000
6	-3.607781000	5.543178000	9.223883000
1	-2.698064000	3.981845000	8.061619000
6	-3.072316000	7.795882000	8.553943000
1	-1.752541000	8.008943000	6.886448000

6	-3.747214000	6.917498000	9.405917000
1	-4.125944000	4.850863000	9.878813000
1	-3.174385000	8.867413000	8.689287000
1	-4.373780000	7.303599000	10.202551000
6	2.318768000	-1.618949000	0.380213000
6	3.652245000	-2.090905000	0.315028000
6	1.370700000	-2.303164000	-0.417953000
6	4.007118000	-3.156824000	-0.503523000
1	4.422318000	-1.624888000	0.920340000
6	1.727857000	-3.371845000	-1.232421000
1	0.334711000	-1.976048000	-0.392393000
6	3.052914000	-3.811419000	-1.288552000
1	5.041574000	-3.487320000	-0.524132000
1	0.966585000	-3.865527000	-1.829329000
1	3.334762000	-4.645305000	-1.922325000

^{1}[Phenylpropiolate]$^{-}$:

6	0.306856000	0.671116000	2.772429000
6	0.629085000	1.229911000	1.746712000
6	1.006893000	1.889284000	0.540012000
6	0.401861000	3.103914000	0.169234000
6	1.991001000	1.336682000	-0.299646000
6	0.773036000	3.745028000	-1.008026000
6	2.357071000	1.983014000	-1.475719000
6	1.750530000	3.187848000	-1.833711000
1	-0.357285000	3.535387000	0.811542000
1	2.461501000	0.400948000	-0.020207000
1	0.298902000	4.681173000	-1.282164000
1	3.117129000	1.546240000	-2.114371000
1	2.037920000	3.689785000	-2.751124000
6	-0.082020000	-0.012471000	4.027052000
8	-0.982941000	0.545563000	4.701968000
8	0.530471000	-1.078866000	4.284807000

^{1}[Phenylacetylide]^{0}:

6	-0.546907000	5.426157000	5.308121000
1	-0.007398000	6.038178000	4.622930000
6	-1.157610000	4.733136000	6.083800000
6	-1.880879000	3.912953000	7.001606000
6	-1.820900000	2.512205000	6.903569000
6	-2.661718000	4.496203000	8.014003000

6	-2.527350000	1.716733000	7.799600000
1	-1.219962000	2.057962000	6.124289000
6	-3.365080000	3.693222000	8.905997000
1	-2.711161000	5.575992000	8.093669000
6	-3.300535000	2.303331000	8.802221000
1	-2.474287000	0.636894000	7.715145000
1	-3.964662000	4.153470000	9.683604000
1	-3.850035000	1.680308000	9.499275000

References

1. D. Domyati, S. L. Hope, R. Latifi, M. D. Hearns and L. Tahsini, *Inorg. Chem.*, 2016, 55, 11685-11693.
2. J. L. Minnick, J. Raincrow, G. Meinders, R. Latifi and L. Tahsini, *Inorg. Chem.*, 2023, 62, 15912-15926.
3. Bruker (2021). APEX4, SAINT and SADABS. Bruker AXS Inc., Madison, Wisconsin, USA.
4. Sheldrick, G. M. (2015) *Acta Crystallogr.*, A**71**, 3-8.
5. Sheldrick, G. M. (2015) *Acta Crystallogr.*, C**71**, 3-8.
6. Dolomanov, O. V.; Bourhis, . L. J.; Gildea, R. J.; Howard, J. A. K.; Puschmann. H. (2009) *J. Appl. Cryst*. **42**, 339-341.

1. D. Domyati, S. L. Hope, R. Latifi, M. D. Hearns and L. Tahsini, Cu(I) complexes of pincer pyridine-based N-heterocyclic carbenes with small wingtip substituents: synthesis and structural and spectroscopic studies, *Inorg. Chem.*, 2016, **55**, 11685-11693.
2. J. L. Minnick, J. Raincrow, G. Meinders, R. Latifi and L. Tahsini, Synthesis, characterization, and spectroscopic studies of 2,6-dimethylpyridyl-linked Cu(I)–CNC complexes: The electronic influence of aryl wingtips on copper centers, *Inorg. Chem.*, 2023, **62**, 15912-15926.
3. S. a. S. B. A. I. Bruker (2021). APEX4, Madison, Wisconsin, USA.
4. G. M. Sheldrick, *Acta Crystallogr.*, 2015, **A71**, 3-8.
5. G. M. Sheldrick, *Acta Crystallogr.*, 2015, **C71**, 3-8.
6. O. V. B. Dolomanov, . L. J.; Gildea, R. J.; Howard, J. A. K.; Puschmann. H. , *J. Appl. Cryst.* , 2009, **42**, 339-341.
7. G. te Velde, F. M. Bickelhaupt, E. J. Baerends, C. Fonseca Guerra, S. J. A. van Gisbergen, J. G. Snijders and T. Ziegler, Chemistry with ADF, *Journal of Computational Chemistry*, 2001, **22**, 931-967.
8. S. ADF 2023.1, Theoretical Chemistry, Vrije Universiteit, Amsterdam, The Netherlands, http://www.scm.com. Optionally, you may add the following list of authors and contributors: E.J. Baerends, T. Ziegler, A.J. Atkins, J. Autschbach, O. Baseggio, D. Bashford, A. Bérces, F.M. Bickelhaupt, C. Bo, P.M. Boerrigter, C. Cappelli, L. Cavallo, C. Daul, D.P. Chong, D.V. Chulhai, L. Deng, R.M. Dickson, J.M. Dieterich, F. Egidi, D.E. Ellis, M. van Faassen, L. Fan, T.H. Fischer, A. Förster, C. Fonseca Guerra, M. Franchini, A. Ghysels, A. Giammona, S.J.A. van Gisbergen, A. Goez, A.W. Götz, J.A. Groeneveld, O.V. Gritsenko, M. Grüning, S. Gusarov, F.E. Harris, P. van den Hoek, Z. Hu, C.R. Jacob, H. Jacobsen, L. Jensen, L. Joubert, J.W. Kaminski, G. van Kessel, C. König, F. Kootstra, A. Kovalenko, M.V. Krykunov, P. Lafiosca, E. van Lenthe, D.A. McCormack, M. Medves, A. Michalak, M. Mitoraj, S.M. Morton, J. Neugebauer, V.P. Nicu, L. Noodleman, V.P. Osinga, S. Patchkovskii, M. Pavanello, C.A. Peeples, P.H.T. Philipsen, D. Post, C.C. Pye, H. Ramanantoanina, P. Ramos, W. Ravenek, M. Reimann, J.I. Rodríguez, P. Ros, R. Rüger, P.R.T. Schipper, D. Schlüns, H. van Schoot, G. Schreckenbach, J.S. Seldenthuis, M. Seth, J.G. Snijders, M. Solà, M. Stener, M. Swart, D. Swerhone, V. Tognetti, G. te Velde, P. Vernooijs, L. Versluis, L. Visscher, O. Visser, F. Wang, T.A. Wesolowski, E.M. van Wezenbeek, G. Wiesenekker, S.K. Wolff, T.K. Woo, A.L. Yakovlev.
